# Identification of anoikis-related genes classification patterns and immune infiltration characterization in ischemic stroke based on machine learning

**DOI:** 10.3389/fnagi.2023.1142163

**Published:** 2023-03-23

**Authors:** Xiaohong Qin, Shangfeng Yi, Jingtong Rong, Haoran Lu, Baowei Ji, Wenfei Zhang, Rui Ding, Liquan Wu, Zhibiao Chen

**Affiliations:** ^1^Department of Neurosurgery, Renmin Hospital of Wuhan University, Wuhan, Hubei, China; ^2^Central Laboratory, Renmin Hospital of Wuhan University, Wuhan, Hubei, China; ^3^Department of Neurosurgery, Enshi Center Hospital, Enshi, Hubei, China; ^4^Department of Psychiatry, Renmin Hospital of Wuhan University, Wuhan, Hubei, China

**Keywords:** ischemic stroke, anoikis, immune infiltration, molecular cluster, machine learning, immunotherapy

## Abstract

**Introduction:**

Ischemic stroke (IS) is a type of stroke that leads to high mortality and disability. Anoikis is a form of programmed cell death. When cells detach from the correct extracellular matrix, anoikis disrupts integrin junctions, thus preventing abnormal proliferating cells from growing or attaching to an inappropriate matrix. Although there is growing evidence that anoikis regulates the immune response, which makes a great contribution to the development of IS, the role of anoikis in the pathogenesis of IS is rarely explored.

**Methods:**

First, we downloaded GSE58294 set and GSE16561 set from the NCBI GEO database. And 35 anoikis-related genes (ARGs) were obtained from GSEA website. The CIBERSORT algorithm was used to estimate the relative proportions of 22 infiltrating immune cell types. Next, consensus clustering method was used to classify ischemic stroke samples. In addition, we used least absolute shrinkage and selection operator (LASSO), support vector machine-recursive feature elimination (SVM-RFE) and random forest (RF) algorithms to screen the key ARGs in ischemic stroke. Next, we performed receiver operating characteristics (ROC) analysis to assess the accuracy of each diagnostic gene. At the same time, the nomogram was constructed to diagnose IS by integrating trait genes. Then, we analyzed the correlation between gene expression and immune cell infiltration of the diagnostic genes in the combined database. And gene ontology (GO) and kyoto encyclopedia of genes and genomes (KEGG) analysis were performed on these genes to explore differential signaling pathways and potential functions, as well as the construction and visualization of regulatory networks using NetworkAnalyst and Cytoscape. Finally, we investigated the expression pattern of ARGs in IS patients across age or gender.

**Results:**

Our study comprehensively analyzed the role of ARGs in IS for the first time. We revealed the expression profile of ARGs in IS and the correlation with infiltrating immune cells. And The results of consensus clustering analysis suggested that we can classify IS patients into two clusters. The machine learning analysis screened five signature genes, including AKT1, BRMS1, PTRH2, TFDP1 and TLE1. We also constructed nomogram models based on the five risk genes and evaluated the immune infiltration correlation, gene-miRNA, gene-TF and drug-gene interaction regulatory networks of these signature genes. The expression of ARGs did not differ by sex or age.

**Discussion:**

This study may provide a beneficial reference for further elucidating the pathogenesis of IS, and render new ideas for drug screening, individualized therapy and immunotherapy of IS.

## Introduction

Stroke, the second leading cause of death and disability worldwide, causes 5.5 million deaths annually (Lindsay et al., 2019). Ischemic stroke (IS) accounts for about 80% of all strokes (Saini et al., 2021). Although tPA is the primary treatment option for IS, an army of patients with IS are at high risk of severe brain injury due to narrow treatment time windows, reperfusion injury, and rebleeding complications (Fukuta et al., 2017; Ajoolabady et al., 2021). Previous studies have shown that IS produces an immune response that leads to neuronal loss and tissue repair, while neuron loss may also lead to disruption of immune homeostasis after stroke (Iadecola et al., 2020; DeLong et al., 2022). Moreover, IS is usually accompanied by changes in immune cells, including macrophages, mast cells, neutrophils, monocytes, T cells, natural killer T cells, gamma delta T Cells, Tregs, B cells (DeLong et al., 2022). However, the mechanism of immune response behind IS is largely unknown. Identification of novel characteristic genes may provide potential therapeutic targets or etiological insights for IS.

Anoikis, a form of programmed cell death, is essentially a process of apoptosis. When cells detach from the correct extracellular matrix, anoikis disrupts integrin junctions, thus preventing abnormal proliferating cells from growing or attaching to an inappropriate matrix (Taddei et al., 2012). Anoikis is characterized by anchoring growth and epithelial-mesenchymal transformation, which is not only important for tissue homeostasis and development, but also plays a critical regulatory role in metastatic cancer, cardiovascular disease and diabetes (Taddei et al., 2012). Studies have shown that various factors are related to the mechanism of anoikis, including integrins, E-calmodulin, EGFR, IGFR, Trk, TGF-β, hippo pathway, NF-κB, eEF-2 kinase, hypoxia, acidosis, ROS, HP and protective autophagy (Adeshakin et al., 2021; Zhu et al., 2022). Similarly, the main pathological damage of IS is the oxidative stress and excitatory amino acid toxicity response induced by hypoxia, resulting in the damage of neurons, glial cells and vascular endothelial cells, and blood–brain barrier (BBB) disruption. The continuous ischemia and hypoxia and the gradual neuroinflammatory response further aggravate the tissue and cell damage, until apoptosis. This is manifested by the massive release of inflammatory cytokines, chemokines, cell adhesion molecules and matrix metalloproteinases (Lakhan et al., 2009; Ceulemans et al., 2010; Iadecola and Anrather, 2011). Therefore, anoikis may play a crucial role in the occurrence and development of IS. Many studies have shown that anoikis is involved in the pathogenesis of several diseases, especially tumor immunity, such as glioblastoma (Sun et al., 2022), head and neck squamous cell carcinoma (Chi et al., 2022a) and lung adenocarcinoma (Diao et al., 2022). Despite growing evidence shows that anoikis regulates immune response, which plays a key role in the development of IS. However, the role of anoikis in the pathogenesis of IS is rarely explored. As a result, a thorough study of the different immune characteristics between normal tissues and IS specimens, as well as the different subtypes of IS, will help to elucidate the changes occurring in anoikis and its related genes. Meanwhile, the establishment of characteristics related to anoikis will provide new clew for individualized treatment of IS patients.

In this study, we comprehensively analyzed the differential expression of ARGs and immune profiles in normal and IS peripheral blood samples for the first time. We also performed consensus clustering, immune infiltration analysis and functional enrichment analysis of IS samples using anoikis differentially expressed genes (DEGs). And we used three machine learning (ML) algorithms to screen five risk signature genes that could be used to predict disease onset. We also constructed nomogram models. In addition, the immune infiltration correlation, gene-miRNA, gene-TF and drug-gene interaction regulatory networks of the five risk genes were also discussed. Finally, we explored the expression patterns of ARGs in IS patients across age or gender. Our study may supply a slice of theoretical basis to individualized treatment of IS and the development of immunomodulatory treatment protocols.

## Materials and methods

### Data acquisition

**Figure 1:**
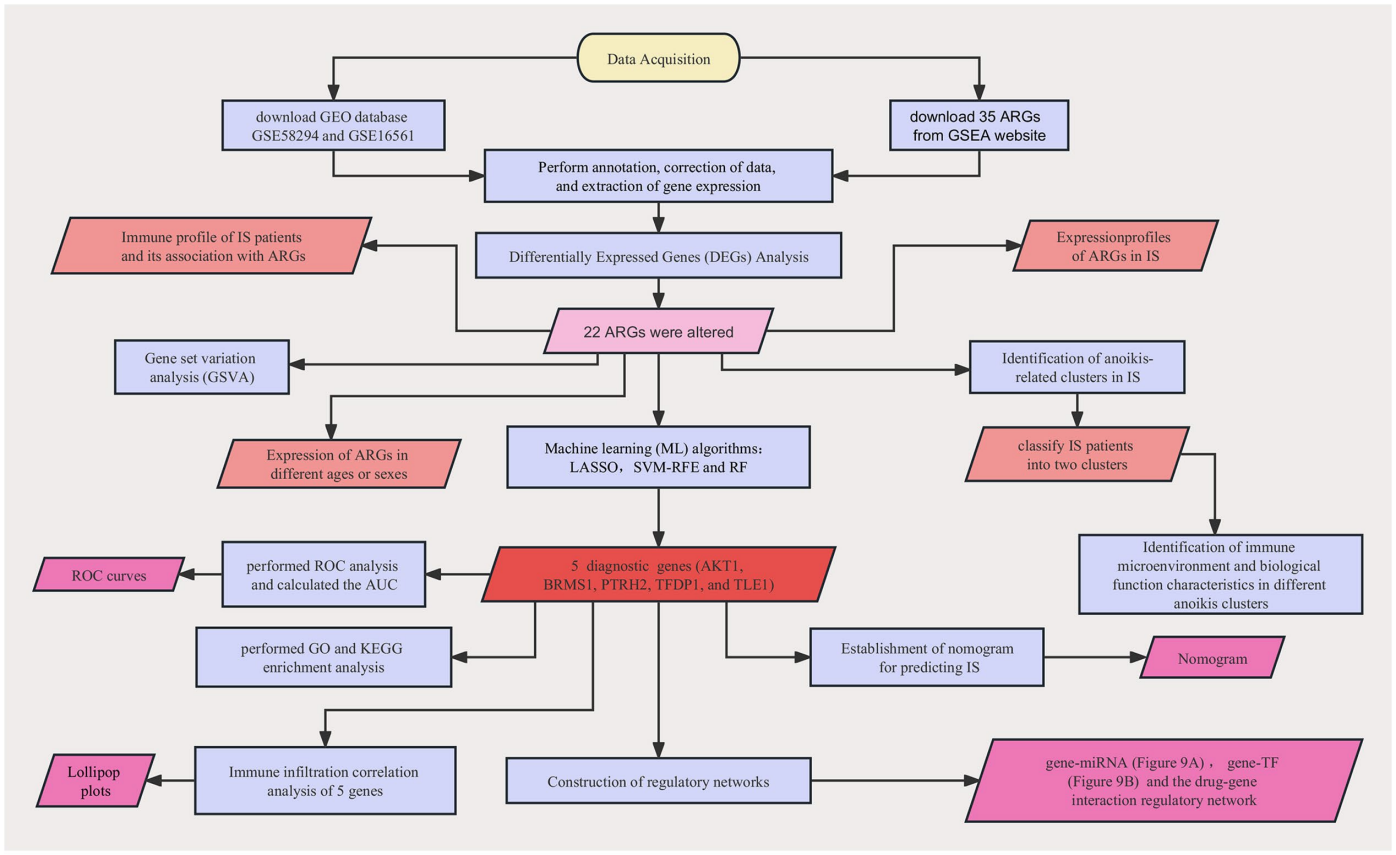
A detailed flow chart about the study of ARGs in IS.

We downloaded the gene expression profiling datasets of two IS-related peripheral blood samples, GSE58294 (GPL570 platform) and GSE16561 (GPL6883 platform), from the NCBI GEO database.^1^ The former included 23 control samples and 69 IS samples as a training set, and the latter included 24 control samples and 39 IS samples as the validation set. The raw GEO data were normalized using the R package “NormalizeBetweenArray.” 35 ARGs were obtained from GSEA website (Yang et al., 2022).^2^ The detailed flow chart is shown in Figure 1.

### Differentially expressed genes analysis

R package “Limma” was utilized to explore Differentially expressed genes (DEGs; Wang X. et al., 2022) between normal samples and IS samples. *p*-value < 0.05 was considered statistically significant.

### Immune cell infiltration profile

The CIBERSORT algorithm was used to estimate the relative proportions of 22 infiltrating immune cell types (Zhao et al., 2023) based on gene expression. *p* < 0.05 was considered statistically significant. And the results were used for further data analysis. We then compared the proportions of immune cells between the different groups by Wilcoxon test. Histograms, heat maps, box plots and violin plots were plotted using the “ggplot2” and “vioplot” R packages. The Pearson correlation coefficient between each immune cell was calculated using the “corrplot” R package, and the results were displayed in the associated heat map.

### Correlation of infiltrating immune cells with ARGs

The correlation coefficient between ARGs expression and the percentage of infiltrating immune cells was calculated, and the results were presented using the R package “ggplot.” *p* < 0.05 was considered as a significant correlation.

### Construction of unsupervised clusters of anoikis and PCA analysis

Unsupervised cluster analysis of ARGs was performed using the “Consens-usClusterPlus” R package to identify different anoikis patterns in IS. The tendency and smoothness of cumulative distribution function (CDF) curve, consensus score and consensus matrix were used to determine the optimal number of subtype k. Principal component analysis (PCA) was conducted by the R package “ggplot2.”

### Gene set variation analysis

We downloaded the”c5.go.symbols” file and”c2.cp.kegg.symbols” file from Gene set variation analysis (GSVA)’s MSigDB database. Then, R packets “GSVA” and “limma” were used to analyze the altered pathways and biological functions (Chi et al., 2022b) between different ARGs-related clusters.

### Machine learning algorithms

We used least absolute shrinkage and selection operator (LASSO; Chi et al., 2022c; Zhao et al., 2022a), support vector machine-recursive feature elimination (SVM-RFE) and random forest (RF) algorithms (Zhao et al., 2022b; Chi et al., 2022d) to screen the key ARGs in DEG obtained between normal samples and IS samples. Then took the intersection of the feature genes screened by the three algorithms, and draw the Venn diagram with “VennDiagram” R packet. Next, the “pROC” R package was used to draw ROC curves to determine the predictive value of these characteristic genes in the training set. At the same time, we used R package “InpROC” to compute the area under the curve (AUC). And the prediction ability of these characteristic genes was validated in the verification set. Finally, we also constructed a nomogram with the R package “rms” based on these signature genes (Cai et al., 2022).

### Gene ontology and Kyoto encyclopedia of genes and genomes analysis

Next, we performed Gene ontology (GO) and Kyoto encyclopedia of genes and genomes (KEGG) enrichment analysis on these genes using the R package “clusterProfiler” to probe the differential signaling pathways and potential functions of the signature genes. *p*-values < 0.05 were considered statistically significant.

### Correlation of immune-infiltrating cells with signature genes

The correlation coefficients between the expression of ARGs and immune infiltrating cells were first computed, and then Spearman’s rank correlation analysis was used to probe the relationship between immune infiltrating cells and the characteristic genes. Finally, Lollipop plots were drawn using the R package “ggplot.”

### Construction of regulatory networks

The regulatory networks of miRNA diagnostic biomarkers and transcription factor (TF) diagnostic biomarkers based on characteristic genes was constructed by NetworkAnalyst.^3^ The file of the interaction between drug and gene was obtained from the drug-gene interaction database (DGIdb),^4^ and then imported into the Cytoscape software for further visualization (Chi et al., 2022a).

## Results

### Expression profiles of ARGs in IS patients

**Figure 2:**
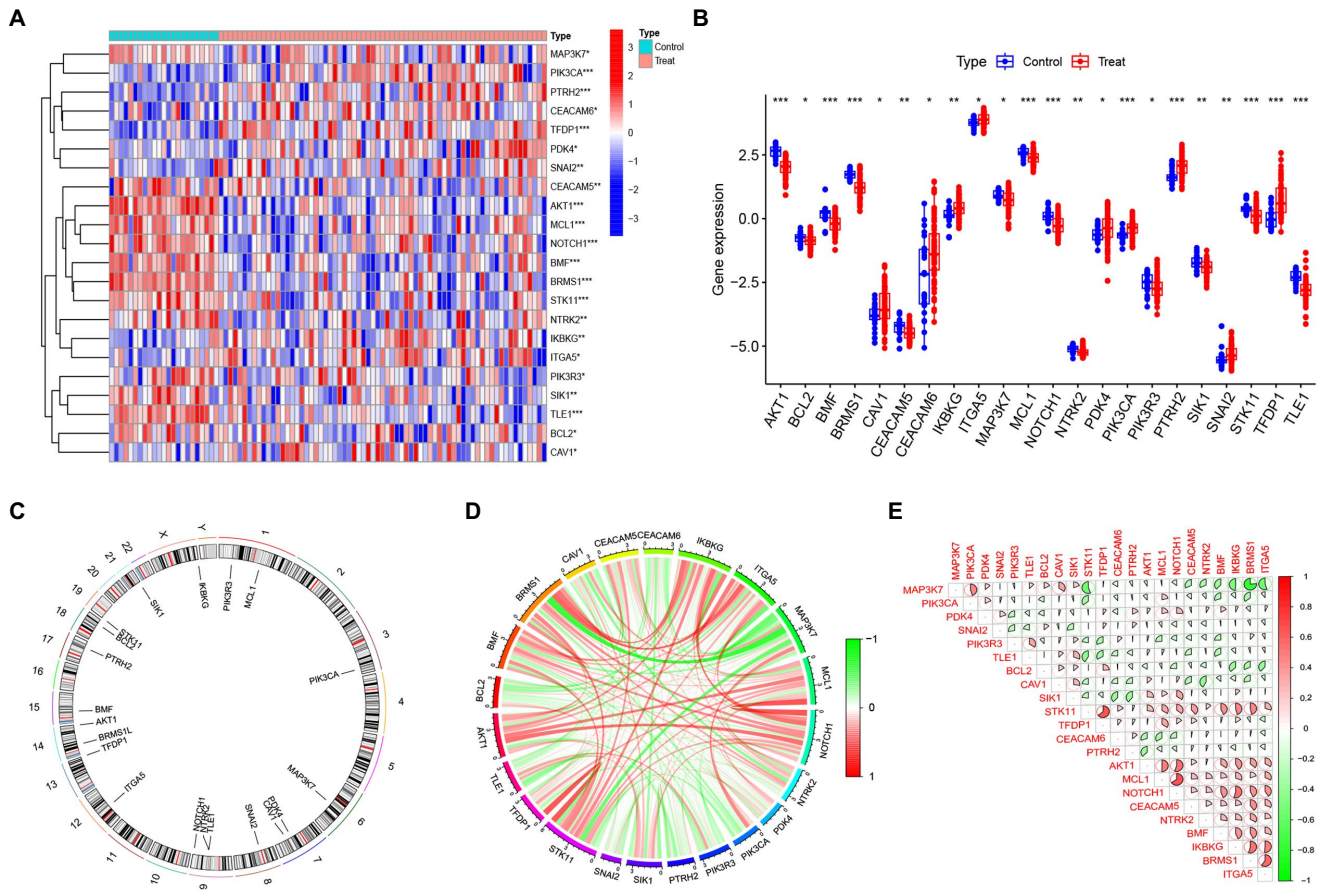
Expression profiles of ARGs in IS. **(A)** Heat map showing the expression of 22 differentially expressed ARGs. **(B)** Box plot showing the expression differences of 22 ARGs between IS and non-IS samples. **(C)** Relative positions of the 22 ARGs on the chromosomes. **(D)** Correlation circle plot showing the correlation of the 22 differentially expressed ARGs. **(E)** Correlation heat map showing the correlation coefficients of the 22 differentially expressed ARGs. Red and green represent positive and negative correlations, respectively. Correlation coefficients are shown as the area of the pie chart. *p < 0.05, **p < 0.01, ***p < 0.001.

In order to explore the role of ARGs in IS, we systematically evaluated the altered expression of ARGs in IS patients through the GSE58294 database. The results showed that the expression profiles of 22 ARGs were altered, of which the expression levels of 9 ARGs (CAV1, CEACAM6, IKBKG, ITGA5, PDK4, PIK3CA, PTRH2, SNAI2, TFDP1) were up-regulated, while 13 ARGs (AKT1, BCL2, BMF, BRMS1, CEACAM5, MAP3K7, MCL1, NOTCH1, NTRK2, PIK3R3, SIK1, STK11, TLE1) were down-regulated (Figures 2A,B). Meanwhile, the chromosome locations of 22 anoikis genes were visualized (Figure 2C). Next, we conducted correlation analysis of these differentially expressed ARGs to explore their interactions. It was obvious that some anoikis regulatory genes, such as STK11 and TFDP1, AKT1 and NOTCH1, MCL1 and NOTCH1, showed strong synergistic effects, while MAP3K7 illustrated significant antagonistic effects with STK11, BRMS1, and ITGA5, respectively (Figures 2D,E).

### Immune profile of IS patients and its association with ARGs

**Figure 3:**
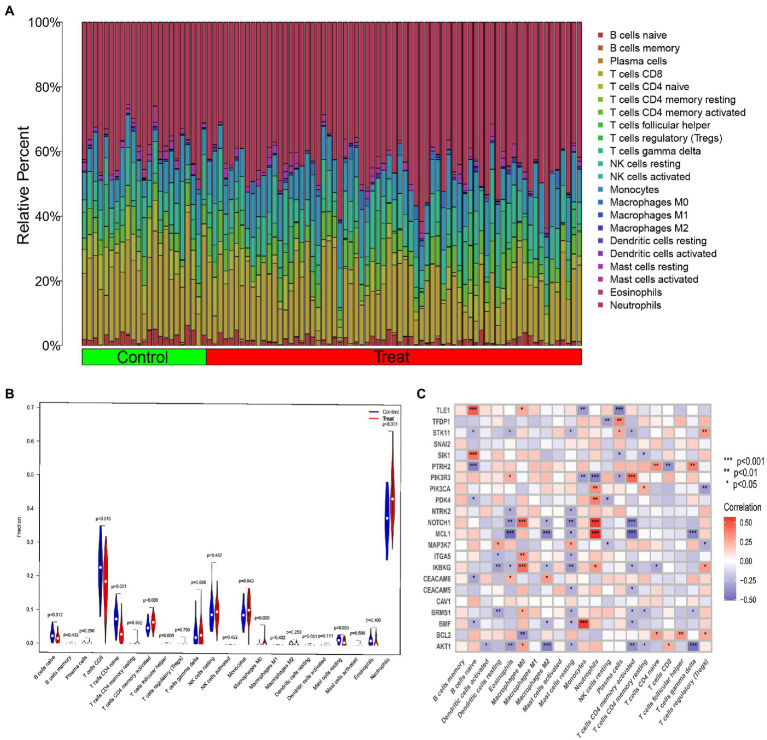
Overview of immune infiltration in IS. **(A)** Relative abundance of 22 infiltrating immune cells between IS and non-IS samples. **(B)** Violin plot showing the difference in immune infiltration between IS and non-IS samples. **(C)** Correlation analysis of 22 differentially expressed ARGs with infiltrating immune cells. *p < 0.05, **p < 0.01, ***p < 0.001.

Based on gene expression, we calculated the difference in the proportion of 22 infiltrating immune cell types in each sample using the CIBERSORT algorithm. The results indicated that activated memory CD4+ T cells, follicular helper T cells, monocytes, M0 macrophages, resting dendritic cells and neutrophils were differentially up-regulated in IS patients, while naive B cells, CD8+ T cells, naive CD4+ T cells and resting mast cells were differentially down-regulated (Figures 3A,B). This means that IS causes changes in the immune system. Meanwhile, correlation analysis demonstrated that naive B cells, eosinophils, M0 macrophages, M2 macrophages, resting mast cells, neutrophils, activated memory CD4+ T cells, plasma cells, and regulatory T cells (Tregs) were closely bound up with anoikis regulators (Figure 3C). These results suggest that ARGs make a great contribution to the alterations of immune infiltration status of IS patients.

### Identification of anoikis-related clusters in IS

**Figure 4:**
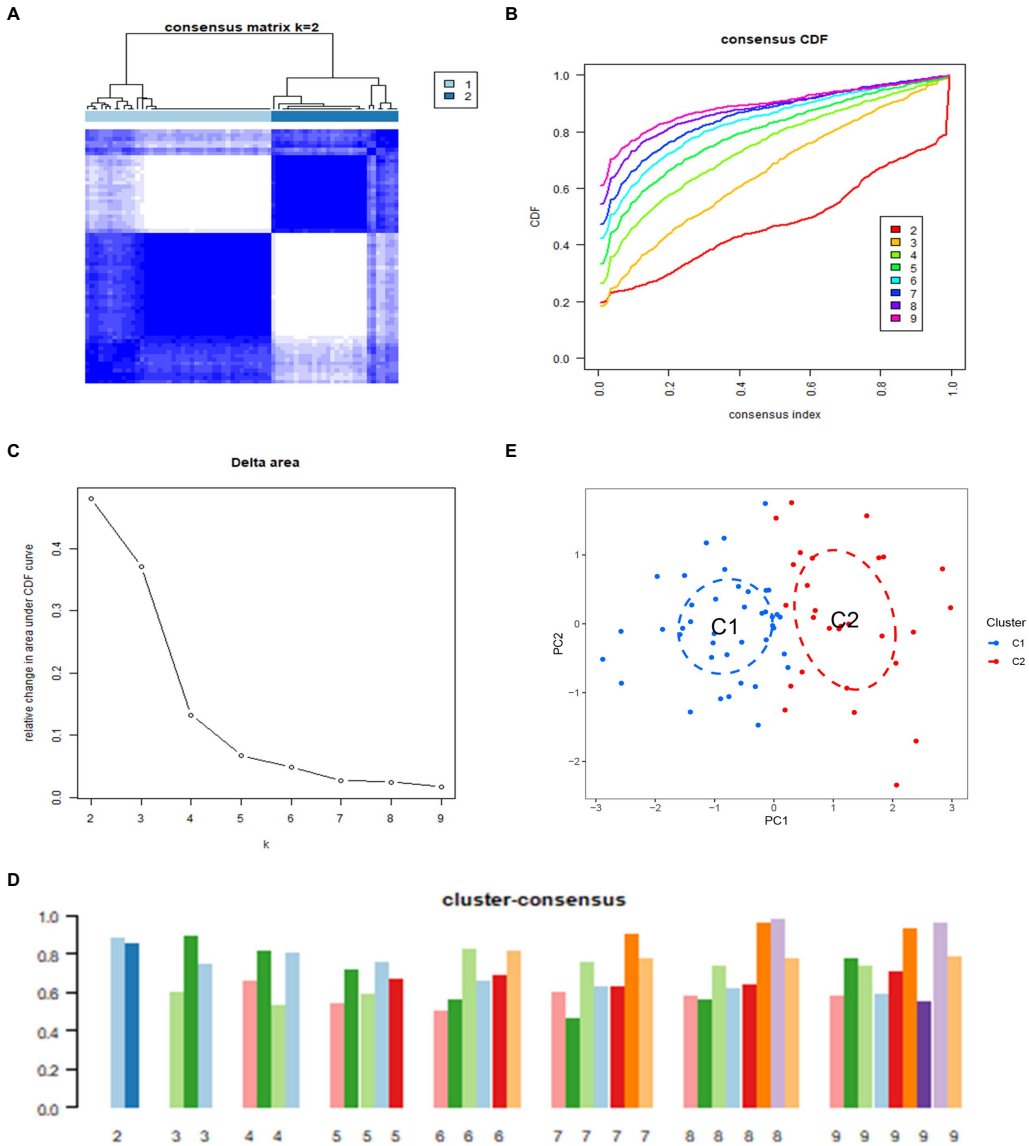
Identification of molecular clusters associated with anoikis in IS. **(A)** Consensus clustering matrix at k = 2. **(B)** Cumulative distribution function (CDF) curves representing k values of 2–9, respectively. **(C)** Representative CDF delta area curves. **(D)** Visualization of the distribution of the two clusters by principal component analysis (PCA). **(E)** Consensus clustering scores for k values of 2–9, respectively.

In order to further clarify the expression profile of ARGs in IS, we used consensus clustering algorithm to group 69 IS samples according to the expression of 22 ARGs. We set the value of k to 2–9 (Supplementary Figure 1), and found that when k = 2, the consensus index of CDF curve fluctuates in the minimum range, and the consensus score is relatively large, indicating that relatively good value of k is 2 (Figures 4A–D). And we validated it in the GSE16561 dataset (Supplementary Figure 2). In addition, the results of principal component analysis (PCA) showed that there were significant differences between the two clusters (Figure 4E). Therefore, we divided 69 IS patients into two clusters, including cluster 1 (*n* = 41) and cluster 2 (*n* = 28).

### Identification of immune microenvironment and biological function characteristics in different anoikis clusters

**Figure 5:**
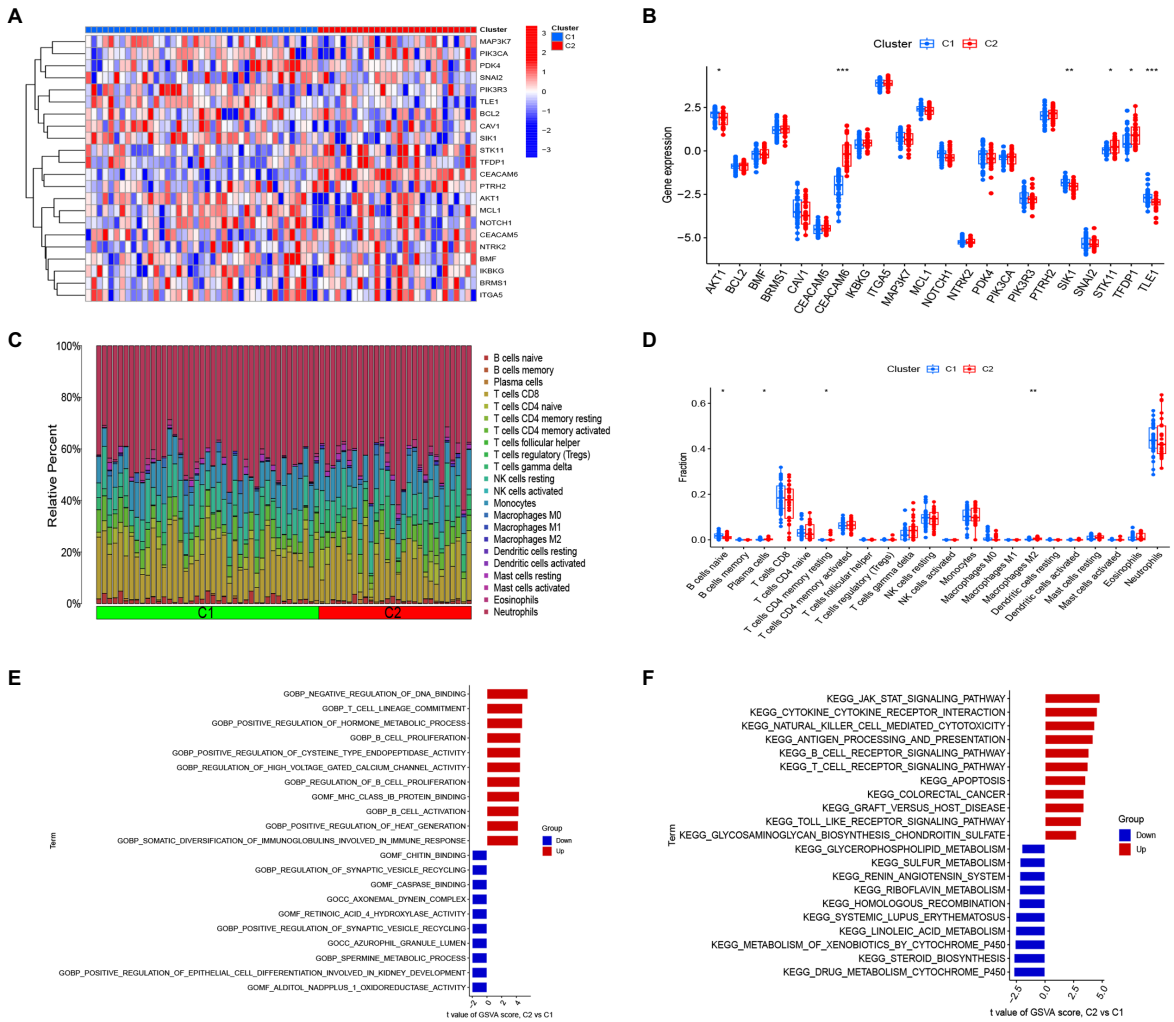
Identification of immune infiltration and biological functional characteristics in different clusters of anoikis. **(A)** Heat map showing the expression profiles of 22 anoikis-associated DEGs between two anoikis clusters. **(B)** Boxplot showing the difference in expression of 22 anoikis -associated DEGs between two anoikis clusters. **(C)** Relative abundance of 22 infiltrating immune cells between two lost apoptosis clusters. **(D)** Boxplot showing the difference in immune infiltration between two anoikis clusters. **(E)** GSVA results of the GO set between two anoikis clusters were plotted in the bar graph. **(F)** GSVA results of the KEGG gene set between two anoikis clusters are plotted in the bar graph. *p < 0.05, **p < 0.01, ***p < 0.001.

We analyzed the differences in 22 DEGs between two different clusters, and the results showed that cluster 1 was characterized by high expression levels of AKT1, SIK1 and TLE1, while cluster 2 showed high expression levels of CEACAM6, STK11, and TFDP1 (Figures 5A,B). To further explore the differences in immune microenvironment characteristics between the different clusters of anoikis, we dissected the differences in infiltrating immune cells and their immune functions. Our results showed that cluster 1 was characterized by a high proportion of naive B cells, whereas cluster 2 exhibited a high proportion of plasma cells, resting memory CD4+ T cells and M2 macrophages (Figures 5C,D). This evidence suggested a different immune profile between the anoikis-associated clusters. Next, we carried out GSVA analysis based on GO and KEGG gene sets. GO results indicated that T cell lineage determination, B cell proliferation, regulation of B cell proliferation, B cell activation and somatic diversity of immunoglobulins in immune response were up-regulated in cluster 2, while chitin binding, cysteine protease binding, axonal dynein complexes, azurophilic granule lumen and Alditol Nadpplus 1 oxidoreductase activities were down-regulated in cluster 2 (Figure 5E). KEGG results showed that JAK-SAT signaling pathway, cytokine-cytokine receptor interaction, natural killer cell-mediated cytotoxicity, antigen processing and presentation, B-cell receptor signaling pathway, T-cell receptor signaling pathway, apoptosis and TOLL-like receptor signaling pathway were up-regulated in cluster 2, while glycerophospholipid metabolism, homologous recombination, systemic lupus erythematosus, metabolism of xenobiotics by cytochrome P450 and drug metabolism cytochrome P450 were down-regulated in cluster 2 (Figure 5F).

### Construction and validation of the lasso model, SVM model, and RF model

**Figure 6:**
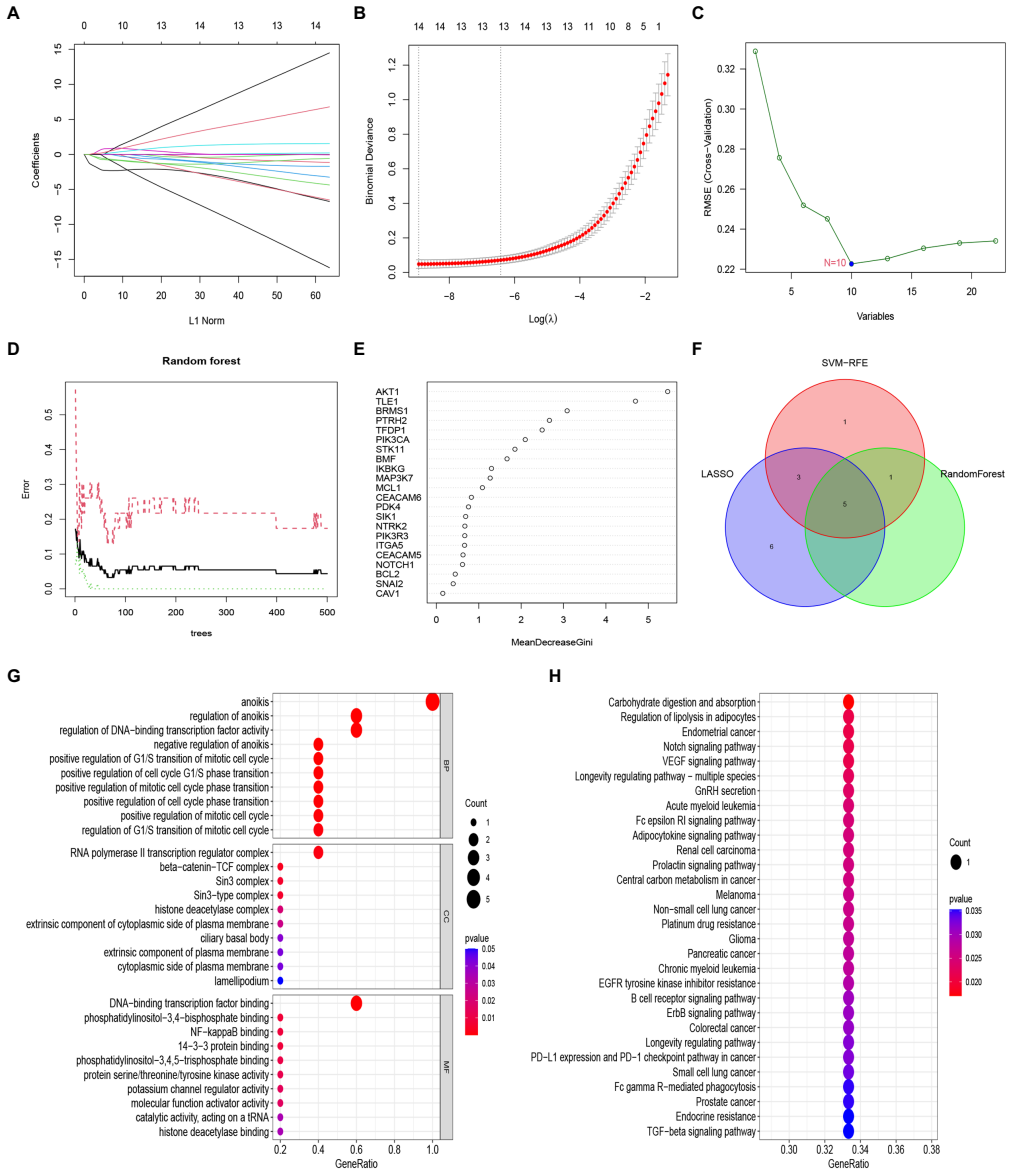
Construction and validation of the Lasso model, SVM model and RF model. **(A)** Fourteen cross-validations of adjusted parameter selection in the LASSO model. Each curve corresponds to one gene. **(B)** LASSO coefficient analysis. Vertical dashed lines are plotted at the best lambda. **(C)** SVM-RFE algorithm for feature gene selection. **(D)** Relationship between the number of random forest trees and error rates. **(E)** Ranking of the relative importance of genes. **(F)** Venn diagram showing the feature genes shared by LASSO, SVM-RFE algorithms, and random forest. **(G)** Bubble plot of GO analysis results based on the 5 feature genes. **(H)** Bubble plot of KEGG analysis results based on 5 signature genes.

We developed three algorithms to select candidate anoikis genes from 22 anoikis-related DEGs to predict the occurrence of IS. The results of the lasso model showed that 14 genes were associated with the occurrence of IS, including AKT1, BCL2, BRMS1, CEACAM5, CEACAM6, ITGA5, MAP3K7, MCL1, NOTCH1, PTRH2, SNAI2, STK11, TFDP1, and TLE1 (Figures 6A,B). Meanwhile, the feature vectors generated by SVM were removed using a support vector machine (SVM) to find the best variables and identify 10 genes for anoikis variables, including AKT1, BRMS1, TLE1, TFDP1, PTRH2, PIK3CA, STK11, BMF, NOTCH1, and MAP3K7 (Figure 6C). For the random forest algorithm, 6 signature genes with relative importance scores greater than two were identified, including AKT1, TLE1, BRMS1, PTRH2, TFDP1, and PIK3CA (Figures 6D,E). Finally, we took the intersection of the genes obtained from the three machine learning models, leaving 5 anoikis genes (AKT1, BRMS1, PTRH2, TFDP1, and TLE1) for follow-up analysis (Figure 6F). And we performed GO and KEGG enrichment analysis on the five diagnostic genes. The GO results depicted that these genes were mainly involved in anoikis, regulation of anoikis, regulation of DNA-binding transcription factor activity, RNA polymerase II transcription regulator complex and DNA-binding transcription factor binding, etc. (Figure 6G). In KEGG, the results indicated that these genes were enriched in Carbohydrate digestion and absorption, Notch signaling pathway, Fc epsilon RI signaling pathway and B cell receptor signaling pathway as well as other pathways (Figure 6H).

**Figure 7:**
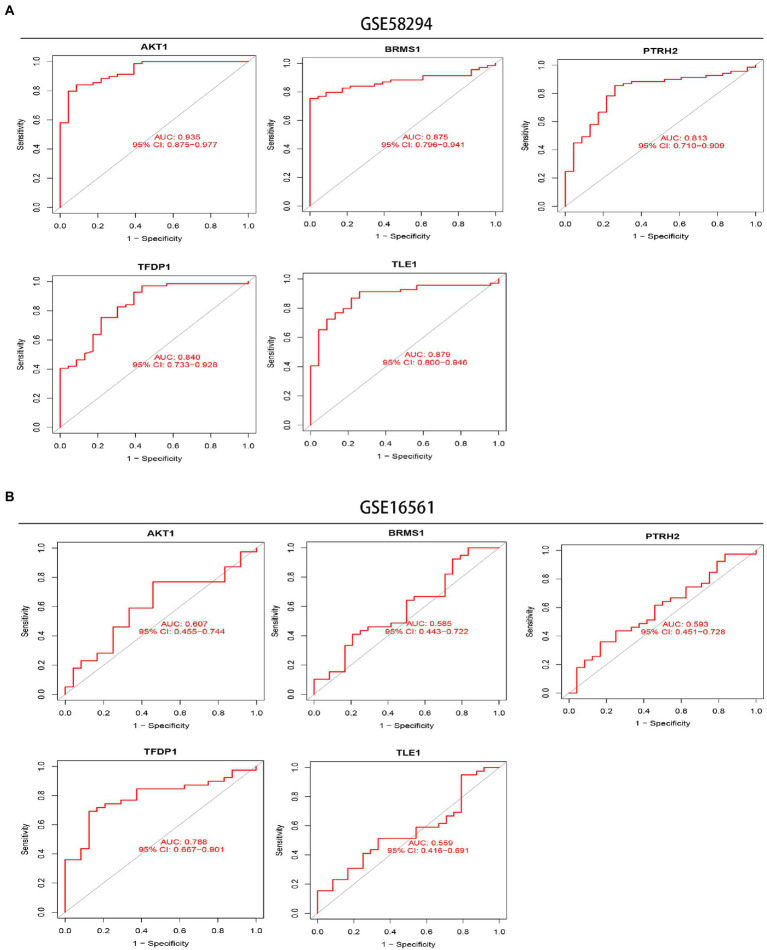
Exploration of the diagnostic value of 5 signature genes. **(A)** ROC curves showing the diagnostic value of the 5 signature genes in the GSE58294 dataset. **(B)** ROC curves showing the diagnostic value of the 5 signature genes validated in the GSE16561 dataset.

Next, we performed ROC analysis and calculated the AUC values of the ROC curves to assess the accuracy of each diagnostic gene. Our results showed that all five genes had relatively high predictive values in the training set (GSE58294; Figure 7A). At the same time, we conducted validation in another dataset (GSE16561; Figure 7B).

### Establishment of nomogram for predicting IS

**Figure 8:**
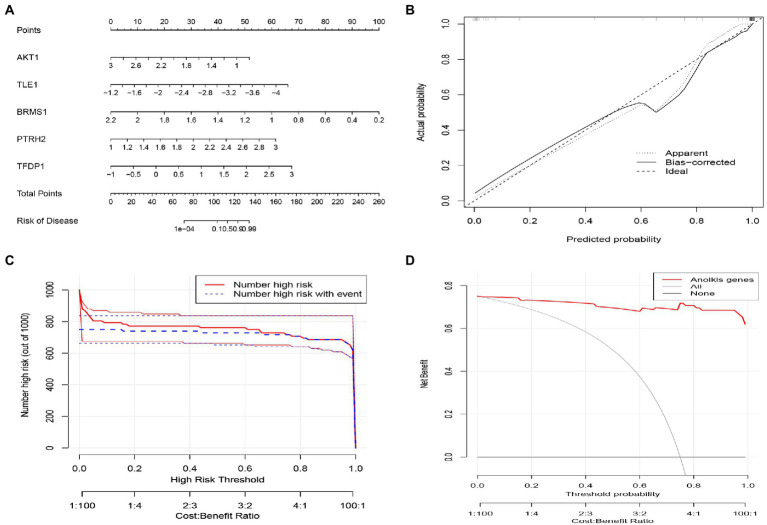
Construction of the nomogram model based on Characteristic ARGs. **(A)** Construction of nomogram integrating Characteristic ARGs for IS. in the nomogram, each variable corresponds to a score, and the total score can be calculated by summing the scores of all variables. **(B)** Calibration curves to estimate the prediction accuracy of the nomogram. **(C)** Clinical impact of the nomogram model as assessed by the clinical impact curve. **(D)** Decision curve analysis showing the clinical benefit of nomogram.

Nomogram were constructed to diagnose IS by integrating trait genes (Figure 8A). In the nomogram, each trait gene corresponds to a score, and the total score is obtained by summing the scores of all trait genes. The total score corresponds to the different risks of IS. The calibration curves showed that nomogram was able to accurately estimate the prediction of IS onset (Figure 8B). The clinical impact curve also showed the significant predictive power of the nomogram model (Figure 8C). As shown in the decision curve analysis, patients with IS can benefit from the nomogram (Figure 8D).

### Immune infiltration correlation analysis of 5 genes

**Figure 9:**
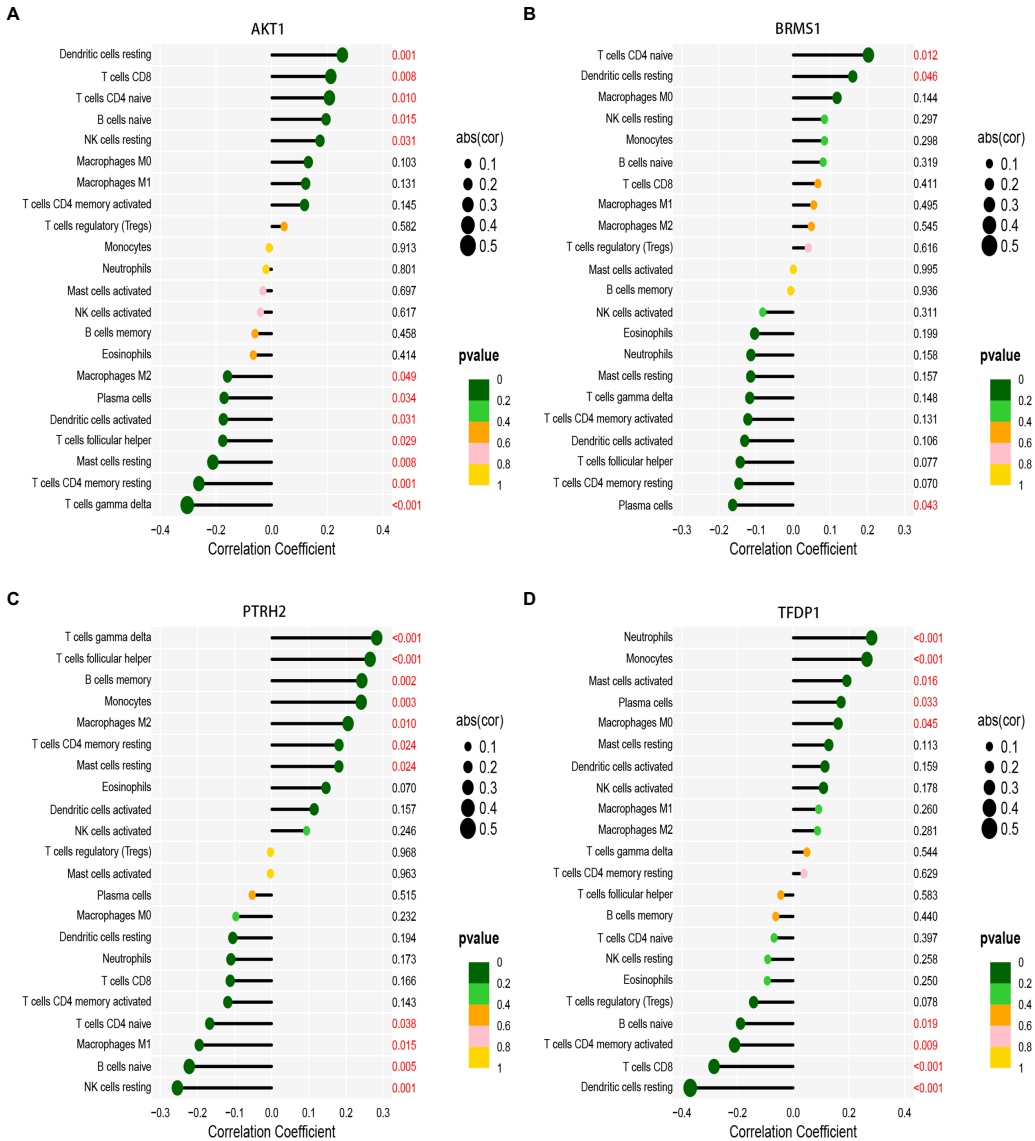
Correlation analysis of immune infiltration with signature gene expression in the combined database of GSE58294 database and GSE16561 database. **(A)** The correlation of AKT1 gene expressions with immune infiltration cell. **(B)** The correlation of BRMS1 gene expressions with immune infiltration cell. **(C)** The correlation of TFDP1 gene expressions with immune infiltration cell. **(D)** The correlation of TLE1 gene expressions with immune infiltration cell. The size of the dots represents the strength of gene correlation with immune cells; the larger the dot, the stronger the correlation. The color of the dots represents the *p*-value; the greener the color, the lower the *p*-value. Numbers marked in red indicate statistical significance. *p* < 0.05 was considered statistically significant.

Then, we analyzed the correlation between gene expression and immune cell infiltration of 5 diagnostic genes in the combined database of GSE58294 database and GSE16561 database. The results showed that AKT1 gene was positively correlated with resting Dendritic cells, CD8+ T cells, naive CD4+ T cells, naive B cells and resting NK cells. And AKT1 gene was negatively correlated with M2 Macrophages, Plasma cells, activated Dendritic cells, follicular helper T cells, resting CD4+ memory T cells, resting Mast cells and gamma delta T cells (Figure 9A). BRMS1 gene was positively correlated with naive CD4+ T cells and resting Dendritic cells, and it was negatively correlated with Plasma cells (Figure 9B). PTRH2 gene was positively correlated with gamma delta T cells, follicular helper T cells, memory B cells, Monocytes, M2 Macrophages, resting memory CD4+ T cells and resting Mast cells. And PTRH2 gene was negatively correlated with naive CD4+ T cells, M1 Macrophages, naive B cells and resting NK cells (Figure 9C). TFDP1 gene was positively correlated with Neutrophils, Monocytes, activated Mast cells, Plasma cells and M0 Macrophages. And TFDP1 gene was negatively correlated with naive B cells, activated CD4+ memory T cells and CD8+ T cells (Figure 9D). But the TLE1 gene showed no statistically significant difference in the combined set. Overall, the expression of these genes may be related to the level of infiltration of a variety of immune cells, implying that these key diagnostic genes are likely to be involved in immune regulation in the pathogenesis of IS.

### Construction of regulatory networks

**Figure 10:**
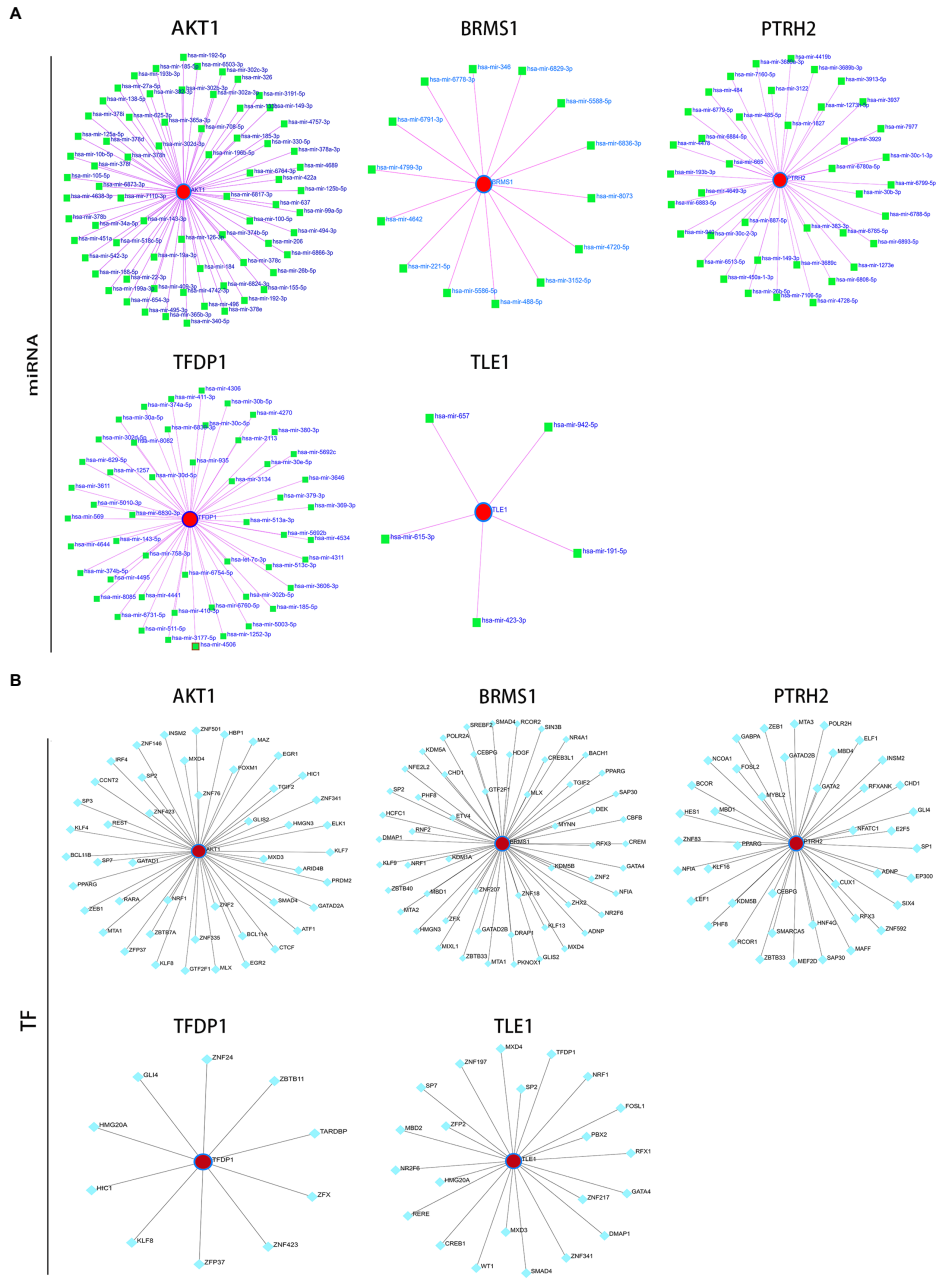
Construction of regulatory network. **(A)** 5 target gene-miRNA regulatory network. **(B)** 5 target gene-TF regulatory network. Red circle nodes represent hub genes, green squares represent miRNAs, and green-blue squares represent TFs.

**Figure 11:**
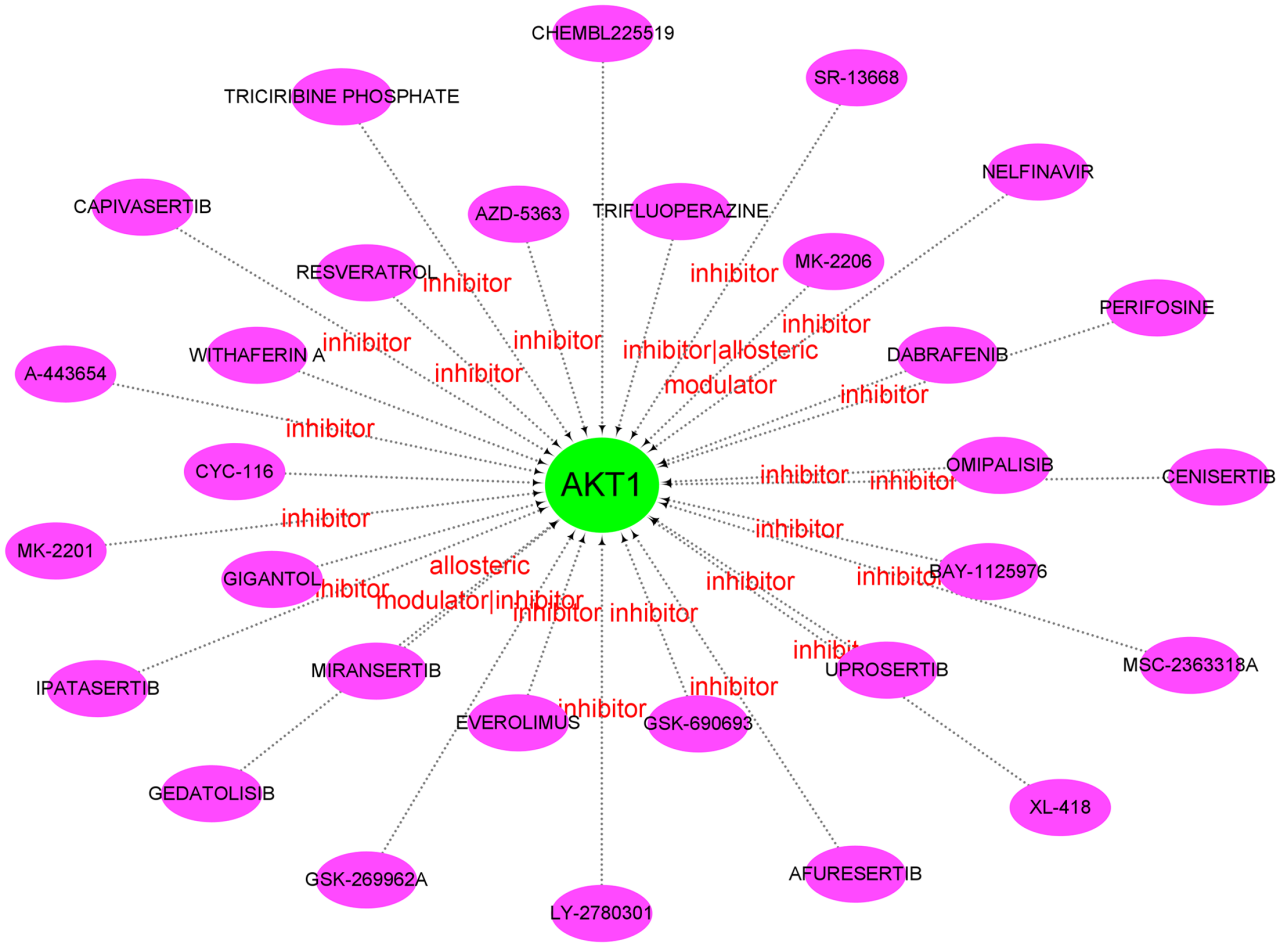
Drug-gene interaction network. Green circle nodes are pivotal genes and purple oval nodes are predicted drugs or molecular compounds. The red words represent the main drug-gene effects.

Afterwards, we constructed the gene-miRNA (Figure 10A) and gene-TF regulatory networks (Figure 10B) of five genes, respectively. The results showed that there were a host of miRNAs and TFs involved in the regulation of these diagnostic genes. In addition, we constructed the drug-gene interaction regulatory network of AKT1. The results indicated that 30 drugs or molecular compounds acted on AKT1, 22 of which inhibited it (Figure 11). In general, these results may provide directions for the future application of these genes in disease diagnosis, disease subtype classification, survival prediction, drug sensitivity analysis and so on.

### Expression of ARGs in different ages or sexes

Finally, we investigated the expression pattern of ARGs in IS patients across age or gender. This may provide a reference for whether patients with IS need personalized treatment. The results showed that there was almost no difference in ARGs expression between IS patients older than 60 years old and IS patients less than or equal to 60 years old (Supplementary Figures 3A,B). Through principal component analysis (PCA), it was clear that IS patients over 60 years of age did not differ well from IS patients less than or equal to 60 years old (Supplementary Figure 3C). Similarly, the expression of ARGs in male IS patients was not significantly different from female IS patients (Supplementary Figures 3D–F). This suggests that individualized treatment strategies may not be necessary for IS treatment in patients of different ages or genders.

## Discussion

IS is a major challenge for clinical neurosurgery due to its very high disability and mortality, which impose a heavy burden on individuals, families and society. Researchers have been exploring new diagnostic methods and therapeutic strategies for a long time to improve early preclinical diagnosis and treatment of IS. Apoptosis is the most common form of programmed cell death in multicellular organisms and can be triggered by intrinsic or extrinsic pathways (Tuo et al., 2022). Anoikis is essentially an apoptotic process. Consistent with classical apoptosis, anoikis can follow either an intrinsic pathway mediated by mitochondria or an extrinsic pathway triggered by cell surface death receptors (Taddei et al., 2012). However, the specific mechanisms by which anoikis regulates disease still need to be further explored. In this study, we used three machine learning algorithms to explore the role of ARGs in IS. For the first time, we comprehensively analyzed the difference of ARGs expression profile between normal samples and IS samples. We found 22 dysfunctional ARGs in IS patients, indicating the potential role of anoikis in the development of IS.

Much of research have shown that IS leads to the up-regulation of activated memory CD4+ T cells, follicular helper T cells, monocytes, M0 macrophages, resting dendritic cells, and neutrophils, as well as the down-regulation of naive B cells, CD8+ T cells, naive CD4+ T cells, and resting mast cells (DeLong et al., 2022; Endres et al., 2022; Zheng et al., 2022), which is consistent with our results. Different kinds of immune cells play key roles in IS. Neutrophils can increase stroke severity through a variety of mechanisms such as triggering capillary sludge, producing free radicals, secreting inflammatory mediators, enhancing thrombosis by forming neutrophil–platelet aggregates and NETs and up-regulating nuclear PKM2 (Aronowski and Roy-O’Reilly, 2019; Denorme et al., 2022; Dhanesha et al., 2022). Similarly, mast cells can exacerbate CNS injury in IS by amplifying the inflammatory response and promoting cerebral blood barrier disruption, brain edema, extravasation, and hemorrhage (Parrella et al., 2019). In contrast, Monocytes mainly secrete TNF-α, IL-6 and IL-1β to exert pro-inflammatory effects, as well as enhance CB2 receptor expression and disrupt BBB to exacerbate IS, but inhibition of their recruitment can significantly prevent brain edema (Boyette et al., 2017; Greco et al., 2021; Qiu et al., 2021). In addition, it was reported that an increase in the number of Treg cells and depletion of CD4+ T cells can improve the long-term outcome after stroke (Shi et al., 2021; Weitbrecht et al., 2021). And M2 macrophages enhance neurogenesis and angiogenesis by secreting various neurotrophic factors such as IGF-1, BDNF and VEGF, and promote the recovery of neurological function after cerebral ischemia/reperfusion injury in rats (Li et al., 2021). And our clustering analysis exhibited that we could classify IS patients into two clusters and that cluster 2 showed elevated proportions of plasma cells, resting memory CD4+ T cells and M2 macrophages. Thus, cluster 2 patients may have a better prognosis and targeting different immune cells after IS may also be an important direction for further exploration of IS treatment strategies in the future.

In recent years, machine learning has been increasingly used to diagnose IS, screen key genes and immune cells due to its better performance of prediction, lower error rate and higher reliability (Brugnara et al., 2020; Wang J. et al., 2022). In our research, we screened five signature genes, AKT1, BRMS1, PTRH2, TFDP1, and TLE1, by LASSO, SVM-RFE and RF algorithms. And these five feature genes have good diagnostic value (all AUC > 0.8) in training set (IS sample size *n* = 69). However, the results of their diagnostic value in the verification set were not very satisfactory. But the AUC values of the five genes were all greater than 0.55, and the AUC value of TFDP1 was 0.788. This may be due to the small sample size of IS patients in the verification group (IS sample size *n* = 39). And in addition, a nomogram containing five genes can combine five signature genes to better diagnose the occurrence of IS.

At the same time, a multitude of studies have shown that some of these key diagnostic genes were involved in the pathogenesis of IS. AKT1 not only promotes neuronal survival after IS and attenuates hippocampal neuronal injury through various signaling pathways such as AKT-nNOS-JNK (Xie et al., 2013; Shao et al., 2017), but also restores BBB senescence by activating the eNOS-SIRT1 axis, thereby preventing IS in aged mice (Li et al., 2022). According to recent reports, PTRH2 mutations have been shown to be one of the molecular mechanisms leading to ataxia and cerebellar atrophy in patients with multisystem neurological, endocrine and pancreatic diseases in infancy (Picker-Minh et al., 2022). The TFDP1 gene controls both the transcriptional activity of cellular genes and was also involved in the cyclic regulation of cellular genes, affecting cell proliferation and apoptosis (Hitchens and Robbins, 2003; Liu et al., 2022). Previous studies have found that the E2F-related transcription factor TFDP1 may be involved in the induction of genes related to functional modules in the transcriptome of ischemic neurons (Jin et al., 2001). BRMS1 plays a central role mainly in the inhibition of cancer metastasis. BRMS1 was able to influence both the association of cells with the immune environment and to regulate the expression of high-impact molecules such as FAK, EGFR, AKT and NF-κB (Zimmermann and Welch, 2020). And TLE1 plays a role in participating in the immune inflammatory response, suppressing apoptosis of lost nests, and suppressing tumor activity but also acting as an oncogene in some tumors (Yu et al., 2022). However, the potential mechanism of action of BRMS1 and TLE1 in IS has not been reported. In conclusion, the researches involving these characteristic genes showed that the results of our screening are reliable to some extent.

Meanwhile, gene enrichment analysis showed that these key genes were mainly involved in anoikis, regulation of anoikis, Notch signaling pathway, Fc epsilon RI signaling pathway, B cell receptor signaling pathway and inflammatory or immune-related signal pathways, etc. However, the regulatory relationship between these key genes, and the mechanism of various signal pathways and IS still need further experimental validation.

In addition, we further analyzed these characteristic genes, including exploring the correlation of their immune infiltration and their interaction networks with miRNA, TF and drug regulation, which can provide directions for our subsequent IS targeting and immunotherapy. In the future, we will continue to explore their potential mechanism in IS through molecular biology experiments.

Finally, we discussed the differences in the expression of ARGs by age or sex. The results showed that there was no difference in the expression of ARGs among different genders or ages. This implies that individualized diagnostic and therapeutic strategies may not be required for the treatment of IS in patients of different ages or genders. However, previous studies have shown that age and gender are key factors in the pathology of IS. The mortality and morbidity of elderly patients with stroke were higher, and their functional recovery was worse than that of younger patients. Men have a higher risk of developing IS when they are young, while stroke is more common in women (Roy-O’Reilly and McCullough, 2018). And the mortality and disability rate of women after stroke is higher than that of men (Gasbarrino et al., 2022). Thus, more research is needed to unravel the link between age and sex and stroke immune response.

It should not be overlooked that this study has several limitations. The first and foremost, our current study was conducted based on a public dataset with profiles from blood samples rather than brain tissue, and the conclusions were drawn by bioinformatics methods, which should thereafter be validated for reliability. There is one more point, it is not clear whether the above genes are expressed at different levels between individuals of different regions or races. The last but not the least, more *in vivo* and *in vitro* studies are needed to elucidate the potential mechanisms underlying these correlations between AKT1, BRMS1, PTRH2, TFDP1, and TLE1 and infiltrating immune cells in IS.

## Conclusion

In brief, our study comprehensively analyzed the role of ARGs in IS for the first time. We revealed the expression profile of ARGs in IS and the correlation with infiltrating immune cells, and demonstrated consensus clustering analysis and machine learning analysis based on ARGs to analyze five signature genes, AKT1, BRMS1, MAP3K7, NOTCH1, PTRH2, STK11, TFDP1, and TLE1, in the immune infiltration and the role in diagnosis. The results suggested that we can classify IS patients into two clusters. The results also indicated that the expression of ARGs did not differ by sex or age. Our study may provide a beneficial reference to further elucidate the pathogenesis of IS and render new ideas to drug screening, individualized treatment and immunotherapy of IS.

## Data availability statement

The data presented in the study are deposited in the jianguoyun repository, and can be obtained from the following link: https://www.jianguoyun.com/p/DXqdIbcQxK21Cxi4kfkEIAA.

## Author contributions

XQ, ZC, and LW have contributed to research conception and design. XQ, SY, and JR have contributed to result interpretation and manuscript drafting. XQ, JR, and HL were involved in data download, statistical analysis, and visualization. SY, BJ, WZ, and RD, were involved in interpretation of the study results and critical revision for important intellectual contents. All authors contributed to the article and approved the submitted version.

## Funding

This research was supported by the Natural Science Foundation of Hubei Provincial Science and Technology Department, Project 2020CFB598.

## Conflict of interest

The authors declare that the research was conducted in the absence of any commercial or financial relationships that could be construed as a potential conflict of interest.

## Publisher’s note

All claims expressed in this article are solely those of the authors and do not necessarily represent those of their affiliated organizations, or those of the publisher, the editors and the reviewers. Any product that may be evaluated in this article, or claim that may be made by its manufacturer, is not guaranteed or endorsed by the publisher.

## Footnotes

^1^ https://www.ncbi.nlm.nih.gov/geo/

^2^ https://www.gsea-msigdb.org/gsea/msigdb/

^3^ http://www.networkanalyst.ca

^4^ https://dgidb.genome.wustl.edu/

## References

[ref1] Adeshakin F. O. Adeshakin A. O. Afolabi L. O. Yan D. Zhang G. Wan X. (2021). Mechanisms for modulating Anoikis resistance in cancer and the relevance of metabolic reprogramming. Front. Oncol. 11:626577. doi: 10.3389/fonc.2021.626577 33854965 PMC8039382

[ref2] Ajoolabady A. Wang S. Kroemer G. Penninger J. M. Uversky V. N. Pratico D.. (2021). Targeting autophagy in ischemic stroke: from molecular mechanisms to clinical therapeutics. Pharmacol. Ther. 225:107848. doi: 10.1016/j.pharmthera.2021.107848 33823204 PMC8263472

[ref3] Aronowski J. Roy-O’Reilly M. A. (2019). Neutrophils, the felons of the brain. Stroke 50:e42-e43. doi: 10.1161/STROKEAHA.118.021563 30674235 PMC6544162

[ref4] Boyette L. B. Macedo C. Hadi K. Elinoff B. D. Walters J. T. Ramaswami B.. (2017). Phenotype, function, and differentiation potential of human monocyte subsets. PLoS One 12:e0176460. doi: 10.1371/journal.pone.0176460 28445506 PMC5406034

[ref5] Brugnara G. Neuberger U. Mahmutoglu M. A. Foltyn M. Herweh C. Nagel S.. (2020). Multimodal predictive modeling of endovascular treatment outcome for acute ischemic stroke using machine-learning. Stroke 51, 3541–3551. doi: 10.1161/STROKEAHA.120.030287 33040701

[ref6] Cai J. Ye Z. Hu Y. Yang J. Wu L. Yuan F.. (2022). Identification of immunogenic cell death-related gene classification patterns and immune infiltration characterization in ischemic stroke based on machine learning. Front. Cell. Neurosci. 16:1094500. doi: 10.3389/fncel.2022.1094500 36601430 PMC9806121

[ref7] Ceulemans A. G. Zgavc T. Kooijman R. Hachimi-Idrissi S. Sarre S. Michotte Y. (2010). The dual role of the neuroinflammatory response after ischemic stroke: modulatory effects of hypothermia. J. Neuroinflammation 7:74. doi: 10.1186/1742-2094-7-74 21040547 PMC2988764

[ref8] Chi H. Jiang P. Xu K. Zhao Y. Song B. Peng G.. (2022a). A novel anoikis-related gene signature predicts prognosis in patients with head and neck squamous cell carcinoma and reveals immune infiltration. Front. Genet. 13:984273. doi: 10.3389/fgene.2022.984273 36092898 PMC9459093

[ref9] Chi H. Peng G. Wang R. Yang F. Xie X. Zhang J.. (2022c). Cuprotosis programmed-cell-death-related lncRNA signature predicts prognosis and immune landscape in PAAD patients. Cells 11:3436. doi: 10.3390/cells11213436 36359832 PMC9658590

[ref10] Chi H. Peng G. Yang J. Zhang J. Song G. Xie X.. (2022d). Machine learning to construct sphingolipid metabolism genes signature to characterize the immune landscape and prognosis of patients with uveal melanoma. Front. Endocrinol. 13:1056310. doi: 10.3389/fendo.2022.1056310 PMC9772281 36568076

[ref11] Chi H. Xie X. Yan Y. Peng G. Strohmer D. F. Lai G.. (2022b). Natural killer cell-related prognosis signature characterizes immune landscape and predicts prognosis of HNSCC. Front. Immunol. 13:1018685. doi: 10.3389/fimmu.2022.1018685 36263048 PMC9575041

[ref12] DeLong J. H. Ohashi S. N. O’Connor K. C. Sansing L. H. (2022). Inflammatory responses after ischemic stroke. Springer Semin. Immunopathol. 44, 625–648. doi: 10.1007/s00281-022-00943-7 35767089

[ref13] Denorme F. Portier I. Rustad J. L. Cody M. J. de Araujo C. V. Hoki C.. (2022). Neutrophil extracellular traps regulate ischemic stroke brain injury. J. Clin. Invest. 132:e154225. doi: 10.1172/JCI154225 35358095 PMC9106355

[ref14] Dhanesha N. Patel R. B. Doddapattar P. Ghatge M. Flora G. D. Jain M.. (2022). PKM2 promotes neutrophil activation and cerebral thromboinflammation: therapeutic implications for ischemic stroke. Blood 139, 1234–1245. doi: 10.1182/blood.2021012322 34529778 PMC8874361

[ref15] Diao X. Guo C. Li S. (2022). Identification of a novel anoikis-related gene signature to predict prognosis and tumor microenvironment in lung adenocarcinoma. Thorac Cancer 14, 320–330. doi: 10.1111/1759-7714.14766 36507553 PMC9870742

[ref16] Endres M. Moro M. A. Nolte C. H. Dames C. Buckwalter M. S. Meisel A. (2022). Immune pathways in etiology, acute phase, and chronic Sequelae of ischemic stroke. Circ. Res. 130:361, 1167–1186. doi: 10.1161/CIRCRESAHA.121.319994 35420915

[ref17] Fukuta T. Asai T. Yanagida Y. Namba M. Koide H. Shimizu K.. (2017). Combination therapy with liposomal neuroprotectants and tissue plasminogen activator for treatment of ischemic stroke. Fed. Proc. 31, 1879–1890. doi: 10.1096/fj.201601209R 28082354

[ref18] Gasbarrino K. Di Iorio D. Daskalopoulou S. S. (2022). Importance of sex and gender in ischaemic stroke and carotid atherosclerotic disease. Eur Heart J Cardiovasc Pharmacother 43, 460–473. doi: 10.1093/eurheartj/ehab756 PMC8830529 34849703

[ref19] Greco R. Demartini C. Zanaboni A. Tumelero E. Elisa C. Persico A.. (2021). Characterization of CB2 receptor expression in peripheral blood monocytes of acute ischemic stroke patients. Transl. Stroke Res. 12, 550–558. doi: 10.1007/s12975-020-00851-8 32960432

[ref20] Hitchens M. R. Robbins P. D. (2003). The role of the transcription factor DP in apoptosis. Apoptosis 8, 461–468. doi: 10.1023/A:1025586207239 12975577

[ref21] Iadecola C. Anrather J. (2011). The immunology of stroke: from mechanisms to translation. Nat. Med. 17, 796–808. doi: 10.1038/nm.2399 21738161 PMC3137275

[ref22] Iadecola C. Buckwalter M. S. Anrather J. (2020). Immune responses to stroke: mechanisms, modulation, and therapeutic potential. J. Clin. Invest. 130, 2777–2788. doi: 10.1172/JCI135530 32391806 PMC7260029

[ref23] Jin K. Mao X. O. Eshoo M. W. Nagayama T. Minami M. Simon R. P.. (2001). Microarray analysis of hippocampal gene expression in global cerebral ischemia. Ann. Neurol. 50, 93–103. doi: 10.1002/ana.1073 11456315

[ref24] Lakhan S. E. Kirchgessner A. Hofer M. (2009). Inflammatory mechanisms in ischemic stroke: therapeutic approaches. J. Transl. Med. 7:97. doi: 10.1186/1479-5876-7-97 19919699 PMC2780998

[ref25] Li L. Gan H. Jin H. Fang Y. Yang Y. Zhang J.. (2021). Astragaloside IV promotes microglia/macrophages M2 polarization and enhances neurogenesis and angiogenesis through PPARgamma pathway after cerebral ischemia/reperfusion injury in rats. Int. Immunopharmacol. 92:107335. doi: 10.1016/j.intimp.2020.107335 33429332

[ref26] Li Q. Niu X. Yi Y. Chen Y. Yuan J. Zhang J.. (2022). Inducible pluripotent stem cell-derived small extracellular vesicles rejuvenate senescent blood-brain barrier to protect against ischemic stroke in aged mice. ACS Nano 17, 775–789. doi: 10.1021/acsnano.2c10824. 36562422

[ref27] Lindsay M. P. Norrving B. Sacco R. L. Brainin M. Hacke W. Martins S.. (2019). World stroke organization (WSO): global stroke fact sheet 2019. Int. J. Stroke 14, 806–817. doi: 10.1177/1747493019881353, PMID: 31658892

[ref28] Liu Y. Guo S. He X. Jiang Y. Hong Q. Lan R.. (2022). Effect of Upregulation of transcription factor TFDP1 binding promoter activity due to RBP4 g.36491960G>C mutation on the proliferation of goat Granulosa cells. Cells 11:2148. doi: 10.3390/cells11142148 35883591 PMC9321149

[ref29] Parrella E. Porrini V. Benarese M. Pizzi M. (2019). The role of mast cells in stroke. Cells 8:437. doi: 10.3390/cells8050437 31083342 PMC6562540

[ref30] Picker-Minh S. Luperi I. Ravindran E. Kraemer N. Zaqout S. Stoltenburg-Didinger G.. (2022). PTRH2 is necessary for Purkinje cell differentiation and survival and its loss recapitulates progressive cerebellar atrophy and ataxia seen in IMNEPD patients. Cerebellum. doi: 10.1007/s12311-022-01488-z, PMID: 36219306 PMC10657312

[ref31] Qiu Y. M. Zhang C. L. Chen A. Q. Wang H. L. Zhou Y. F. Li Y. N.. (2021). Immune cells in the BBB disruption after acute ischemic stroke: targets for immune therapy? Front. Immunol. 12:678744. doi: 10.3389/fimmu.2021.678744 34248961 PMC8260997

[ref32] Roy-O’Reilly M. McCullough L. D. (2018). Age and sex are critical factors in ischemic stroke pathology. Endocrinology 159, 3120–3131. doi: 10.1210/en.2018-00465 30010821 PMC6963709

[ref33] Saini V. Guada L. Yavagal D. R. (2021). Global epidemiology of stroke and access to acute ischemic stroke interventions. Neurol. Genet. 97, S6–S16. doi: 10.1212/WNL.0000000000012781 34785599

[ref34] Shao S. Xu M. Zhou J. Ge X. Chen G. Guo L.. (2017). Atorvastatin attenuates ischemia/reperfusion-induced hippocampal neurons injury via Akt-nNOS-JNK signaling pathway. Cell. Mol. Neurobiol. 37, 753–762. doi: 10.1007/s10571-016-0412-x 27488855 PMC11482104

[ref35] Shi L. Sun Z. Su W. Xu F. Xie D. Zhang Q.. (2021). Treg cell-derived osteopontin promotes microglia-mediated white matter repair after ischemic stroke. Immunity 54, 1527–1542. doi: 10.1016/j.immuni.2021.04.022 34015256 PMC8282725

[ref36] Sun Z. Zhao Y. Wei Y. Ding X. Tan C. Wang C. (2022). Identification and validation of an anoikis-associated gene signature to predict clinical character, stemness, IDH mutation, and immune filtration in glioblastoma. Front. Immunol. 13:939523. doi: 10.3389/fimmu.2022.939523 36091049 PMC9452727

[ref37] Taddei M. L. Giannoni E. Fiaschi T. Chiarugi P. (2012). Anoikis: an emerging hallmark in health and diseases. J. Pathol. 226, 380–393. doi: 10.1002/path.3000 21953325

[ref38] Tuo Q. Z. Zhang S. T. Lei P. (2022). Mechanisms of neuronal cell death in ischemic stroke and their therapeutic implications. Med. Res. Rev. 42, 259–305. doi: 10.1002/med.21817 33957000

[ref39] Wang J. Kang Z. Liu Y. Li Z. Liu Y. Liu J. (2022). Identification of immune cell infiltration and diagnostic biomarkers in unstable atherosclerotic plaques by integrated bioinformatics analysis and machine learning. Front. Immunol. 13:956078. doi: 10.3389/fimmu.2022.956078 36211422 PMC9537477

[ref40] Wang X. Zhao Y. Strohmer D. F. Yang W. Xia Z. Yu C. (2022). The prognostic value of MicroRNAs associated with fatty acid metabolism in head and neck squamous cell carcinoma. Front. Genet. 13:983672. doi: 10.3389/fgene.2022.983672 36110217 PMC9468645

[ref41] Weitbrecht L. Berchtold D. Zhang T. Jagdmann S. Dames C. Winek K.. (2021). CD4(+) T cells promote delayed B cell responses in the ischemic brain after experimental stroke. Brain Behav Immun Health 91, 601–614. doi: 10.1016/j.bbi.2020.09.029, PMID: 33002634

[ref42] Xie R. Cheng M. Li M. Xiong X. Daadi M. Sapolsky R. M.. (2013). Akt isoforms differentially protect against stroke-induced neuronal injury by regulating mTOR activities. Cerebrovasc. Brain Metab. Rev. 33, 1875–1885. doi: 10.1038/jcbfm.2013.132 PMC3851893 23942361

[ref43] Yang X. Zhang C. Yan C. Ma L. Ma J. Meng X. (2022). System analysis based on the ER stress-related genes identifies WFS1 as a novel therapy target for colon cancer. Aging 14, 9243–9263. doi: 10.18632/aging.204404 36445321 PMC9740360

[ref44] Yu G. Chen Y. Hu Y. Zhou Y. Ding X. Zhou X. (2022). Roles of transducin-like enhancer of split (TLE) family proteins in tumorigenesis and immune regulation. Front. Cell Dev. Biol. 10:1010639. doi: 10.3389/fcell.2022.1010639 36438567 PMC9692235

[ref45] Zhao S. Chi H. Ji W. He Q. Lai G. Peng G.. (2022a). A bioinformatics-based analysis of an Anoikis-related gene signature predicts the prognosis of patients with low-grade Gliomas. Brain Sci. 12:1349. doi: 10.3390/brainsci12101349 36291283 PMC9599312

[ref46] Zhao S. Chi H. Yang Q. Chen S. Wu C. Lai G.. (2023). Identification and validation of neurotrophic factor-related gene signatures in glioblastoma and Parkinson’s disease. Front. Immunol. 14:1090040. doi: 10.3389/fimmu.2023.1090040 36825022 PMC9941742

[ref47] Zhao S. Zhang L. Ji W. Shi Y. Lai G. Chi H.. (2022b). Machine learning-based characterization of cuprotosis-related biomarkers and immune infiltration in Parkinson’s disease. Front. Genet. 13:1010361. doi: 10.3389/fgene.2022.1010361 36338988 PMC9629507

[ref48] Zheng P. F. Chen L. Z. Liu P. Pan H. W. Fan W. J. Liu Z. Y. (2022). Identification of immune-related key genes in the peripheral blood of ischaemic stroke patients using a weighted gene coexpression network analysis and machine learning. J. Transl. Med. 20:361. doi: 10.1186/s12967-022-03562-w 35962388 PMC9373395

[ref49] Zhu Z. Fang C. Xu H. Yuan L. Du Y. Ni Y.. (2022). Anoikis resistance in diffuse glioma: the potential therapeutic targets in the future. Front. Oncol. 12:976557. doi: 10.3389/fonc.2022.976557 36046036 PMC9423707

[ref50] Zimmermann R. C. Welch D. R. (2020). BRMS1: a multifunctional signaling molecule in metastasis. Cancer Metastasis Rev. 39, 755–768. doi: 10.1007/s10555-020-09871-0 32232621 PMC7487056

