# Supplementary material for: Identification of anoikis-related genes classification patterns and immune infiltration characterization in ischemic stroke based on machine learning
Source: Front Aging Neurosci. 2023 Mar 23;15:1142163. doi: 10.3389/fnagi.2023.1142163 (PMC10076550; doi:10.3389/fnagi.2023.1142163)
Supplement: Supplementary file 1 [file Data_Sheet_1.ZIP › Supplementary material/Supplementary material-Figure Legends.pdf]

Supplementary Figures 1. Consensus clustering K=2-9 and corresponding cumulative distribution function (CDF) curves in dataset 58294.

Supplementary Figures 2. Consensus clustering K=2-9 and corresponding cumulative distribution function (CDF) curves in dataset 16561.

Supplementary Figures 3. Expression profiles of ARGs across age and sex. **(A)** Heat map showing the expression of ARGs across different ages (>60 years or ≤60 years). **(B)** Box plot showing the difference in ARGs expression across different ages (>60 or ≤60 years). **(C)** Principal component analysis (PCA) of ARGs expression by age (>60 years or ≤60 years). **(D)** Heat map showing the expression of ARGs by gender. **(E)** Box plot showing gender differences in ARGs expression. **(F)** Principal component analysis (PCA) of ARGs expression by gender.
