# Supplementary figures and images for: Identification of anoikis-related genes classification patterns and immune infiltration characterization in ischemic stroke based on machine learning (part 1 of 2)
Source: Front Aging Neurosci. 2023 Mar 23;15:1142163. doi: 10.3389/fnagi.2023.1142163 (PMC10076550; doi:10.3389/fnagi.2023.1142163)

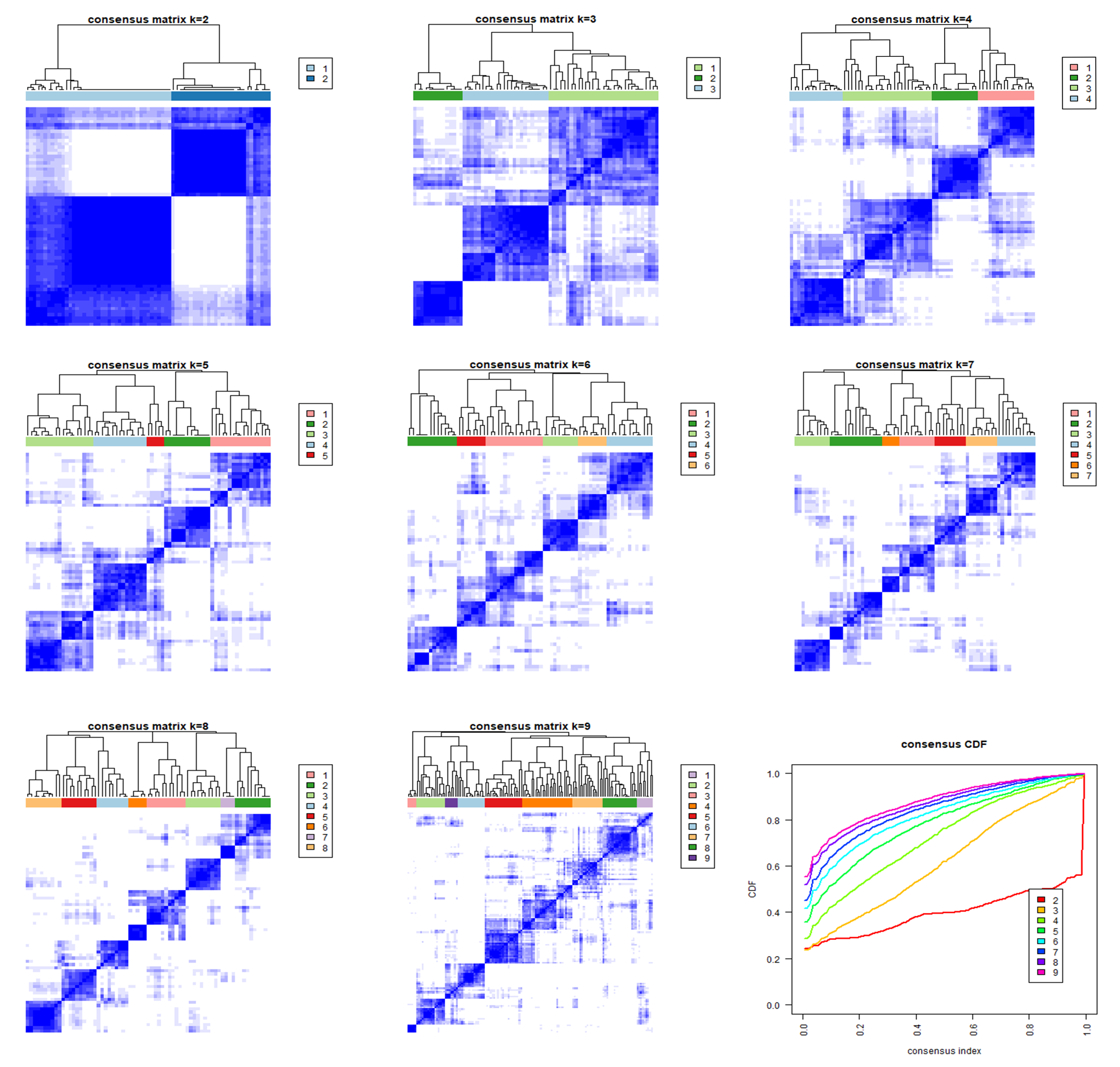

Supplement: Supplementary file 1 [file Data_Sheet_1.ZIP › Supplementary material/Supplementary Figures 1.tif]

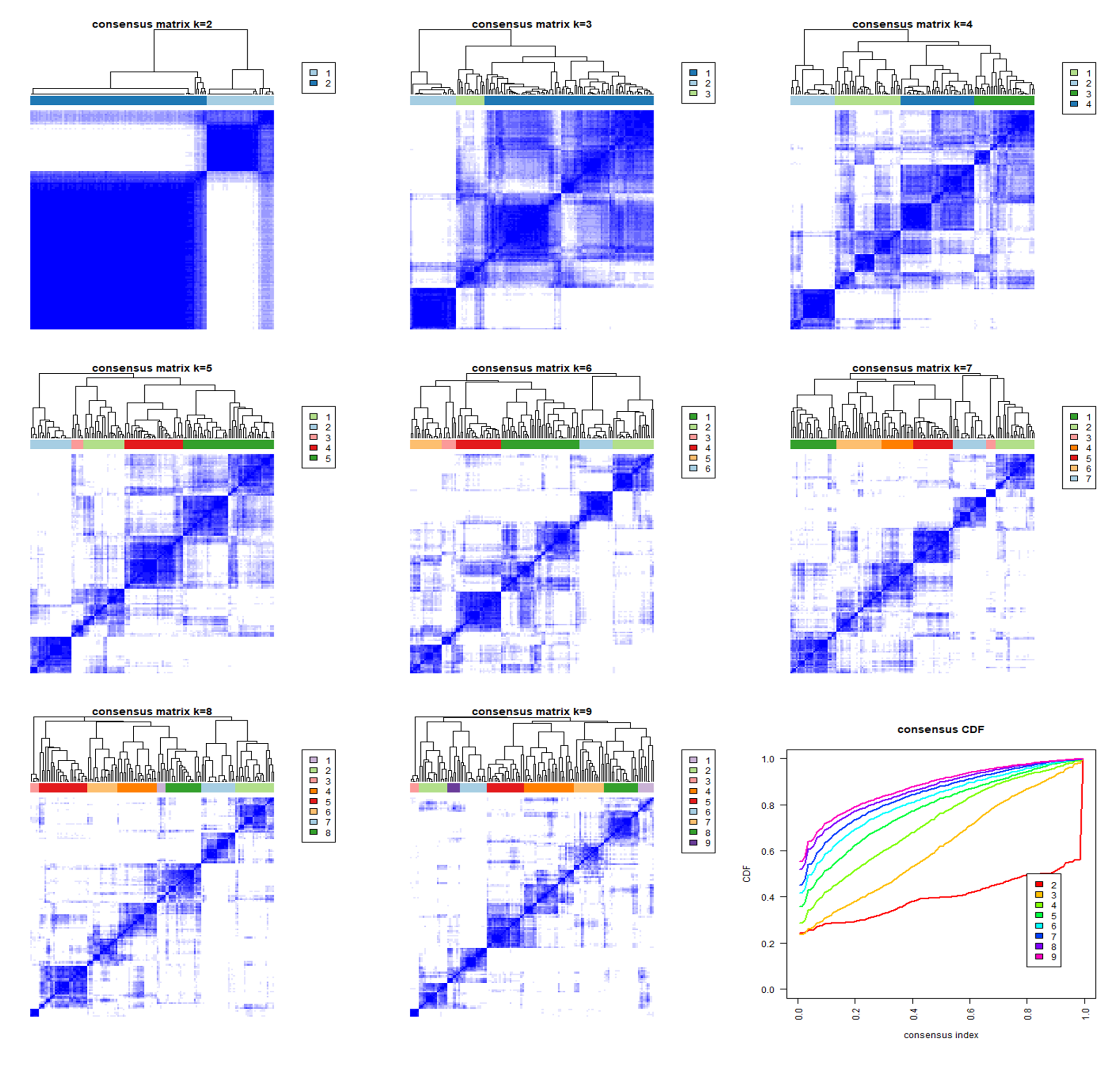

Supplement: Supplementary file 1 [file Data_Sheet_1.ZIP › Supplementary material/Supplementary Figures 2.tif]

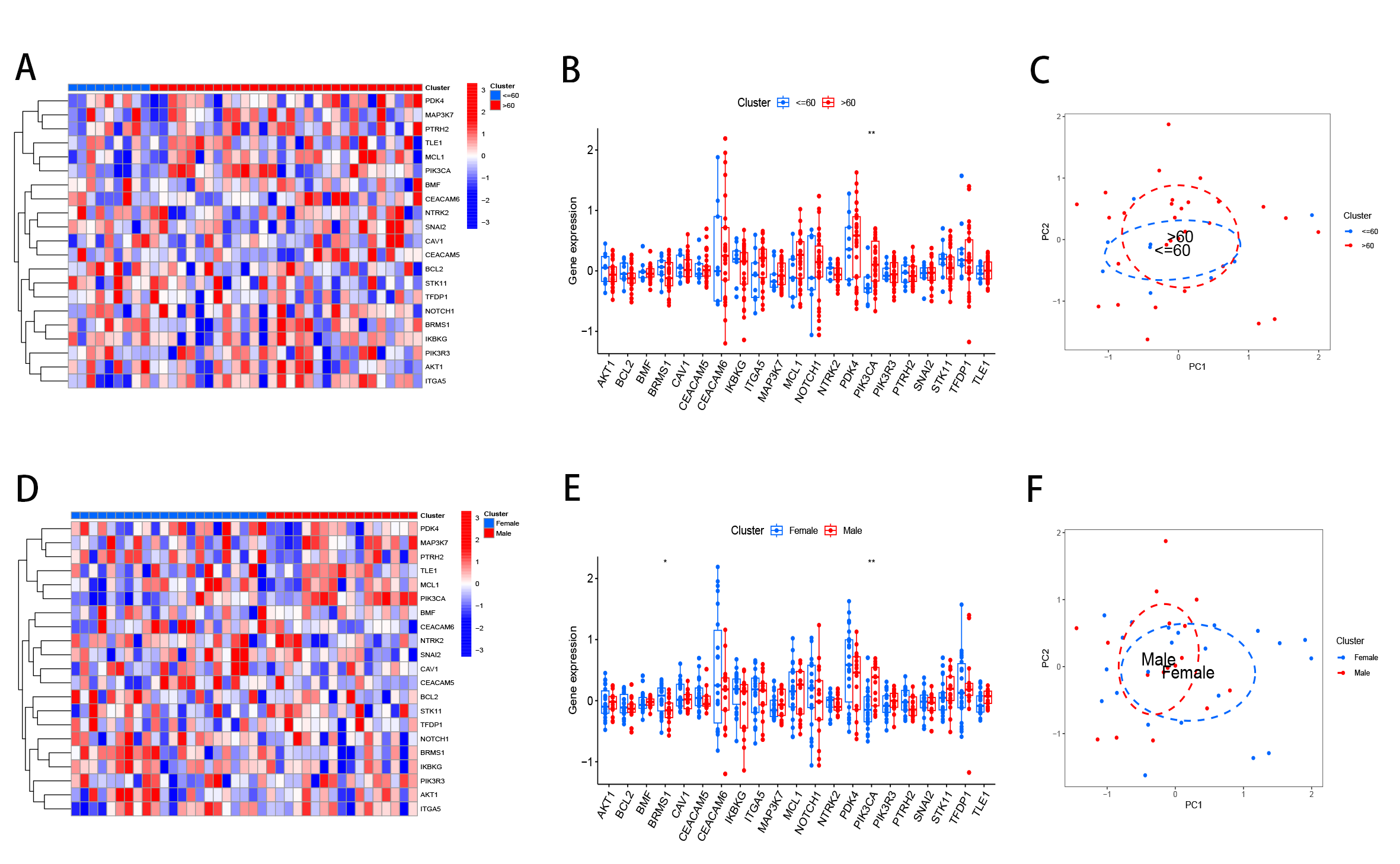

Supplement: Supplementary file 1 [file Data_Sheet_1.ZIP › Supplementary material/Supplementary Figures 3.tif]

Cluster 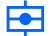 C1 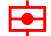 C2

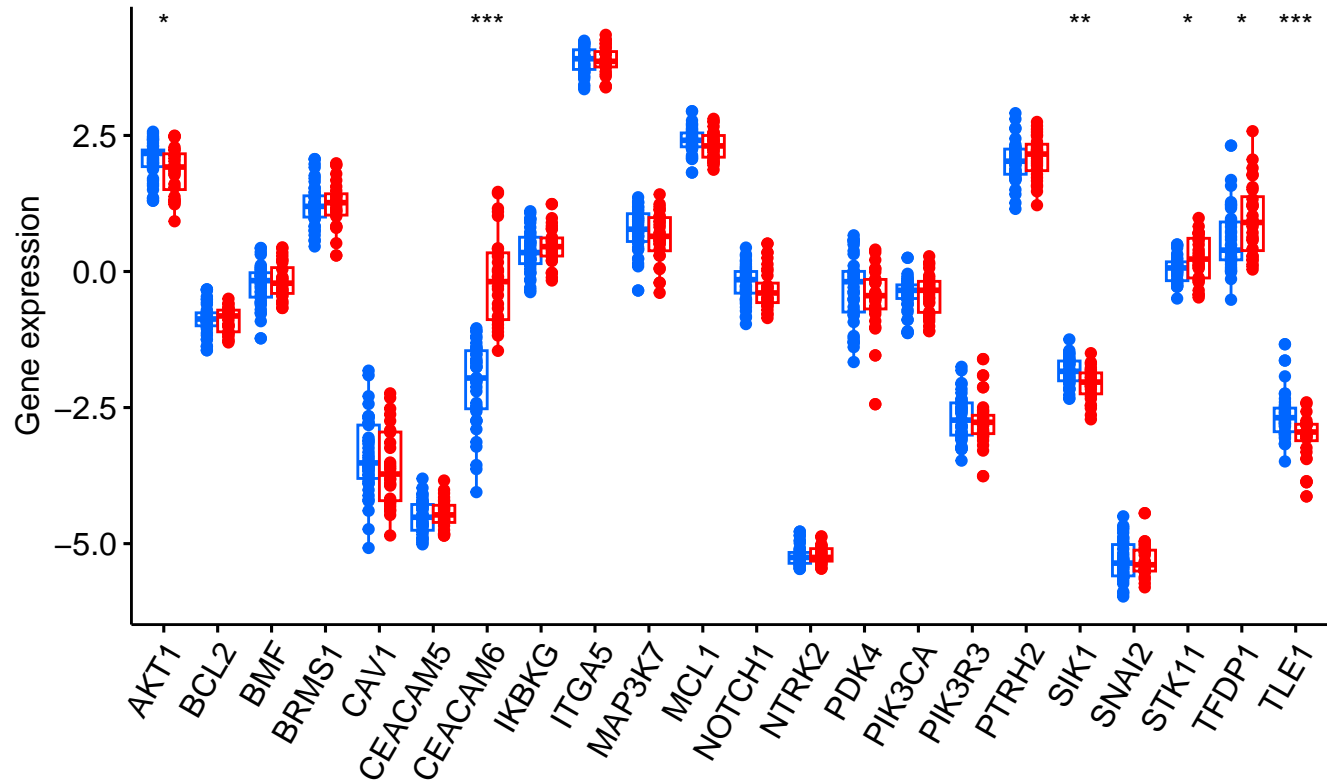

Supplement: Supplementary file 2 [file Data_Sheet_2.ZIP › Fig 5/═╝5/15.boxplot.pdf]

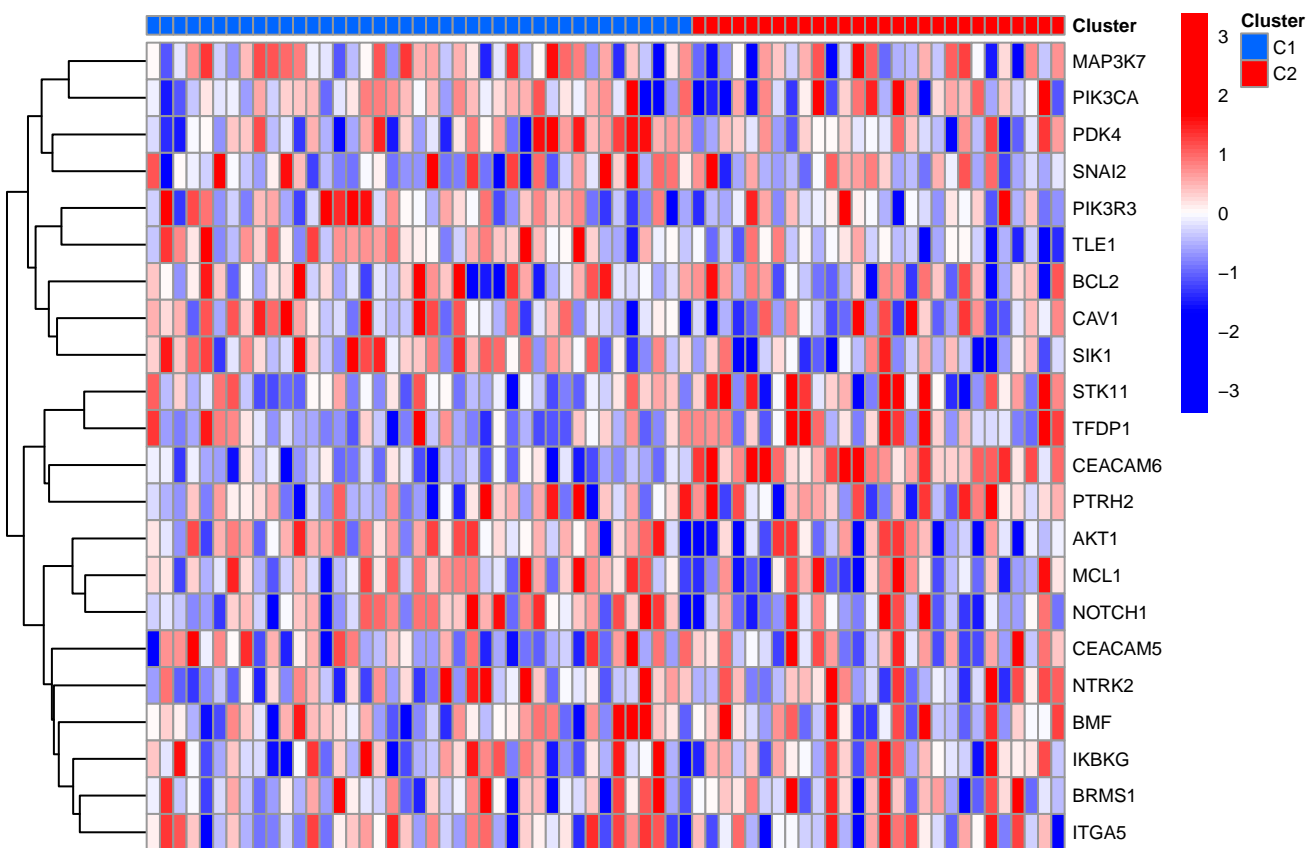

Supplement: Supplementary file 2 [file Data_Sheet_2.ZIP › Fig 5/═╝5/15.heatmap.pdf]

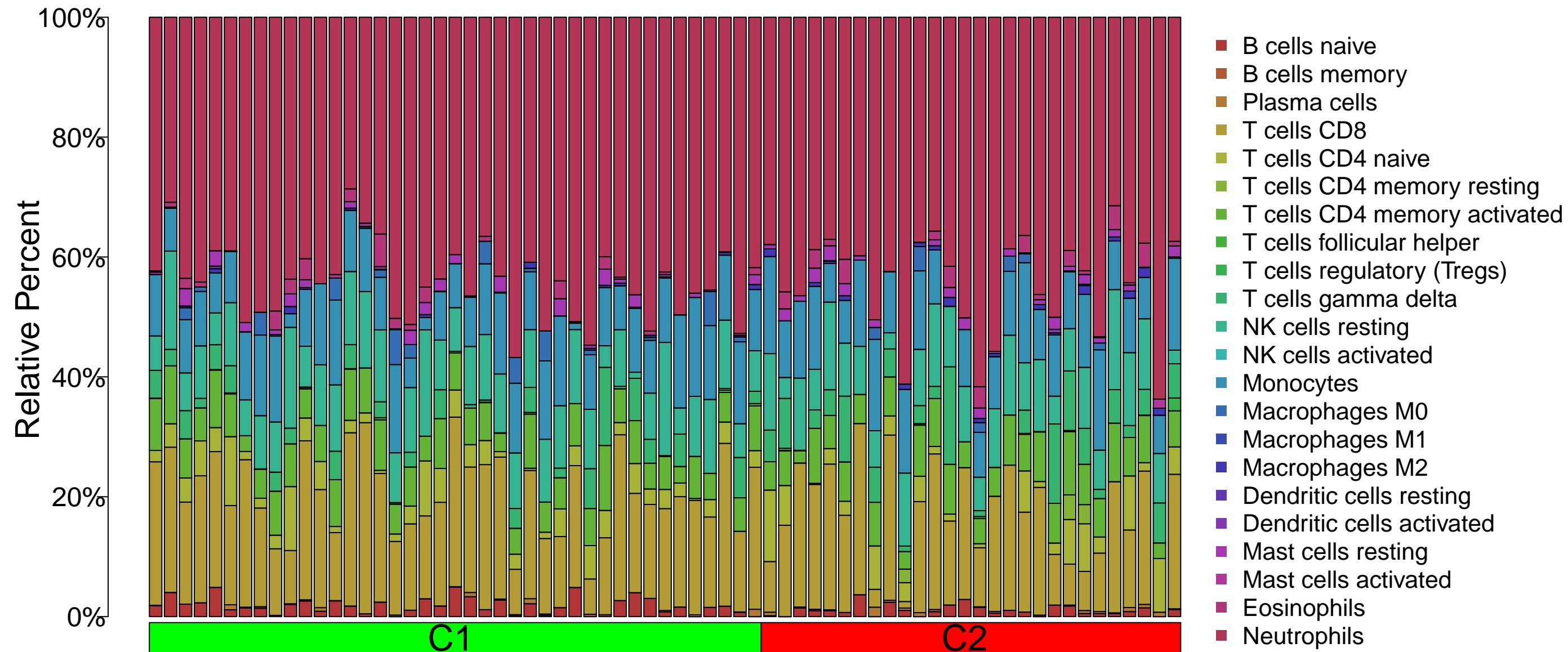

Supplement: Supplementary file 2 [file Data_Sheet_2.ZIP › Fig 5/═╝5/17.barplot.pdf]

Cluster ■ C1 ■ C2

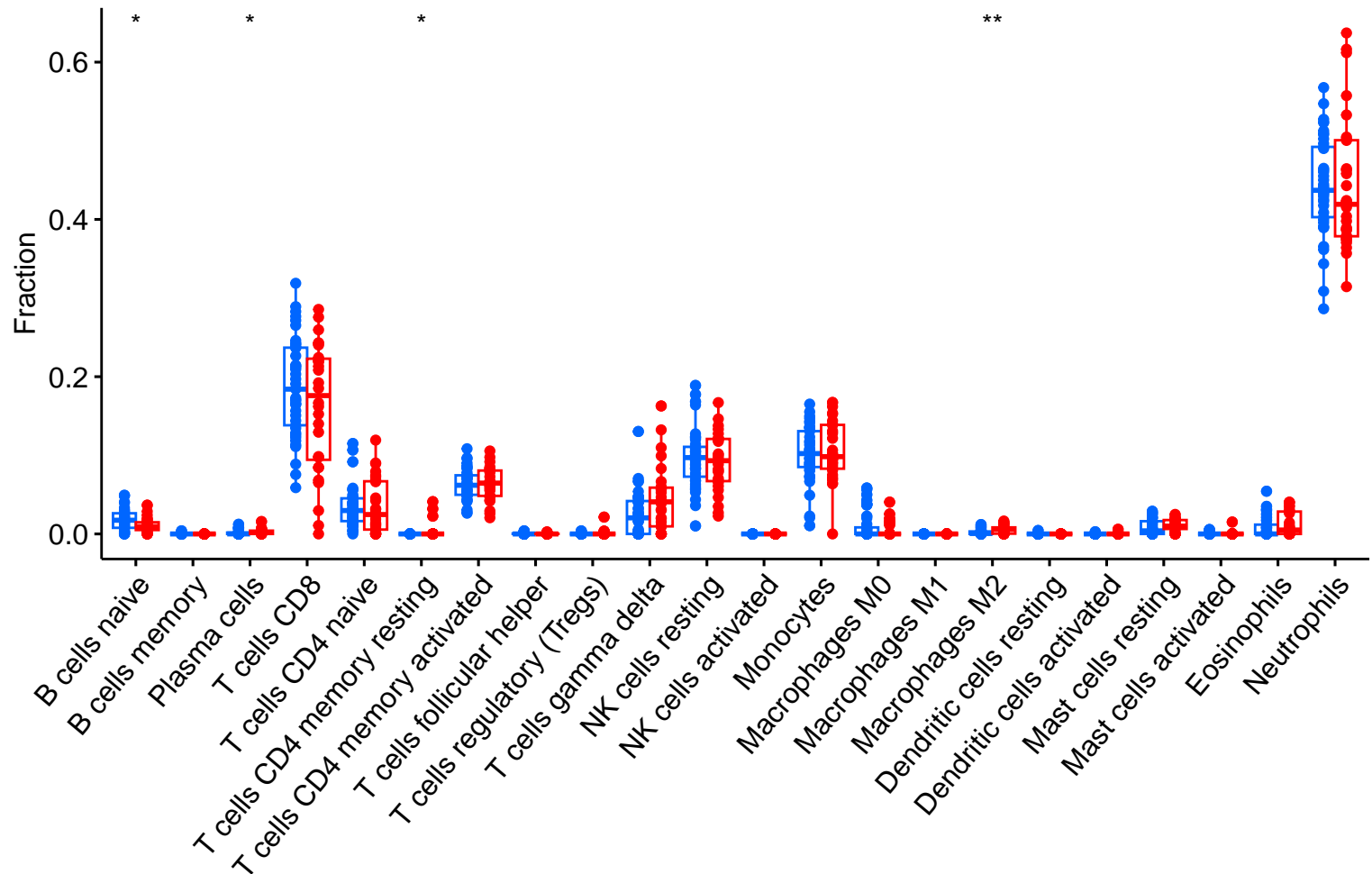

Supplement: Supplementary file 2 [file Data_Sheet_2.ZIP › Fig 5/═╝5/17.immune.diff.pdf]

Term

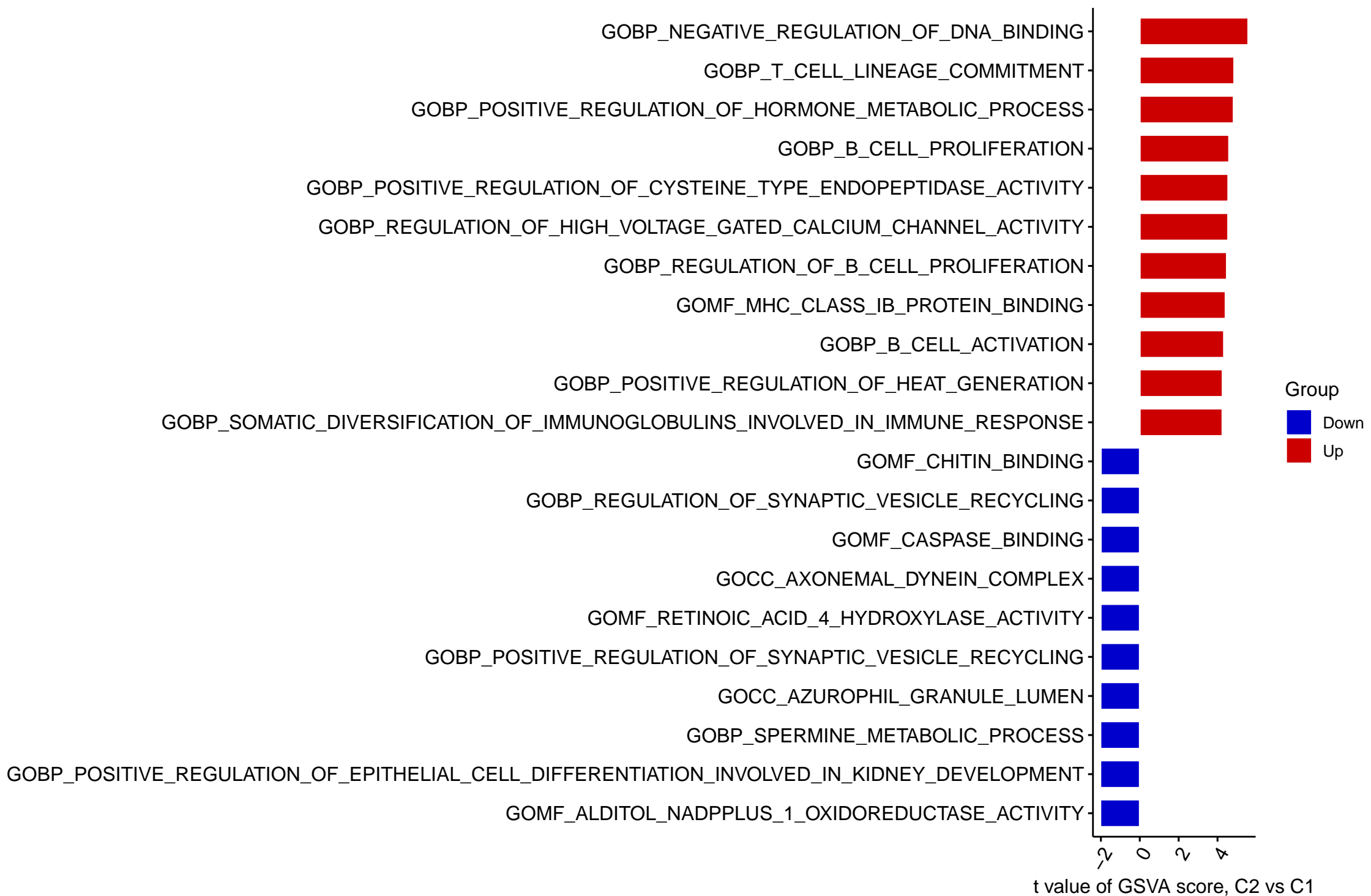

Supplement: Supplementary file 2 [file Data_Sheet_2.ZIP › Fig 5/═╝5/18.GO-barplot.pdf]

Term

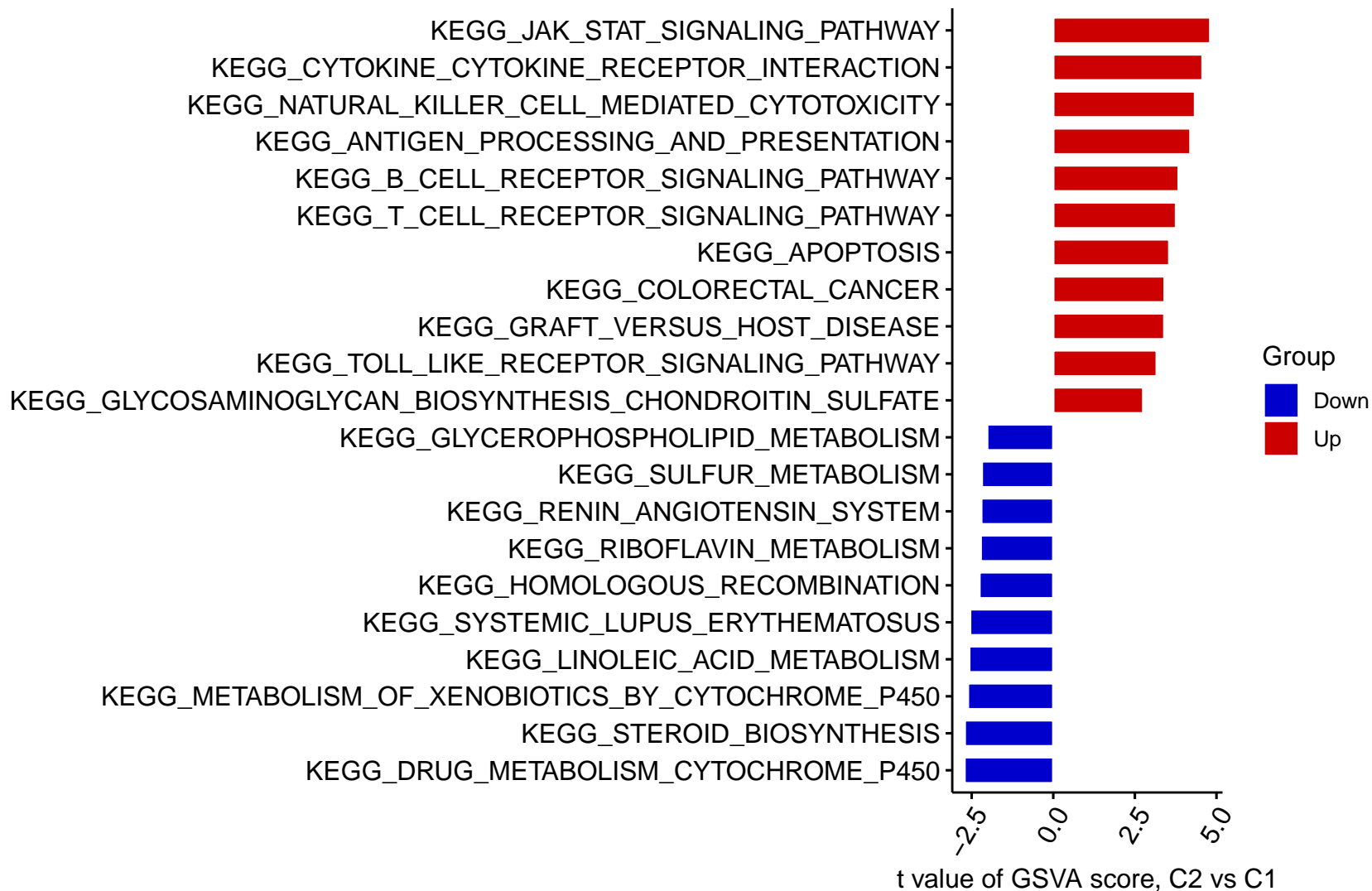

Supplement: Supplementary file 2 [file Data_Sheet_2.ZIP › Fig 5/═╝5/18.KEGG-barplot.pdf]

## Random forest

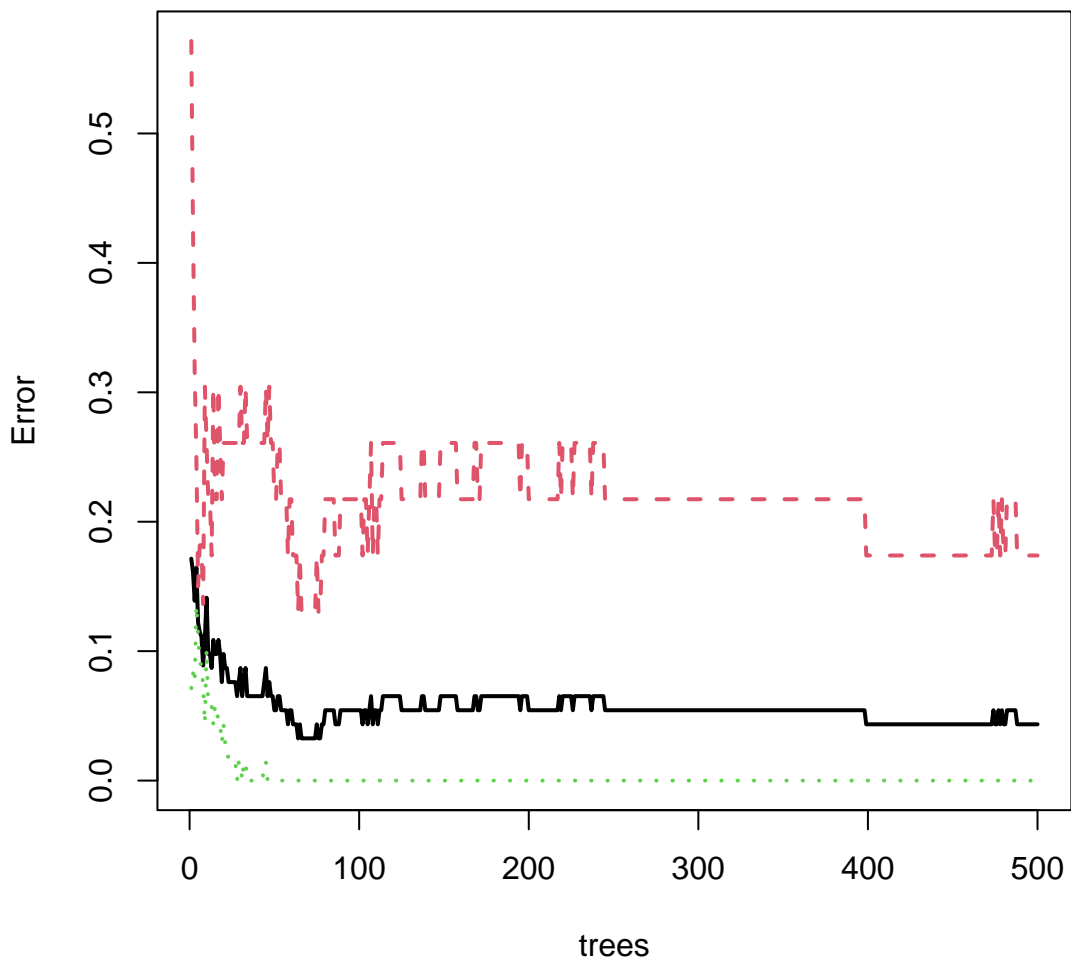

Supplement: Supplementary file 3 [file Data_Sheet_3.ZIP › Fig 6/50.RF/forest.pdf]

SVM-RFE

LASSO

RandomForest

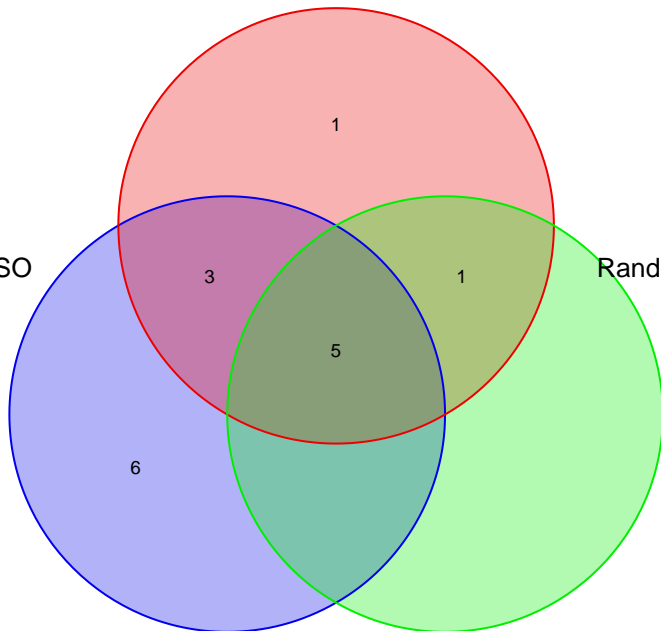

Supplement: Supplementary file 3 [file Data_Sheet_3.ZIP › Fig 6/51.venn/venn.pdf]

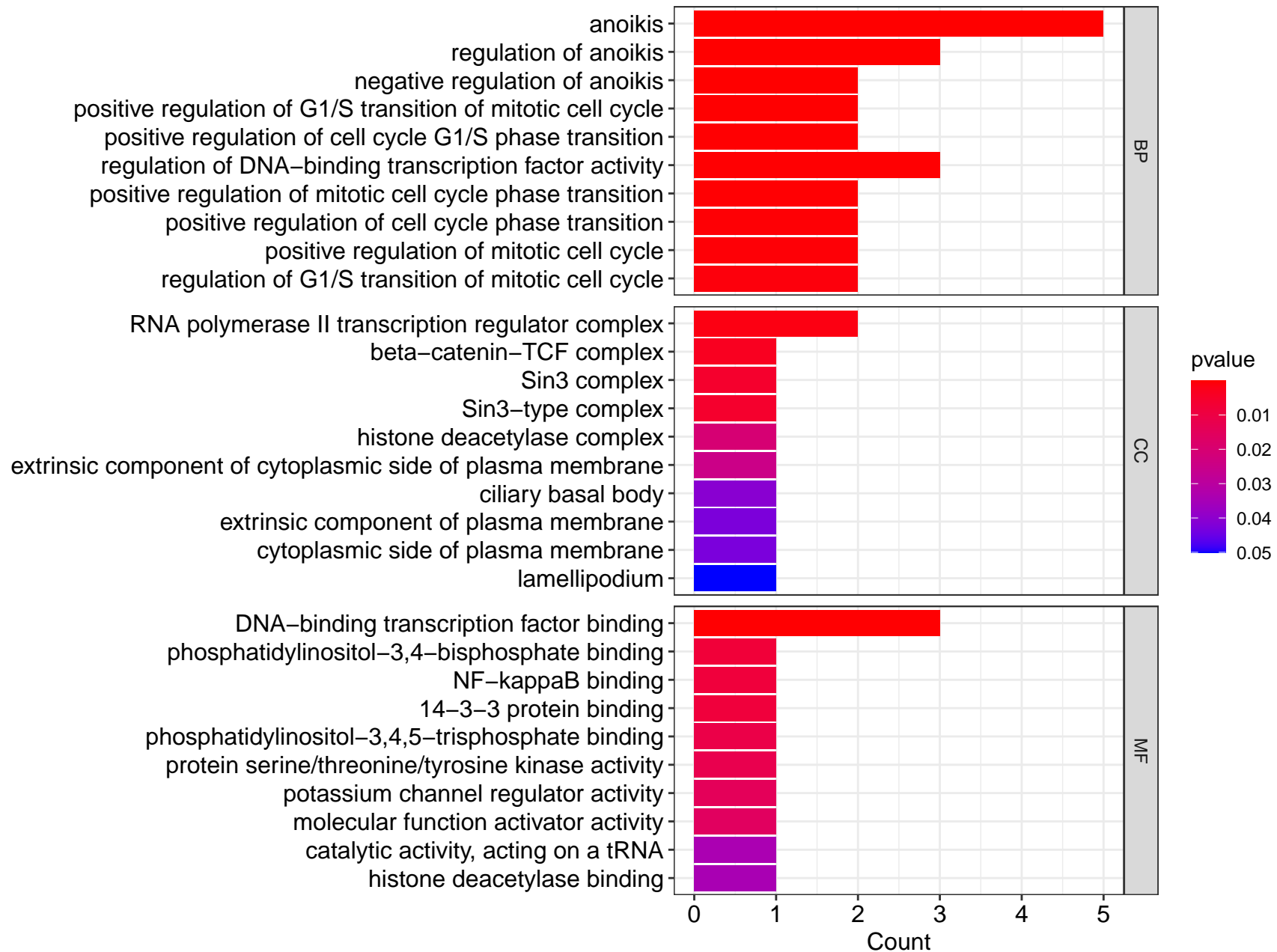

Supplement: Supplementary file 3 [file Data_Sheet_3.ZIP › Fig 6/55.GO-5╕÷╗∙╥≥/barplot.pdf]

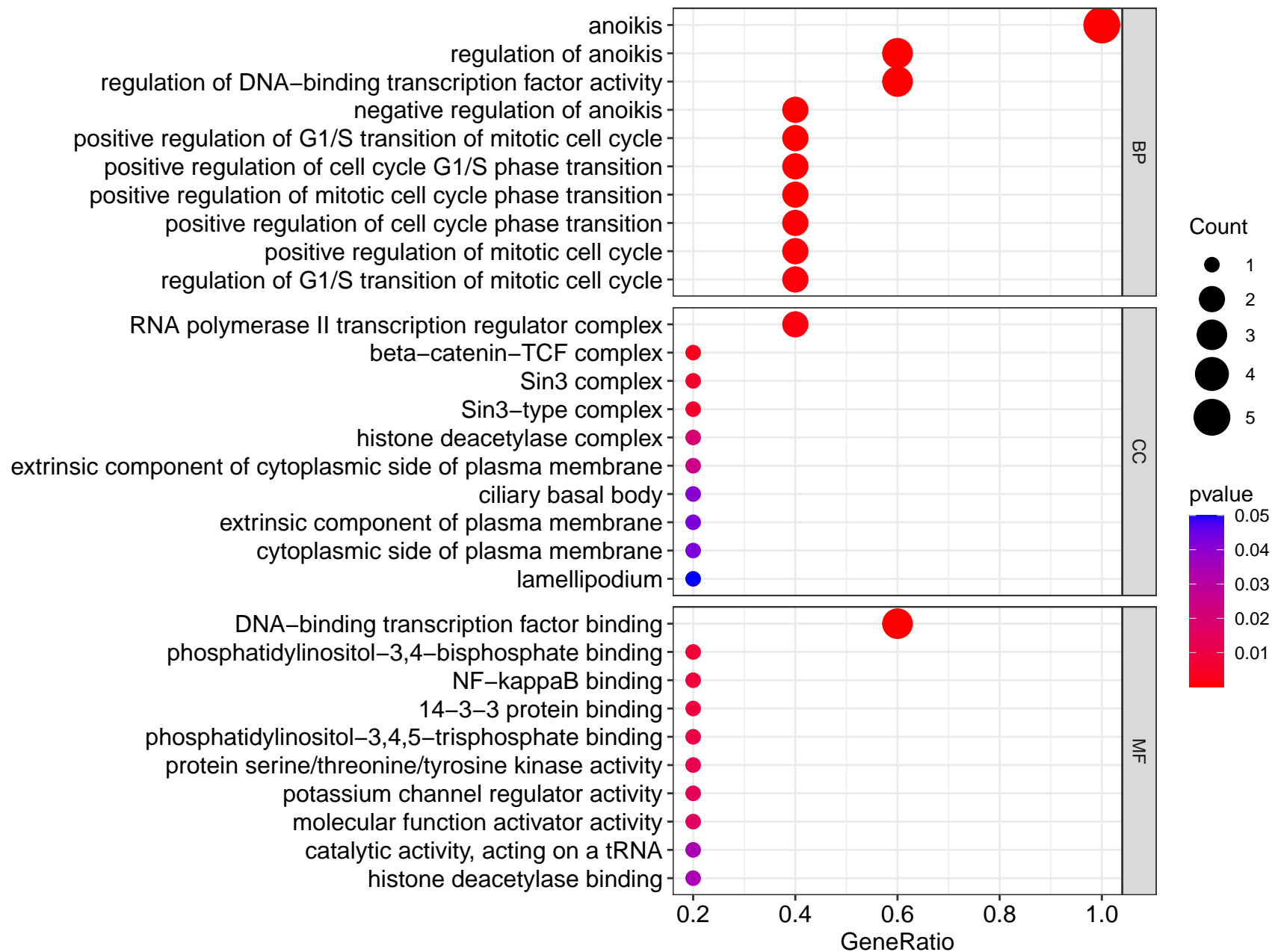

Supplement: Supplementary file 3 [file Data_Sheet_3.ZIP › Fig 6/55.GO-5╕÷╗∙╥≥/bubble.pdf]

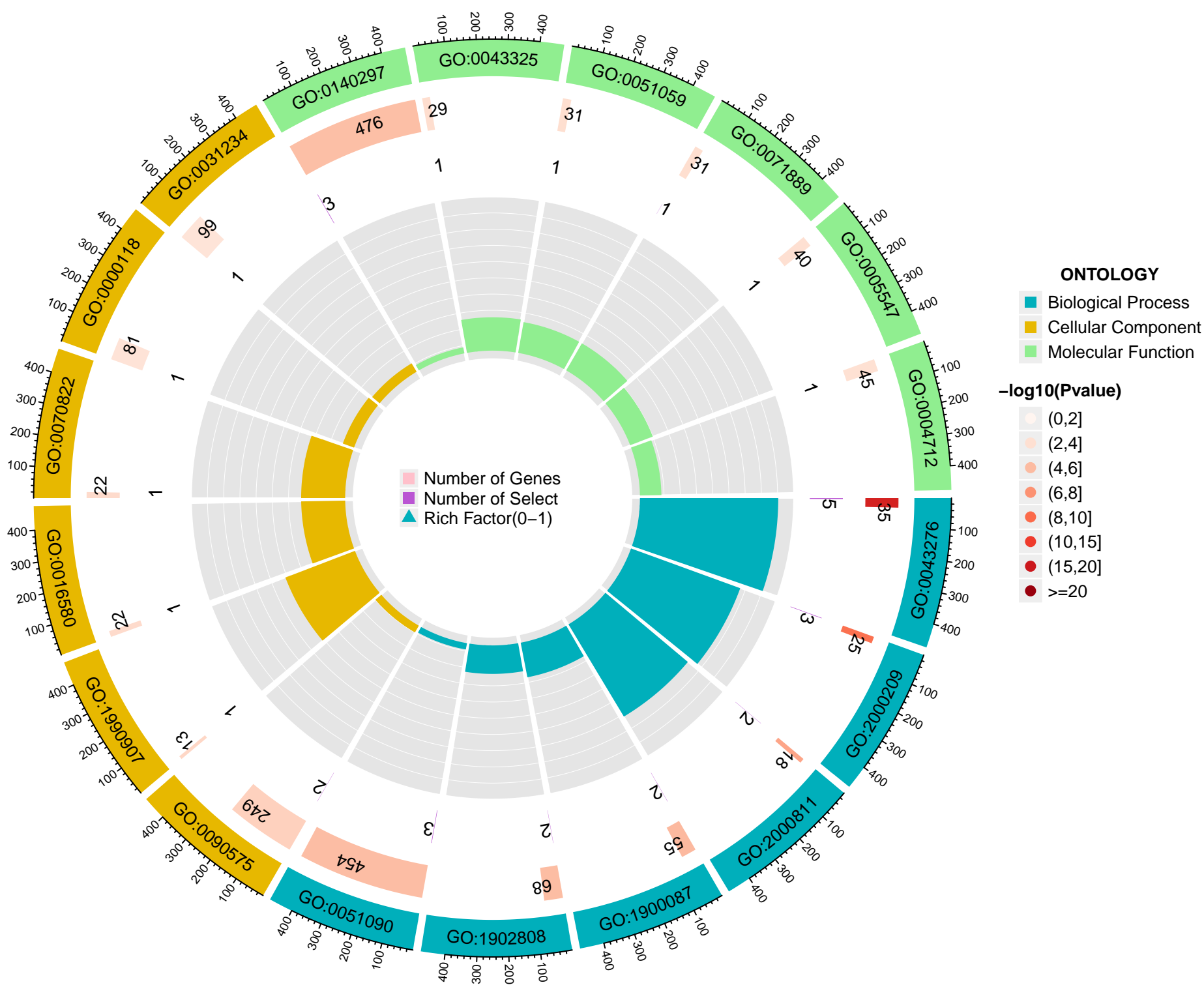

Supplement: Supplementary file 3 [file Data_Sheet_3.ZIP › Fig 6/55.GO-5╕÷╗∙╥≥/GO.circlize.pdf]

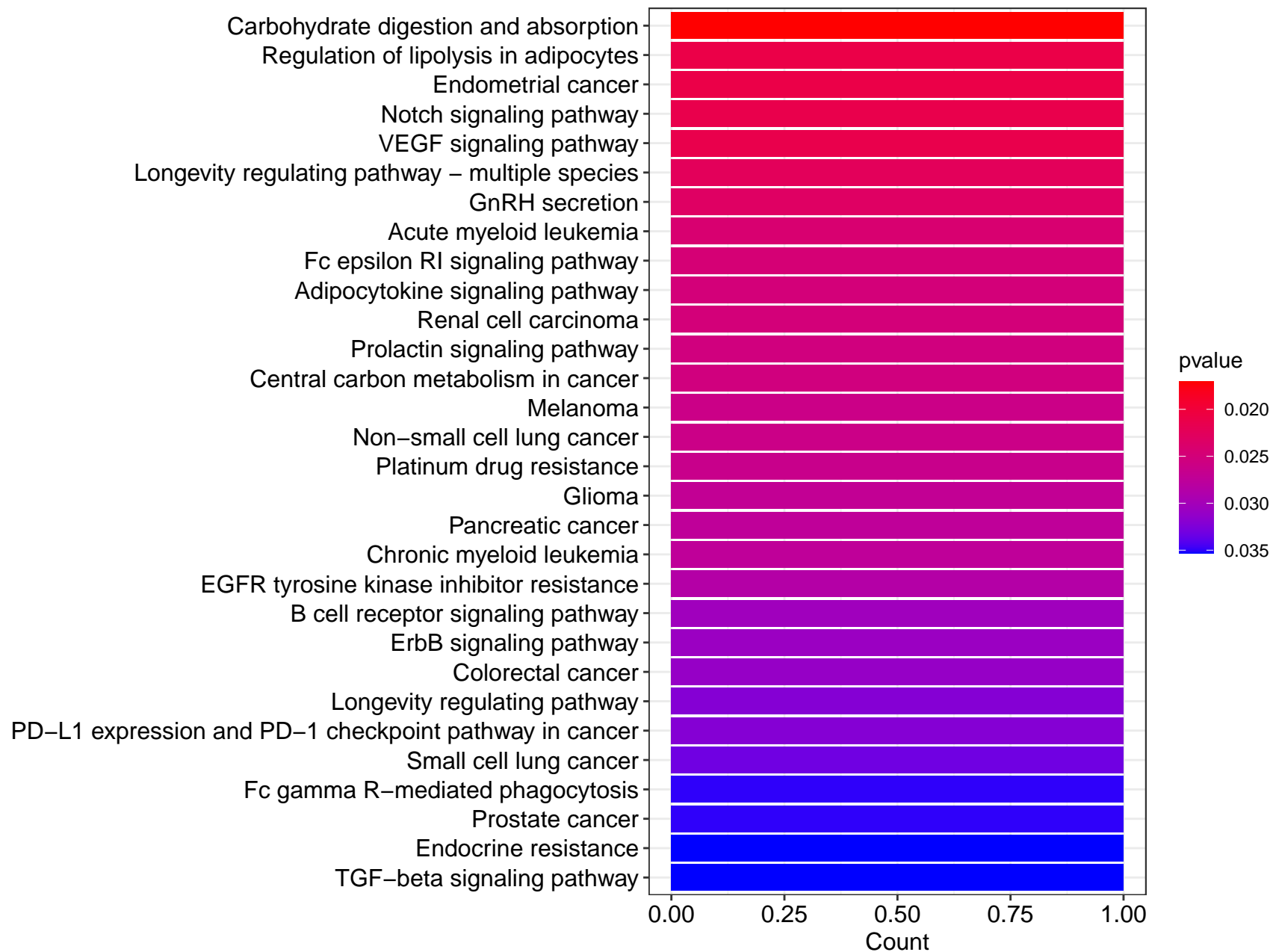

Supplement: Supplementary file 3 [file Data_Sheet_3.ZIP › Fig 6/56.KEGG-5╕÷╗∙╥≥/barplot.pdf]

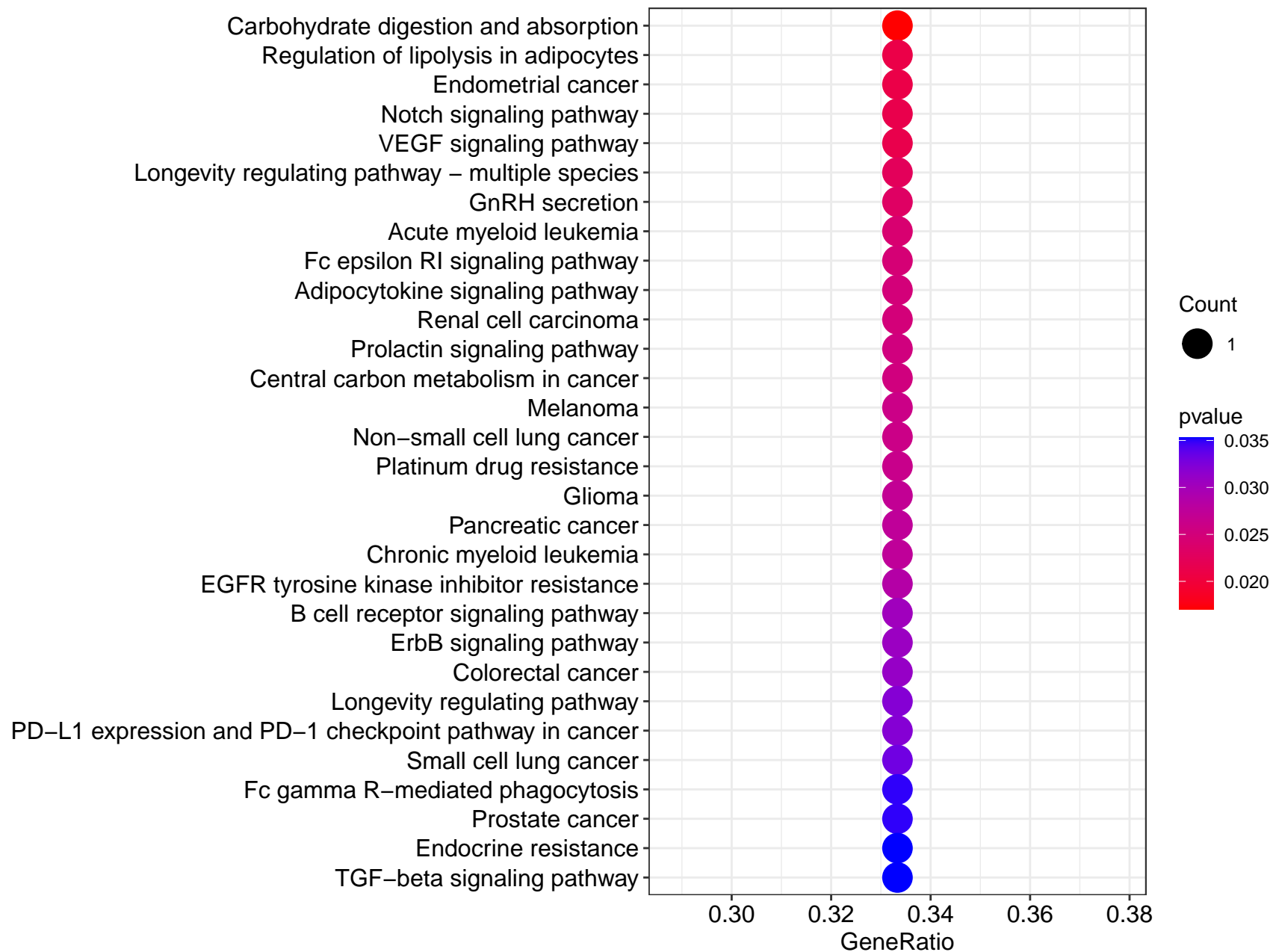

Supplement: Supplementary file 3 [file Data_Sheet_3.ZIP › Fig 6/56.KEGG-5╕÷╗∙╥≥/bubble.pdf]

Binomial Deviance

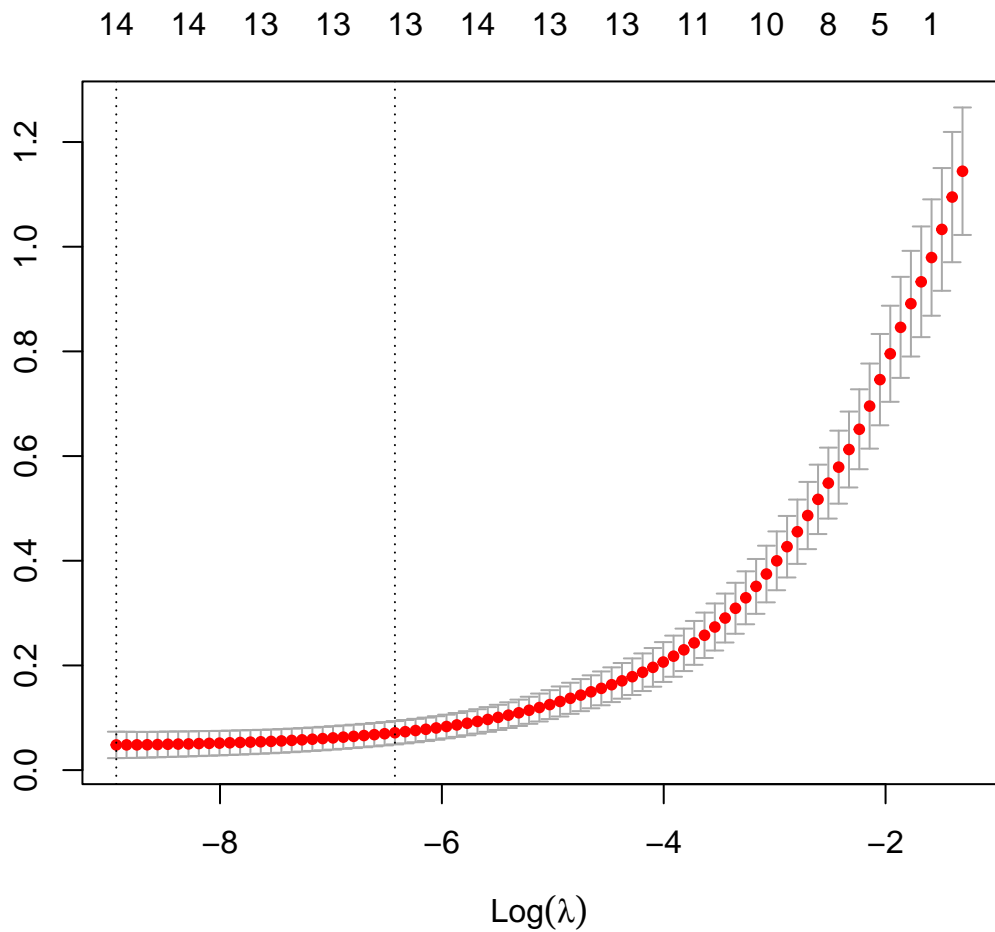

Supplement: Supplementary file 3 [file Data_Sheet_3.ZIP › Fig 6/═╝6/19.cvfit.pdf]

Coefficients

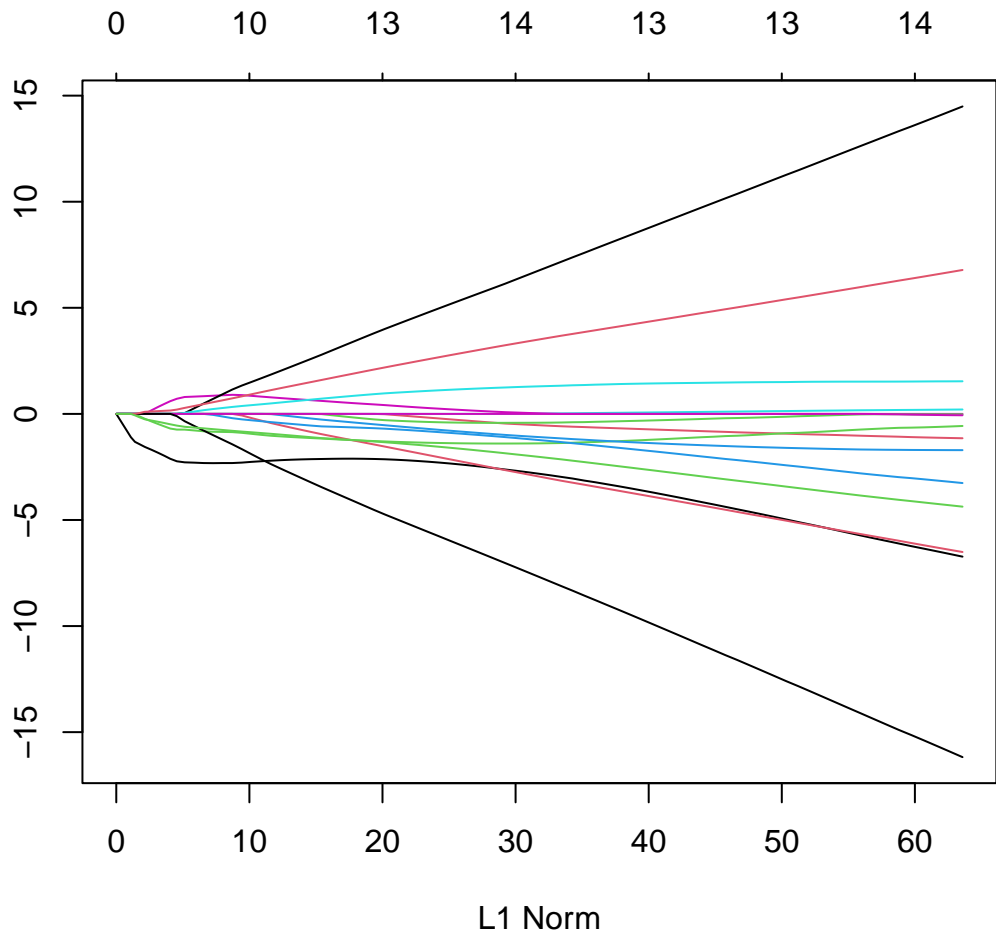

Supplement: Supplementary file 3 [file Data_Sheet_3.ZIP › Fig 6/═╝6/19.lasso.pdf]

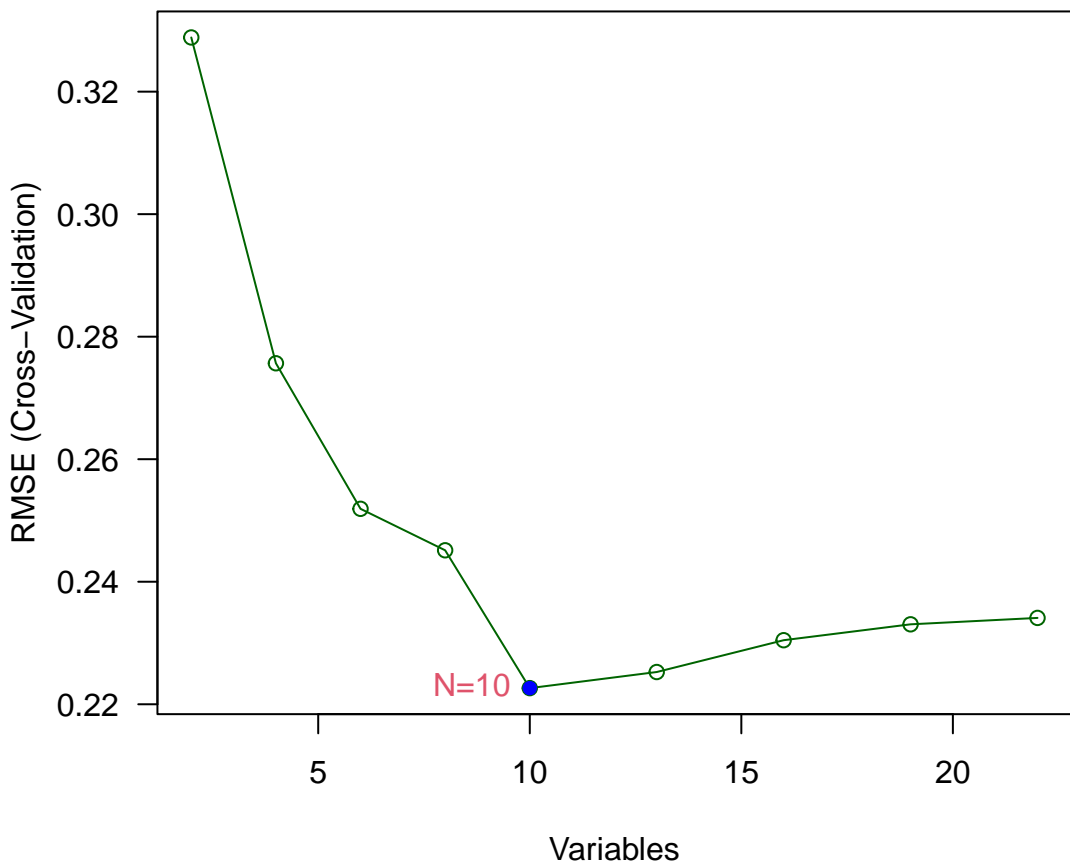

Supplement: Supplementary file 3 [file Data_Sheet_3.ZIP › Fig 6/═╝6/20.SVM-RFE.pdf]

LASSO

SVM-RFE

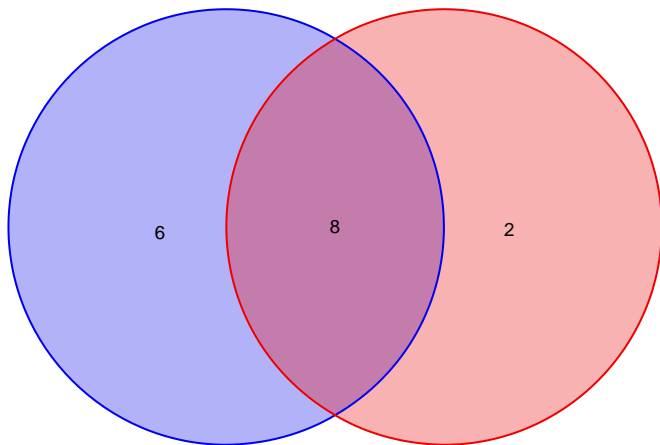

Supplement: Supplementary file 3 [file Data_Sheet_3.ZIP › Fig 6/═╝6/21.venn.pdf]

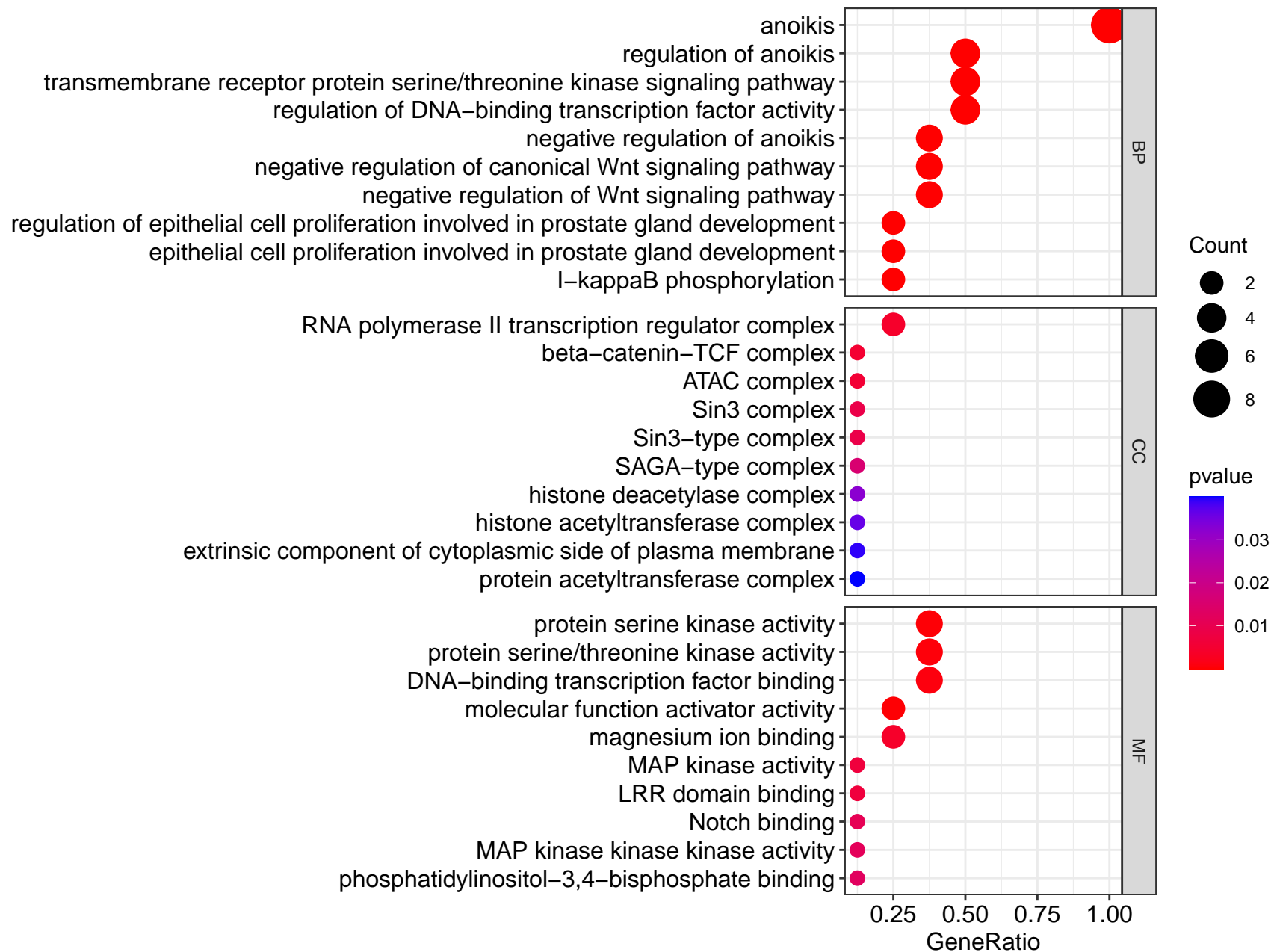

Supplement: Supplementary file 3 [file Data_Sheet_3.ZIP › Fig 6/═╝6/bubble.pdf]

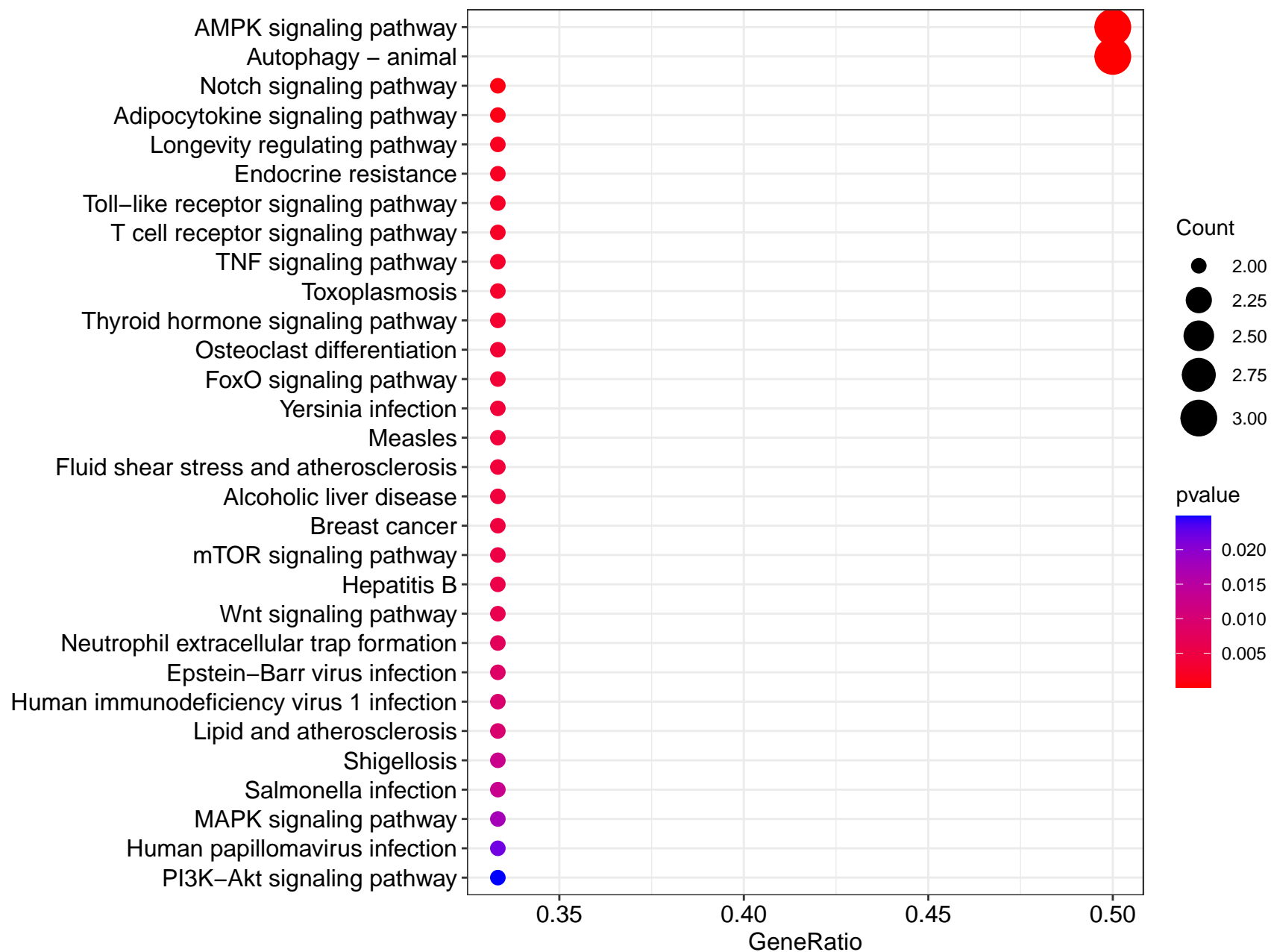

Supplement: Supplementary file 3 [file Data_Sheet_3.ZIP › Fig 6/═╝6/KEGG-bubble.pdf]

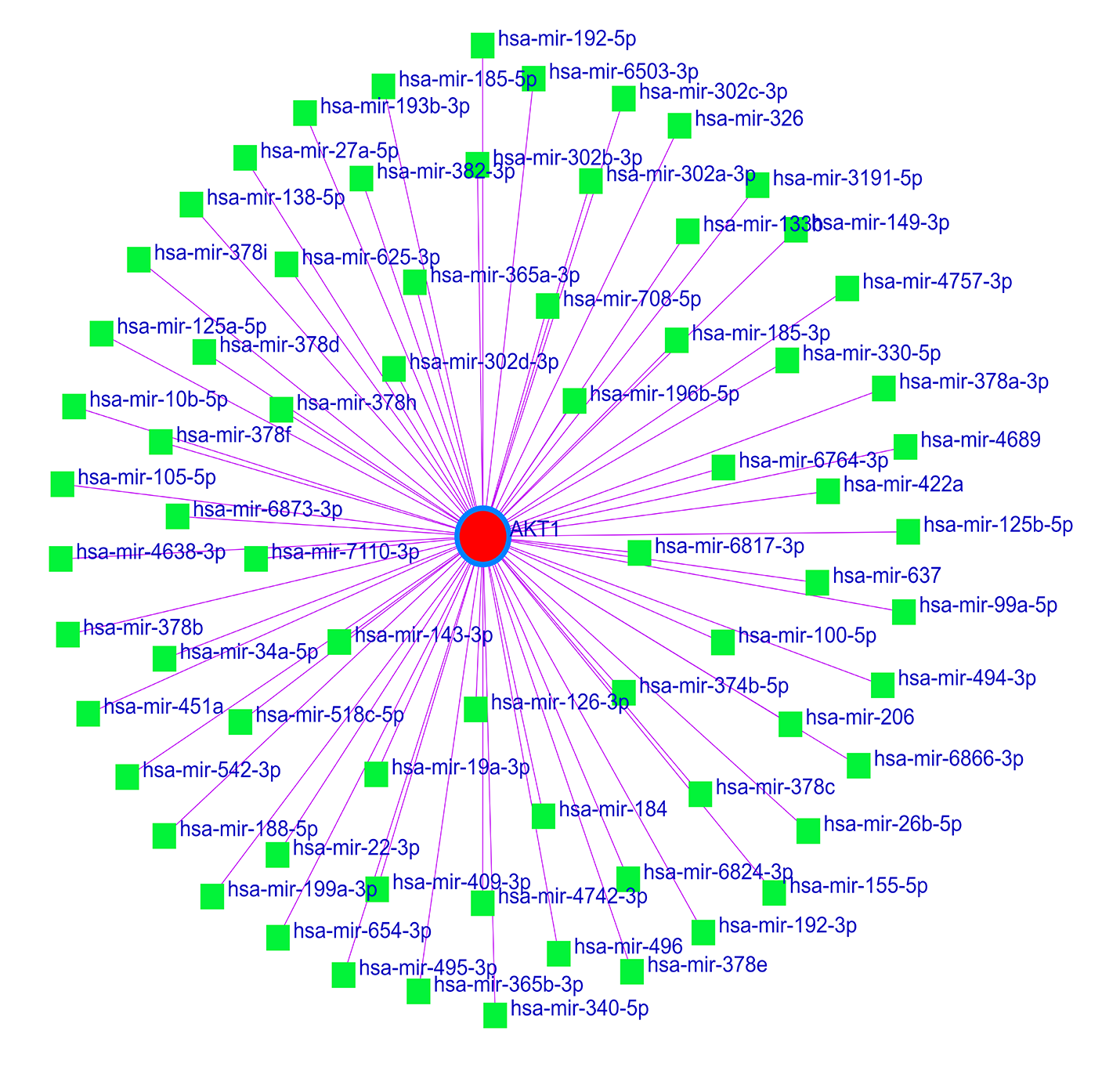

Supplement: Supplementary file 4 [file Data_Sheet_4.ZIP › Fig 10/Fig 10/AKT1.png]

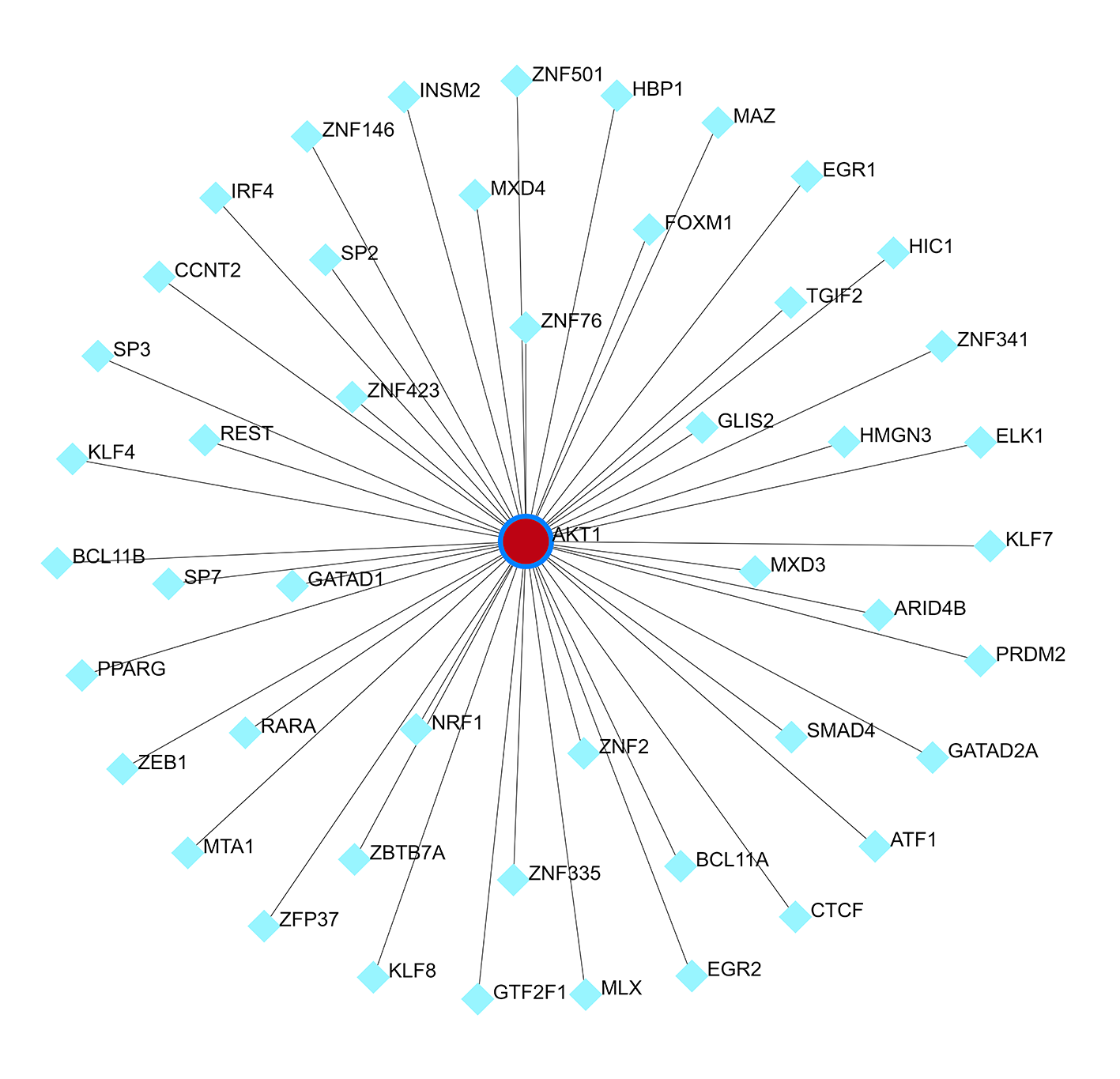

Supplement: Supplementary file 4 [file Data_Sheet_4.ZIP › Fig 10/Fig 10/akt1-tf.png]

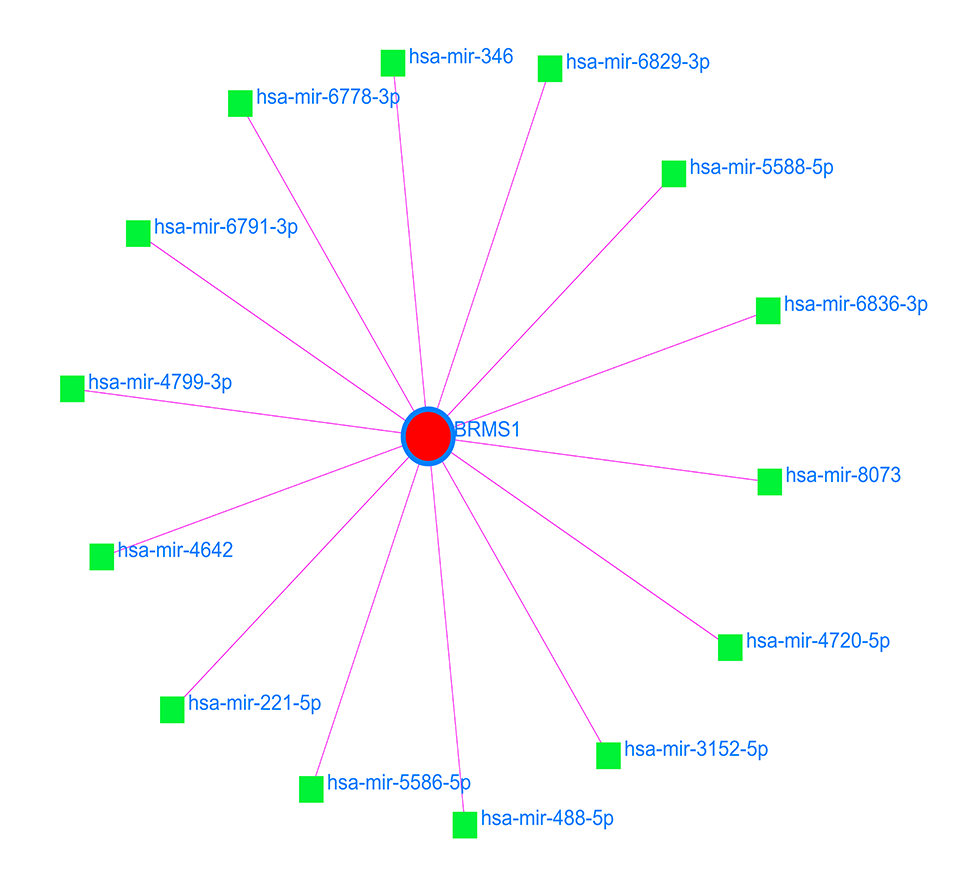

Supplement: Supplementary file 4 [file Data_Sheet_4.ZIP › Fig 10/Fig 10/BRSM1.png]

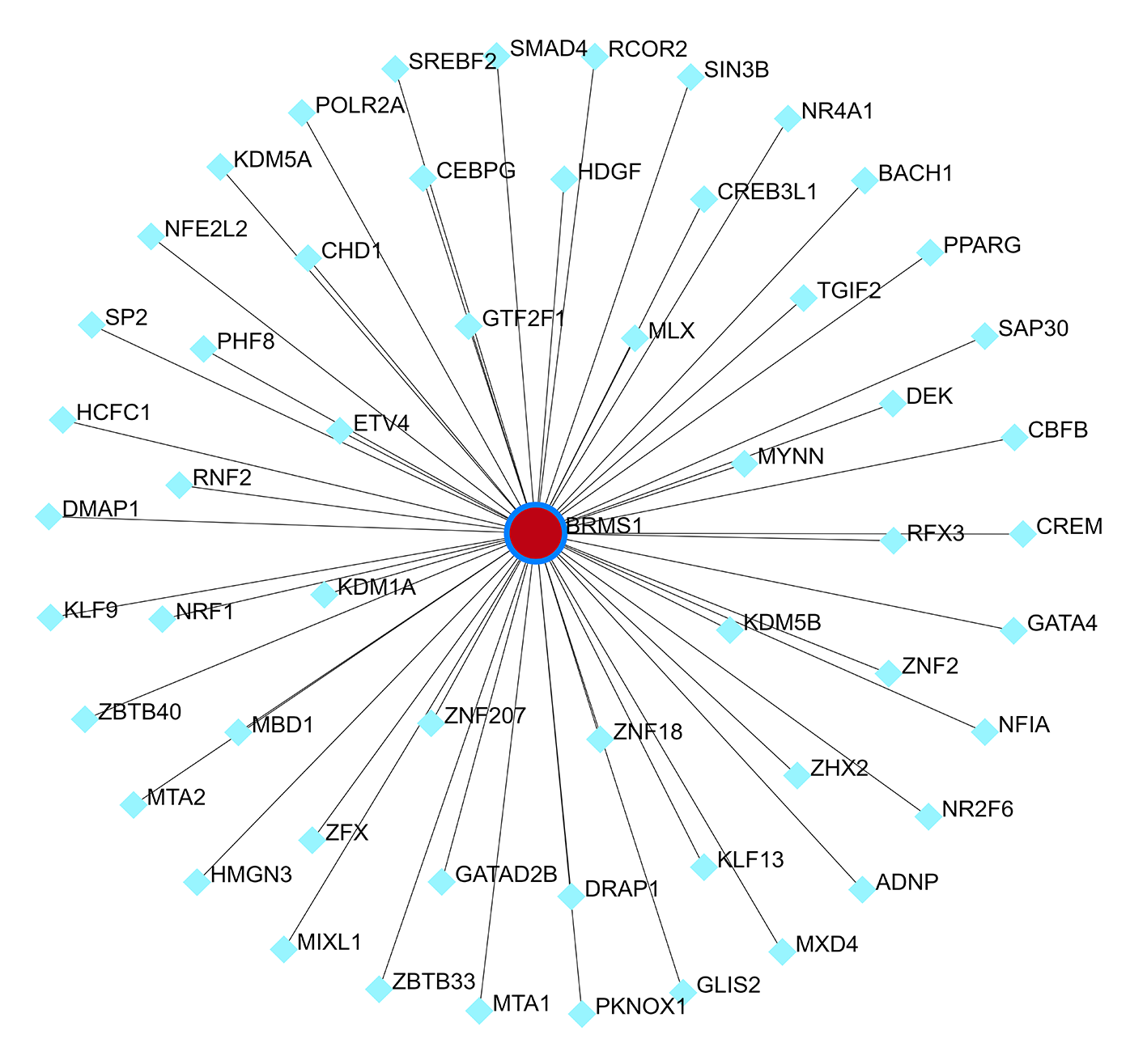

Supplement: Supplementary file 4 [file Data_Sheet_4.ZIP › Fig 10/Fig 10/brsm1-tf.png]

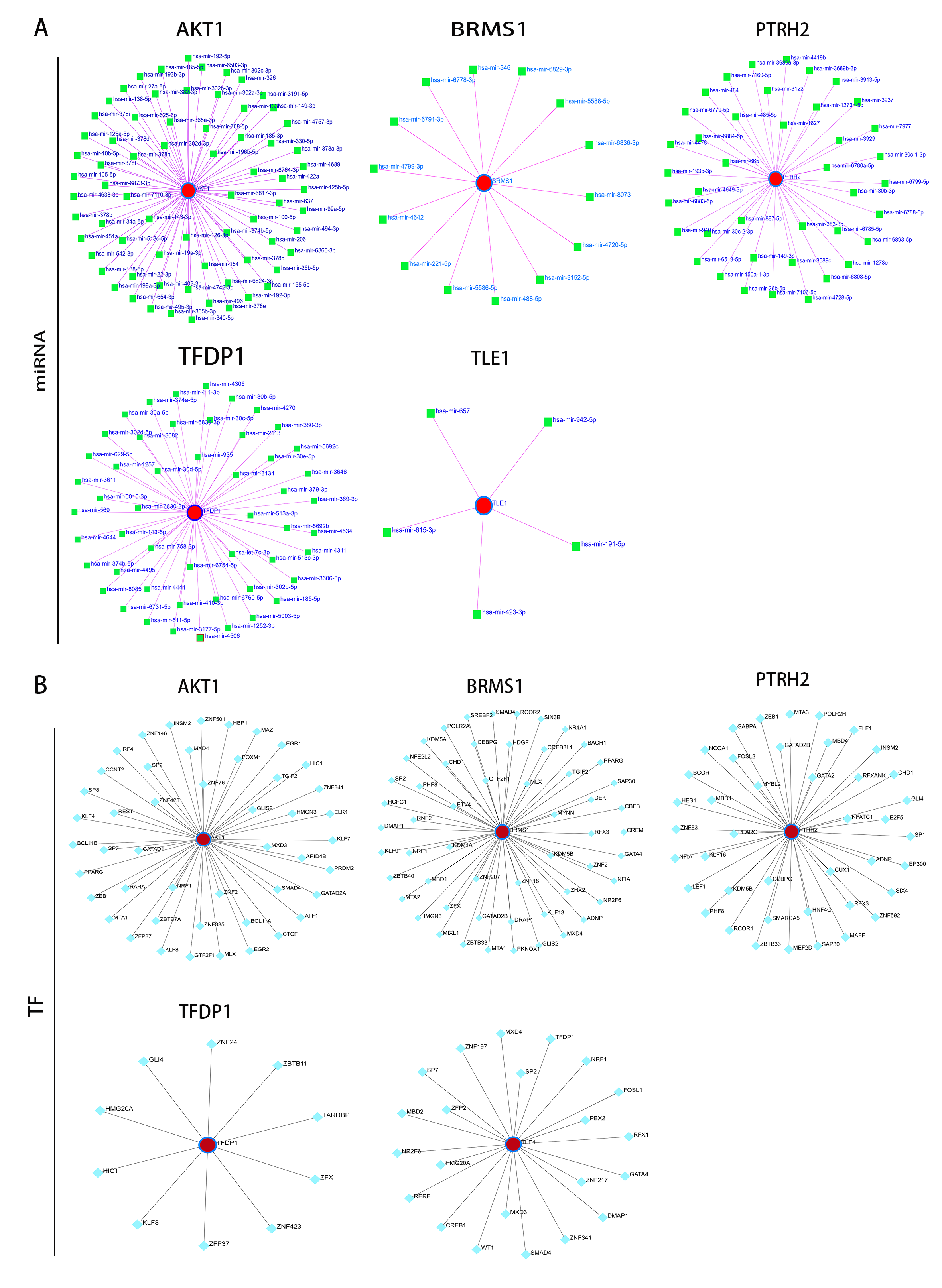

Supplement: Supplementary file 4 [file Data_Sheet_4.ZIP › Fig 10/Fig 10/Fig 10.tif]

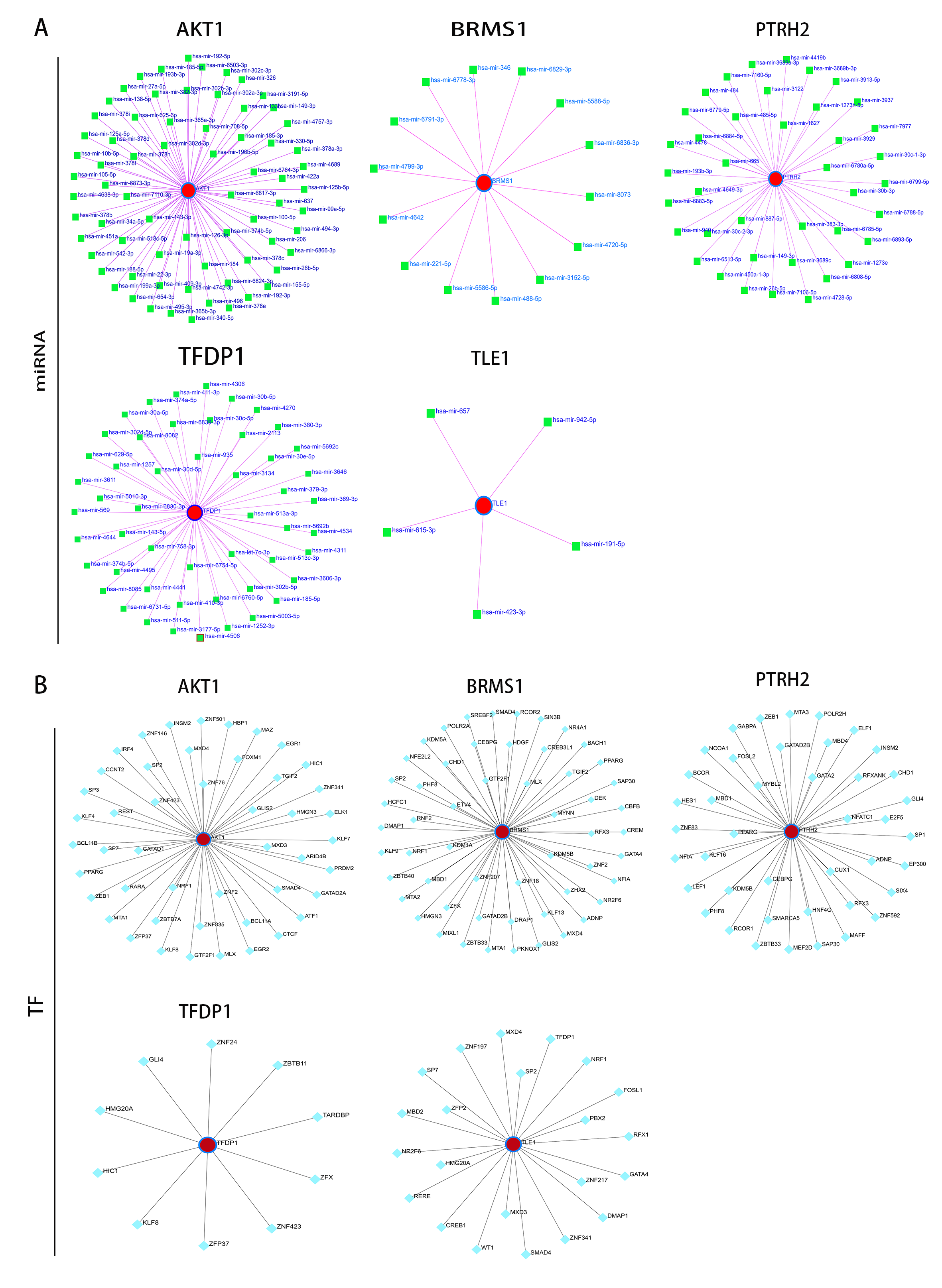

Supplement: Supplementary file 4 [file Data_Sheet_4.ZIP › Fig 10/Fig 10/miRNA/╨▐╕─═╝8.tif]

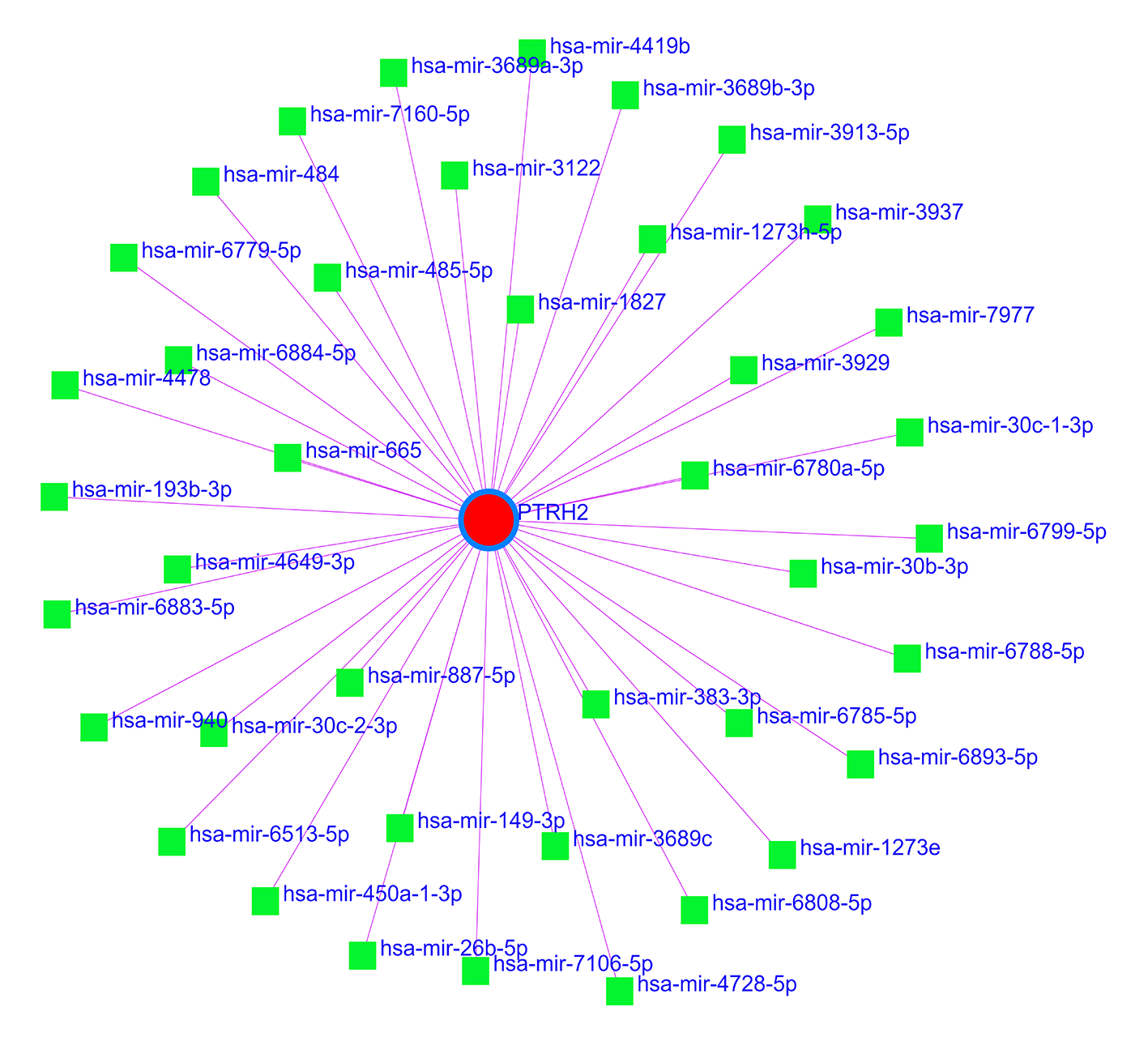

Supplement: Supplementary file 4 [file Data_Sheet_4.ZIP › Fig 10/Fig 10/PTHR2.png]

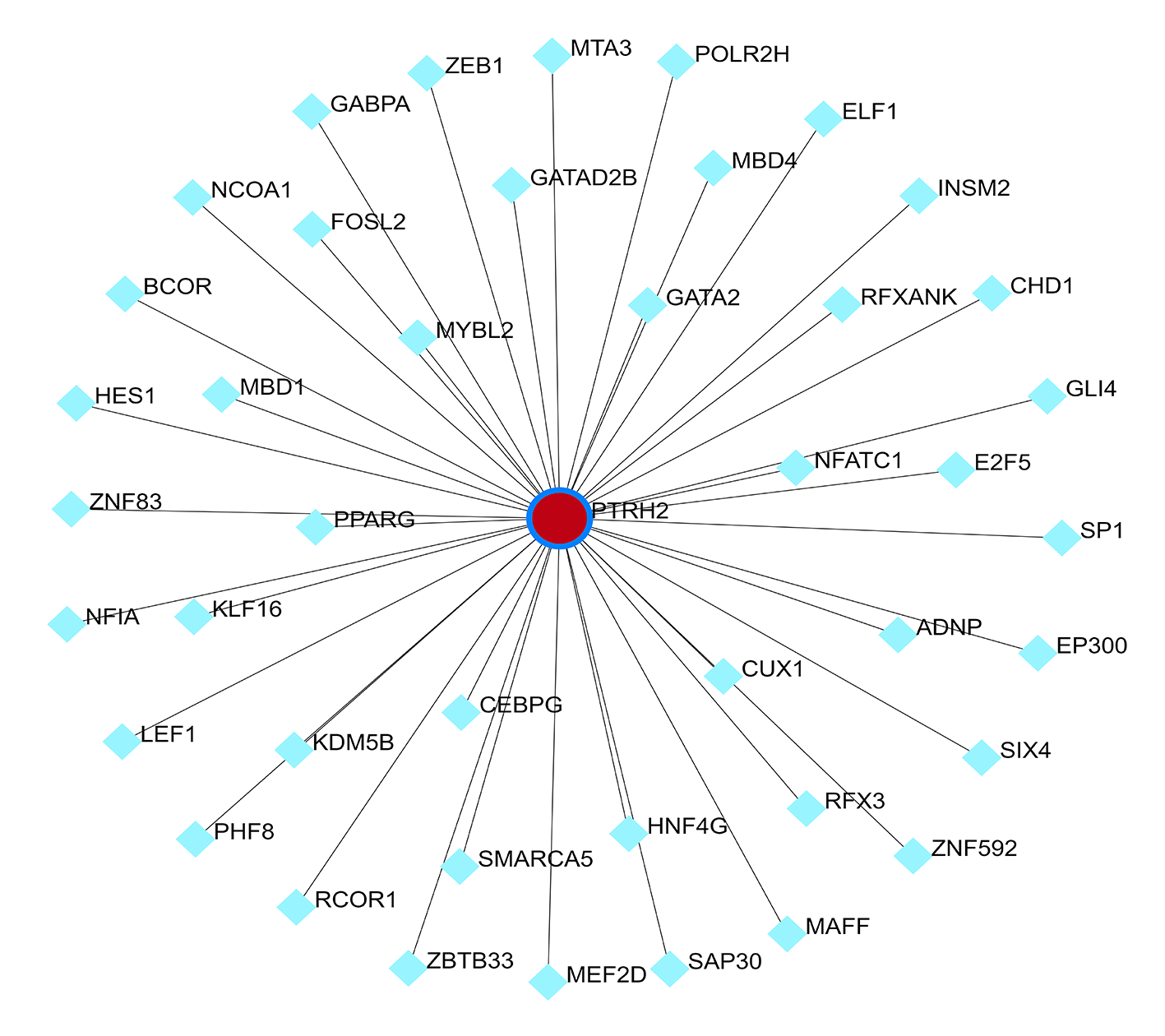

Supplement: Supplementary file 4 [file Data_Sheet_4.ZIP › Fig 10/Fig 10/ptrh2-tf.png]

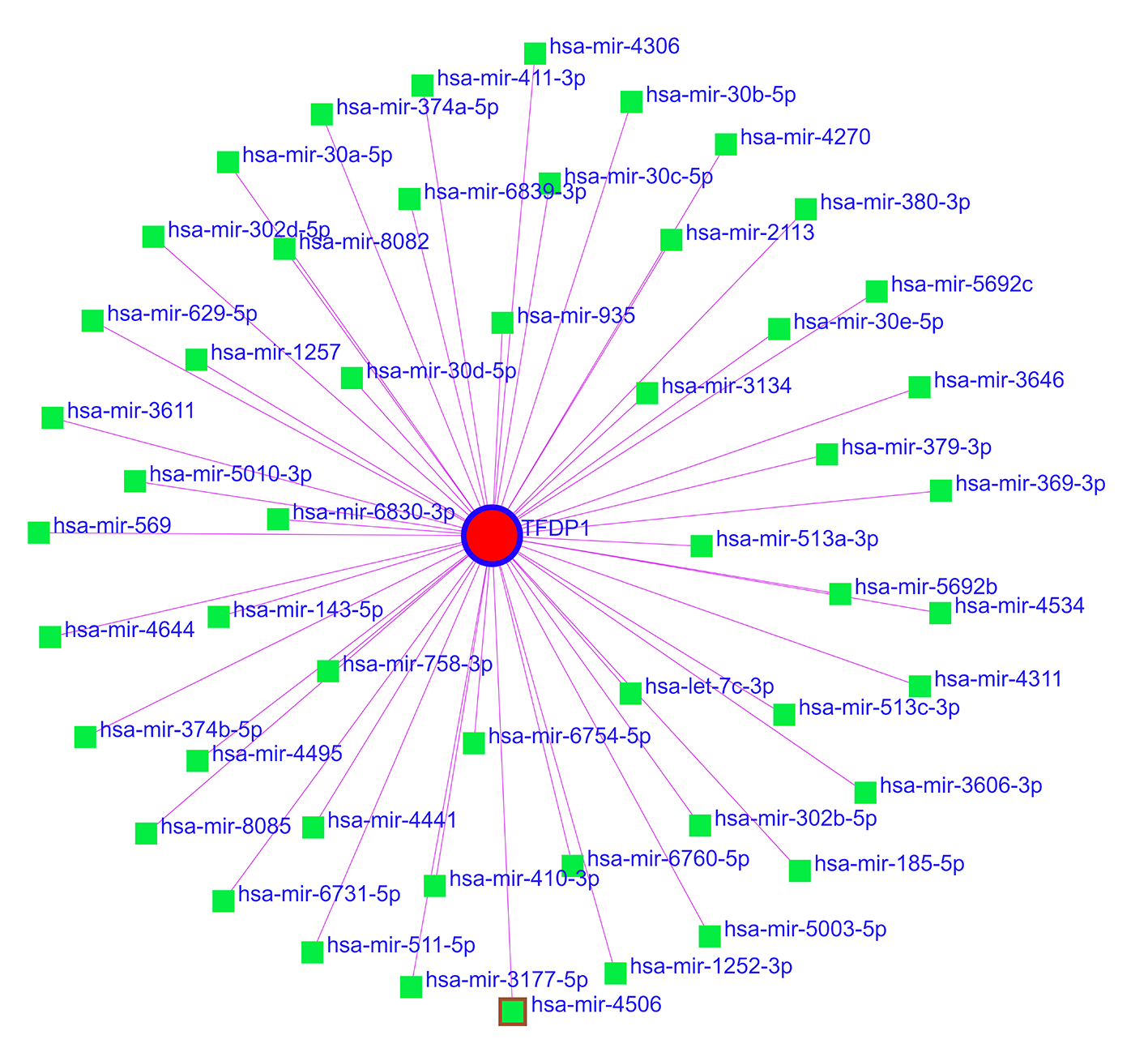

Supplement: Supplementary file 4 [file Data_Sheet_4.ZIP › Fig 10/Fig 10/TFDP1.png]

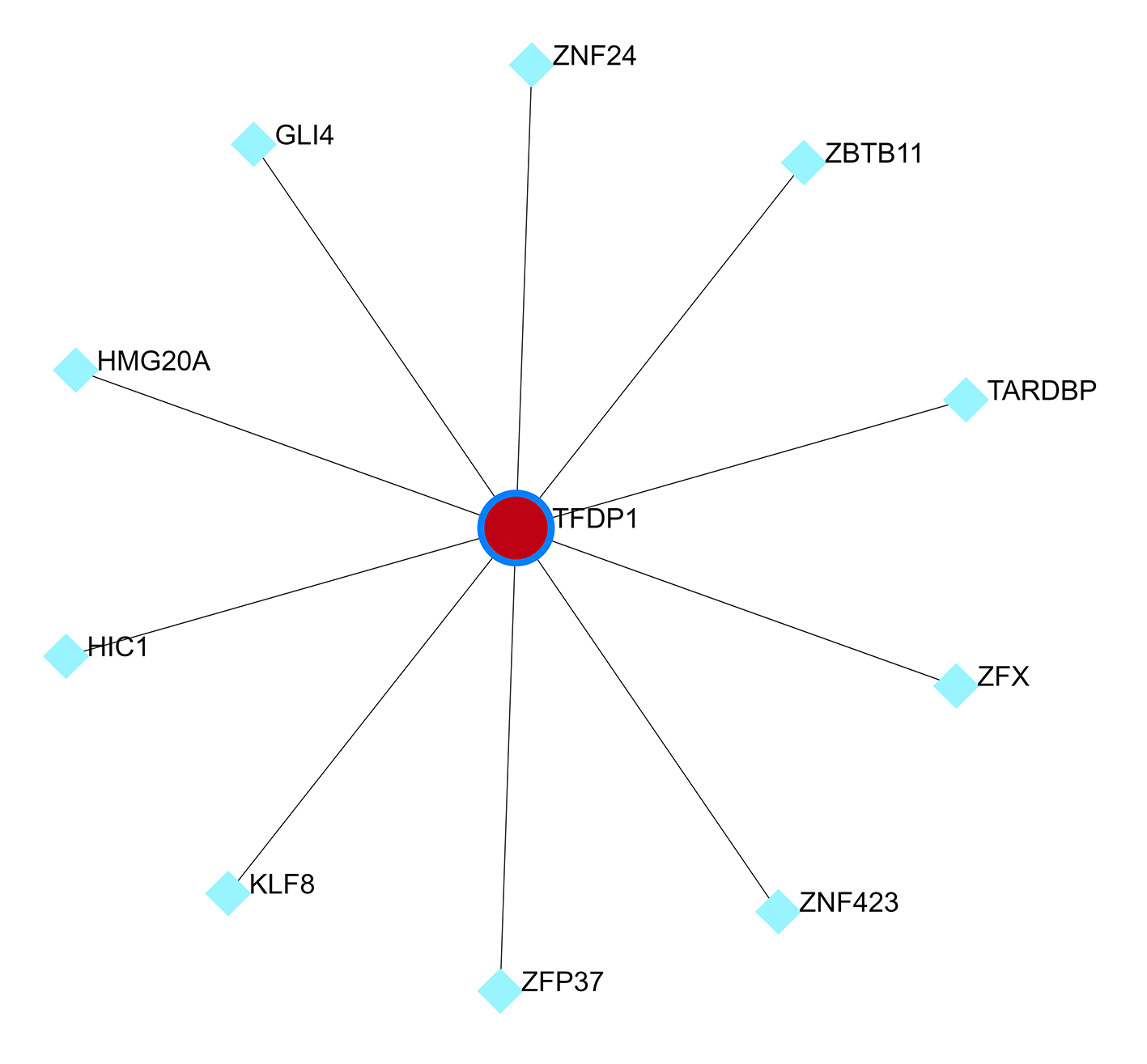

Supplement: Supplementary file 4 [file Data_Sheet_4.ZIP › Fig 10/Fig 10/tfdp1-tf.png]

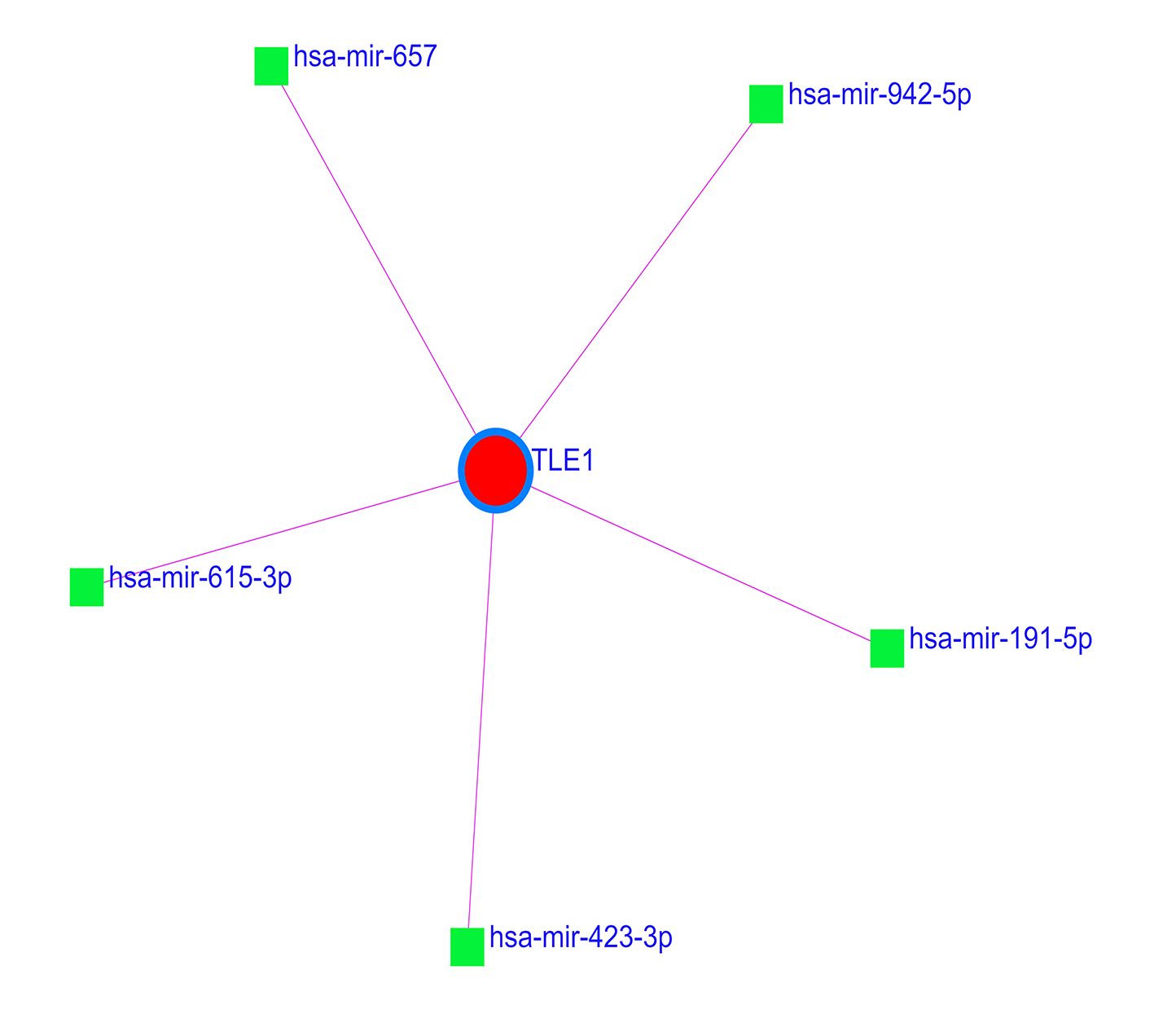

Supplement: Supplementary file 4 [file Data_Sheet_4.ZIP › Fig 10/Fig 10/TLE1.png]

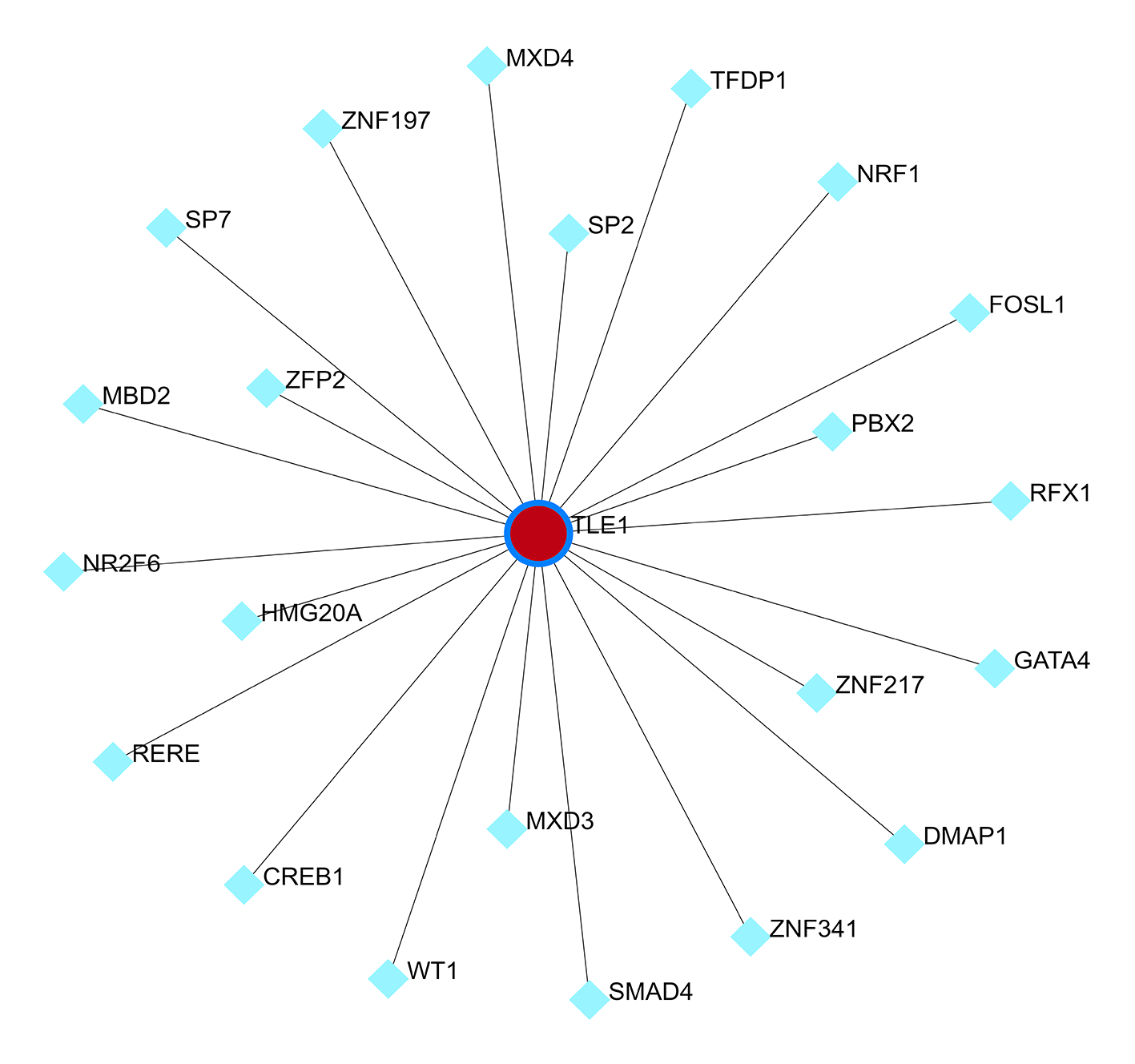

Supplement: Supplementary file 4 [file Data_Sheet_4.ZIP › Fig 10/Fig 10/tle1-tf.png]

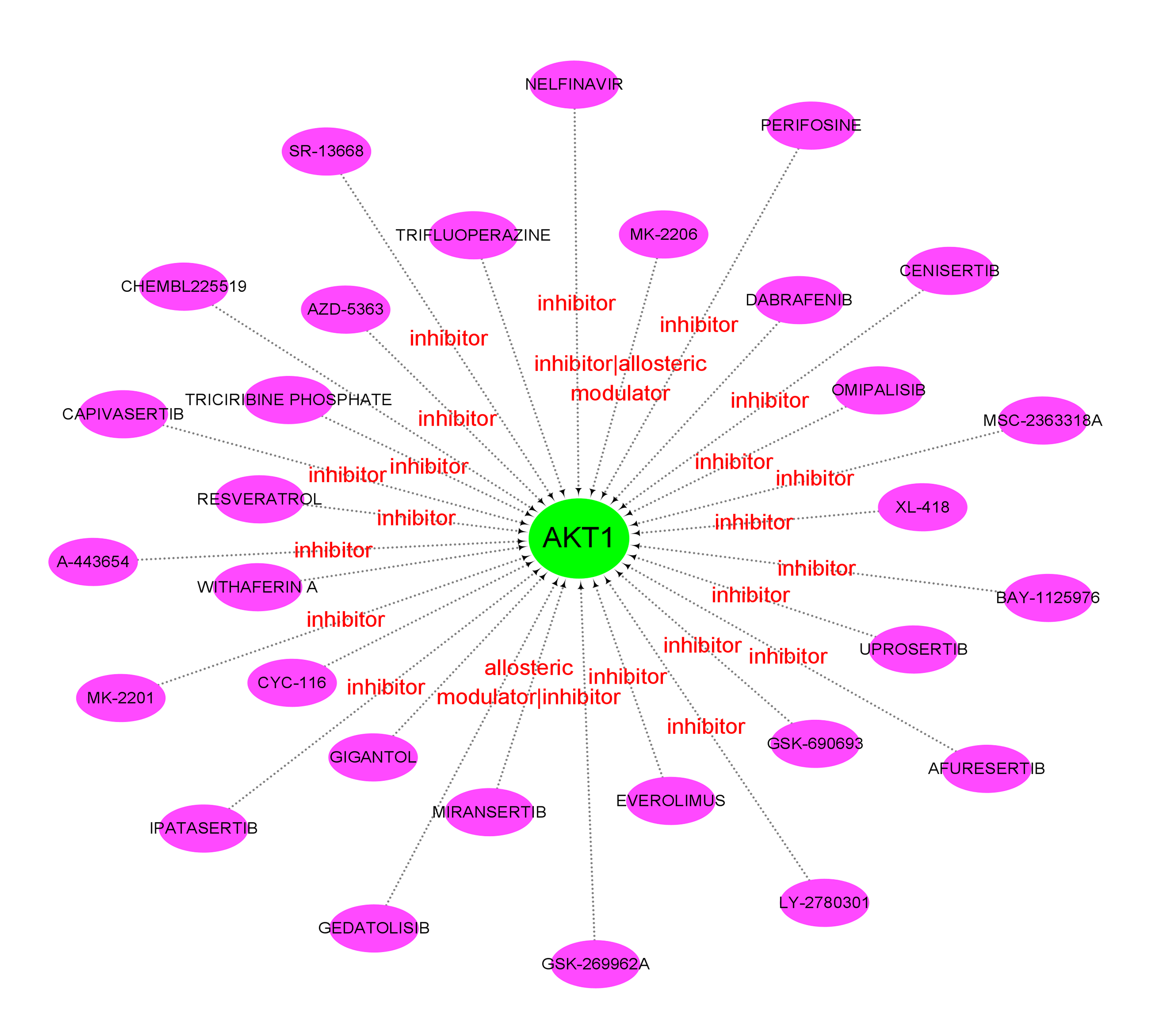

Supplement: Supplementary file 5 [file Data_Sheet_5.ZIP › Fig 11/Fig 11.tif]

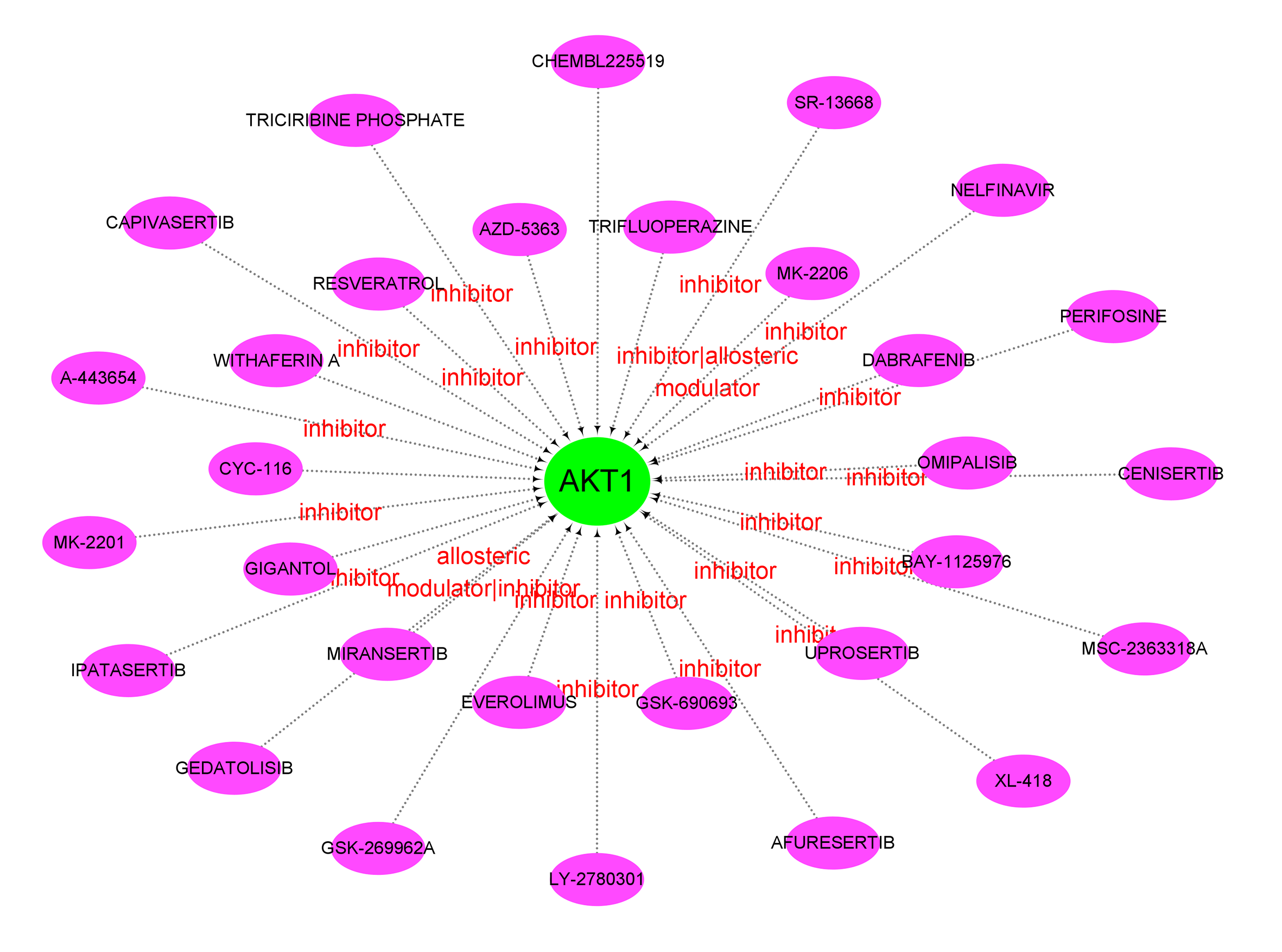

Supplement: Supplementary file 5 [file Data_Sheet_5.ZIP › Fig 11/Fig 11-2.tif]

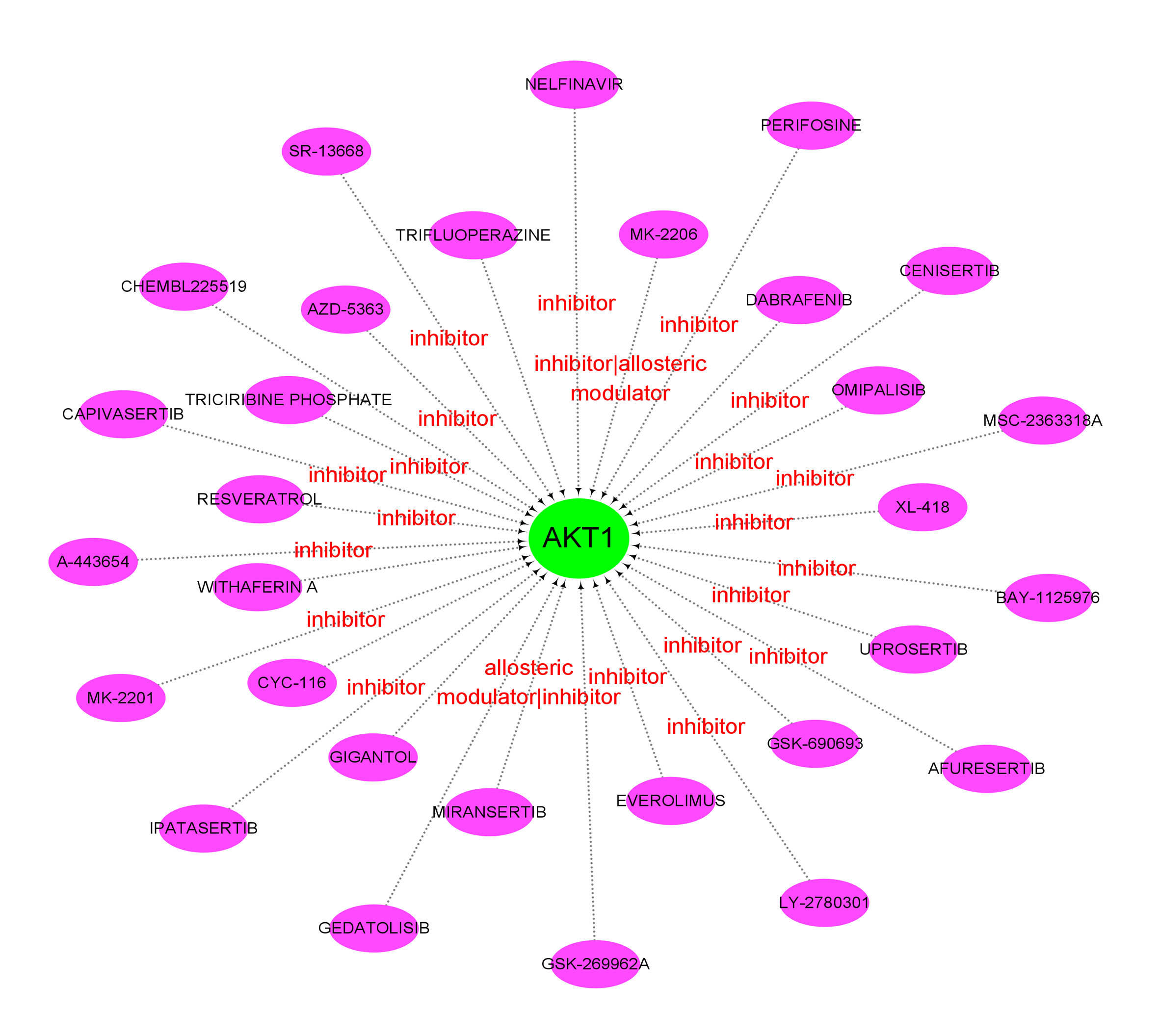

Supplement: Supplementary file 5 [file Data_Sheet_5.ZIP › Fig 11/╨▐╕─═╝9.tif]

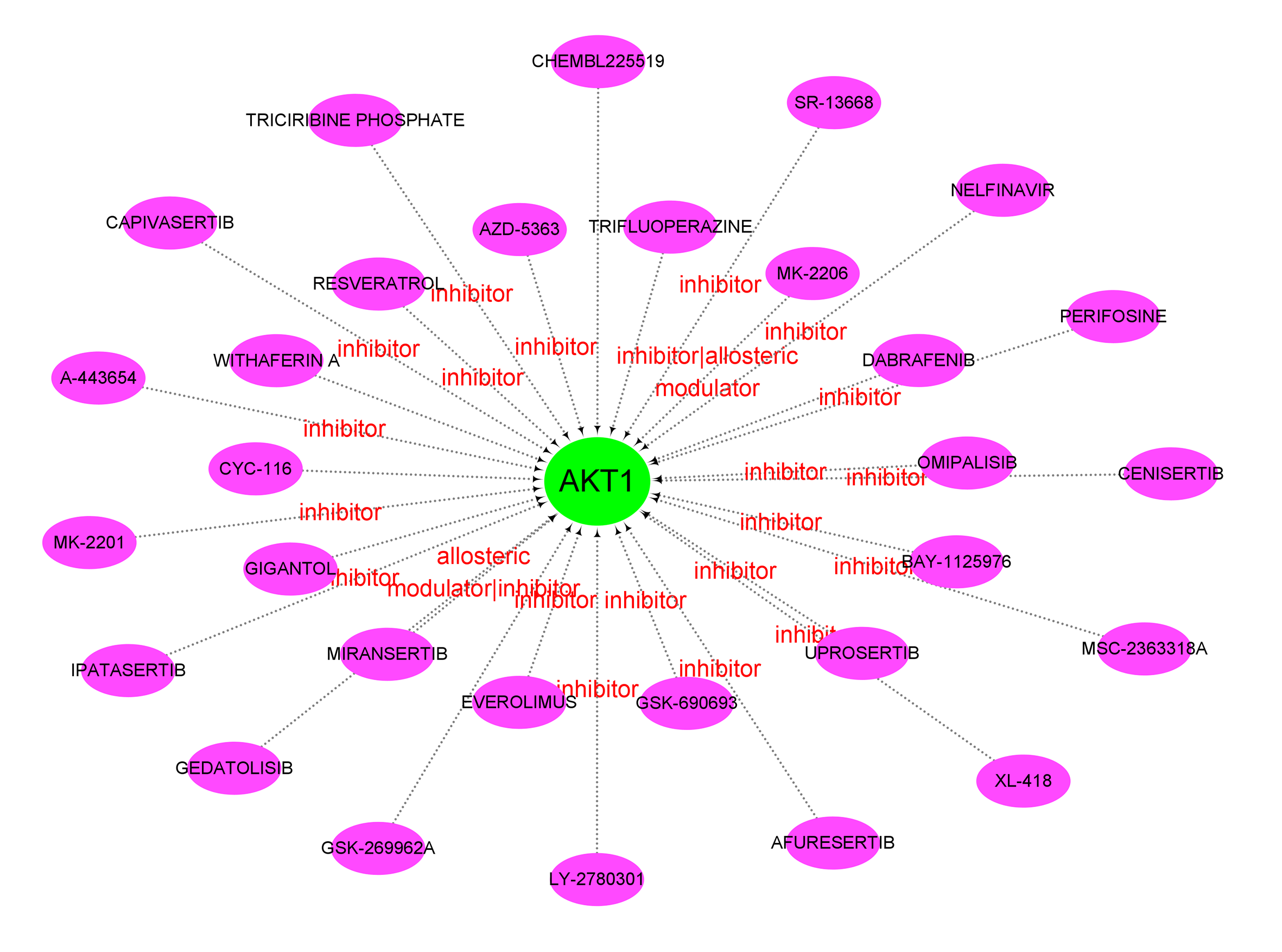

Supplement: Supplementary file 5 [file Data_Sheet_5.ZIP › Fig 11/╨▐╕─═╝9-2.tif]

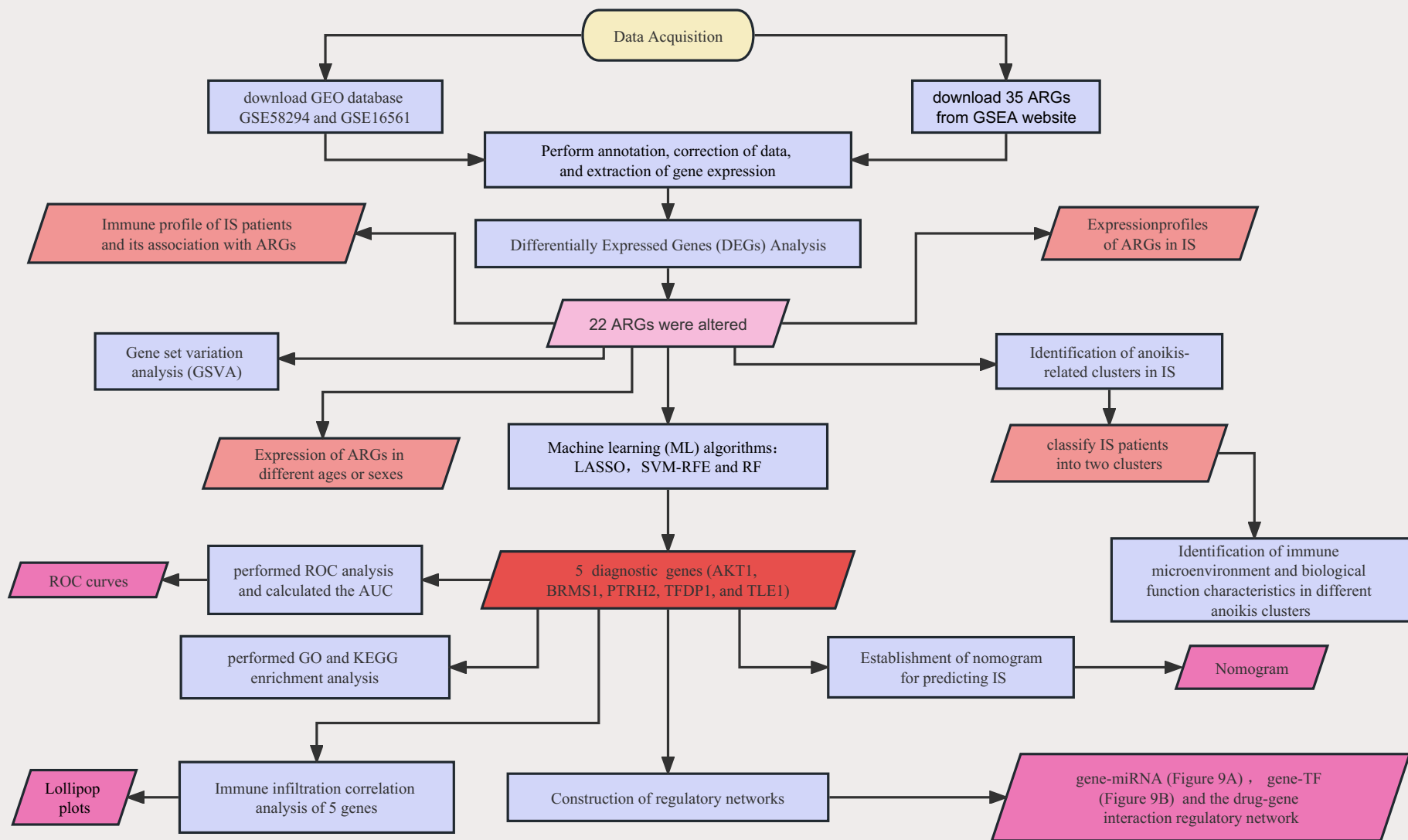

Supplement: Supplementary file 6 [file Data_Sheet_6.ZIP › Fig 1/Fig 1.pdf]

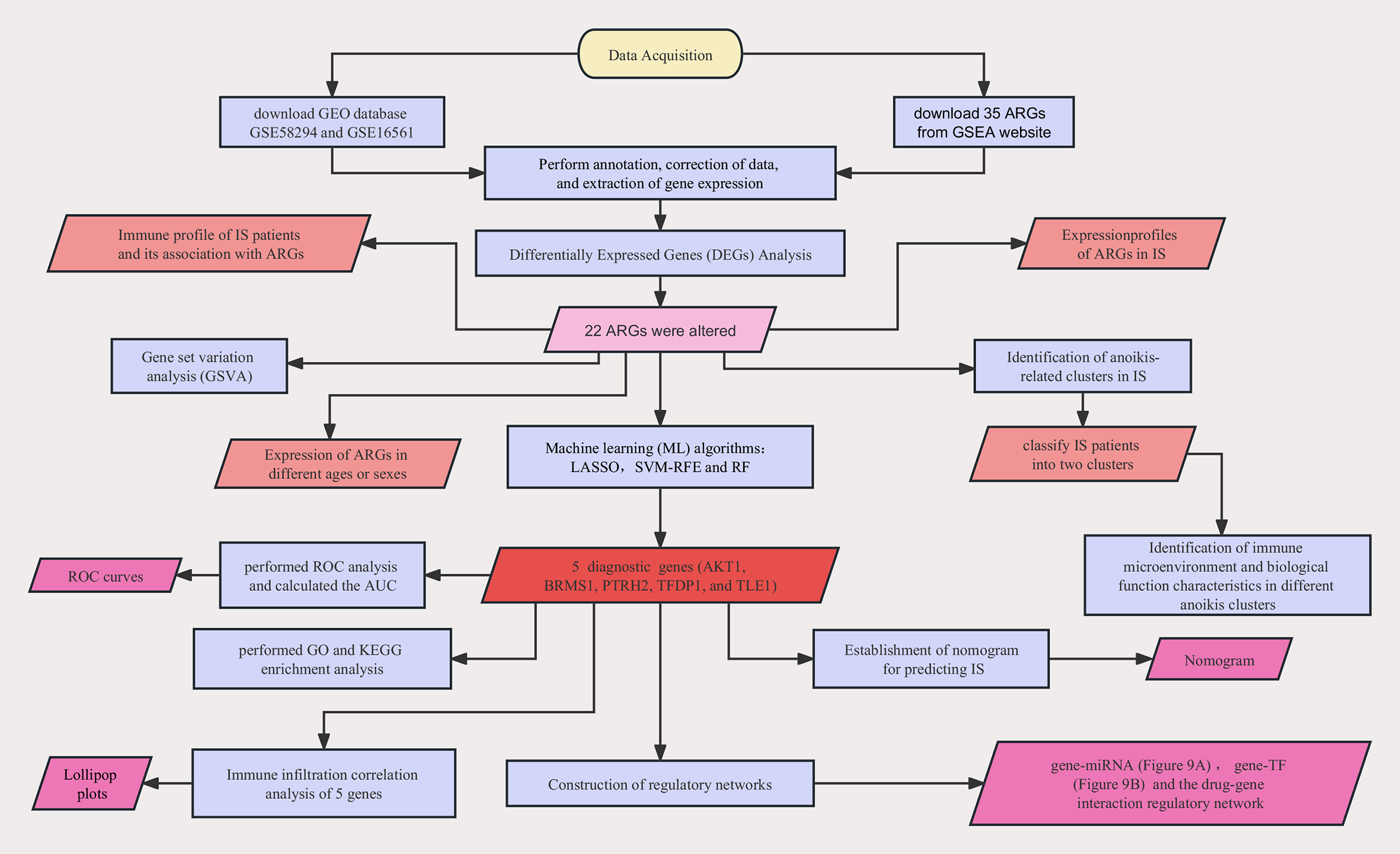

Supplement: Supplementary file 6 [file Data_Sheet_6.ZIP › Fig 1/Fig 1.tif]

Type Control Treat

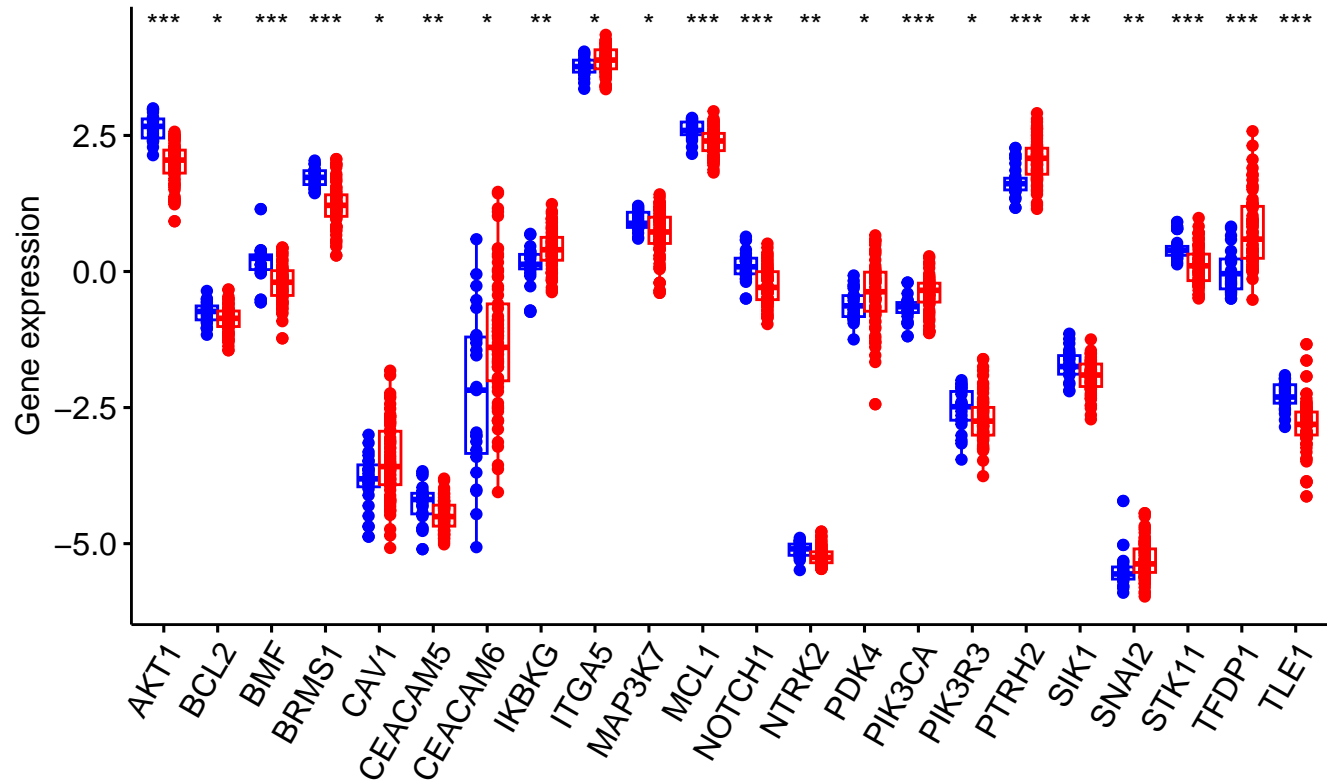

Supplement: Supplementary file 6 [file Data_Sheet_6.ZIP › Fig 2/═╝2/07.diff-boxplot.pdf]

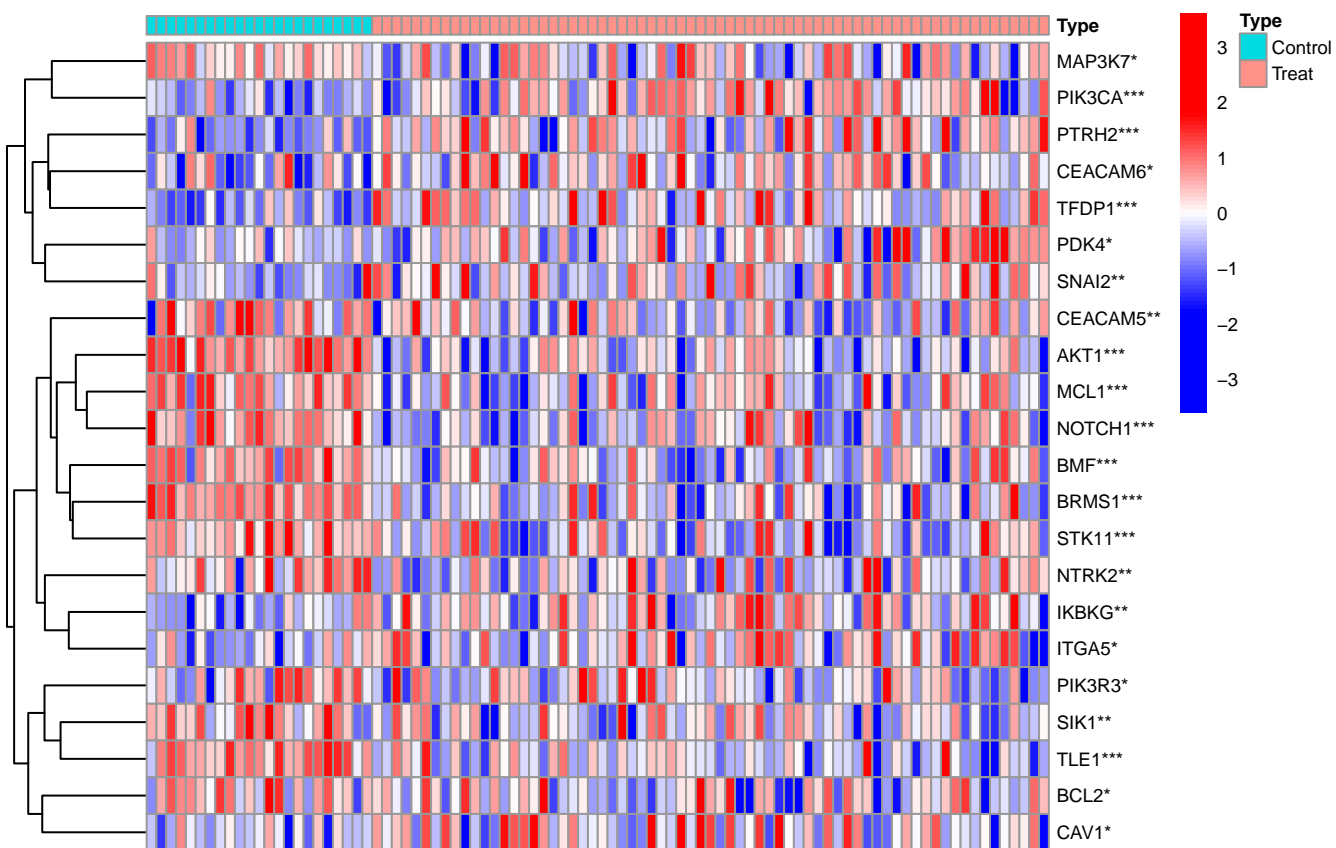

Supplement: Supplementary file 6 [file Data_Sheet_6.ZIP › Fig 2/═╝2/07.diff-heatmap.pdf]

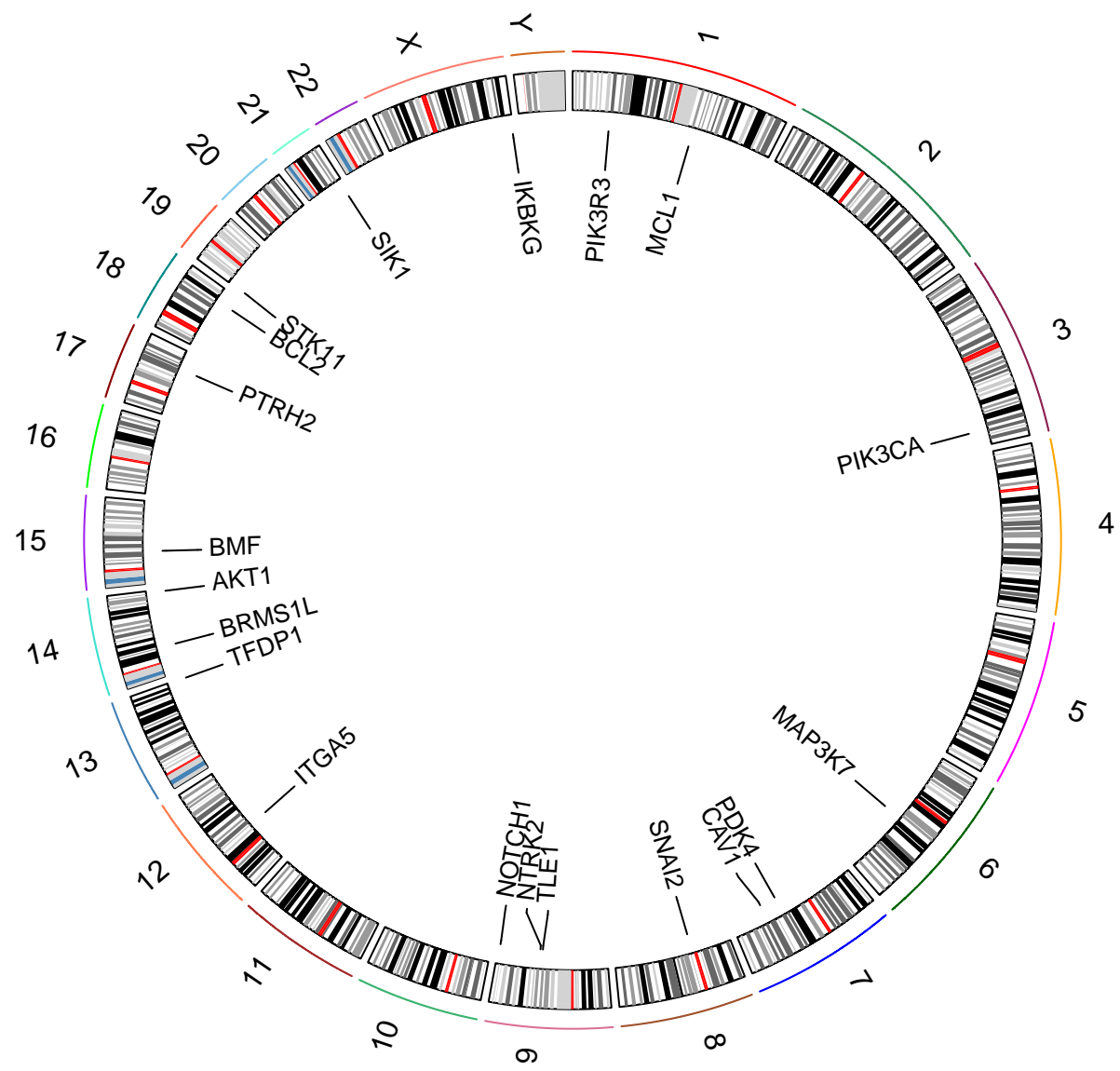

Supplement: Supplementary file 6 [file Data_Sheet_6.ZIP › Fig 2/═╝2/09.Rcircos-╗∙╥≥╚╛╔1⁄2╠σ╚a═╝.pdf]

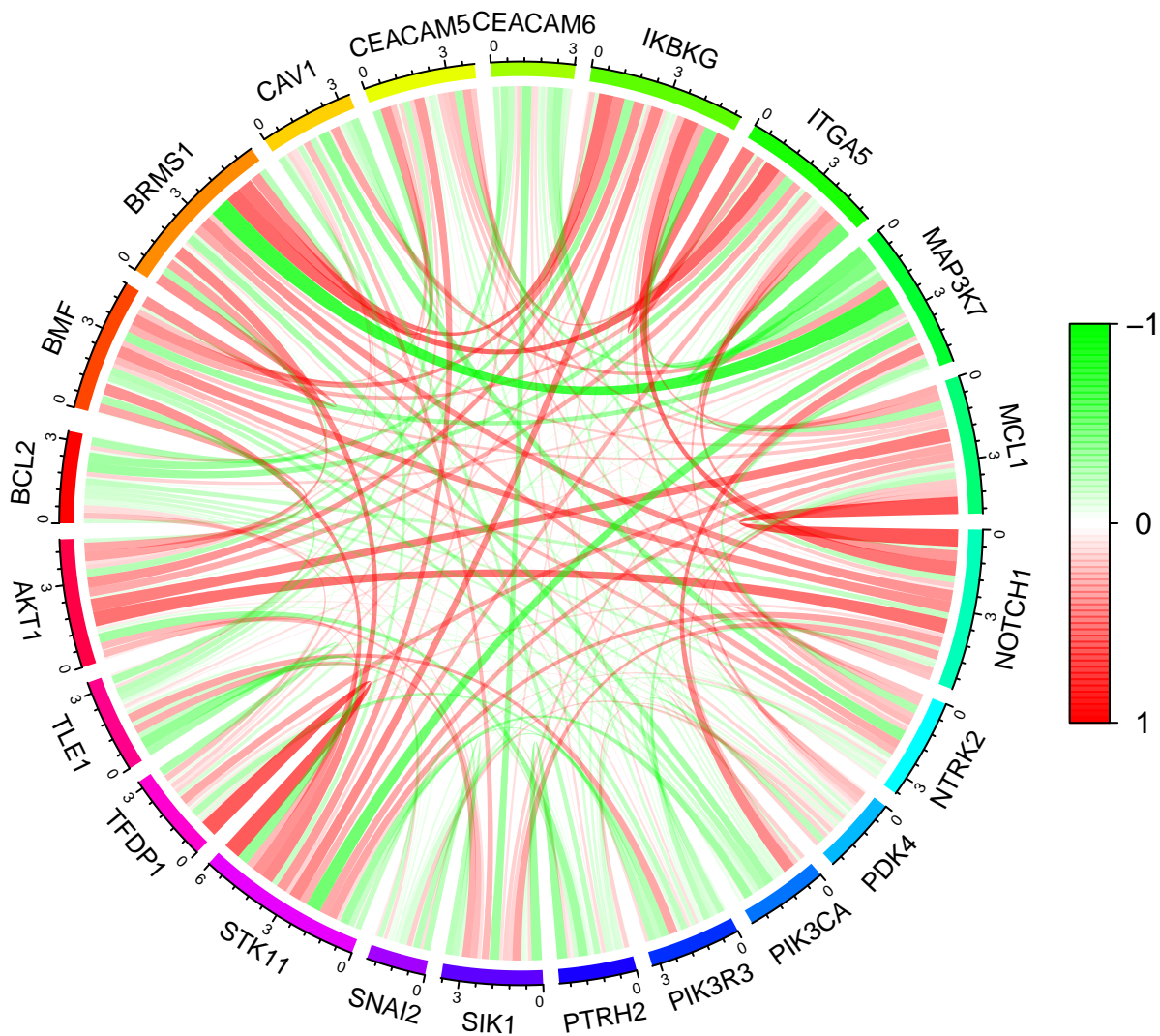

Supplement: Supplementary file 6 [file Data_Sheet_6.ZIP › Fig 2/═╝2/10.circos.pdf]

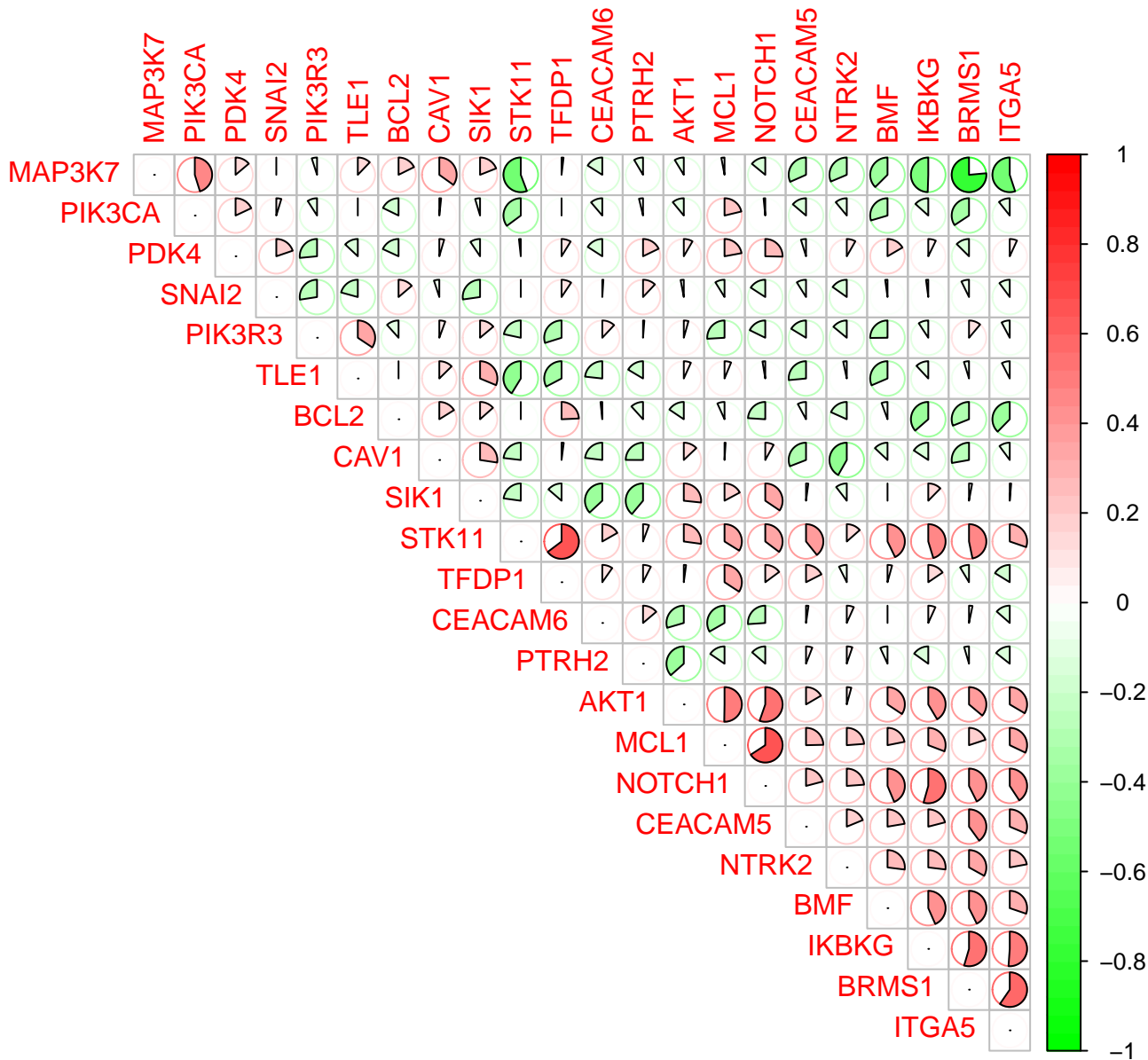

Supplement: Supplementary file 6 [file Data_Sheet_6.ZIP › Fig 2/═╝2/10.corrplot.pdf]

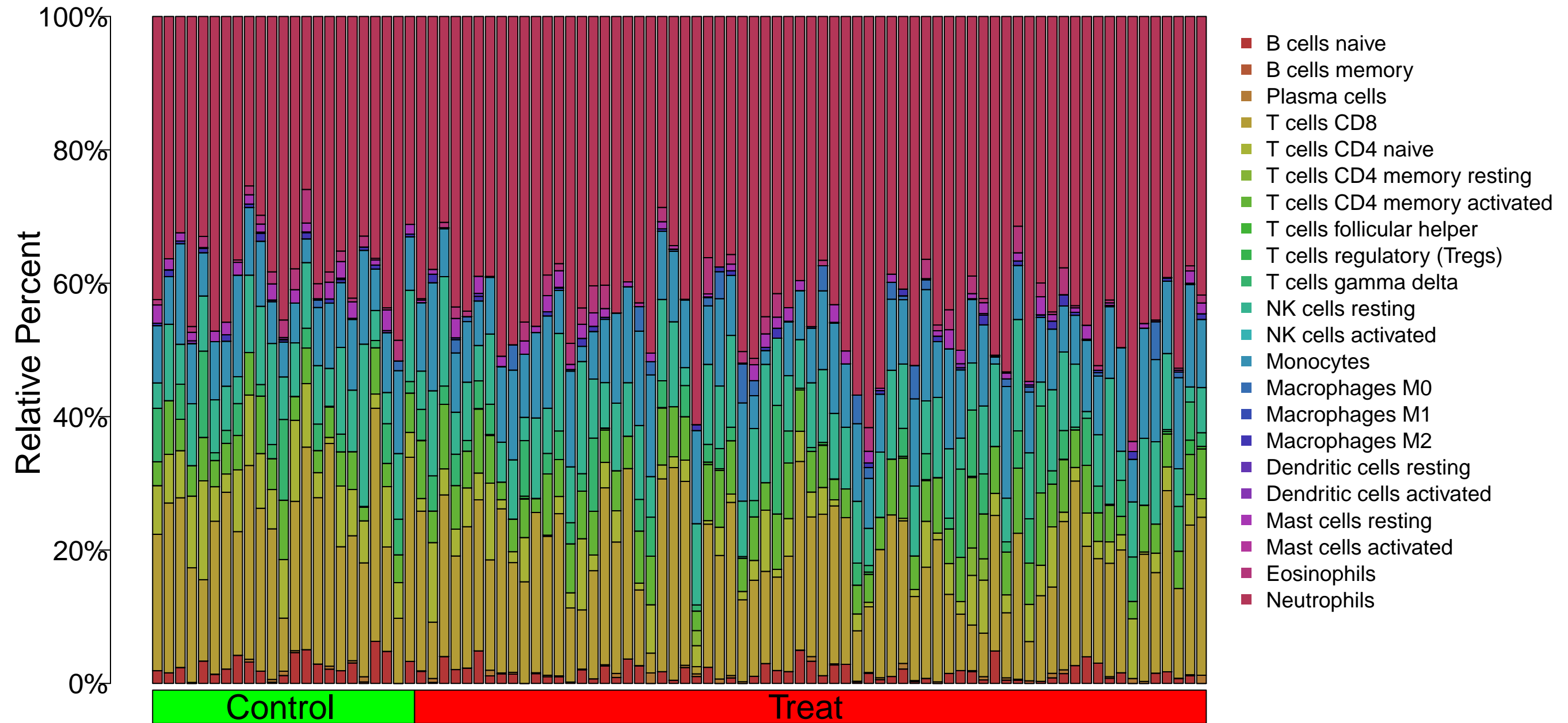

Supplement: Supplementary file 6 [file Data_Sheet_6.ZIP › Fig 3/═╝3/12.barplot.pdf]

Type 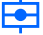 Control 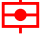 Treat

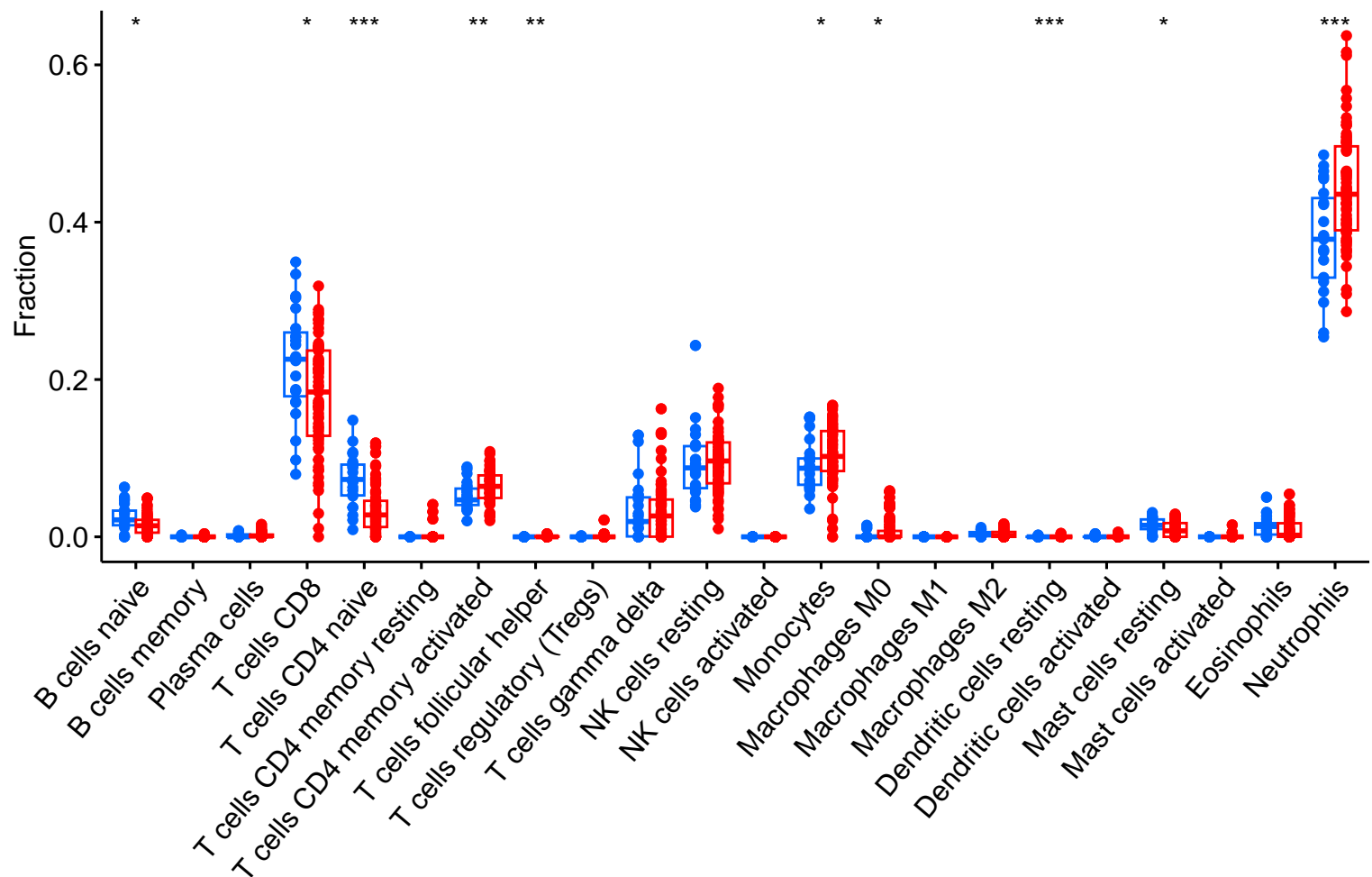

Supplement: Supplementary file 6 [file Data_Sheet_6.ZIP › Fig 3/═╝3/12.immune.diff.pdf]

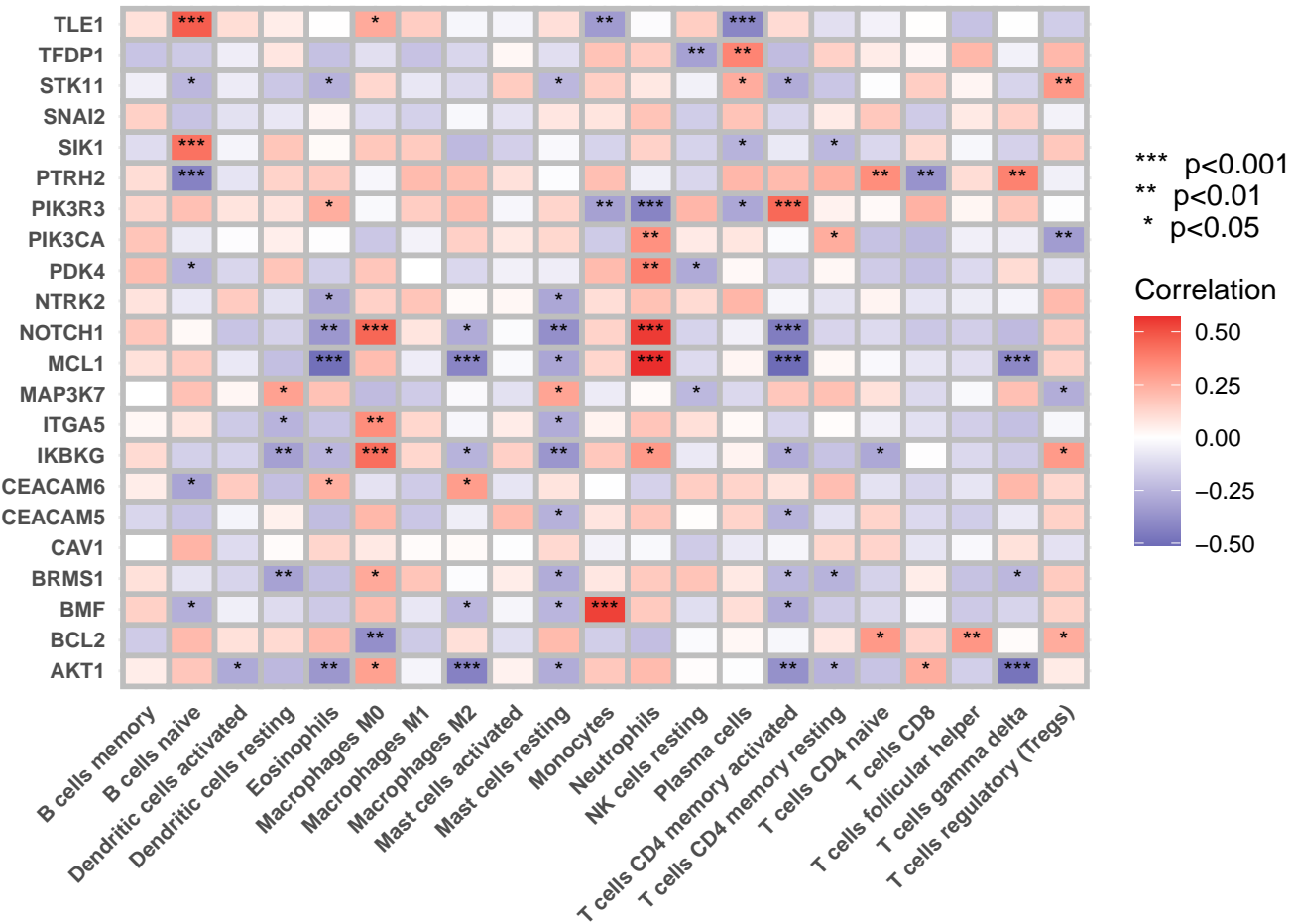

Supplement: Supplementary file 6 [file Data_Sheet_6.ZIP › Fig 3/═╝3/13.cor.pdf]

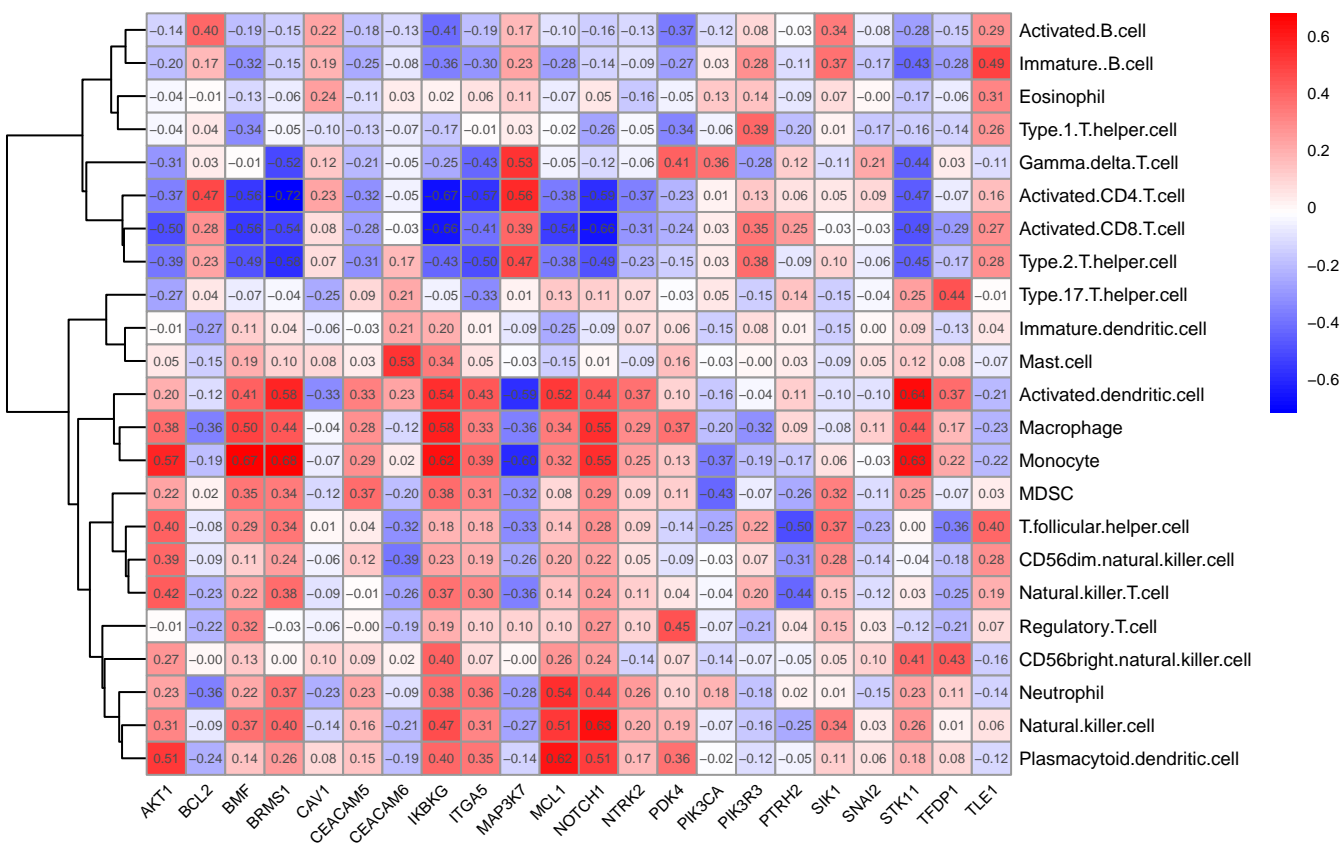

Supplement: Supplementary file 6 [file Data_Sheet_6.ZIP › Fig 3/═╝3/13.heatmap.pdf]

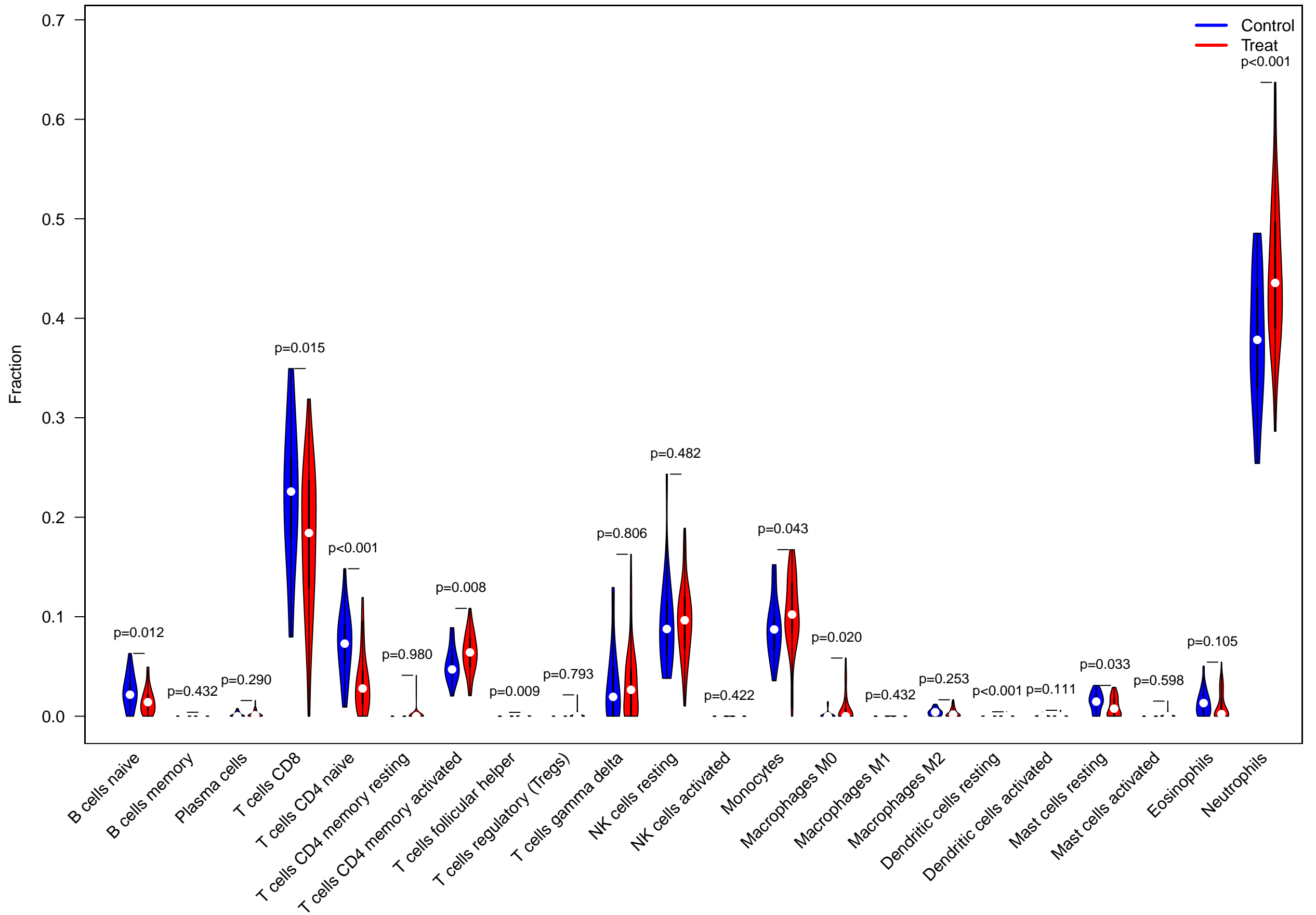

Supplement: Supplementary file 6 [file Data_Sheet_6.ZIP › Fig 3/═╝3/26.vioplot.pdf]

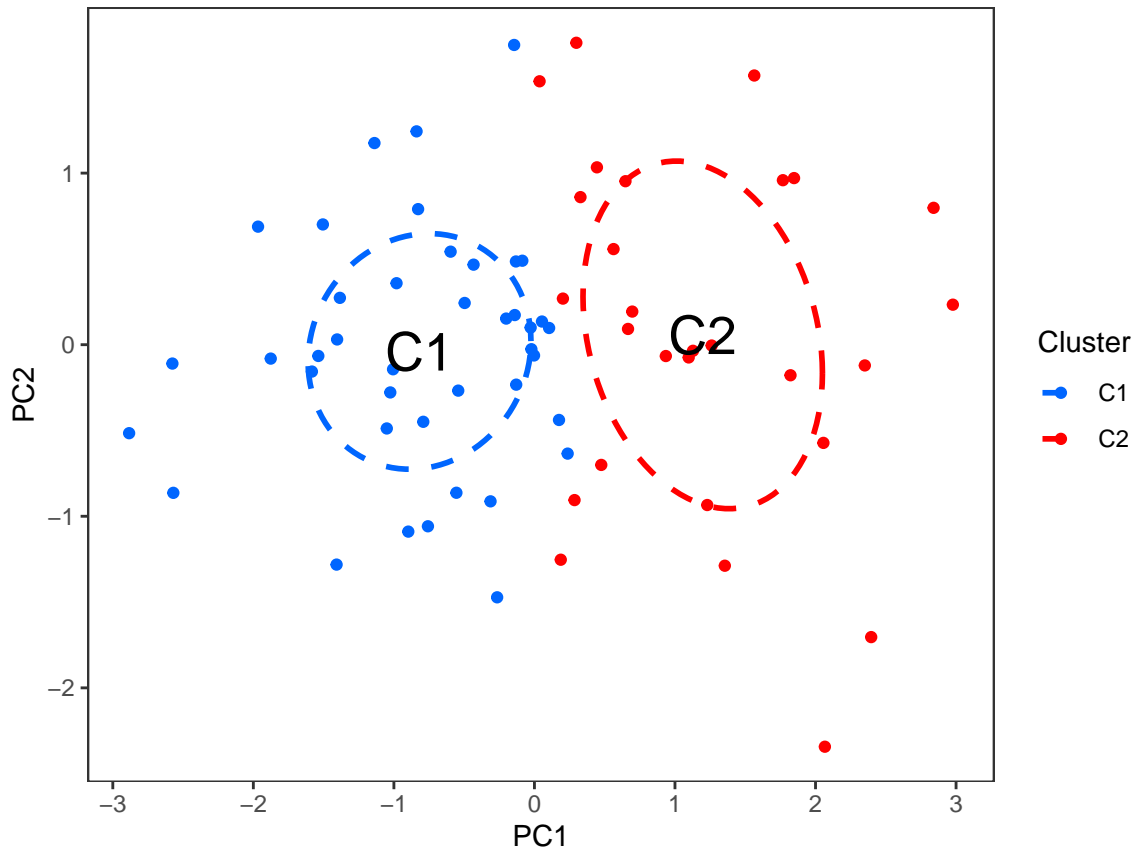

Supplement: Supplementary file 6 [file Data_Sheet_6.ZIP › Fig 4/═╝4/16.PCA.pdf]

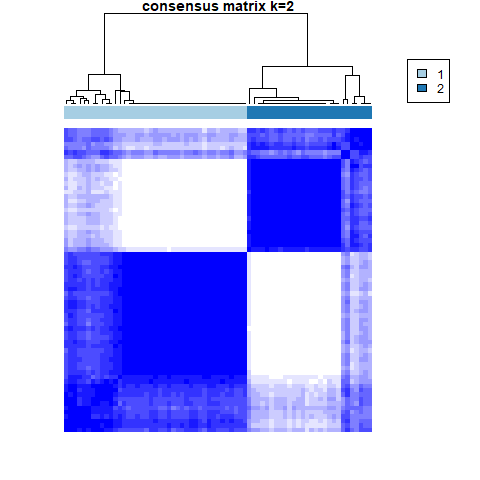

Supplement: Supplementary file 6 [file Data_Sheet_6.ZIP › Fig 4/═╝4/consensus002.png]

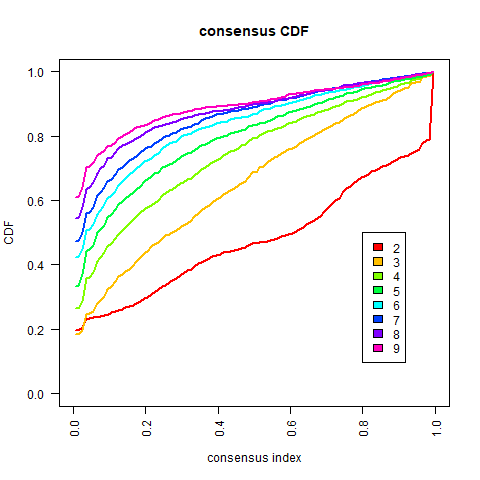

Supplement: Supplementary file 6 [file Data_Sheet_6.ZIP › Fig 4/═╝4/consensus010.png]

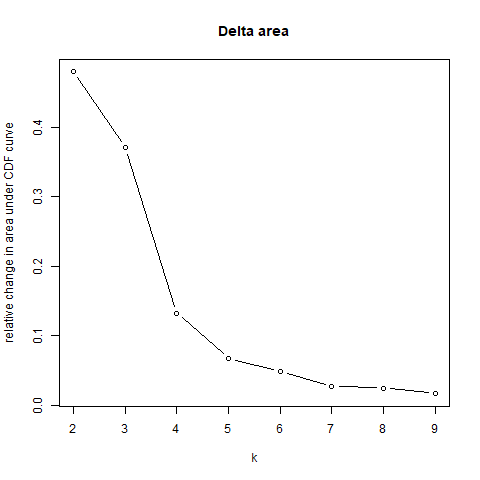

Supplement: Supplementary file 6 [file Data_Sheet_6.ZIP › Fig 4/═╝4/consensus011.png]

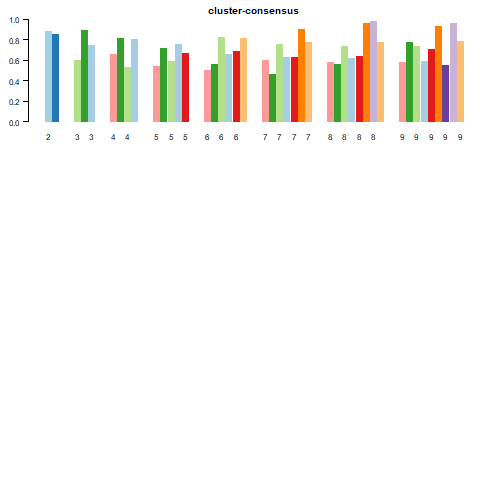

Supplement: Supplementary file 6 [file Data_Sheet_6.ZIP › Fig 4/═╝4/icl004.png]

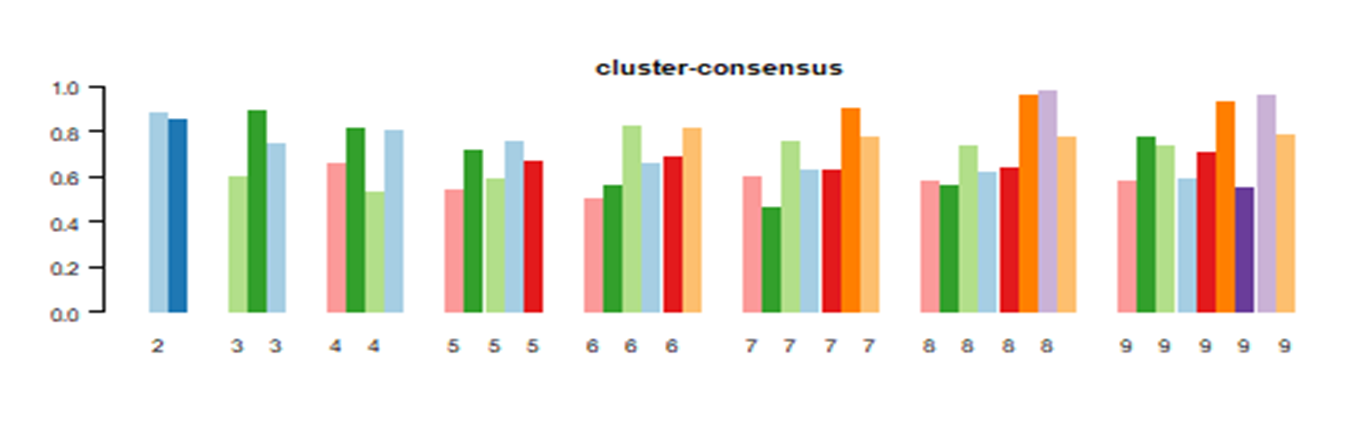

Supplement: Supplementary file 6 [file Data_Sheet_6.ZIP › Fig 4/═╝4/╜╪═╝.PDF.png]

# AKT1

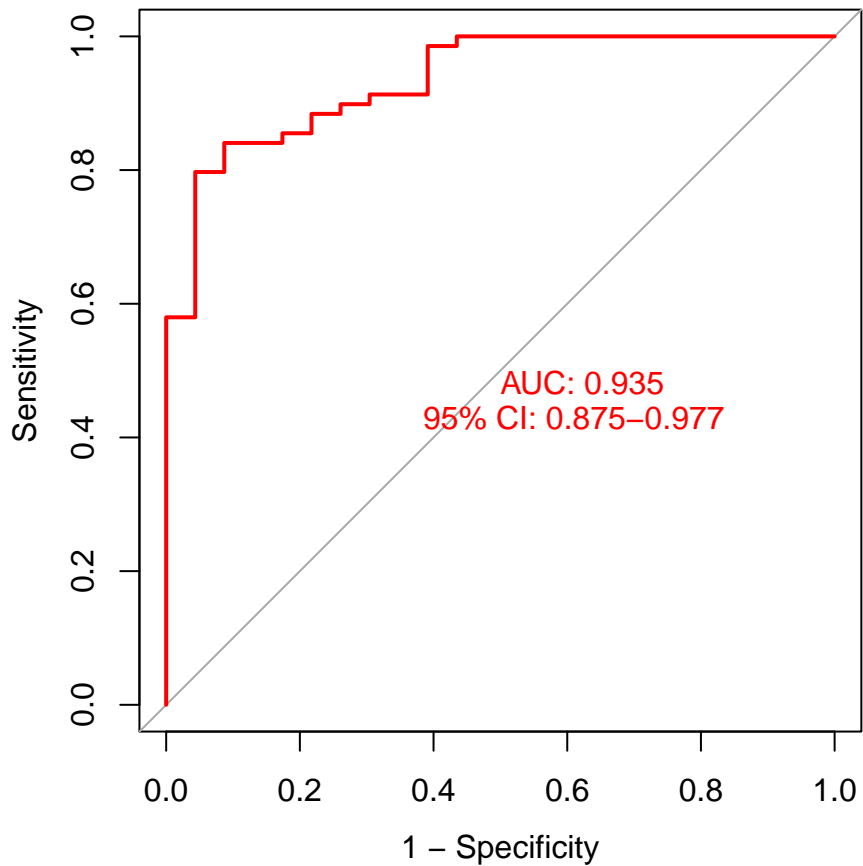

Supplement: Supplementary file 7 [file Data_Sheet_7.ZIP › Fig 7/═╝7/╤╡┴╖╫Θ-ROC/ROC.AKT1.pdf]

# BRMS1

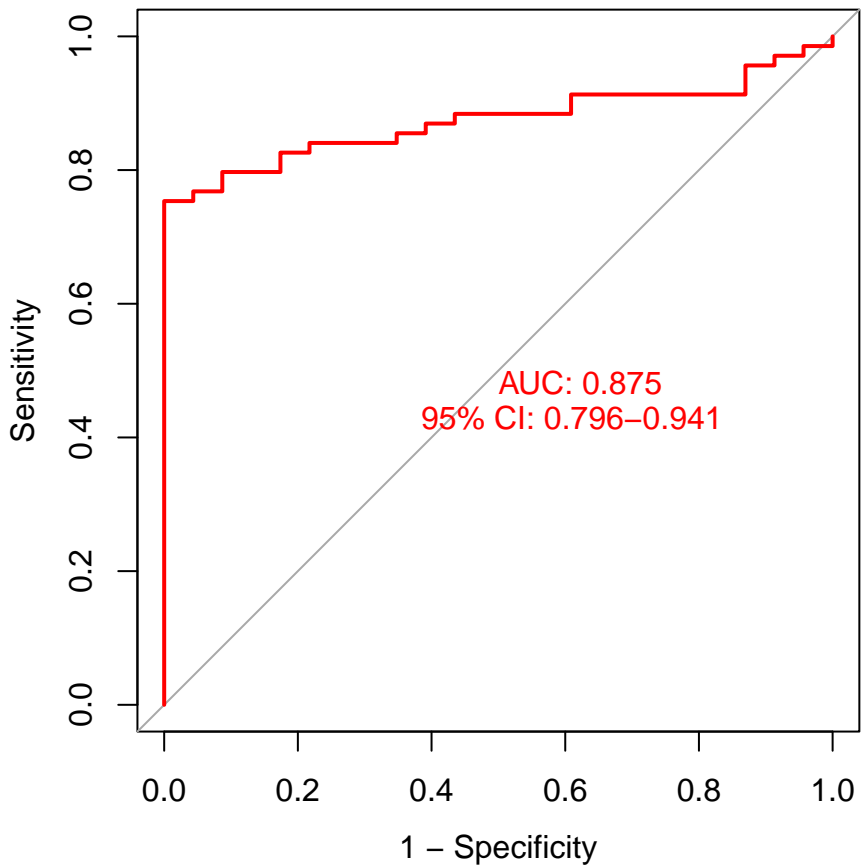

Supplement: Supplementary file 7 [file Data_Sheet_7.ZIP › Fig 7/═╝7/╤╡┴╖╫Θ-ROC/ROC.BRMS1.pdf]

# MAP3K7

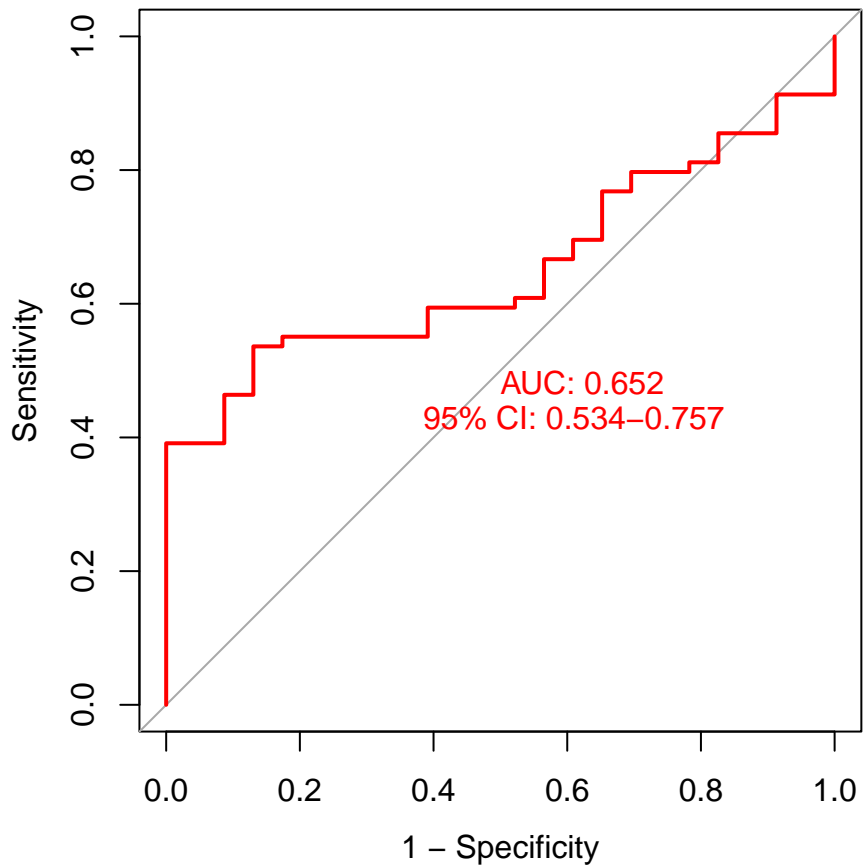

Supplement: Supplementary file 7 [file Data_Sheet_7.ZIP › Fig 7/═╝7/╤╡┴╖╫Θ-ROC/ROC.MAP3K7.pdf]

# NOTCH1

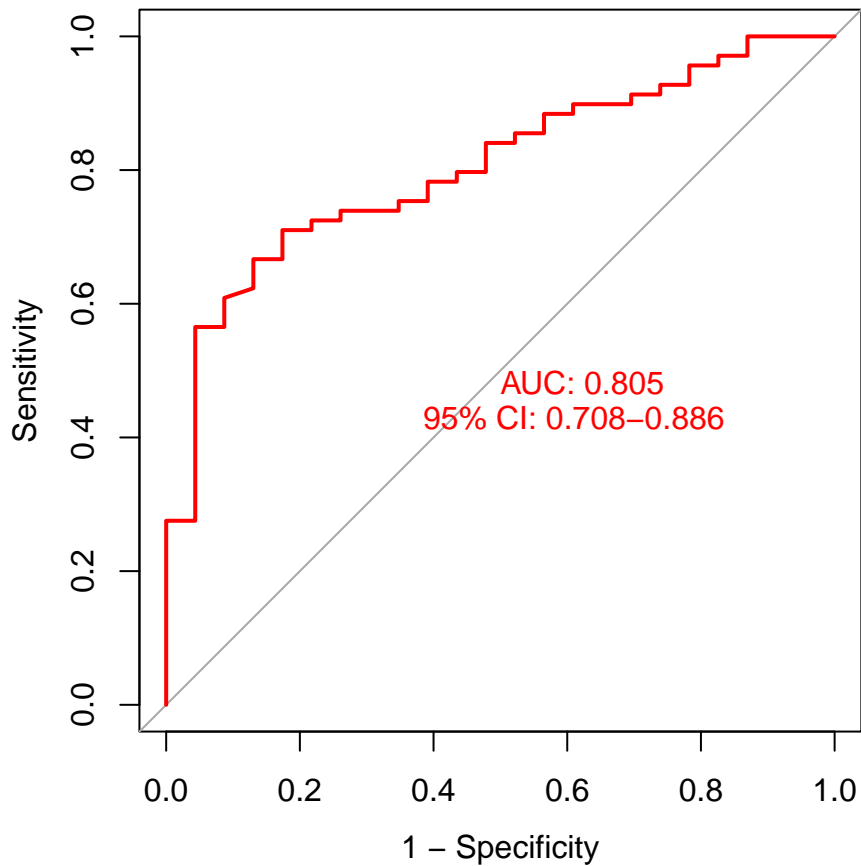

Supplement: Supplementary file 7 [file Data_Sheet_7.ZIP › Fig 7/═╝7/╤╡┴╖╫Θ-ROC/ROC.NOTCH1.pdf]

## PTRH2

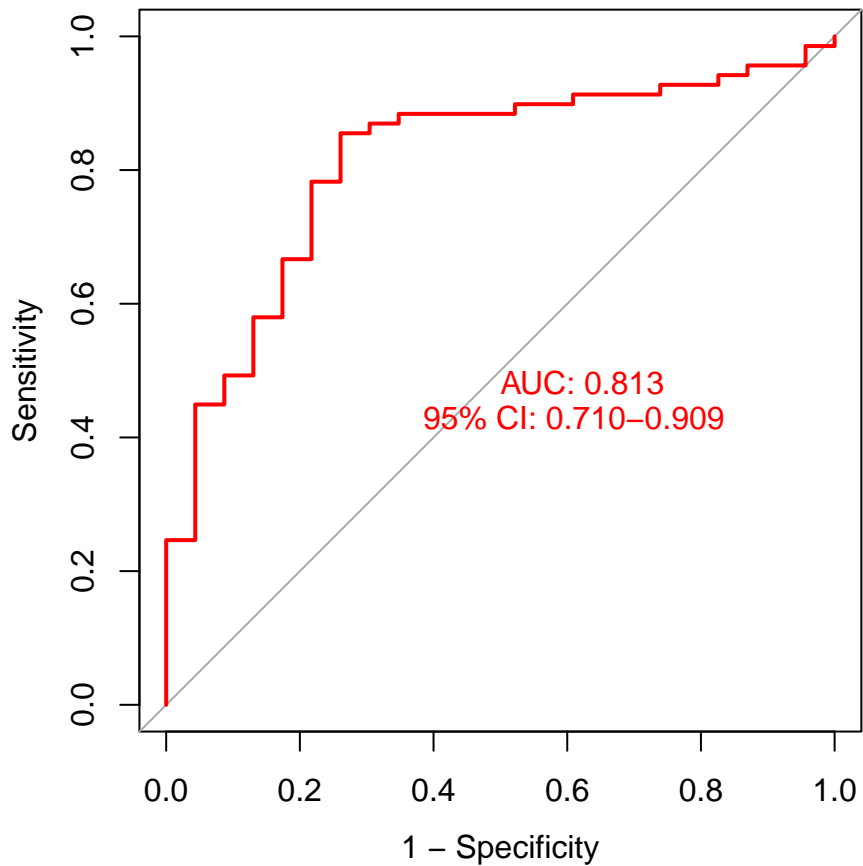

Supplement: Supplementary file 7 [file Data_Sheet_7.ZIP › Fig 7/═╝7/╤╡┴╖╫Θ-ROC/ROC.PTRH2.pdf]

# STK11

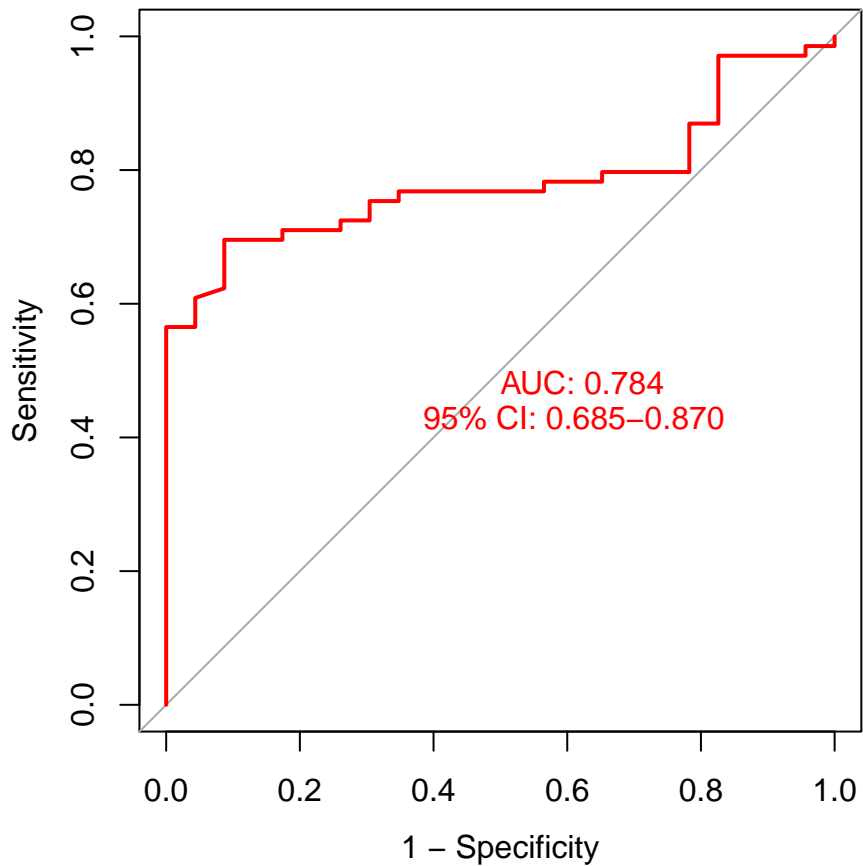

Supplement: Supplementary file 7 [file Data_Sheet_7.ZIP › Fig 7/═╝7/╤╡┴╖╫Θ-ROC/ROC.STK11.pdf]

# TFDP1

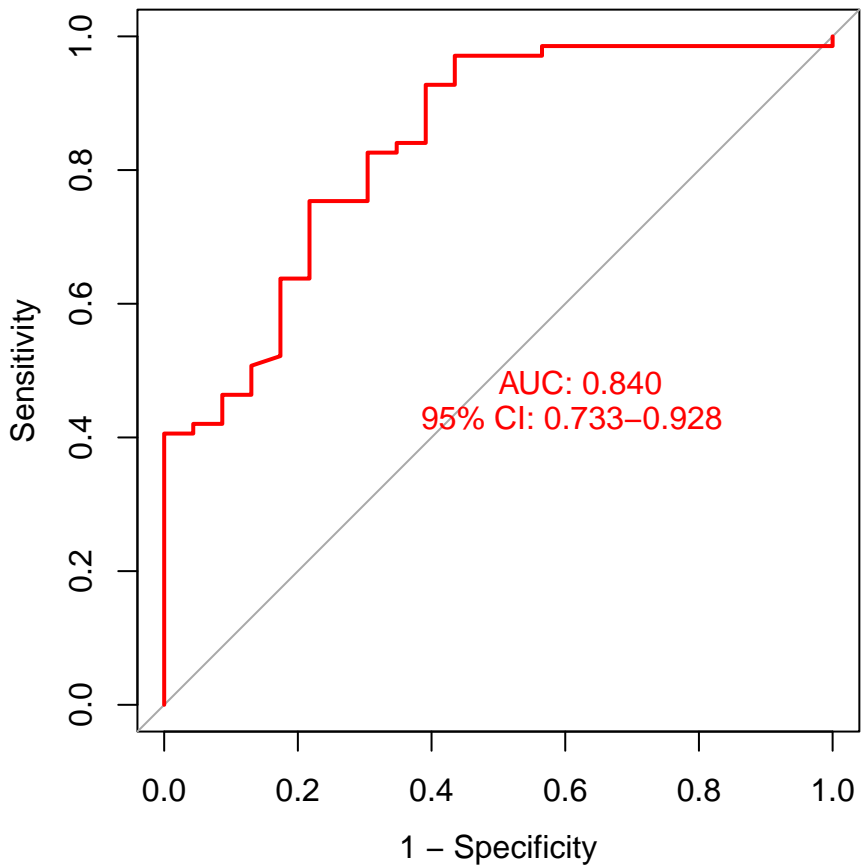

Supplement: Supplementary file 7 [file Data_Sheet_7.ZIP › Fig 7/═╝7/╤╡┴╖╫Θ-ROC/ROC.TFDP1.pdf]

# TLE1

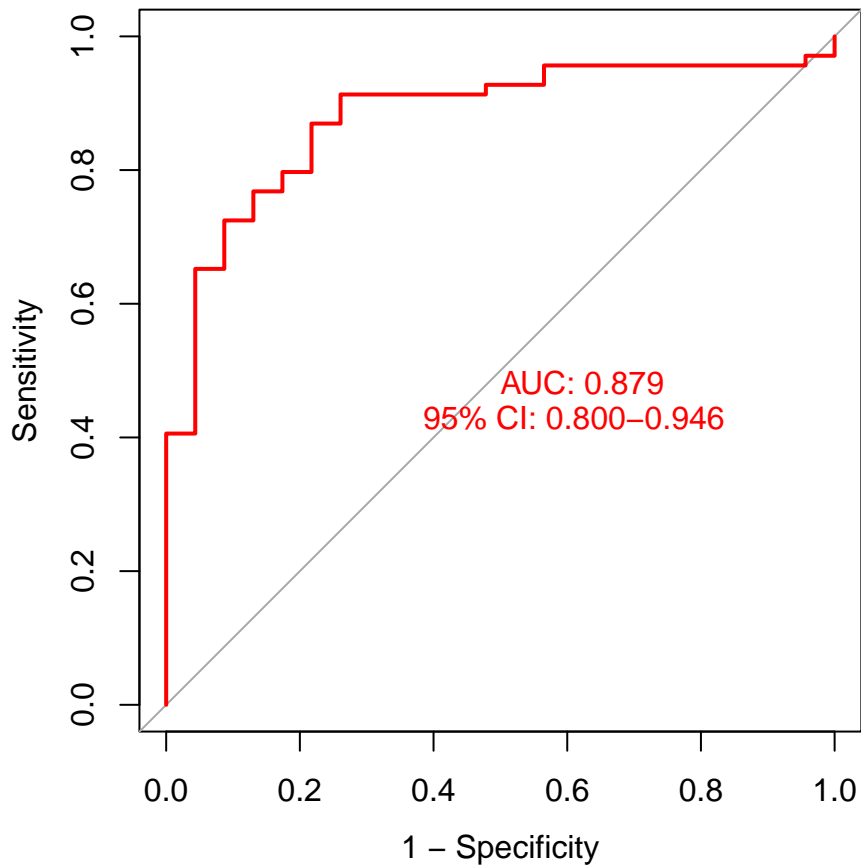

Supplement: Supplementary file 7 [file Data_Sheet_7.ZIP › Fig 7/═╝7/╤╡┴╖╫Θ-ROC/ROC.TLE1.pdf]

# AKT1

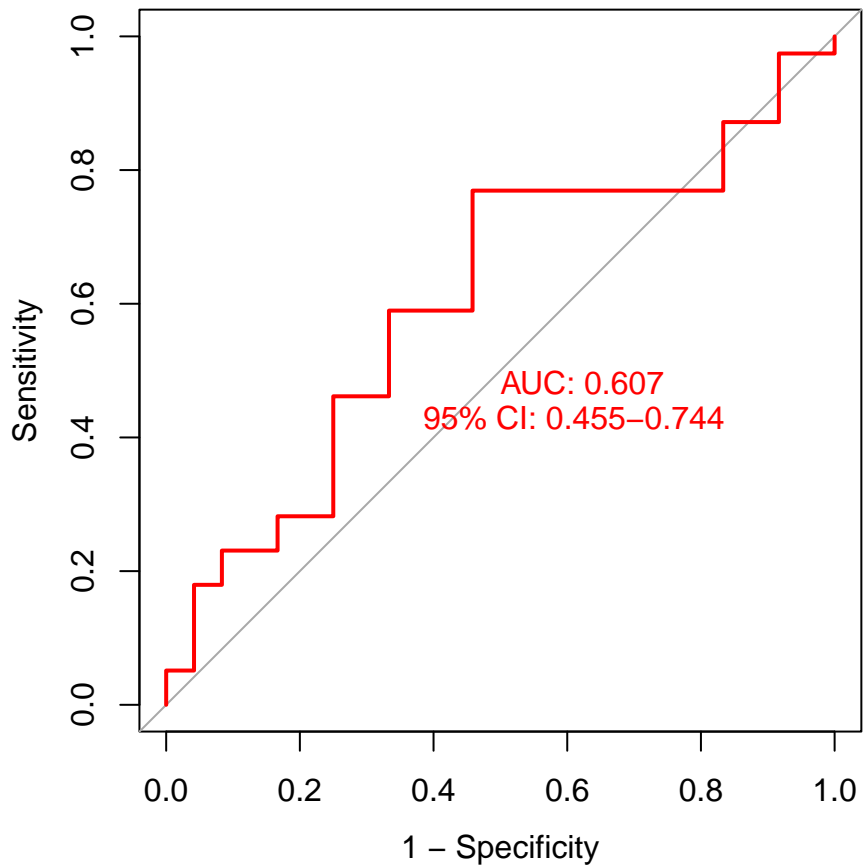

Supplement: Supplementary file 7 [file Data_Sheet_7.ZIP › Fig 7/═╝7/╤Θ╓ñ╫Θ-ROC/ROC.AKT1.pdf]

# BRMS1

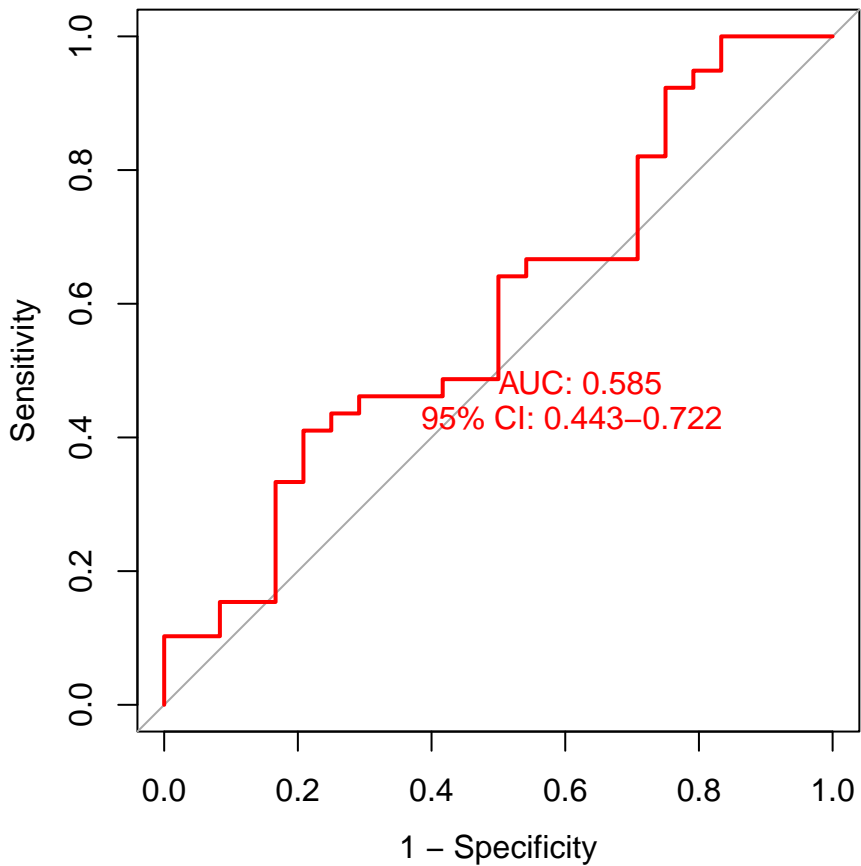

Supplement: Supplementary file 7 [file Data_Sheet_7.ZIP › Fig 7/═╝7/╤Θ╓ñ╫Θ-ROC/ROC.BRMS1.pdf]

# MAP3K7

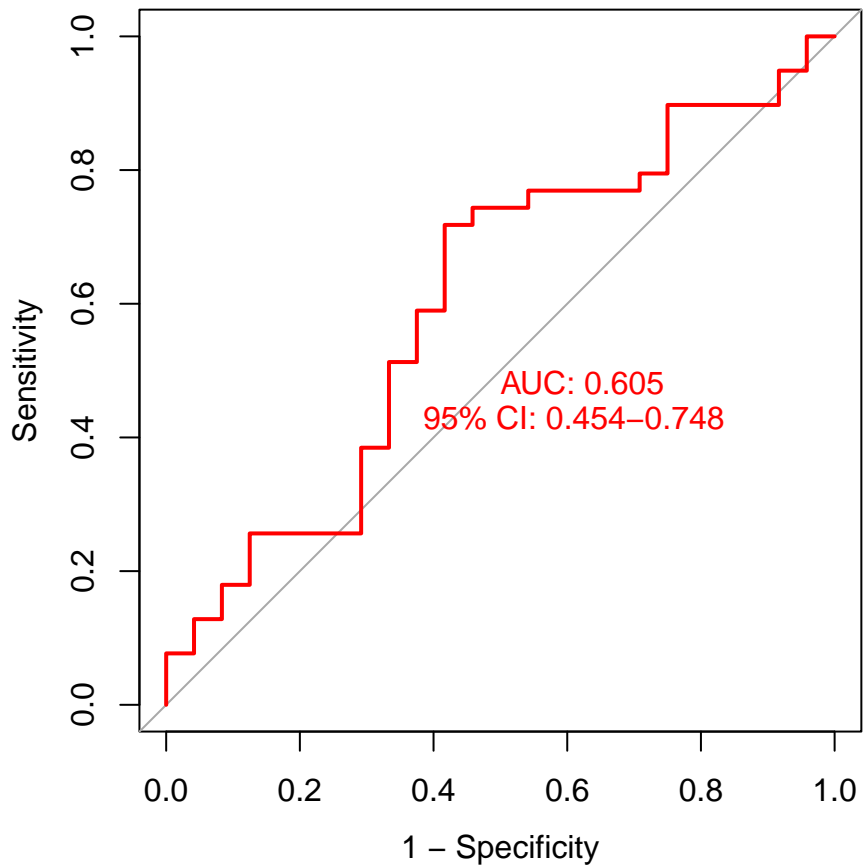

Supplement: Supplementary file 7 [file Data_Sheet_7.ZIP › Fig 7/═╝7/╤Θ╓ñ╫Θ-ROC/ROC.MAP3K7.pdf]

# NOTCH1

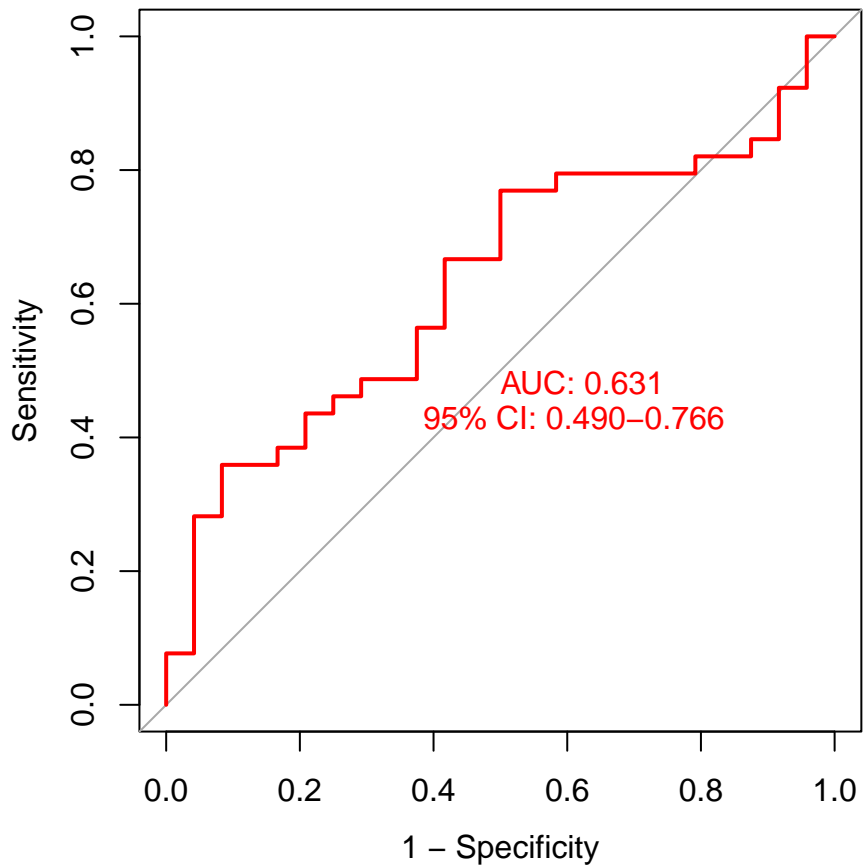

Supplement: Supplementary file 7 [file Data_Sheet_7.ZIP › Fig 7/═╝7/╤Θ╓ñ╫Θ-ROC/ROC.NOTCH1.pdf]

## PTRH2

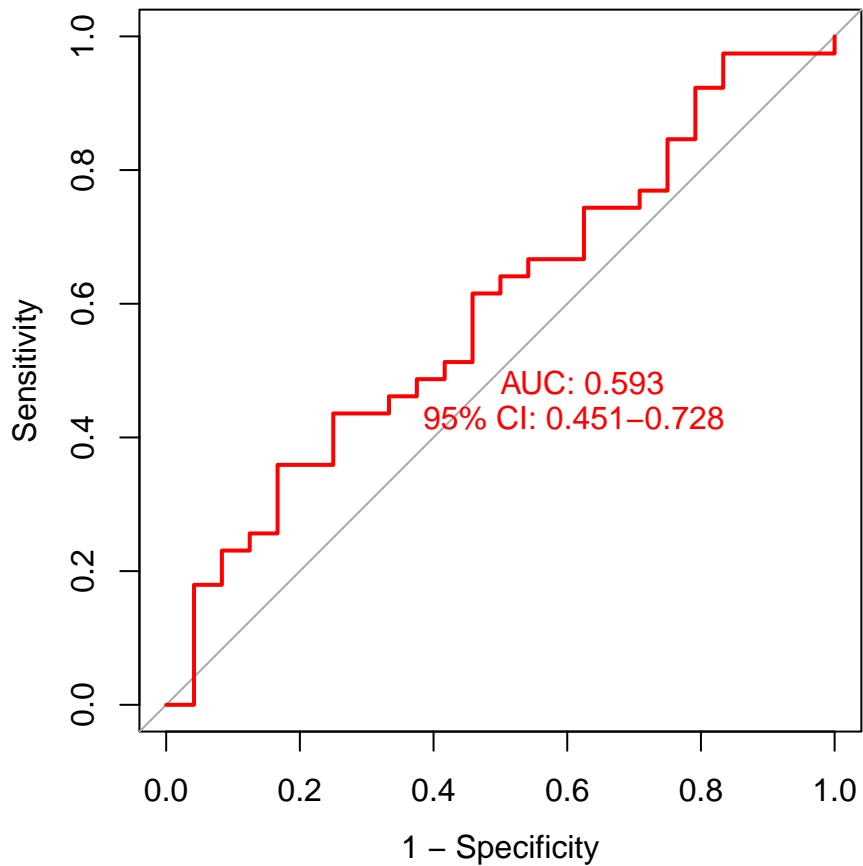

Supplement: Supplementary file 7 [file Data_Sheet_7.ZIP › Fig 7/═╝7/╤Θ╓ñ╫Θ-ROC/ROC.PTRH2.pdf]

# STK11

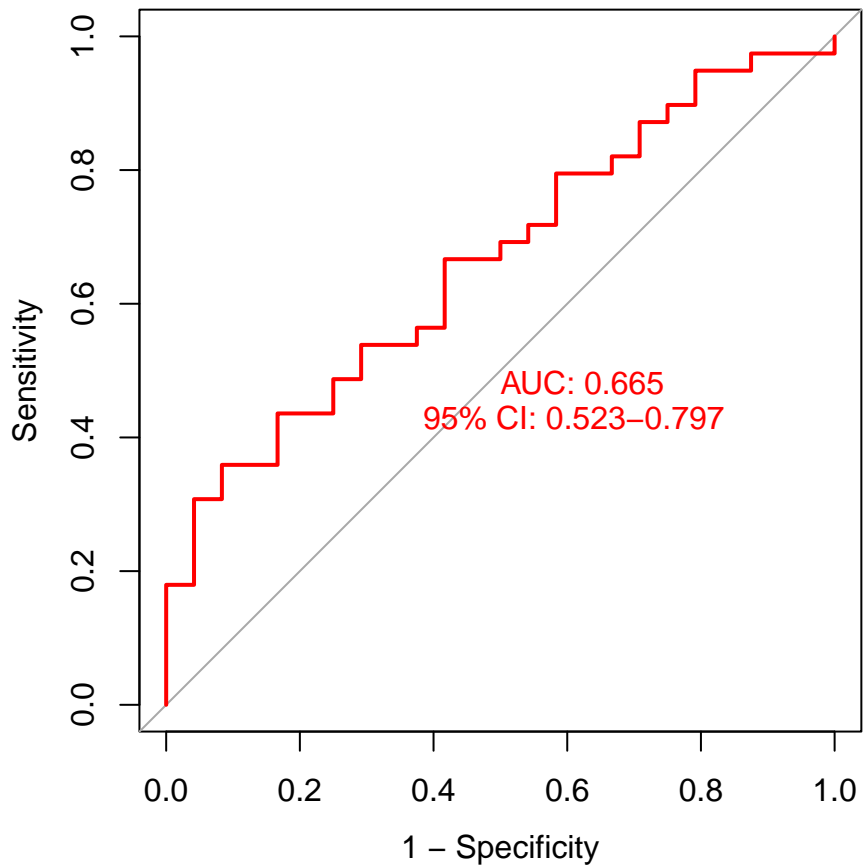

Supplement: Supplementary file 7 [file Data_Sheet_7.ZIP › Fig 7/═╝7/╤Θ╓ñ╫Θ-ROC/ROC.STK11.pdf]

# TFDP1

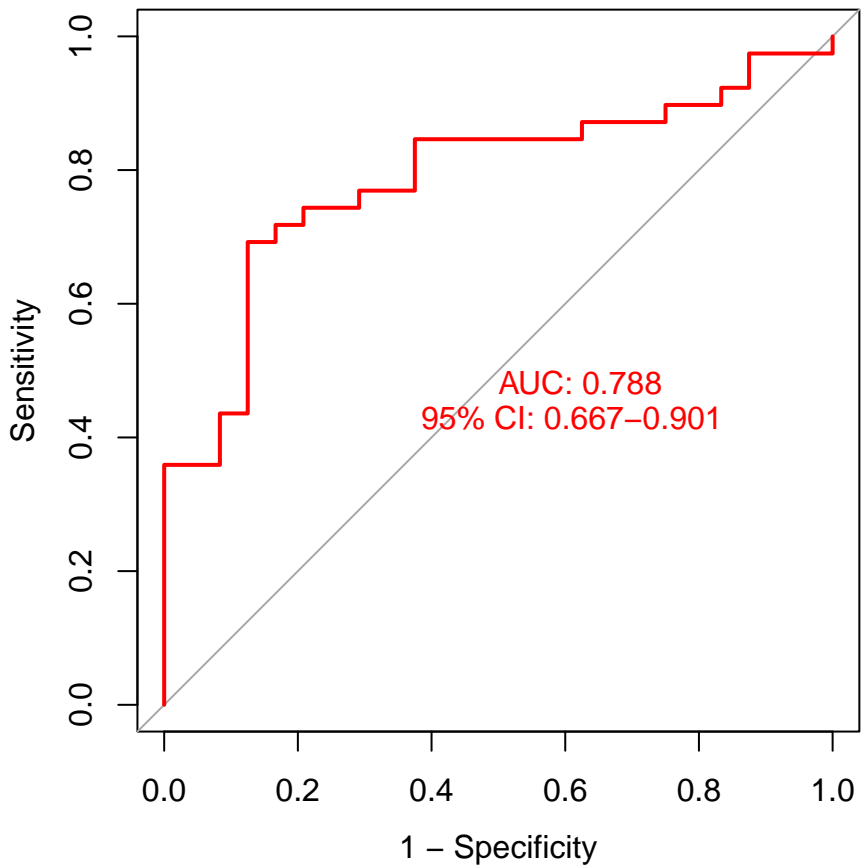

Supplement: Supplementary file 7 [file Data_Sheet_7.ZIP › Fig 7/═╝7/╤Θ╓ñ╫Θ-ROC/ROC.TFDP1.pdf]

# TLE1

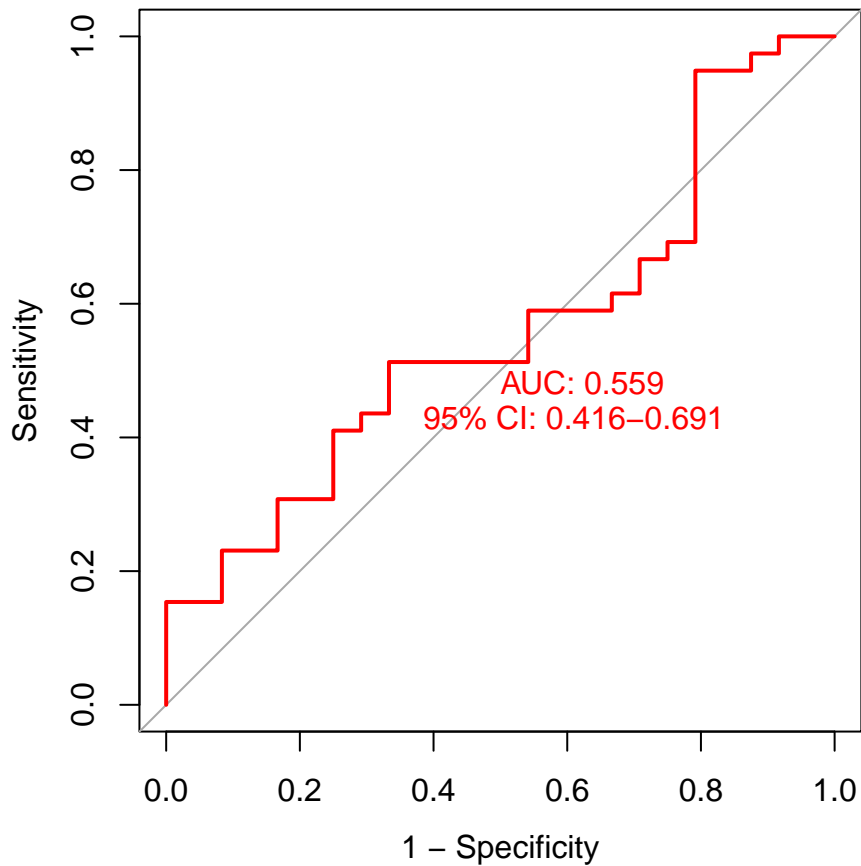

Supplement: Supplementary file 7 [file Data_Sheet_7.ZIP › Fig 7/═╝7/╤Θ╓ñ╫Θ-ROC/ROC.TLE1.pdf]

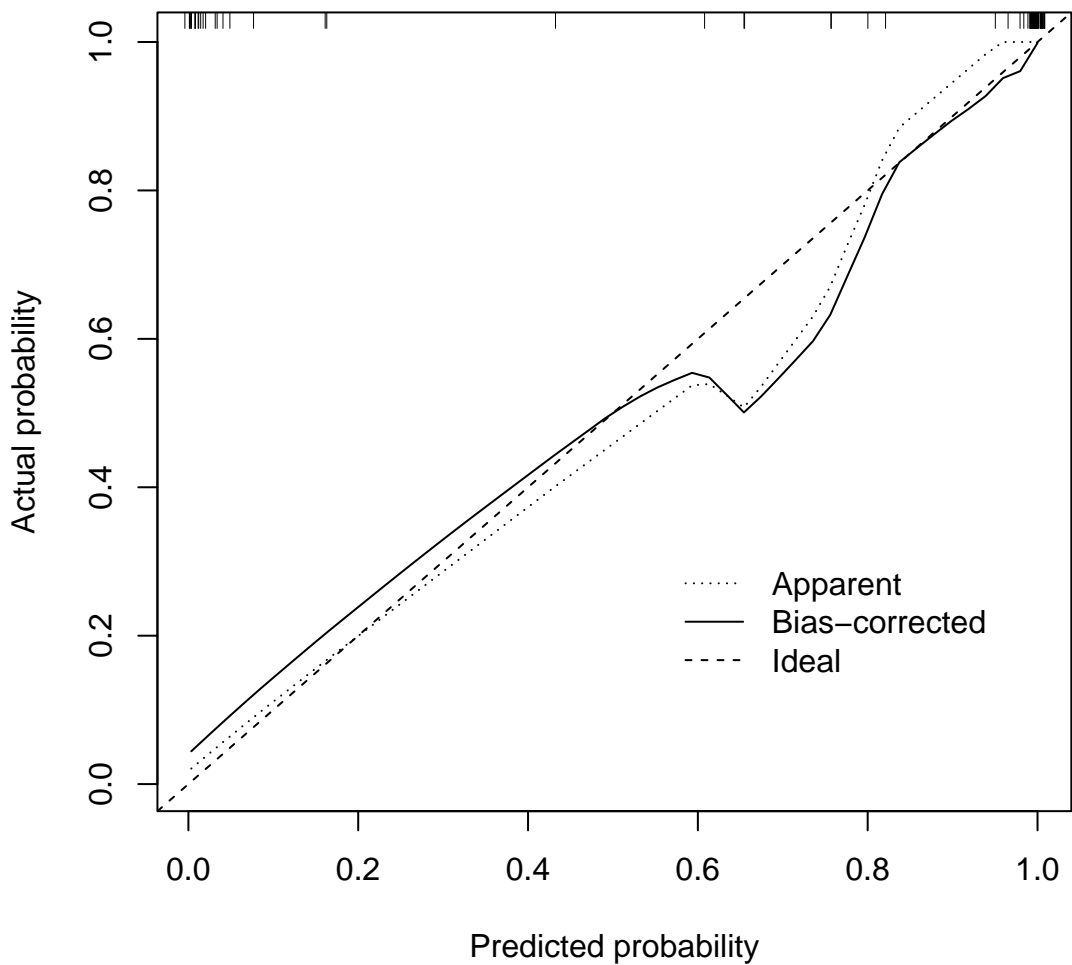

Supplement: Supplementary file 8 [file Data_Sheet_8.ZIP › Fig 8/52.Nomo/Calibration.pdf]

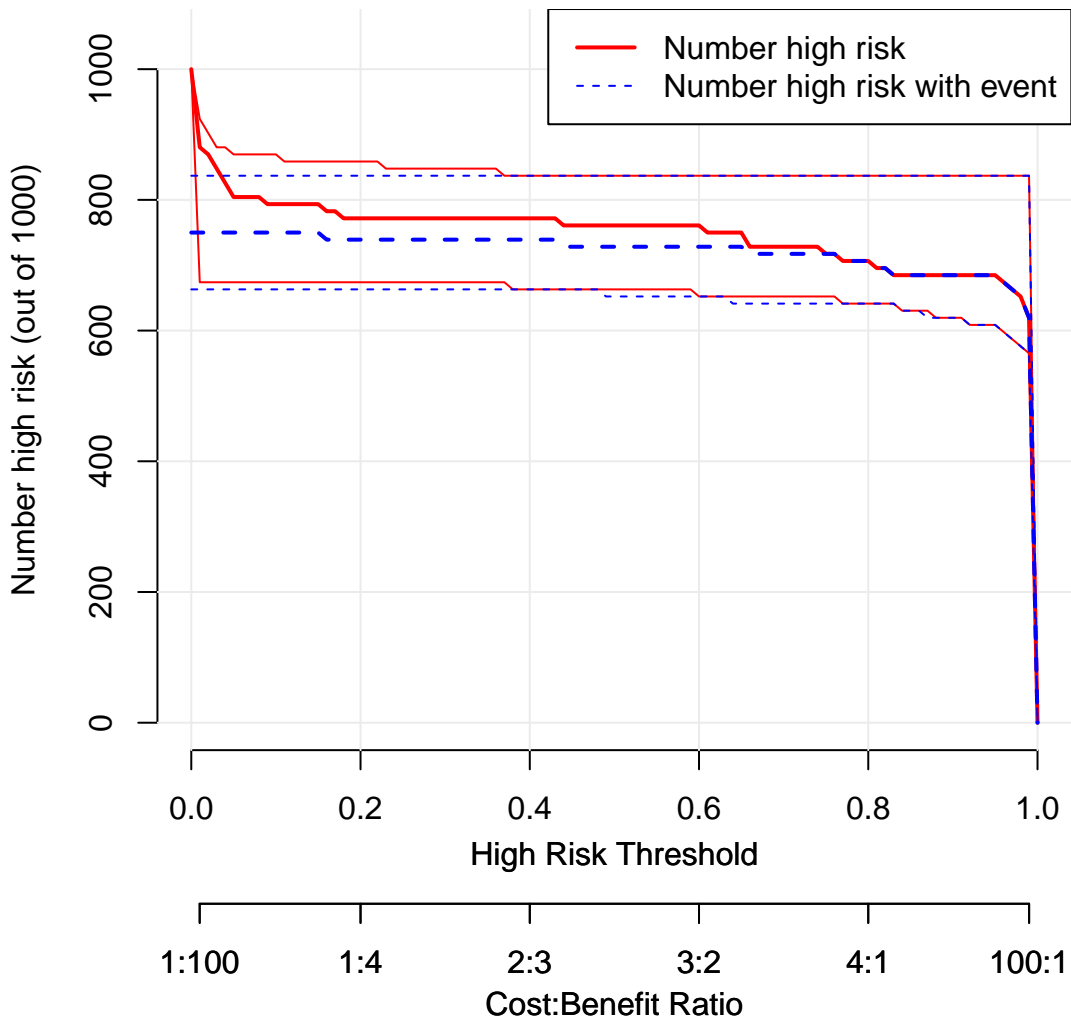

Supplement: Supplementary file 8 [file Data_Sheet_8.ZIP › Fig 8/52.Nomo/clinical_impact.pdf]

Net Benefit

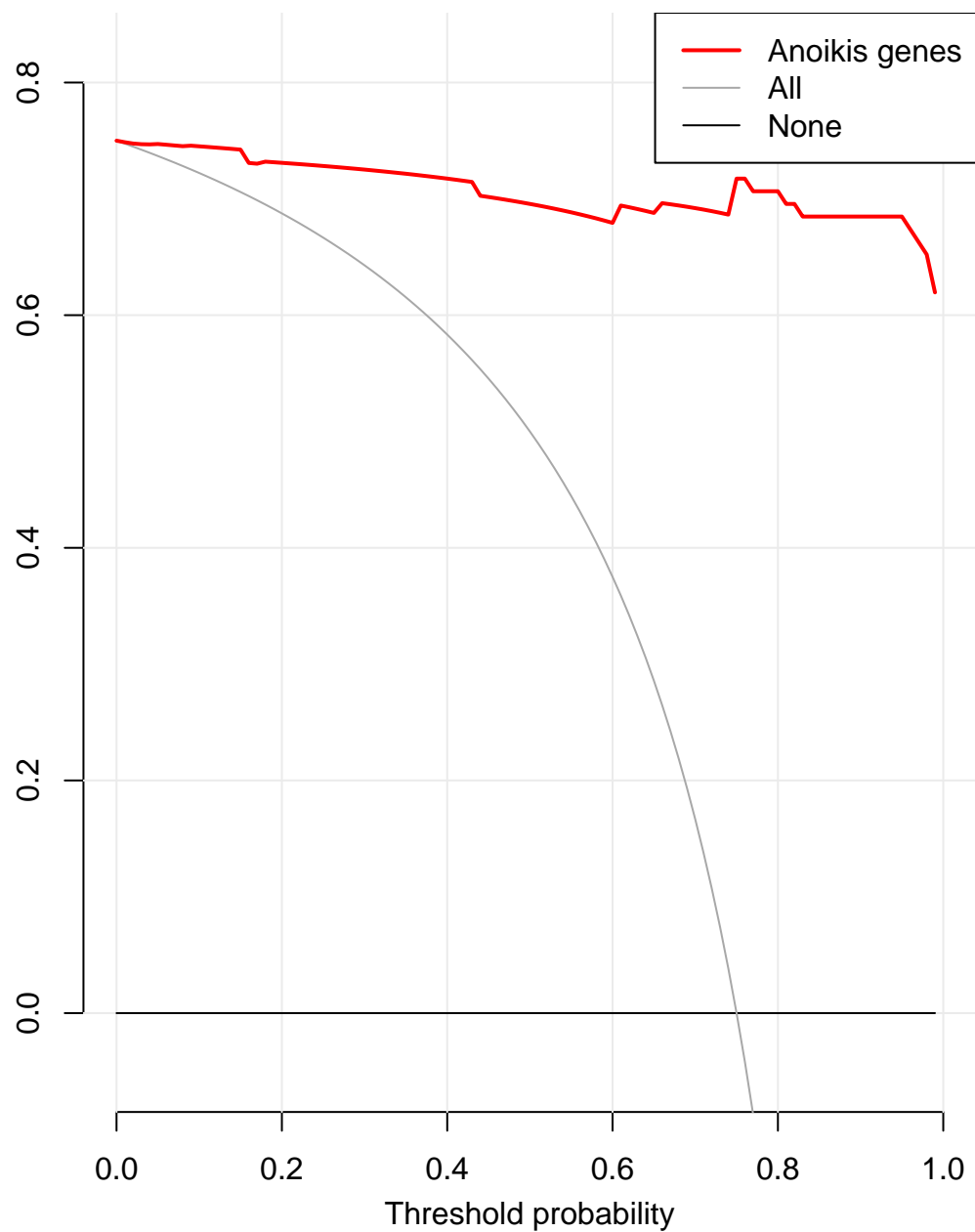

Cost:Benefit Ratio

1:100 1:4 2:3 3:2 4:1 100:1

Supplement: Supplementary file 8 [file Data_Sheet_8.ZIP › Fig 8/52.Nomo/DCA.pdf]

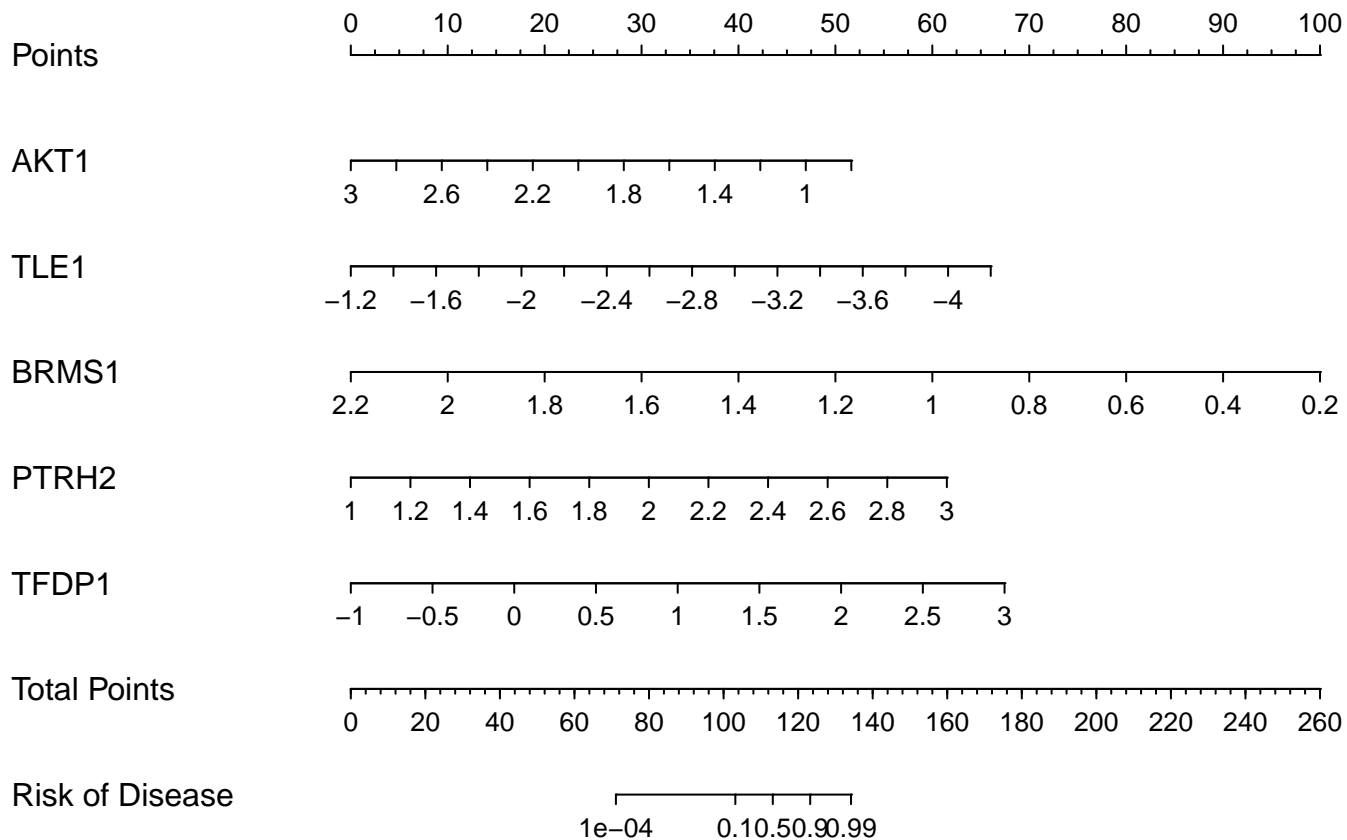

Supplement: Supplementary file 8 [file Data_Sheet_8.ZIP › Fig 8/52.Nomo/Nom.pdf]

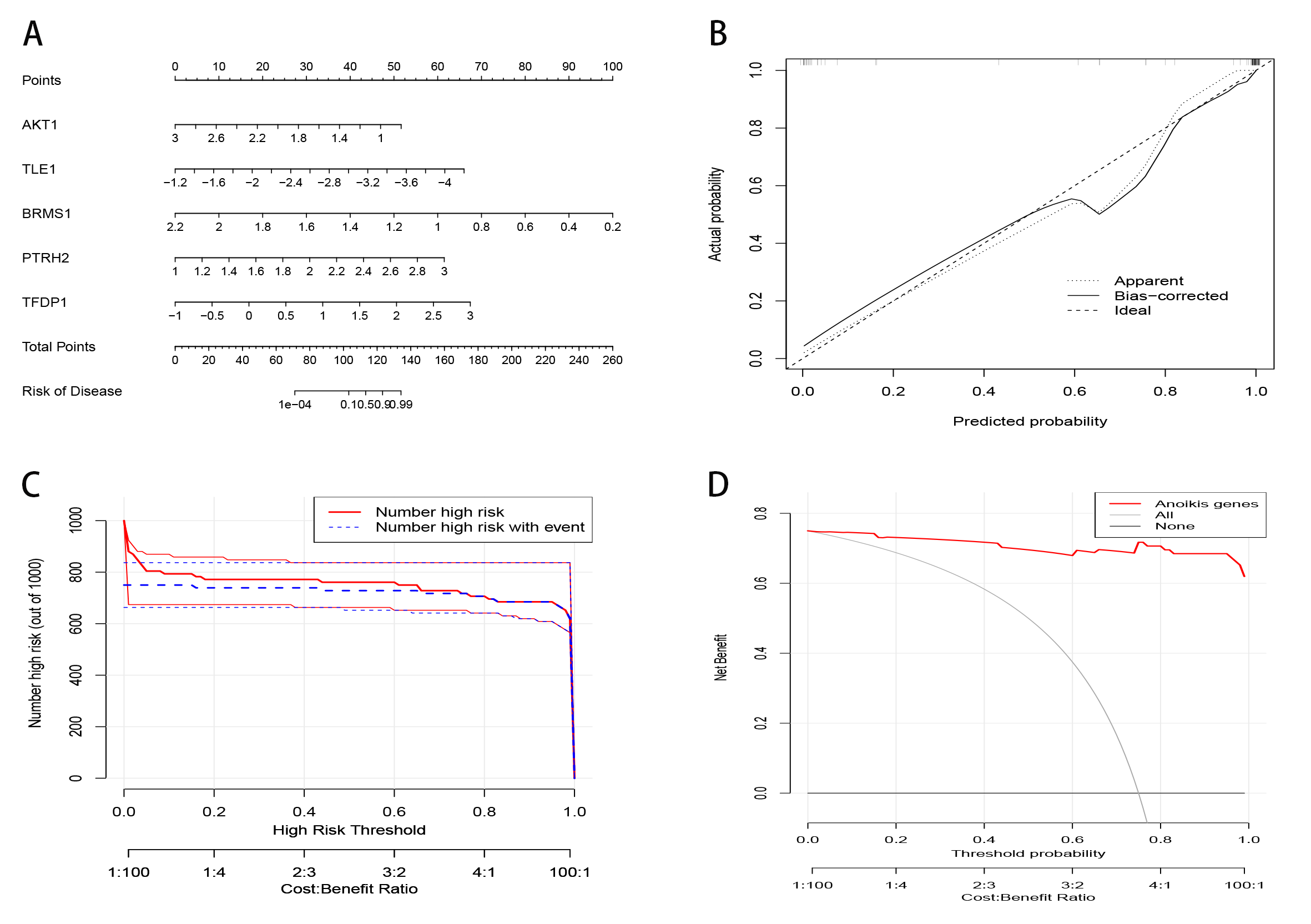

Supplement: Supplementary file 8 [file Data_Sheet_8.ZIP › Fig 8/Fig 8.tif]

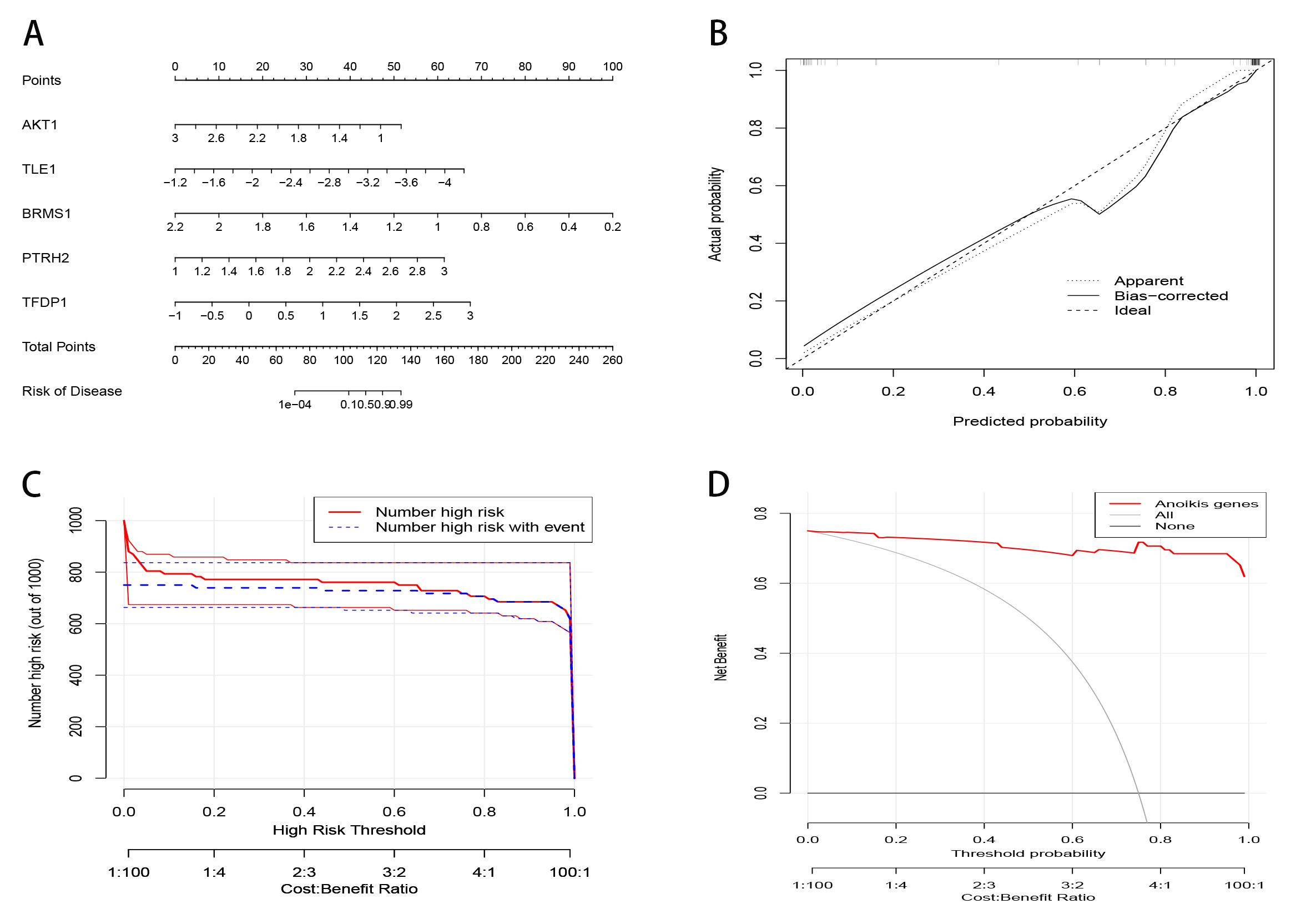

Supplement: Supplementary file 8 [file Data_Sheet_8.ZIP › Fig 8/▓╣│Σ┴╨╧▀═╝╕▒▒╛.tif]

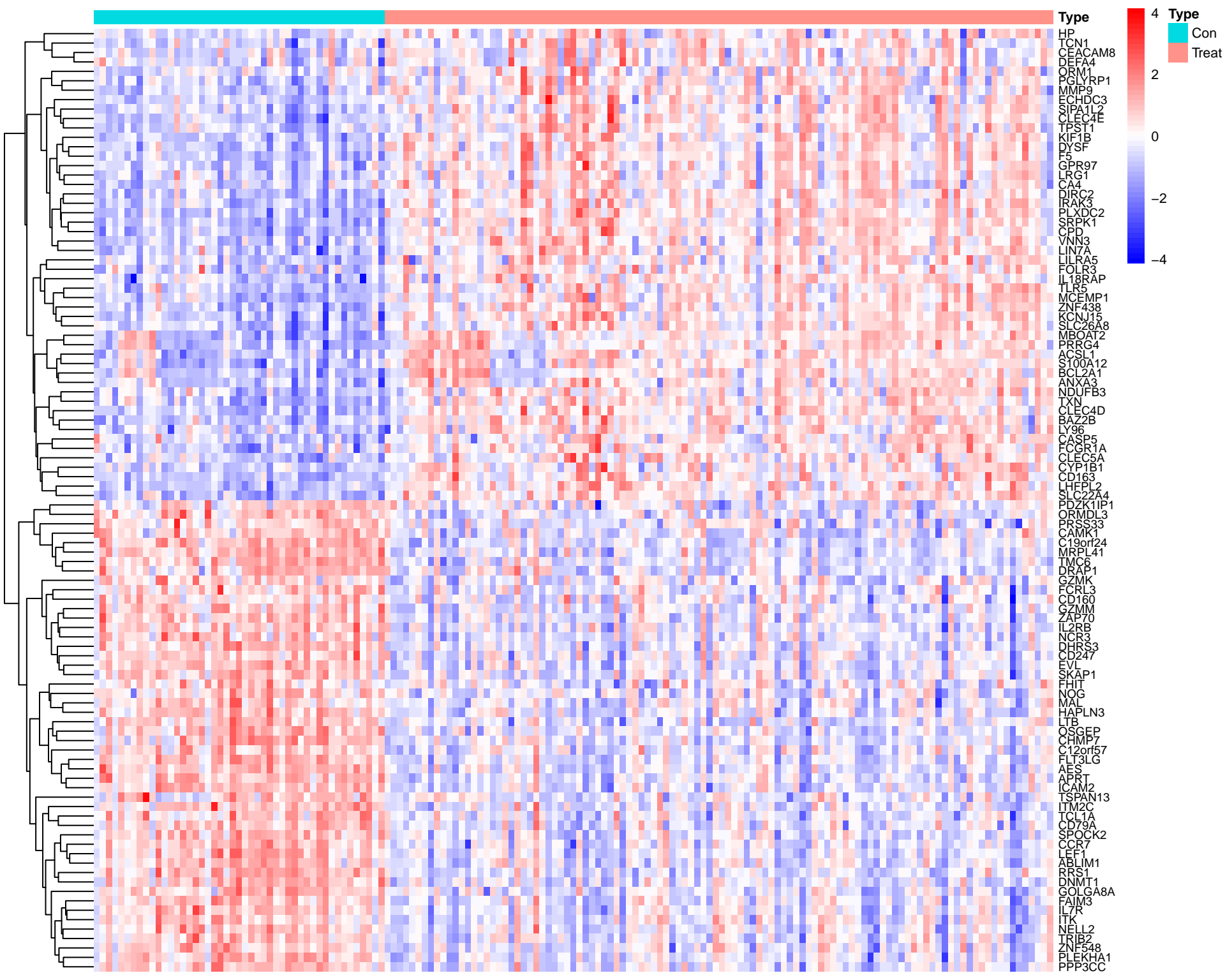

Supplement: Supplementary file 10 [file Data_Sheet_10.ZIP › 06.diff/heatmap.pdf]

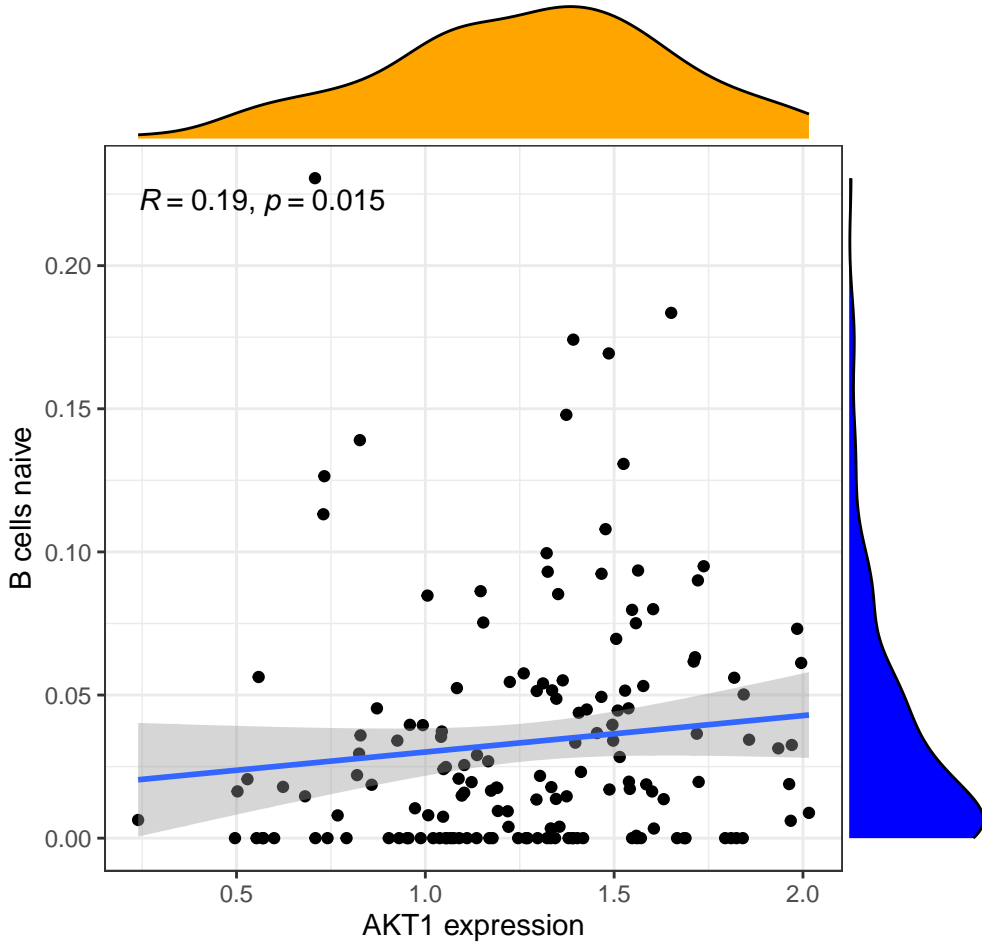

Supplement: Supplementary file 11 [file Data_Sheet_11.ZIP › 21.immuneCor/AKT1/cor.B cells naive.pdf]

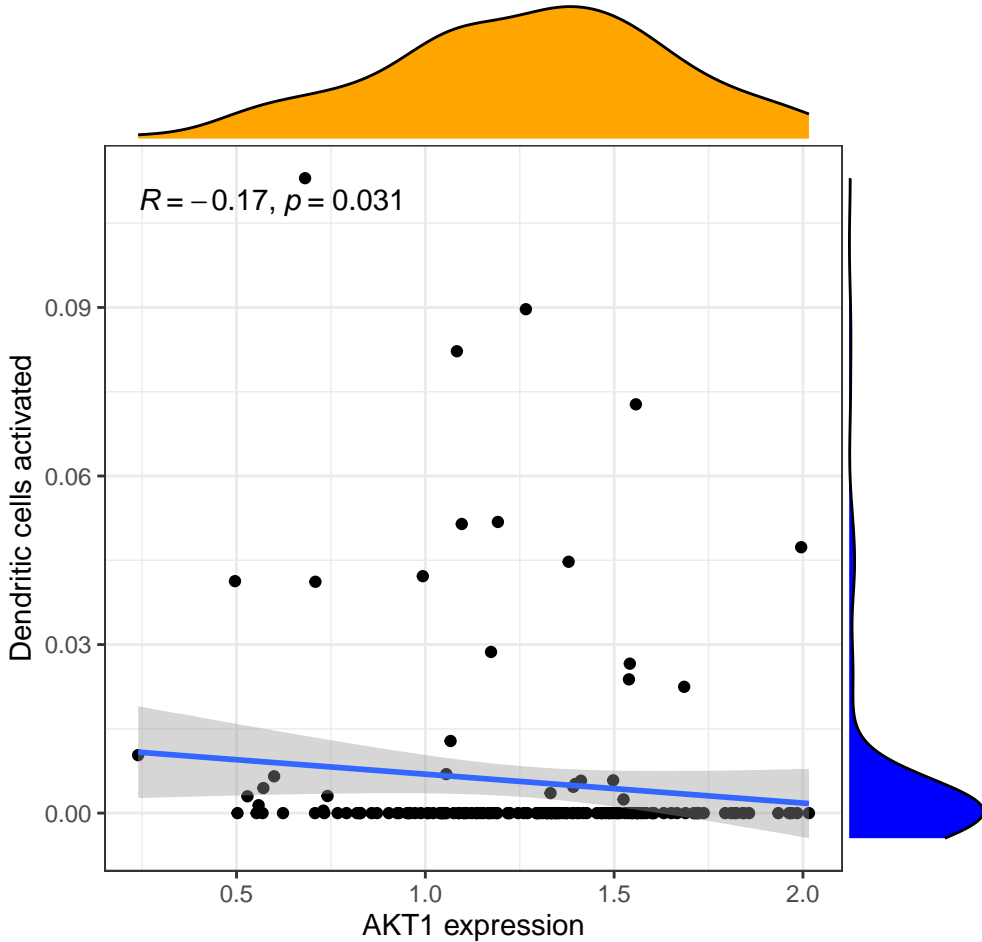

Supplement: Supplementary file 11 [file Data_Sheet_11.ZIP › 21.immuneCor/AKT1/cor.Dendritic cells activated.pdf]

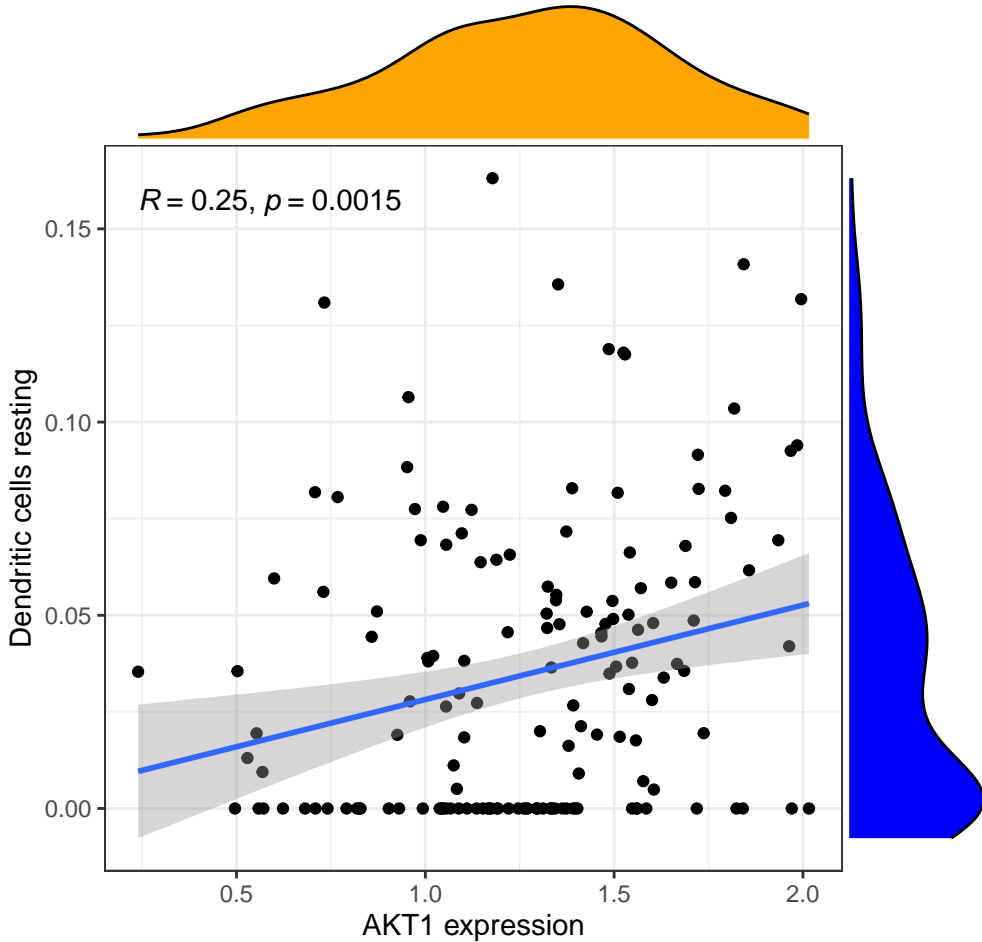

Supplement: Supplementary file 11 [file Data_Sheet_11.ZIP › 21.immuneCor/AKT1/cor.Dendritic cells resting.pdf]

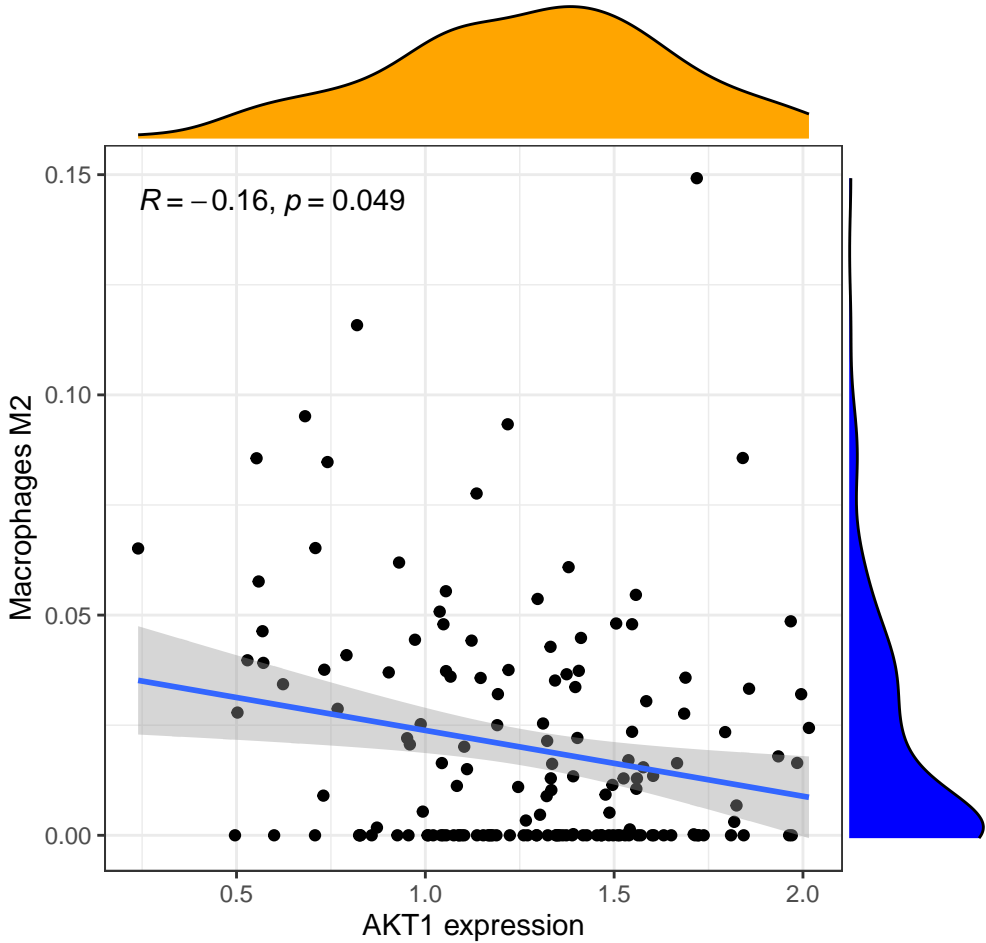

Supplement: Supplementary file 11 [file Data_Sheet_11.ZIP › 21.immuneCor/AKT1/cor.Macrophages M2.pdf]

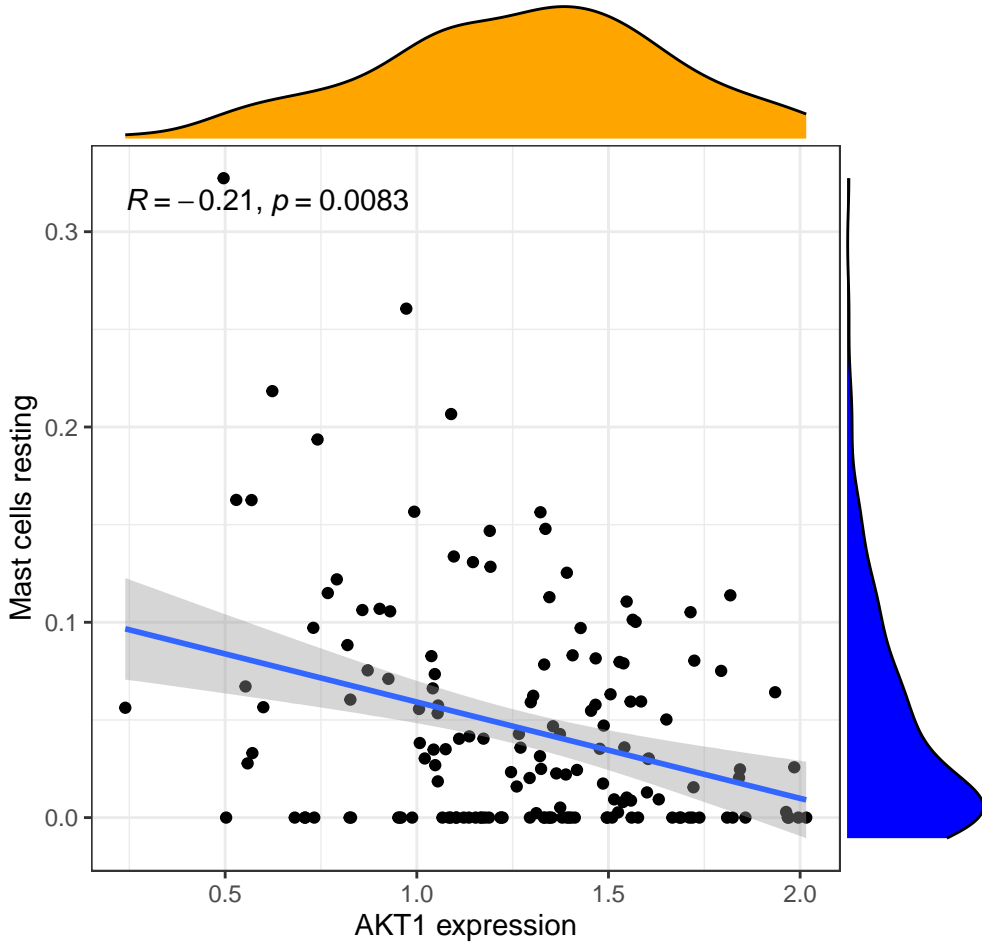

Supplement: Supplementary file 11 [file Data_Sheet_11.ZIP › 21.immuneCor/AKT1/cor.Mast cells resting.pdf]

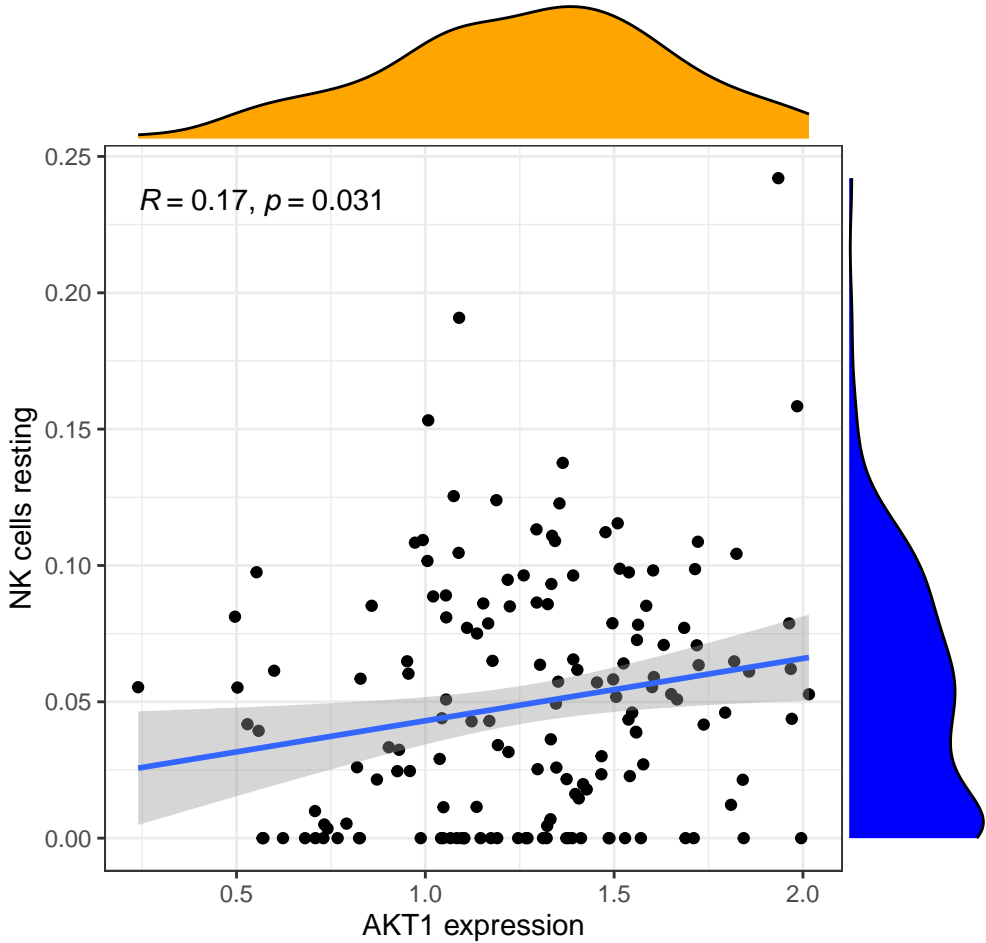

Supplement: Supplementary file 11 [file Data_Sheet_11.ZIP › 21.immuneCor/AKT1/cor.NK cells resting.pdf]

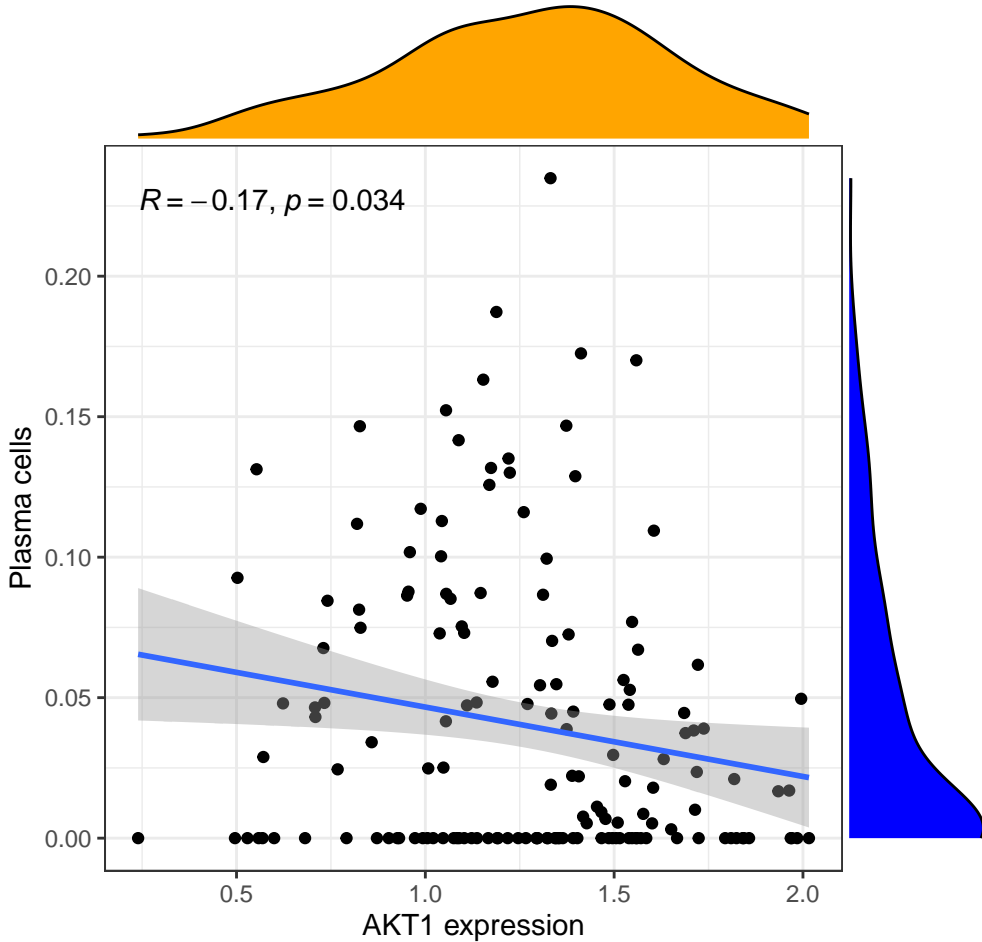

Supplement: Supplementary file 11 [file Data_Sheet_11.ZIP › 21.immuneCor/AKT1/cor.Plasma cells.pdf]

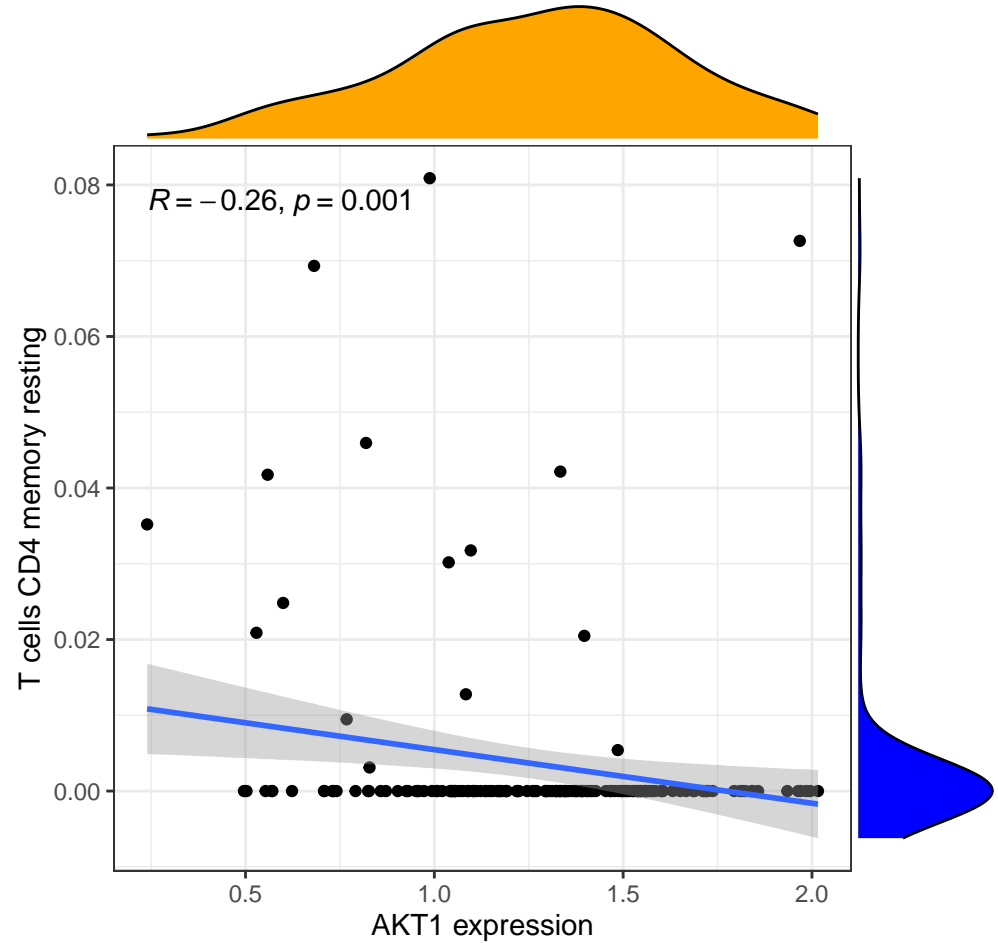

Supplement: Supplementary file 11 [file Data_Sheet_11.ZIP › 21.immuneCor/AKT1/cor.T cells CD4 memory resting.pdf]

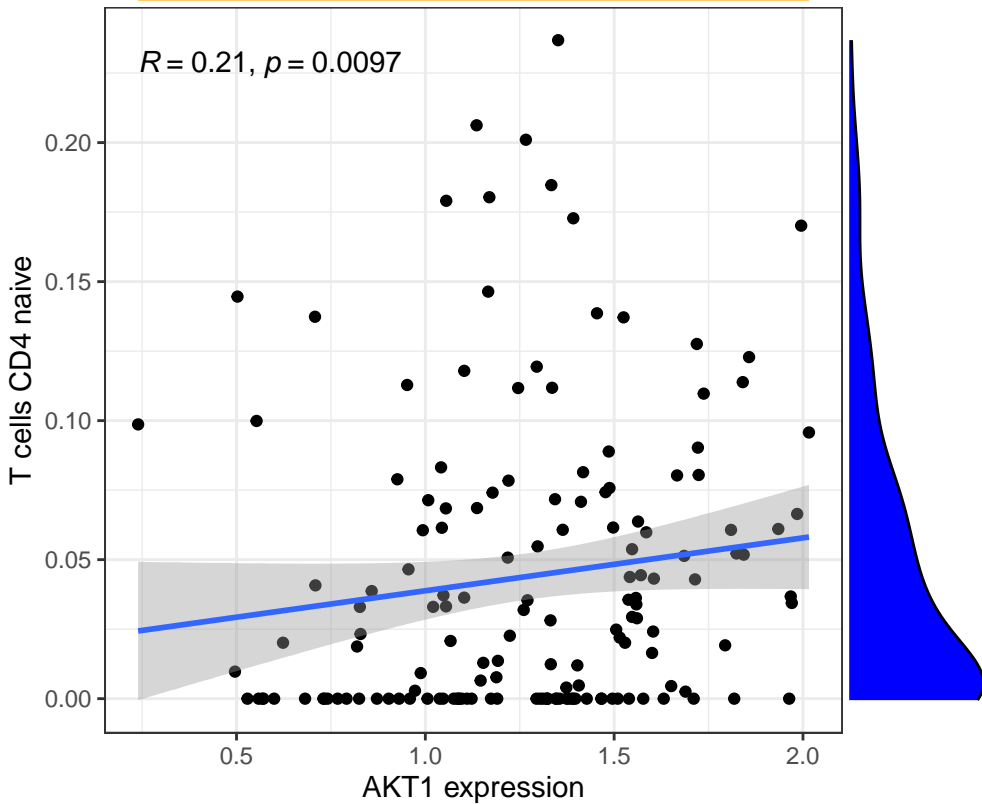

Supplement: Supplementary file 11 [file Data_Sheet_11.ZIP › 21.immuneCor/AKT1/cor.T cells CD4 naive.pdf]

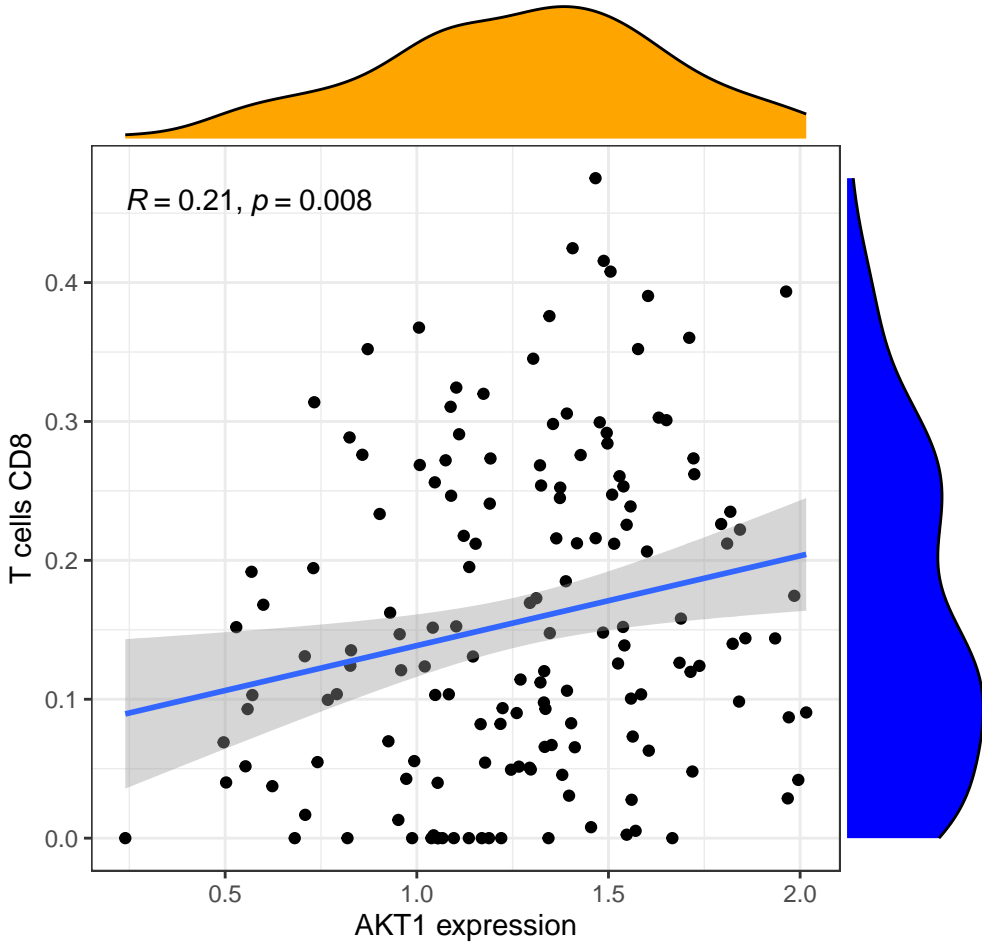

Supplement: Supplementary file 11 [file Data_Sheet_11.ZIP › 21.immuneCor/AKT1/cor.T cells CD8.pdf]

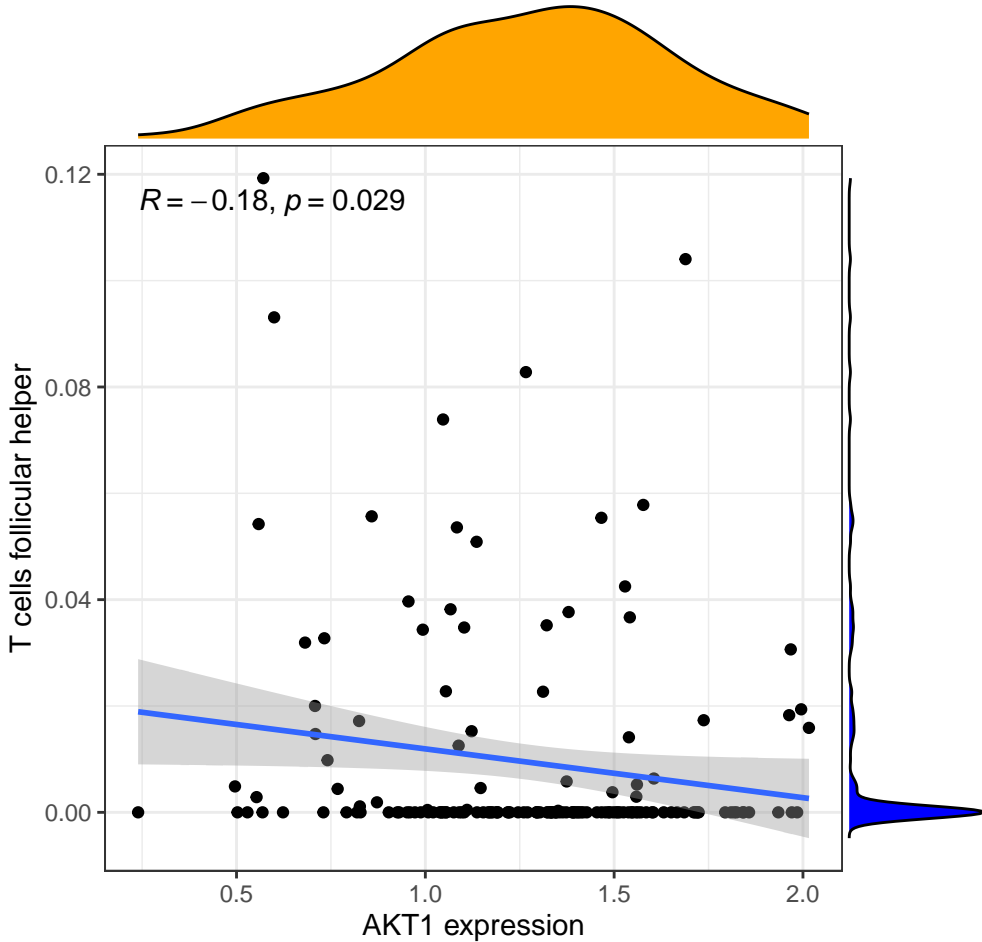

Supplement: Supplementary file 11 [file Data_Sheet_11.ZIP › 21.immuneCor/AKT1/cor.T cells follicular helper.pdf]

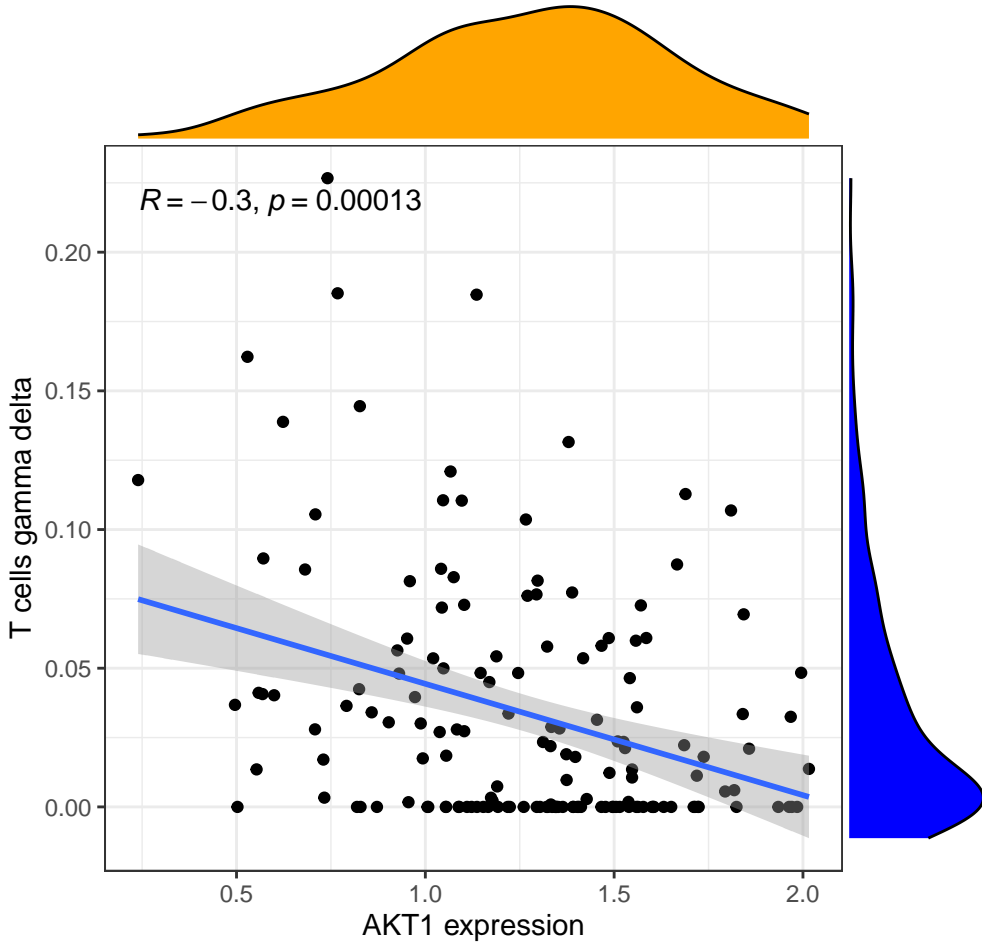

Supplement: Supplementary file 11 [file Data_Sheet_11.ZIP › 21.immuneCor/AKT1/cor.T cells gamma delta.pdf]

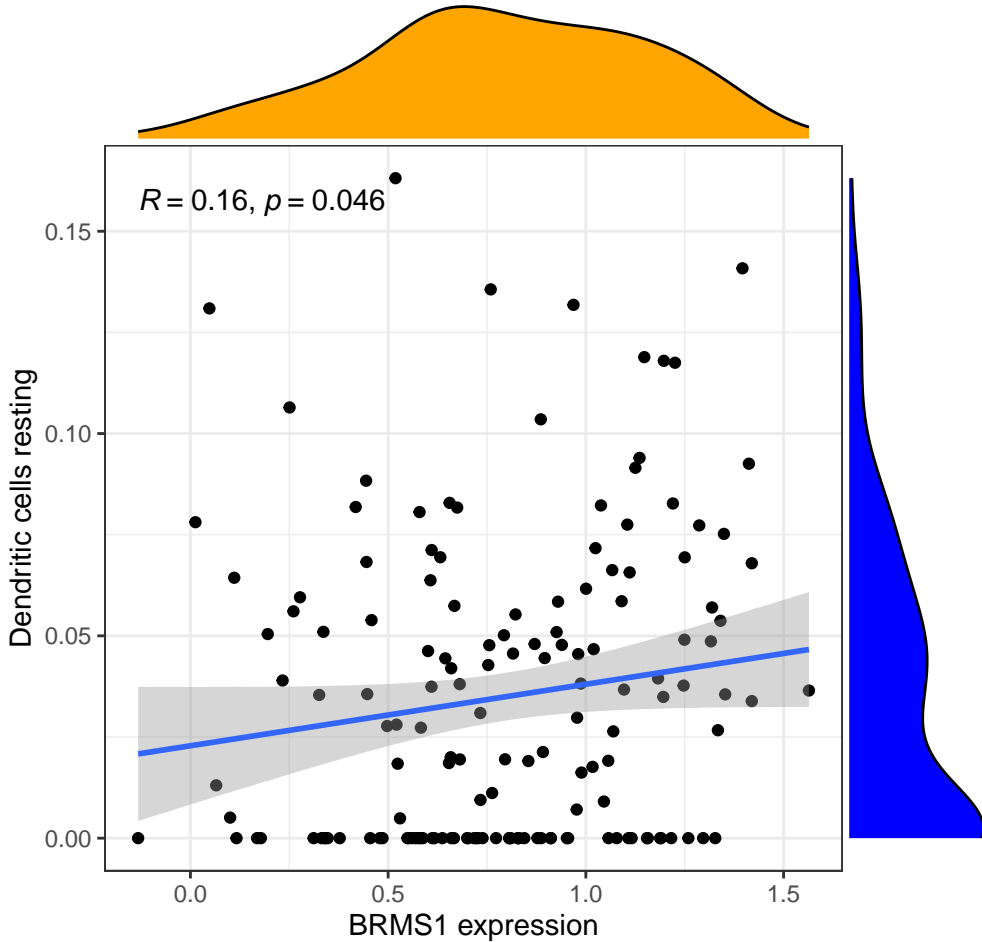

Supplement: Supplementary file 11 [file Data_Sheet_11.ZIP › 21.immuneCor/BRMS1/cor.Dendritic cells resting.pdf]

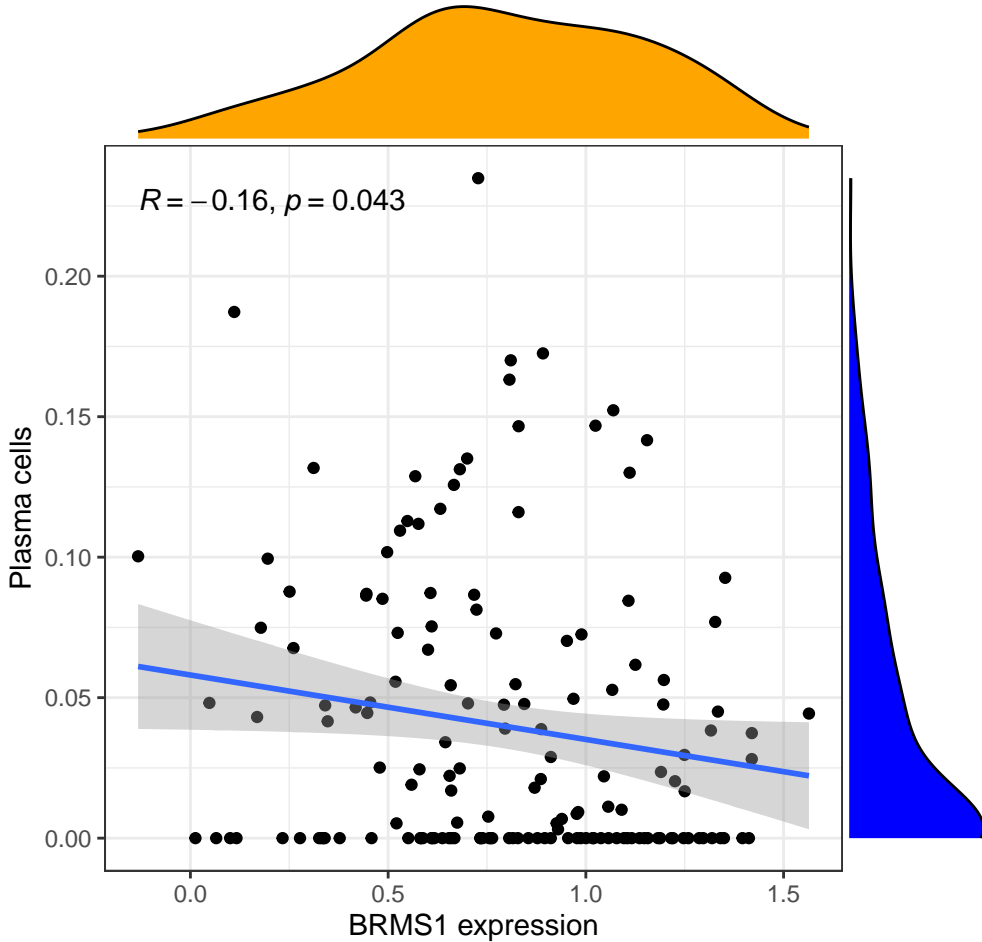

Supplement: Supplementary file 11 [file Data_Sheet_11.ZIP › 21.immuneCor/BRMS1/cor.Plasma cells.pdf]

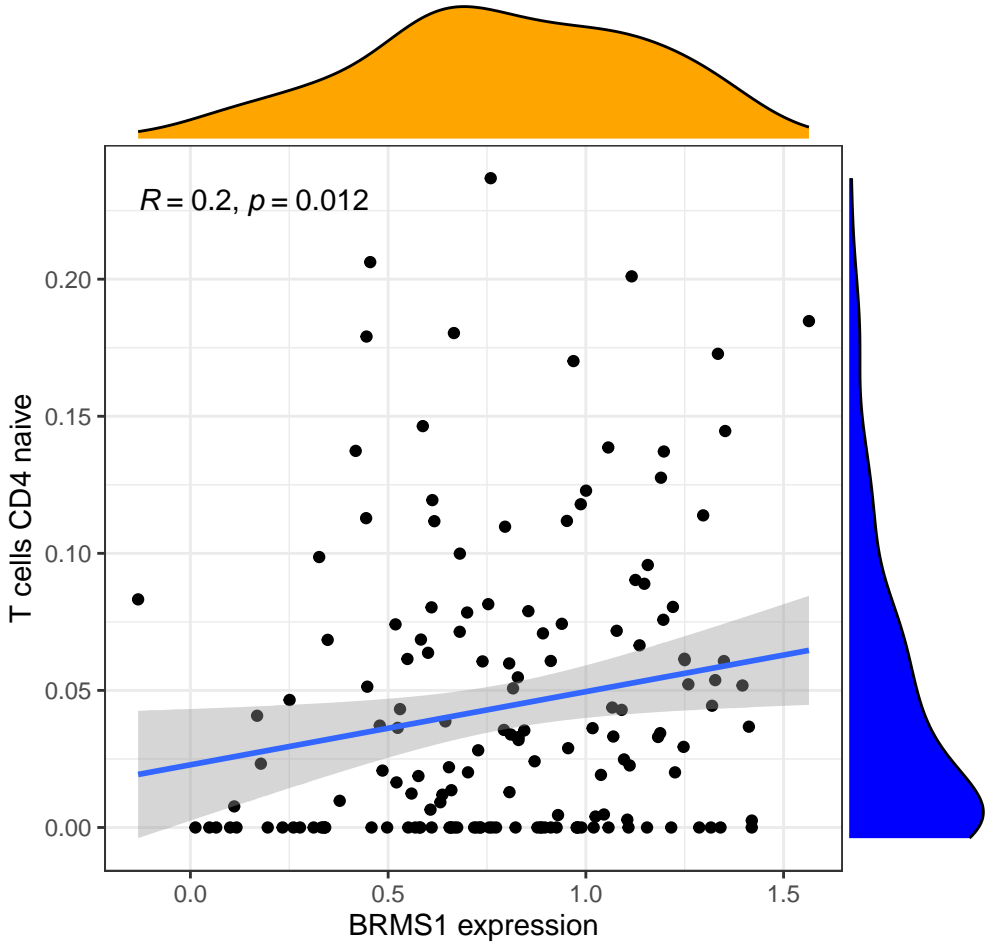

Supplement: Supplementary file 11 [file Data_Sheet_11.ZIP › 21.immuneCor/BRMS1/cor.T cells CD4 naive.pdf]

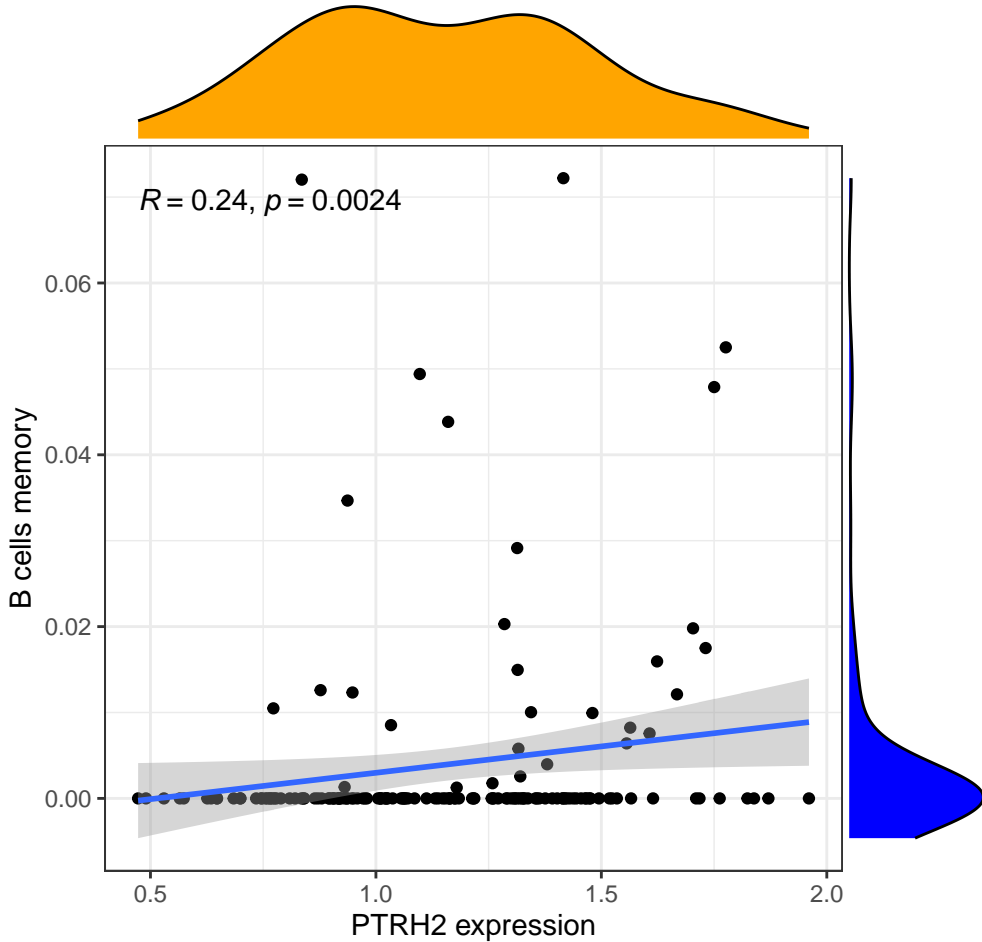

Supplement: Supplementary file 11 [file Data_Sheet_11.ZIP › 21.immuneCor/PTRH2/cor.B cells memory.pdf]

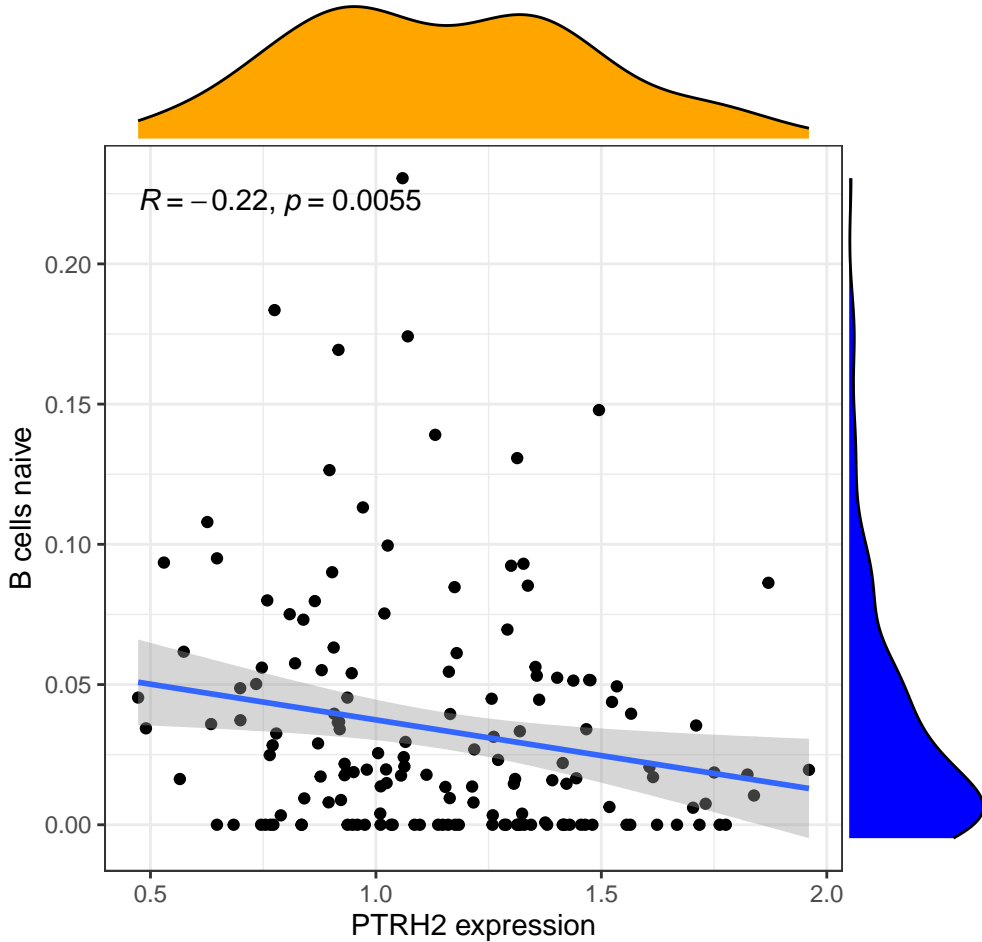

Supplement: Supplementary file 11 [file Data_Sheet_11.ZIP › 21.immuneCor/PTRH2/cor.B cells naive.pdf]

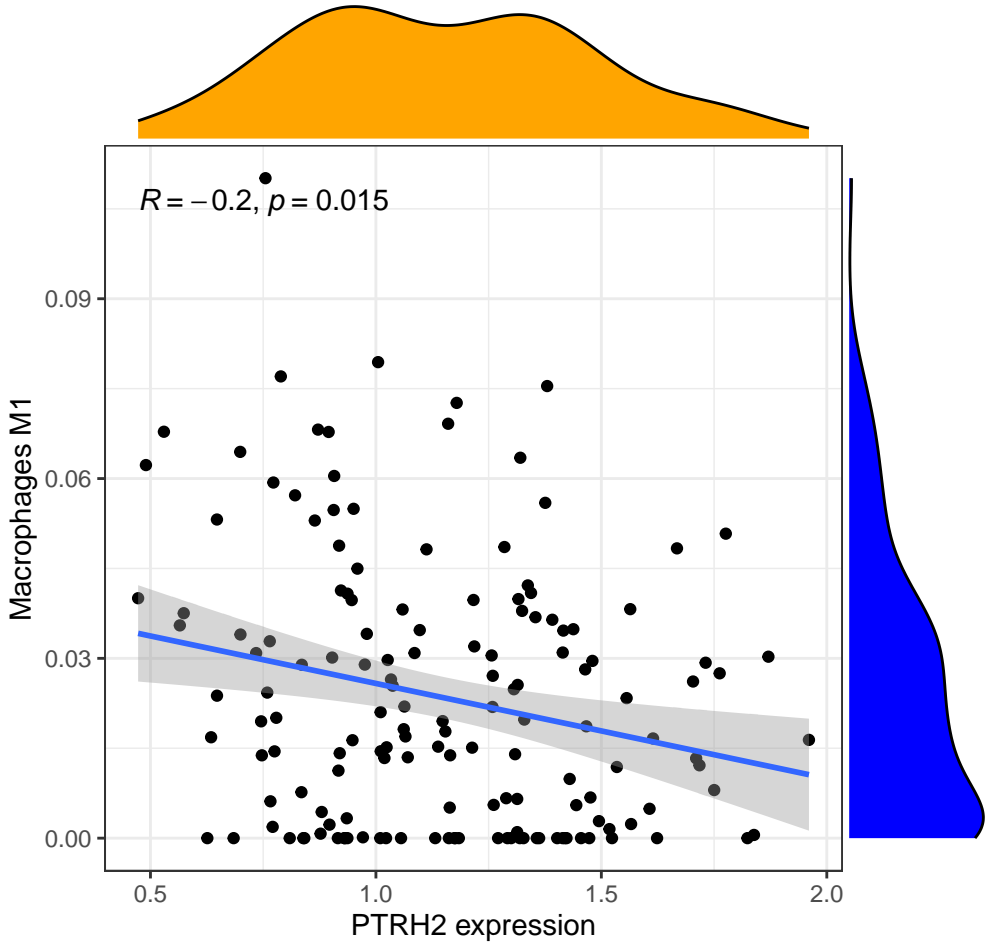

Supplement: Supplementary file 11 [file Data_Sheet_11.ZIP › 21.immuneCor/PTRH2/cor.Macrophages M1.pdf]

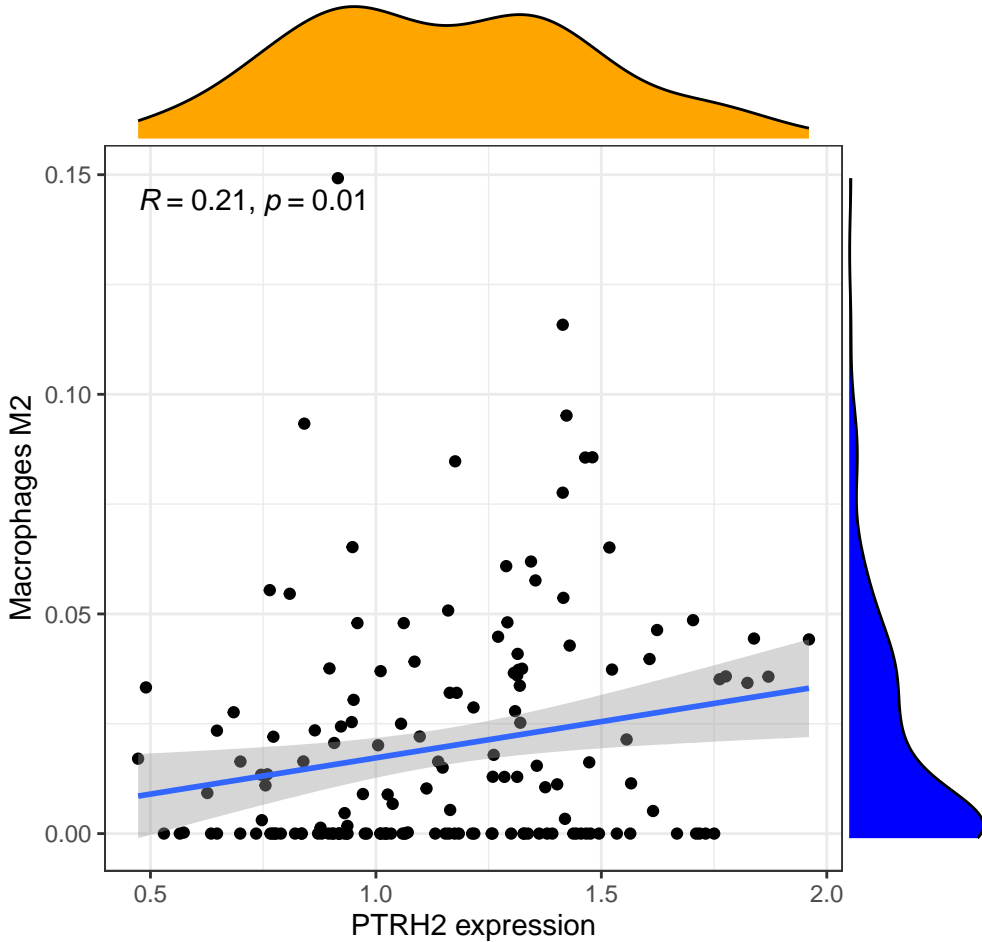

Supplement: Supplementary file 11 [file Data_Sheet_11.ZIP › 21.immuneCor/PTRH2/cor.Macrophages M2.pdf]

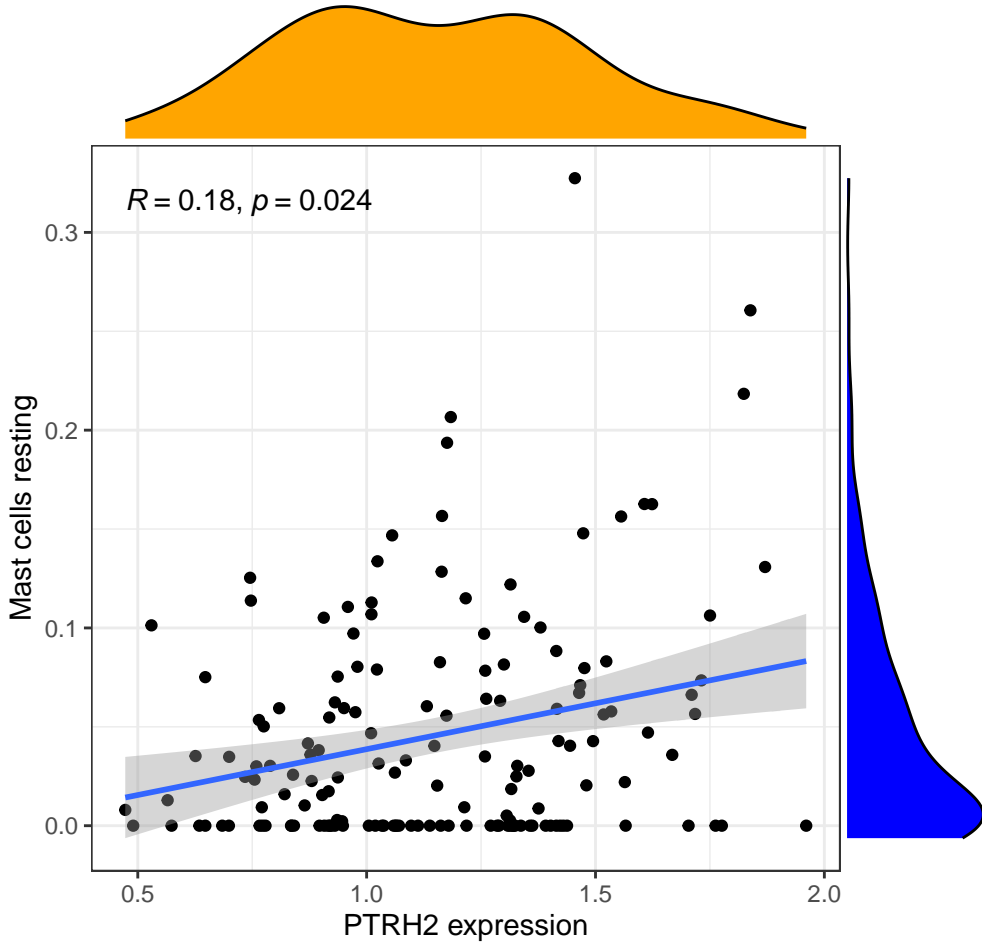

Supplement: Supplementary file 11 [file Data_Sheet_11.ZIP › 21.immuneCor/PTRH2/cor.Mast cells resting.pdf]

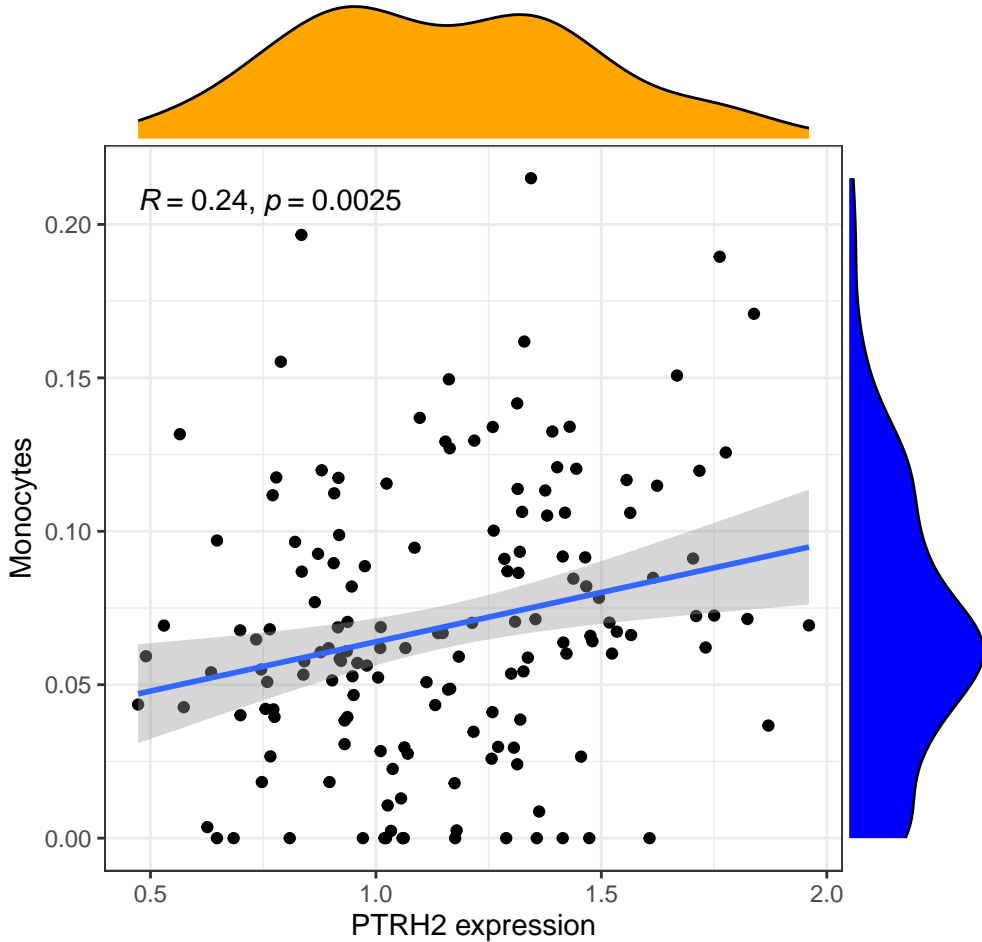

Supplement: Supplementary file 11 [file Data_Sheet_11.ZIP › 21.immuneCor/PTRH2/cor.Monocytes.pdf]
