# Supplementary material for: Identification of anoikis-related genes classification patterns and immune infiltration characterization in ischemic stroke based on machine learning
Source: Front Aging Neurosci. 2023 Mar 23;15:1142163. doi: 10.3389/fnagi.2023.1142163 (PMC10076550; doi:10.3389/fnagi.2023.1142163)
Supplement: Supplementary file 3 [file Data_Sheet_3.ZIP › Fig 6/50.RF/geneImportance.pdf]

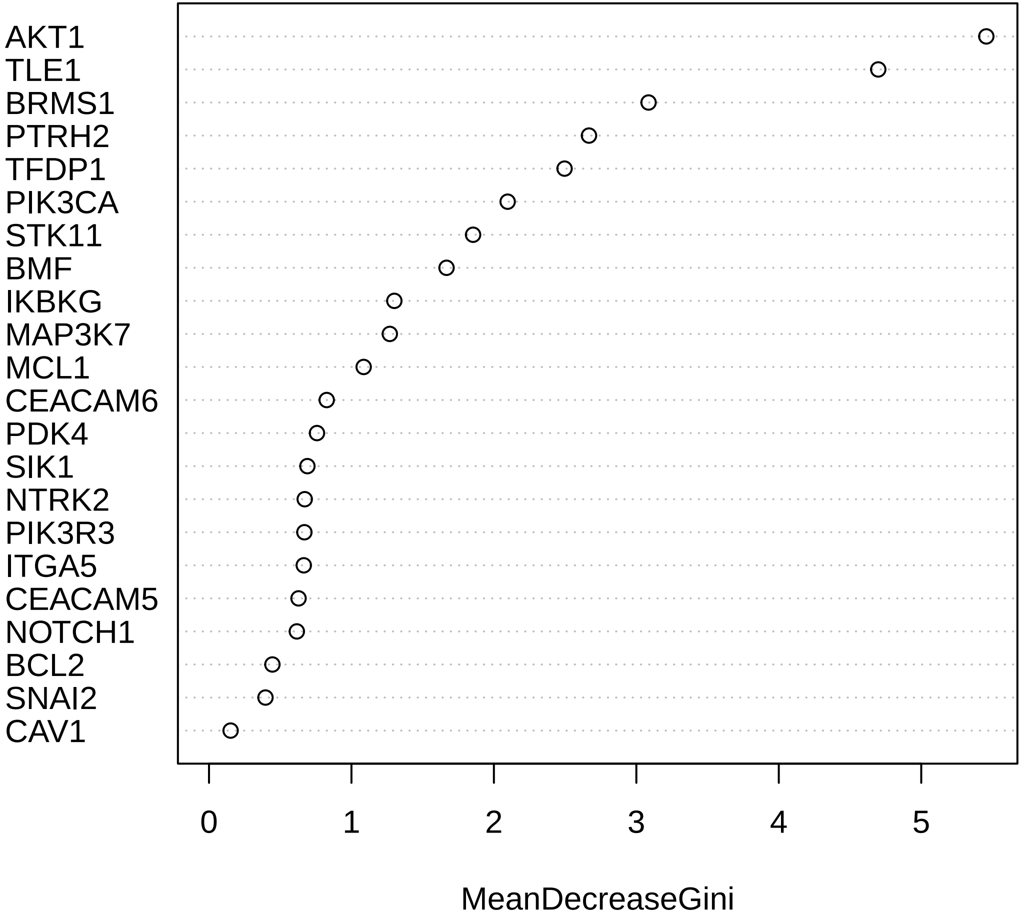
AKT1
TLE1
BRMS1
PTRH2
TFDP1
PIK3CA
STK11
BMF
IKBKG
MAP3K7
MCL1
CEACAM6
PDK4
SIK1
NTRK2
PIK3R3
ITGA5
CEACAM5
NOTCH1
BCL2
SNAI2
CAV1
0
1
2
3
4
5
MeanDecreaseGini
