# Supplementary figures and images for: Identification of anoikis-related genes classification patterns and immune infiltration characterization in ischemic stroke based on machine learning (part 2 of 2)
Source: Front Aging Neurosci. 2023 Mar 23;15:1142163. doi: 10.3389/fnagi.2023.1142163 (PMC10076550; doi:10.3389/fnagi.2023.1142163)

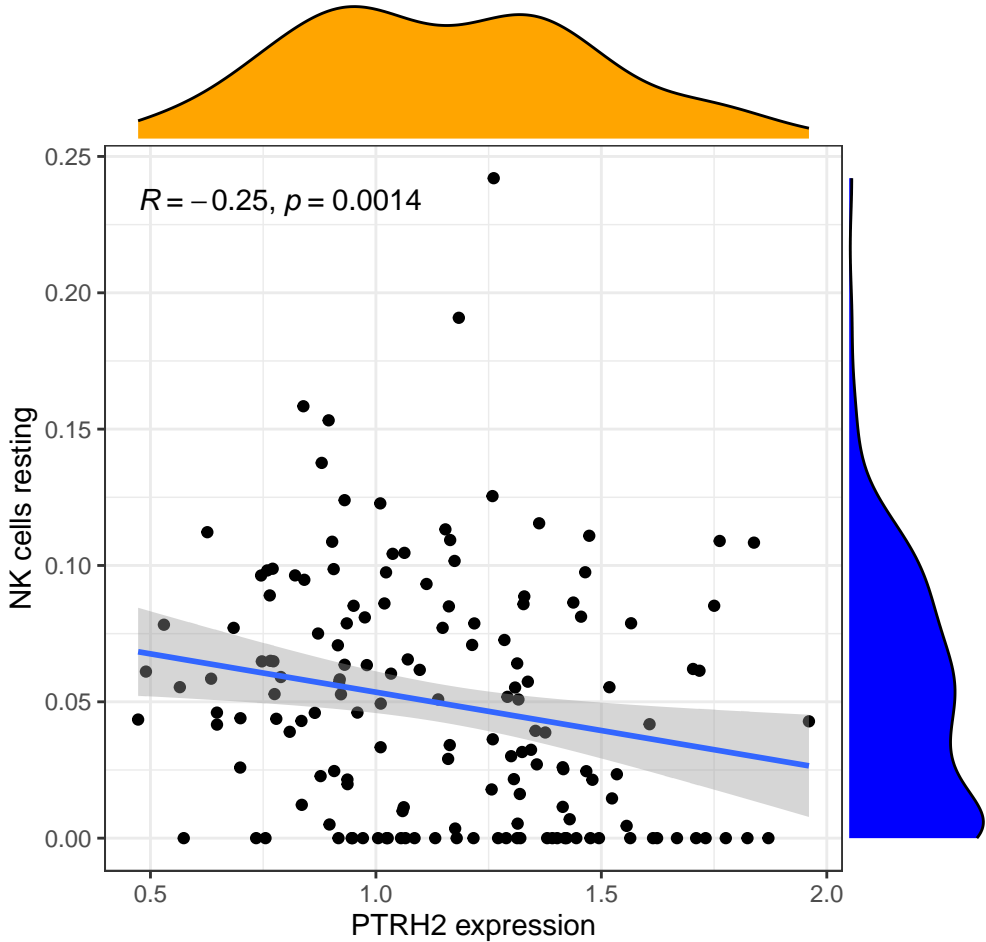

Supplement: Supplementary file 11 [file Data_Sheet_11.ZIP › 21.immuneCor/PTRH2/cor.NK cells resting.pdf]

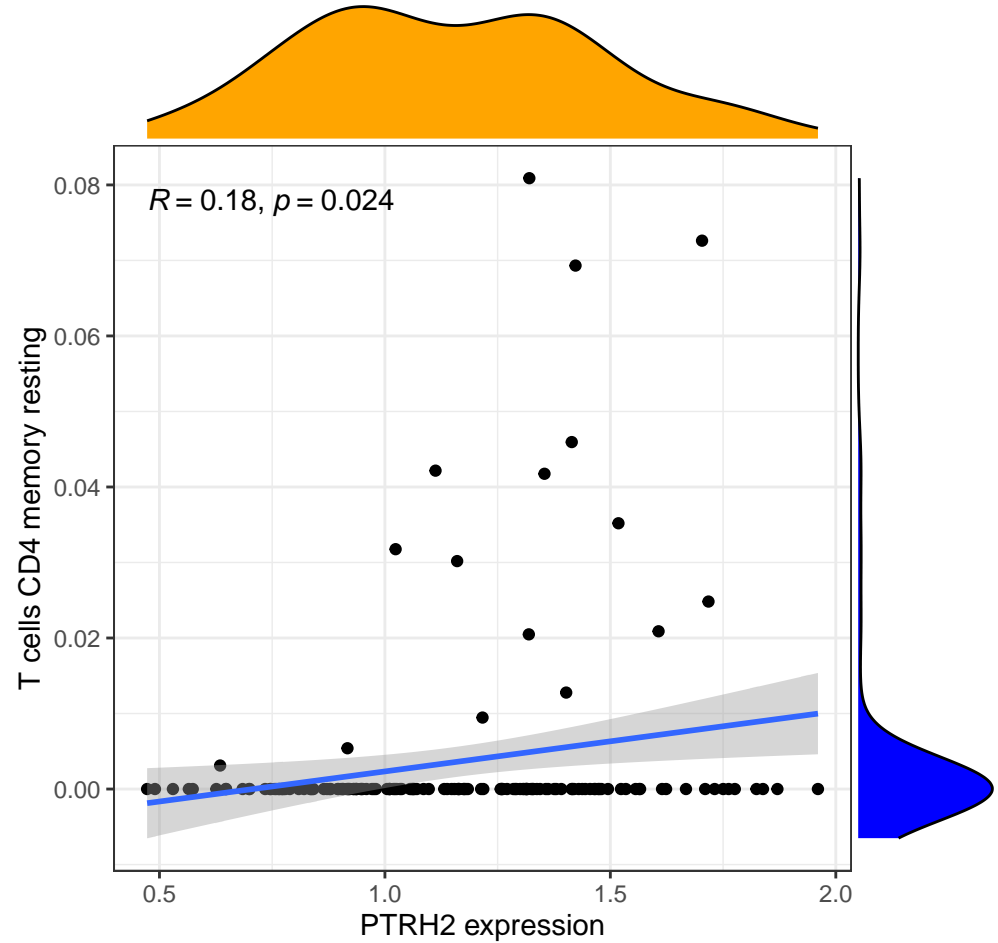

Supplement: Supplementary file 11 [file Data_Sheet_11.ZIP › 21.immuneCor/PTRH2/cor.T cells CD4 memory resting.pdf]

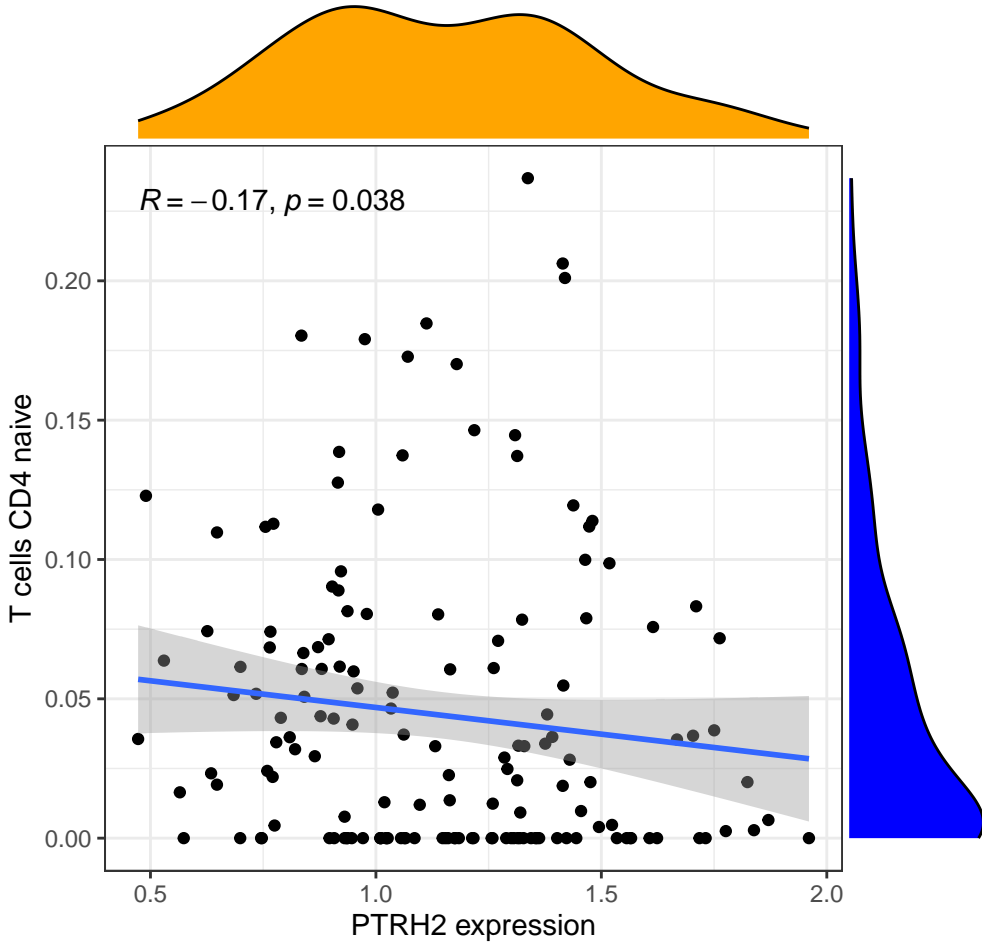

Supplement: Supplementary file 11 [file Data_Sheet_11.ZIP › 21.immuneCor/PTRH2/cor.T cells CD4 naive.pdf]

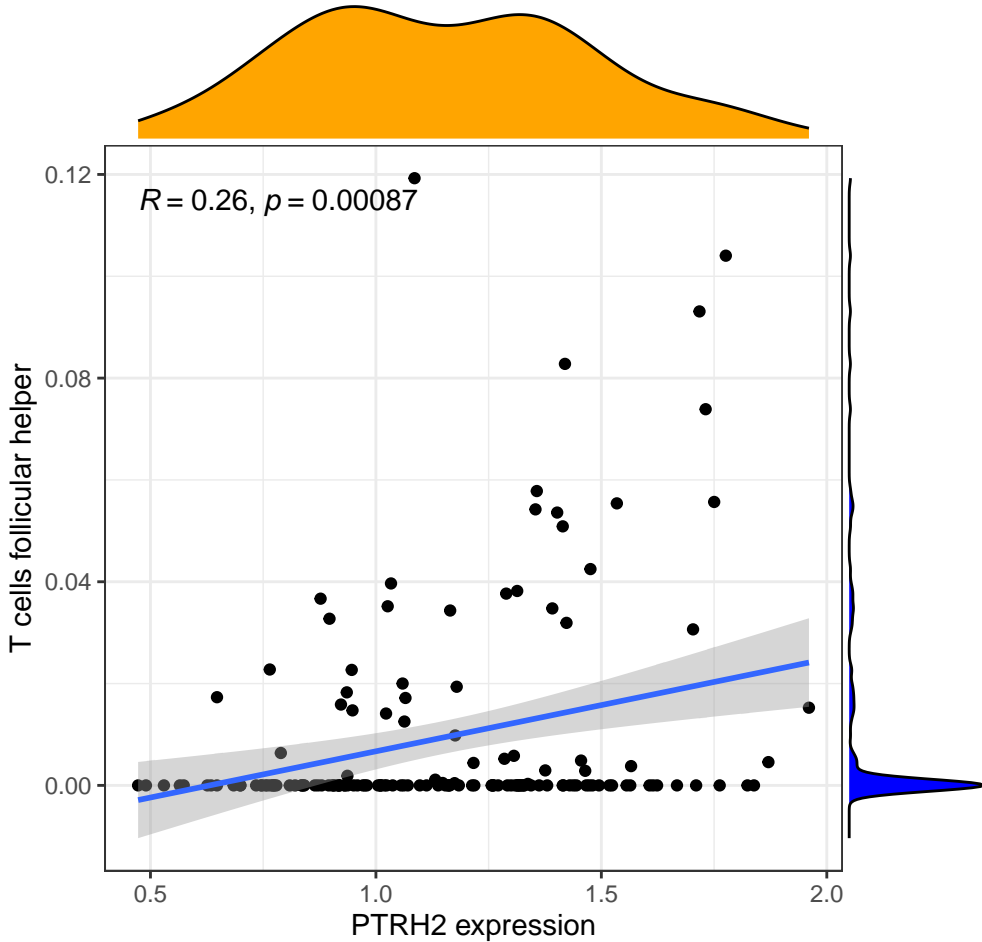

Supplement: Supplementary file 11 [file Data_Sheet_11.ZIP › 21.immuneCor/PTRH2/cor.T cells follicular helper.pdf]

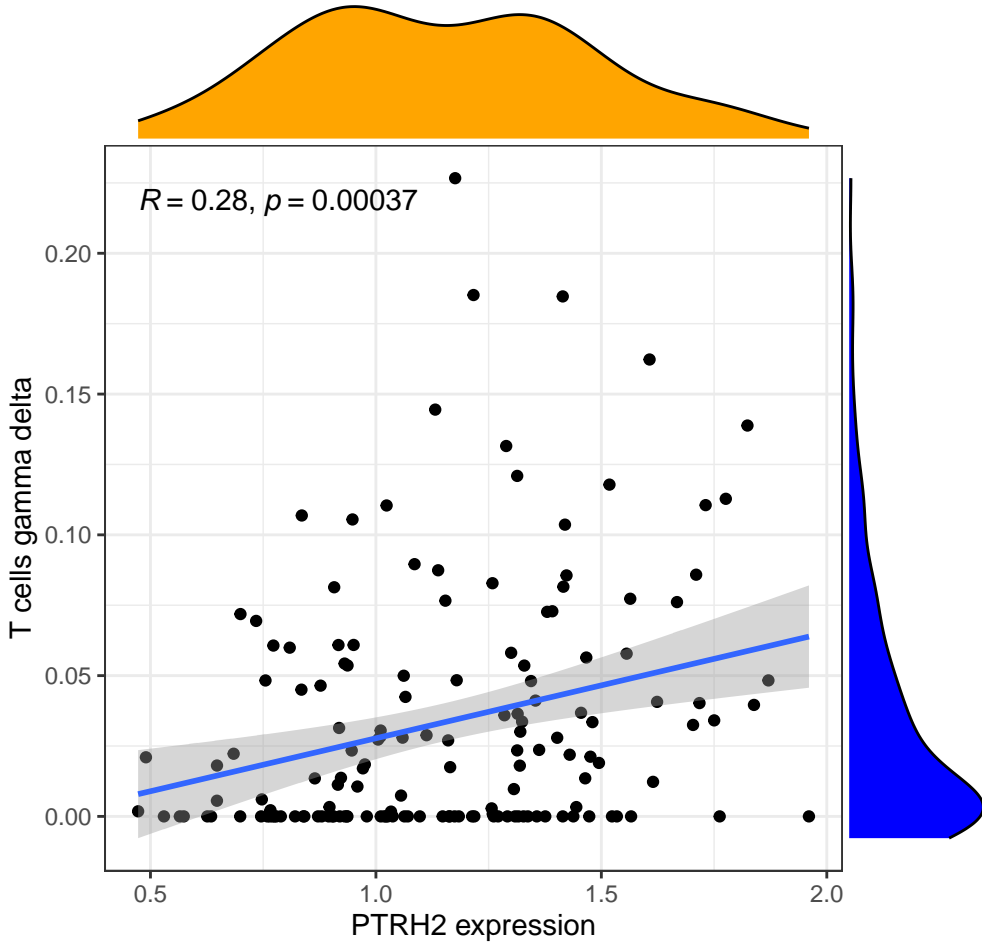

Supplement: Supplementary file 11 [file Data_Sheet_11.ZIP › 21.immuneCor/PTRH2/cor.T cells gamma delta.pdf]

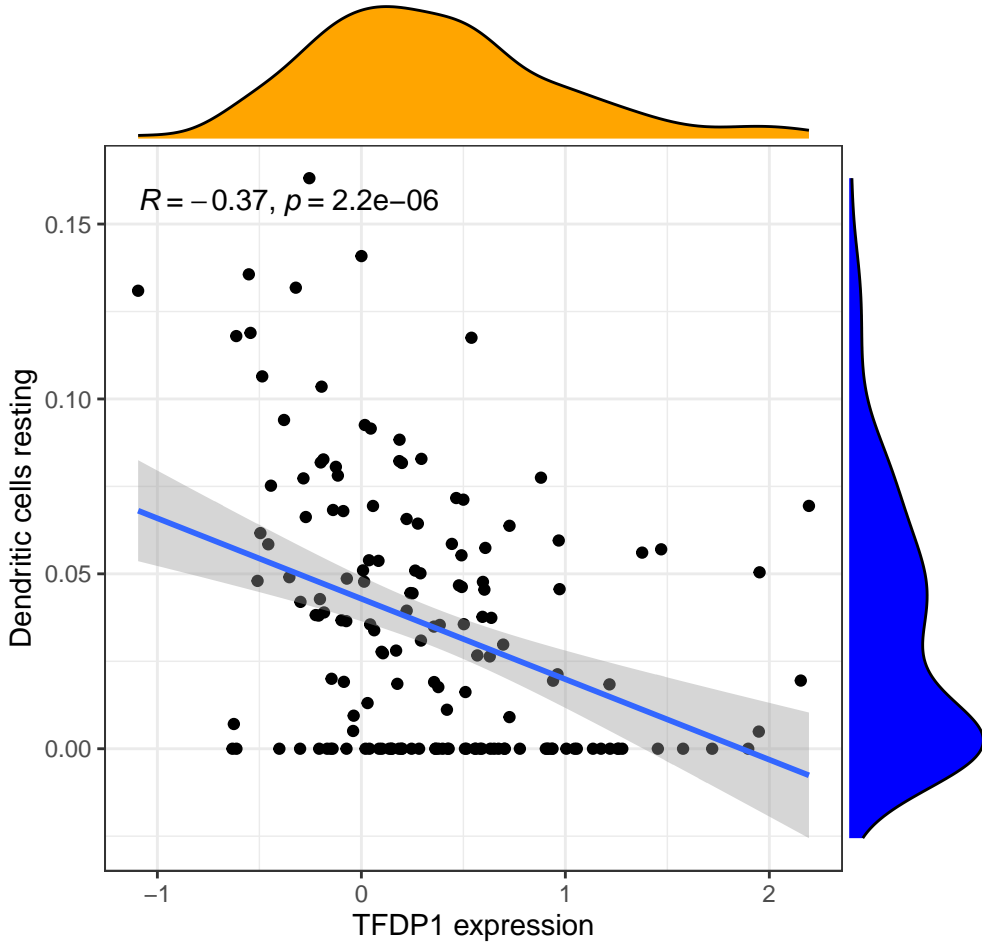

Supplement: Supplementary file 11 [file Data_Sheet_11.ZIP › 21.immuneCor/TFDP1/cor.Dendritic cells resting.pdf]

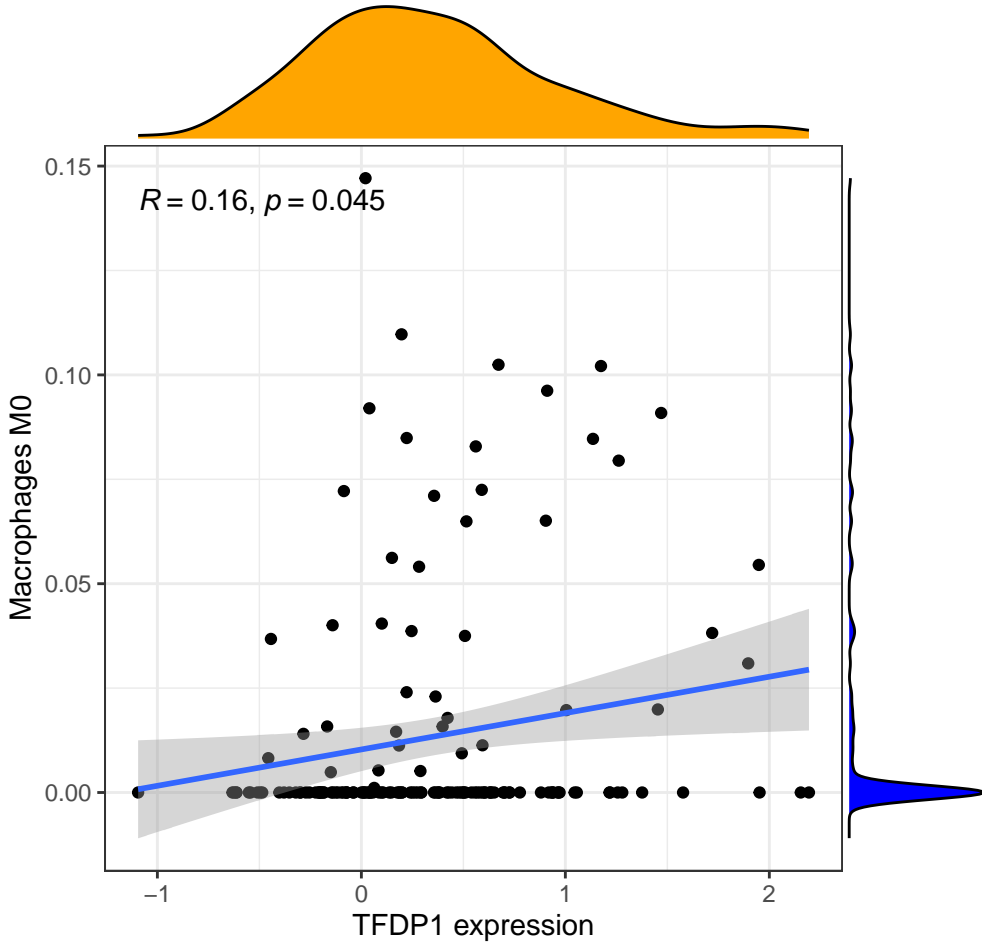

Supplement: Supplementary file 11 [file Data_Sheet_11.ZIP › 21.immuneCor/TFDP1/cor.Macrophages M0.pdf]

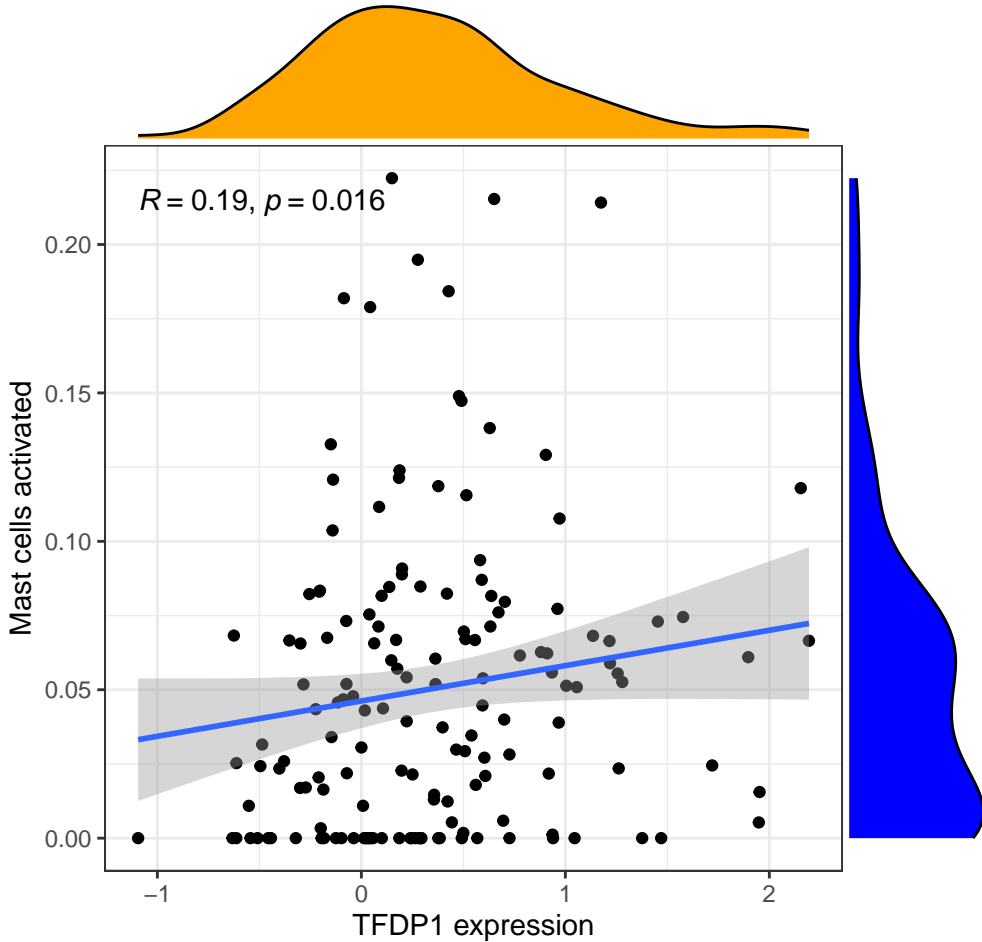

Supplement: Supplementary file 11 [file Data_Sheet_11.ZIP › 21.immuneCor/TFDP1/cor.Mast cells activated.pdf]

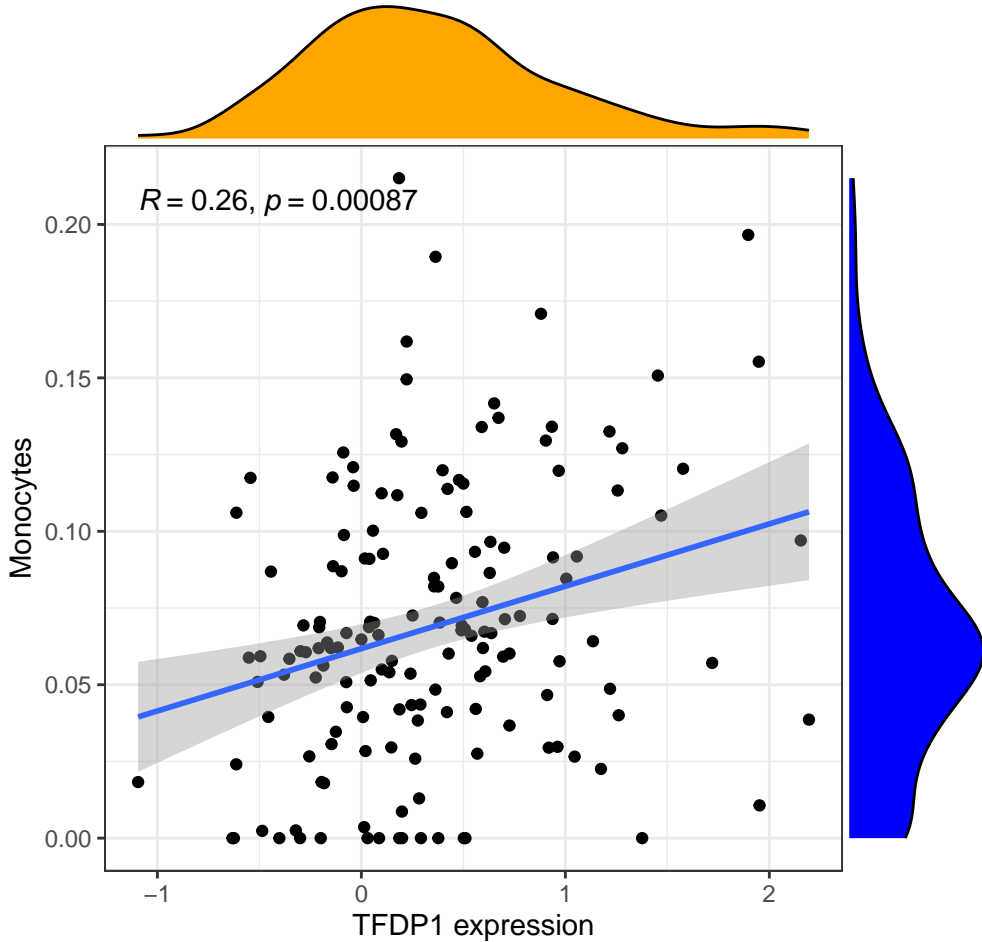

Supplement: Supplementary file 11 [file Data_Sheet_11.ZIP › 21.immuneCor/TFDP1/cor.Monocytes.pdf]

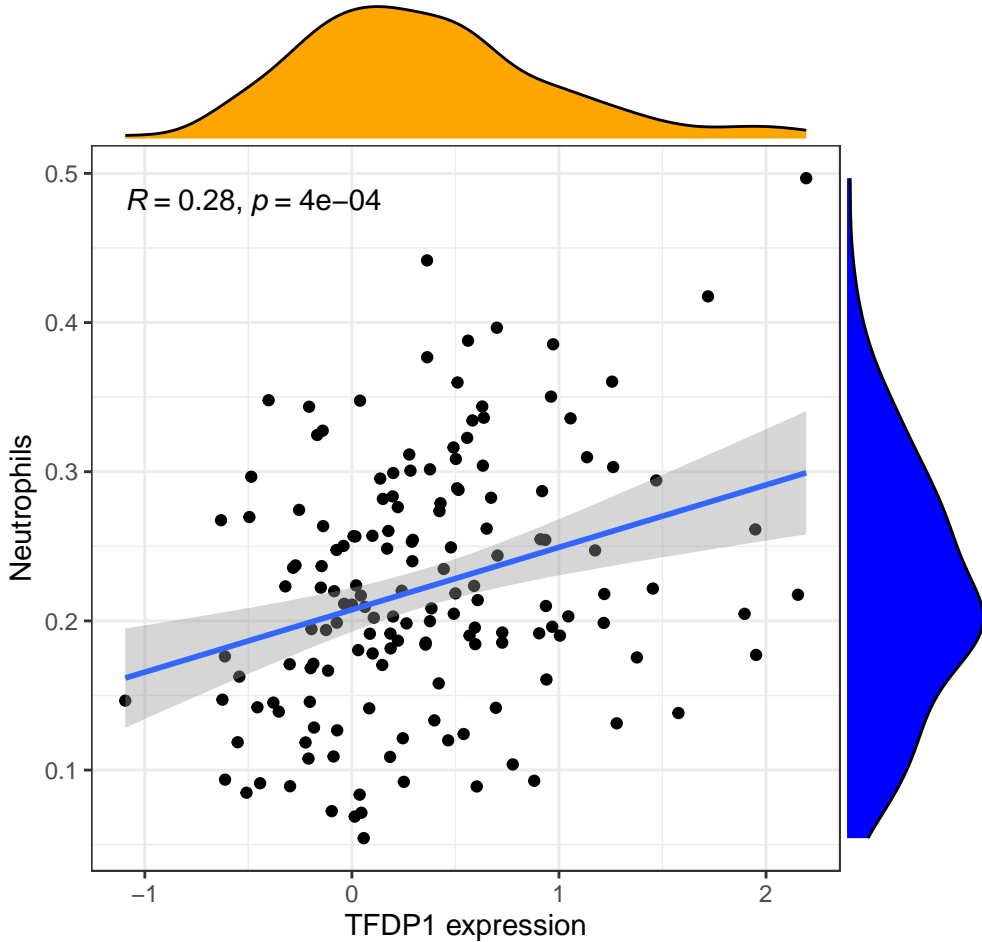

Supplement: Supplementary file 11 [file Data_Sheet_11.ZIP › 21.immuneCor/TFDP1/cor.Neutrophils.pdf]

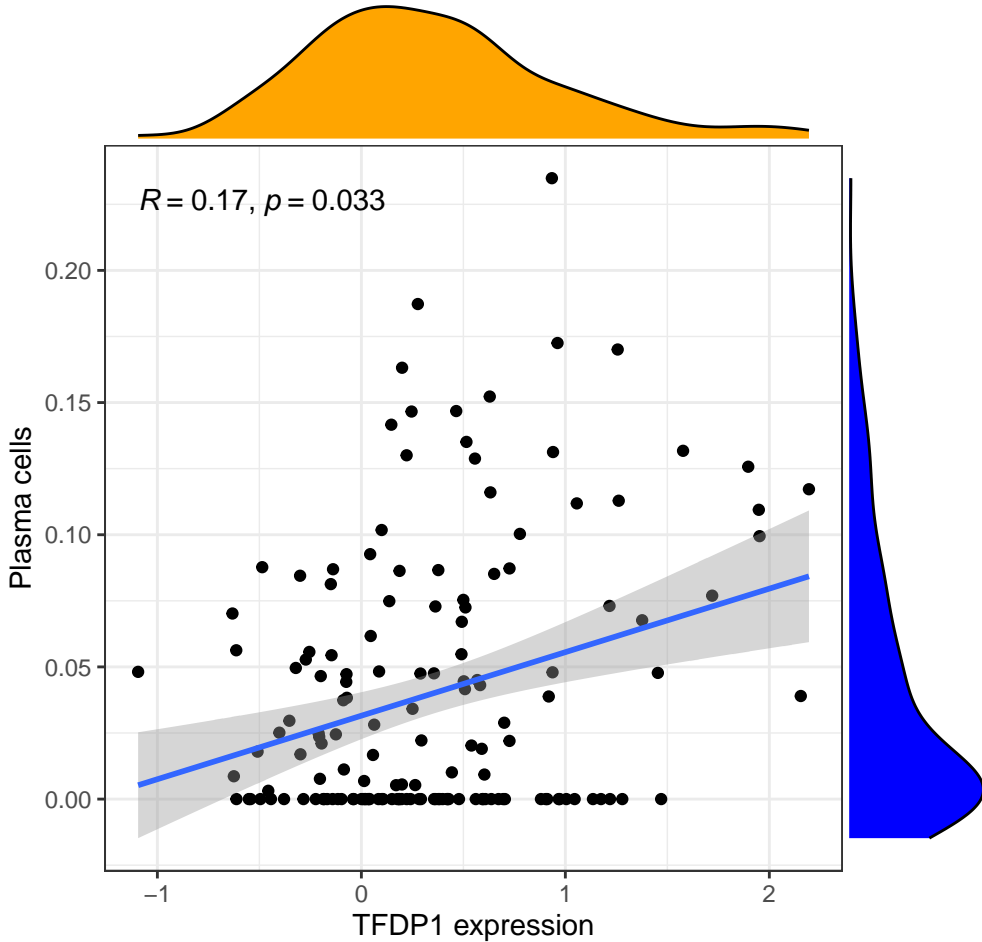

Supplement: Supplementary file 11 [file Data_Sheet_11.ZIP › 21.immuneCor/TFDP1/cor.Plasma cells.pdf]

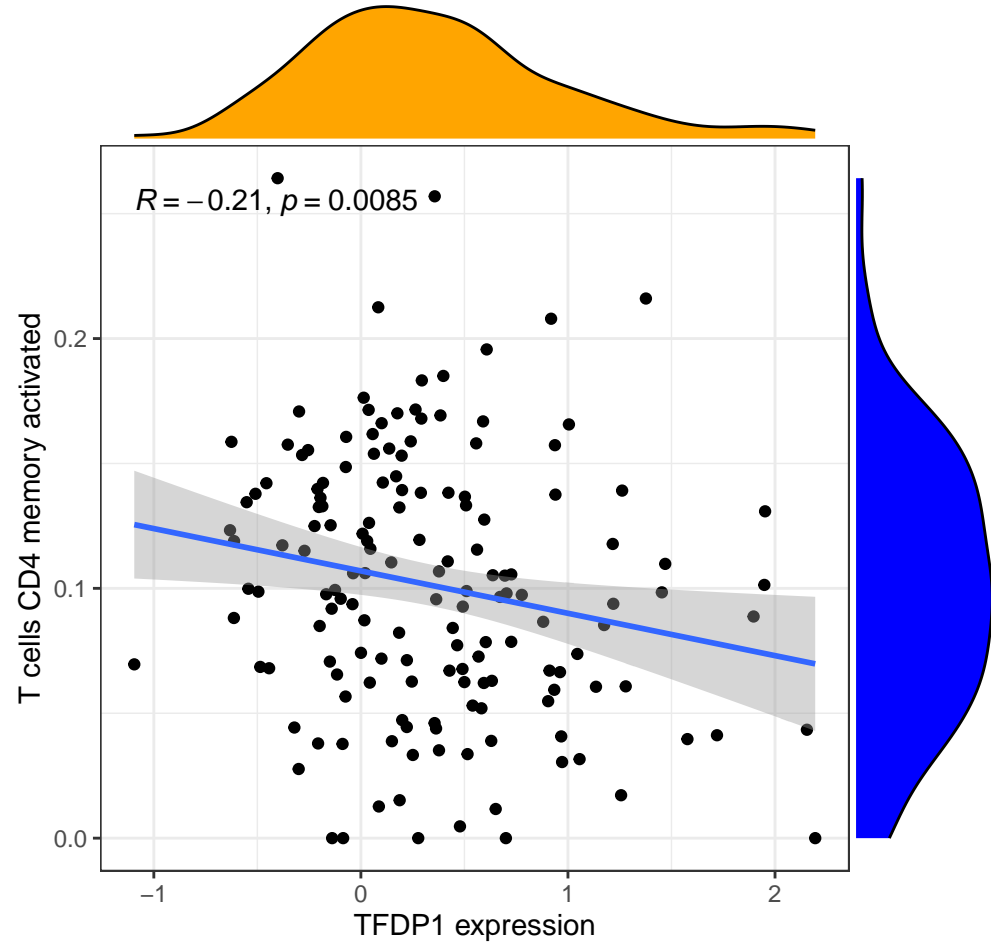

Supplement: Supplementary file 11 [file Data_Sheet_11.ZIP › 21.immuneCor/TFDP1/cor.T cells CD4 memory activated.pdf]

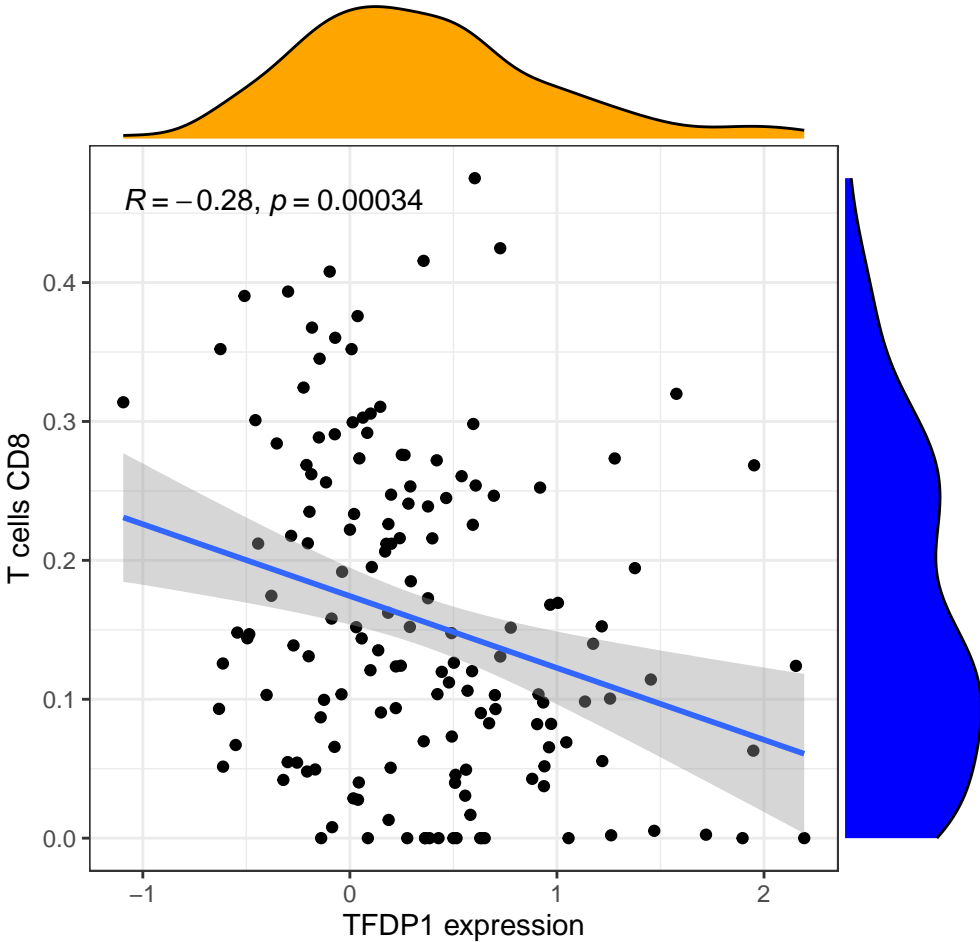

Supplement: Supplementary file 11 [file Data_Sheet_11.ZIP › 21.immuneCor/TFDP1/cor.T cells CD8.pdf]

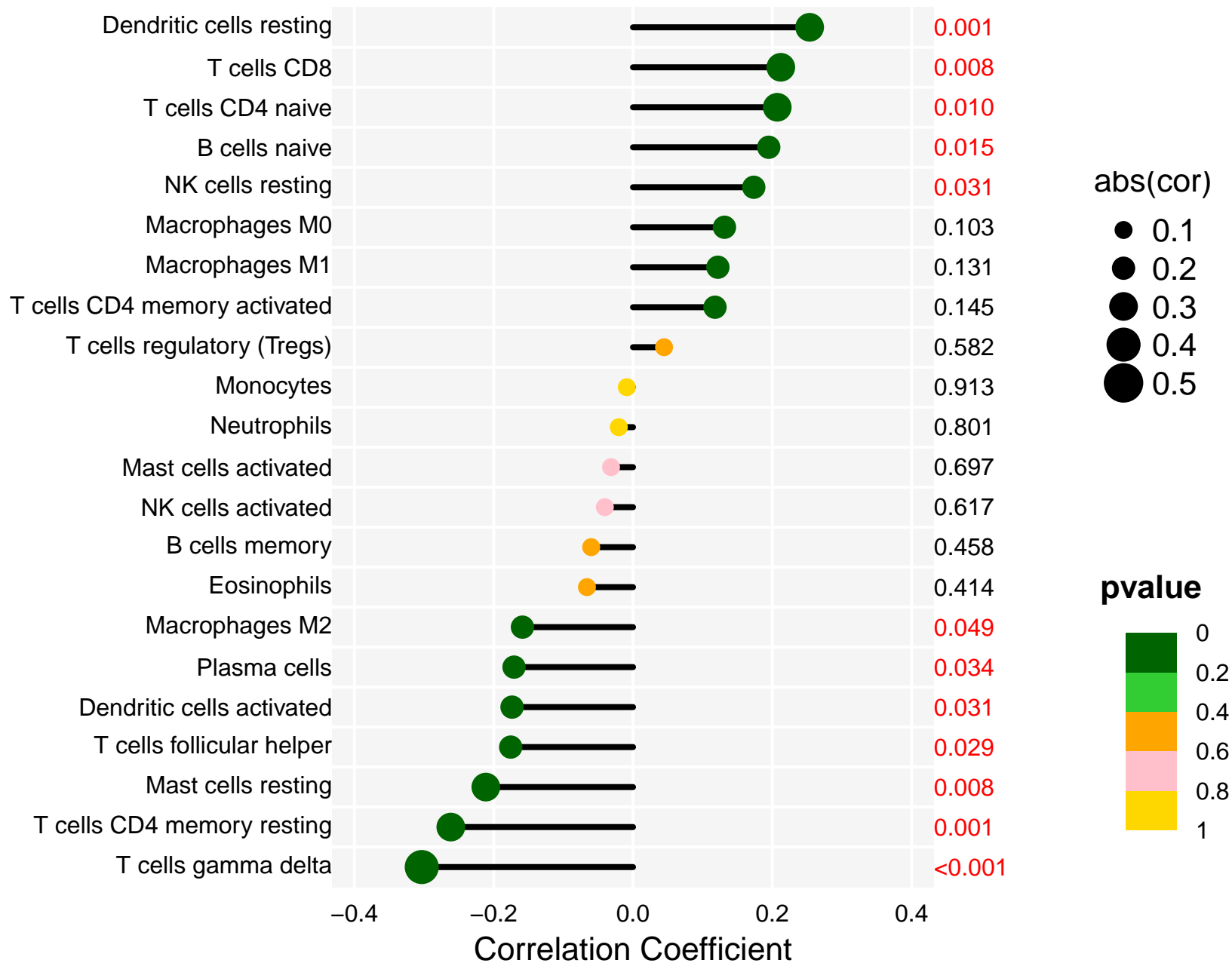

Supplement: Supplementary file 11 [file Data_Sheet_11.ZIP › 22.Lollipop/AKT1/Lollipop-AKT1.pdf]

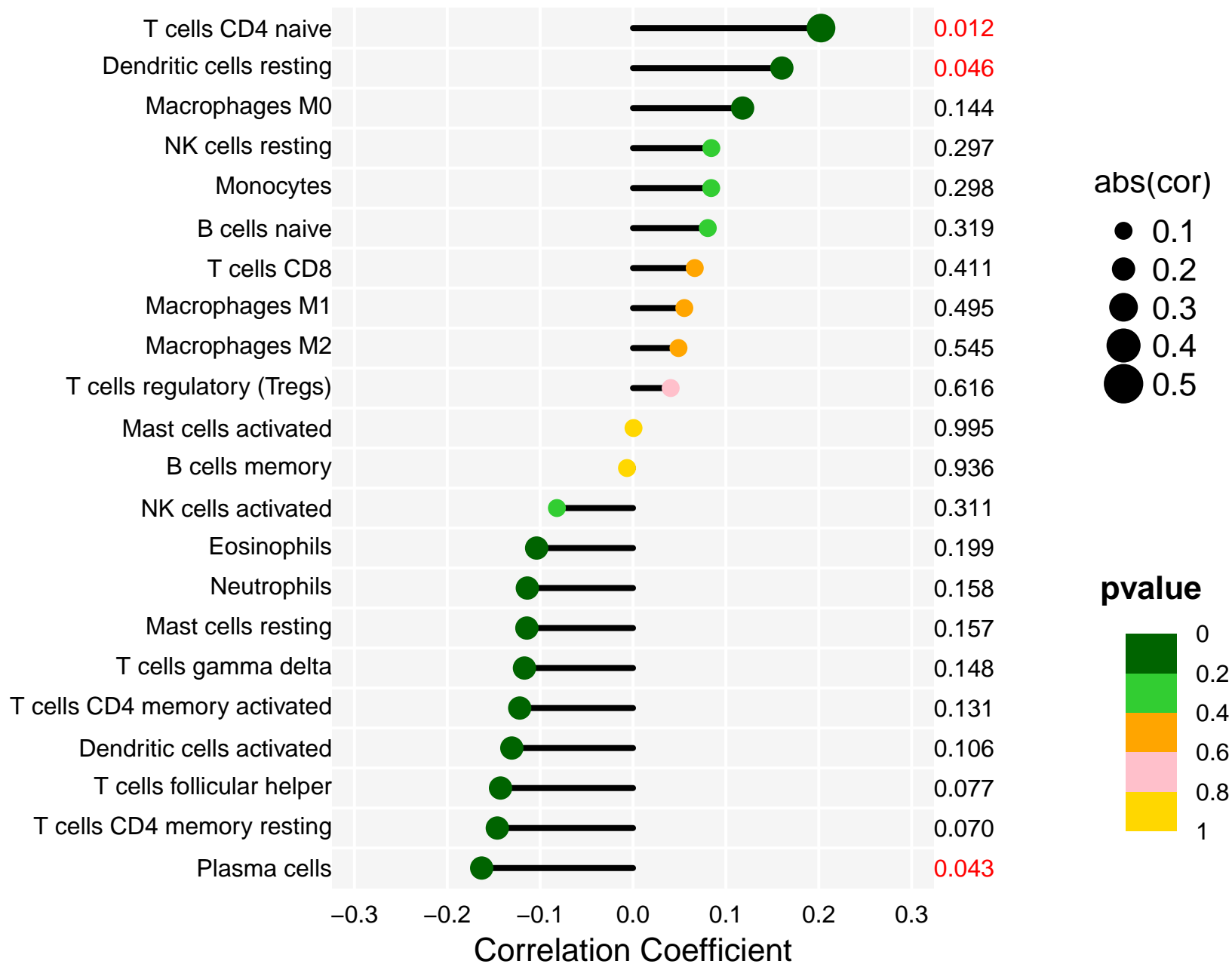

Supplement: Supplementary file 11 [file Data_Sheet_11.ZIP › 22.Lollipop/BRMS1/Lollipop-BRMS1.pdf]

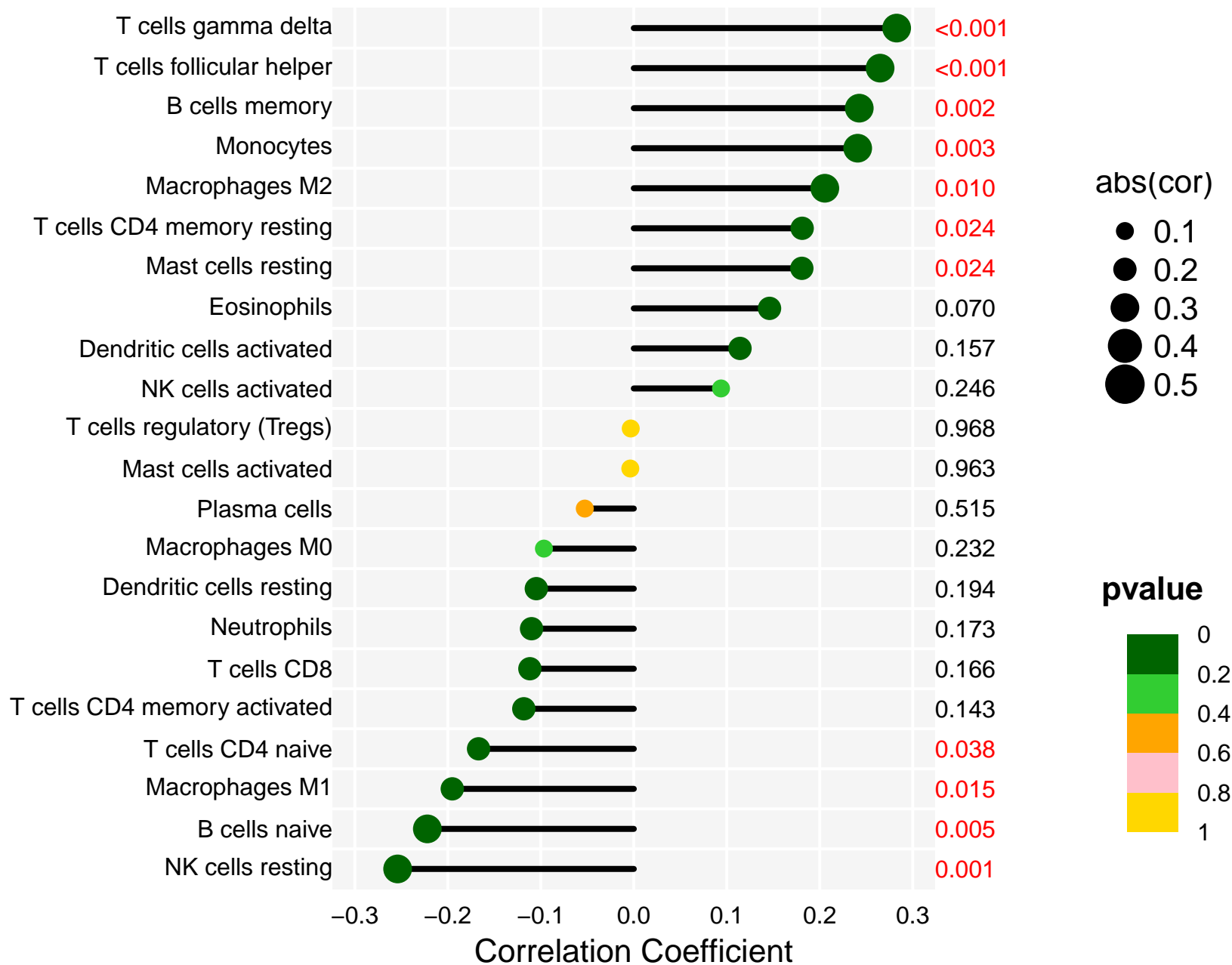

Supplement: Supplementary file 11 [file Data_Sheet_11.ZIP › 22.Lollipop/PTRH2/Lollipop-PTRH2.pdf]

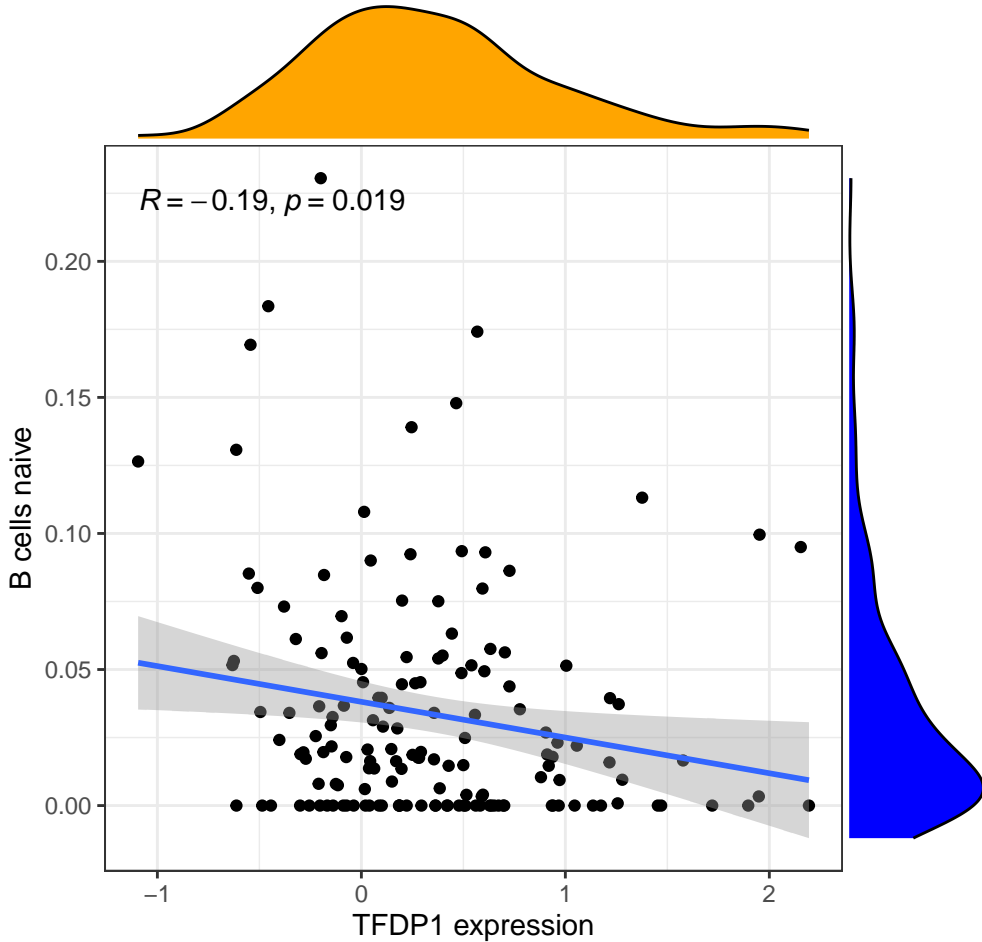

Supplement: Supplementary file 11 [file Data_Sheet_11.ZIP › 22.Lollipop/TFDP1/cor.B cells naive.pdf]

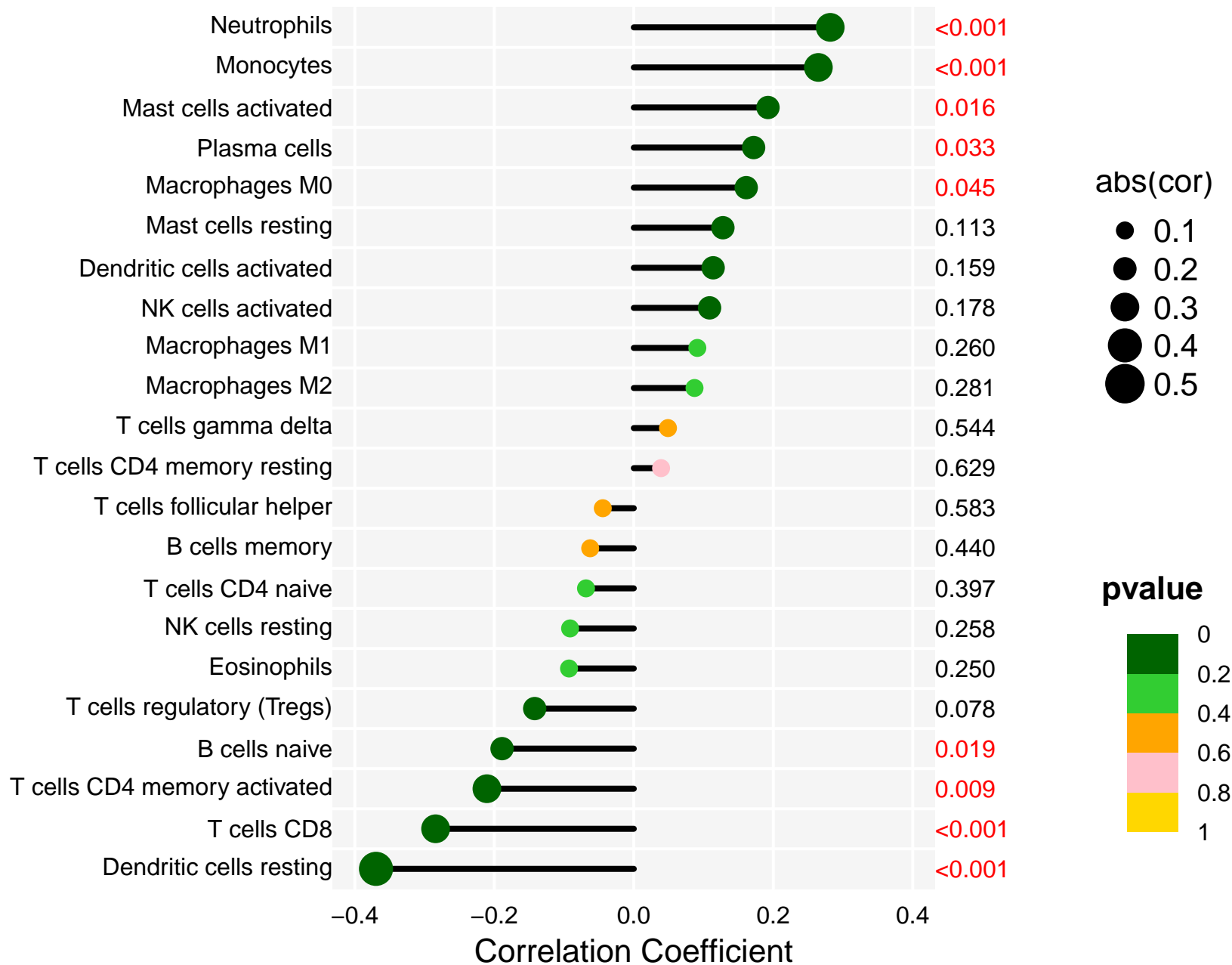

Supplement: Supplementary file 11 [file Data_Sheet_11.ZIP › 22.Lollipop/TFDP1/Lollipop-TFDP1.pdf]

abs(cor)

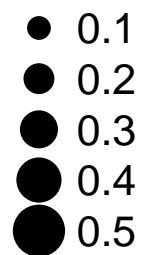

pvalue

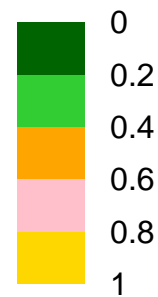

Supplement: Supplementary file 11 [file Data_Sheet_11.ZIP › 22.Lollipop/TLE1/Lollipop.pdf]

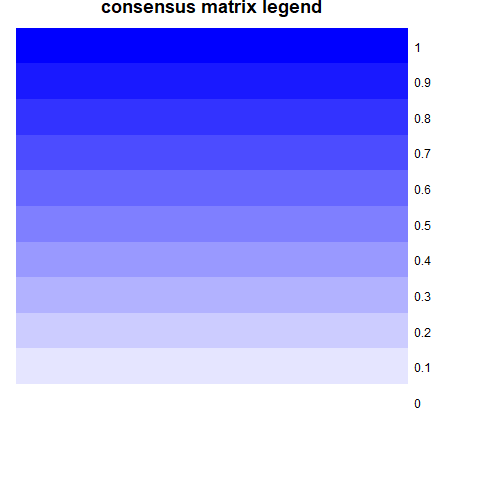

Supplement: Supplementary file 12 [file Data_Sheet_12.ZIP › Supplementary Figures 1/14.cluster-╤∙╞╖╖╓╨═/consensus001.png]

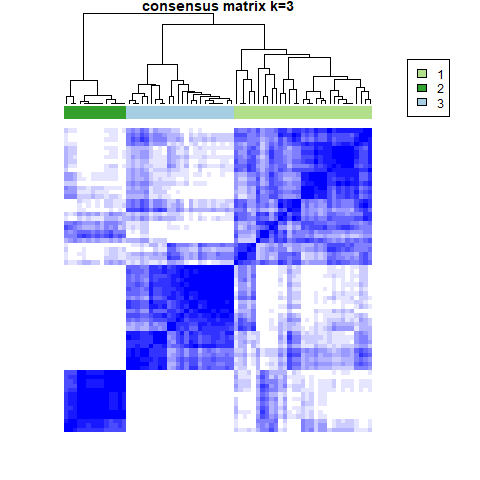

Supplement: Supplementary file 12 [file Data_Sheet_12.ZIP › Supplementary Figures 1/14.cluster-╤∙╞╖╖╓╨═/consensus003.png]

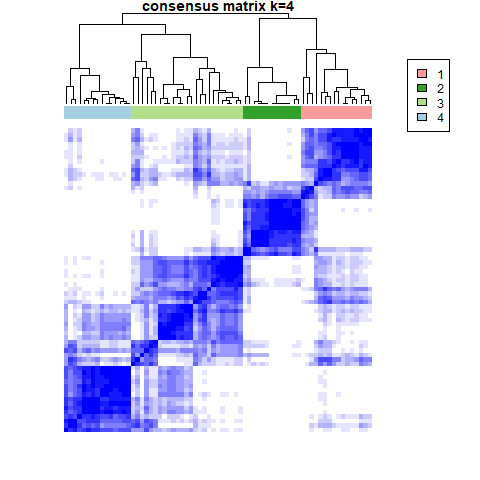

Supplement: Supplementary file 12 [file Data_Sheet_12.ZIP › Supplementary Figures 1/14.cluster-╤∙╞╖╖╓╨═/consensus004.png]

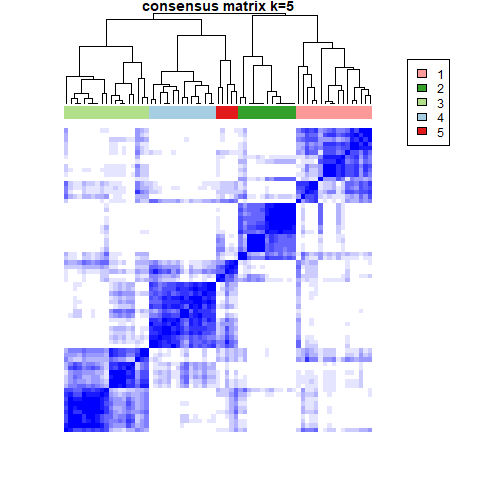

Supplement: Supplementary file 12 [file Data_Sheet_12.ZIP › Supplementary Figures 1/14.cluster-╤∙╞╖╖╓╨═/consensus005.png]

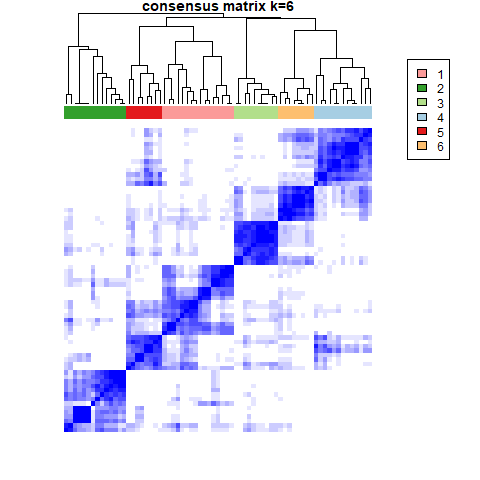

Supplement: Supplementary file 12 [file Data_Sheet_12.ZIP › Supplementary Figures 1/14.cluster-╤∙╞╖╖╓╨═/consensus006.png]

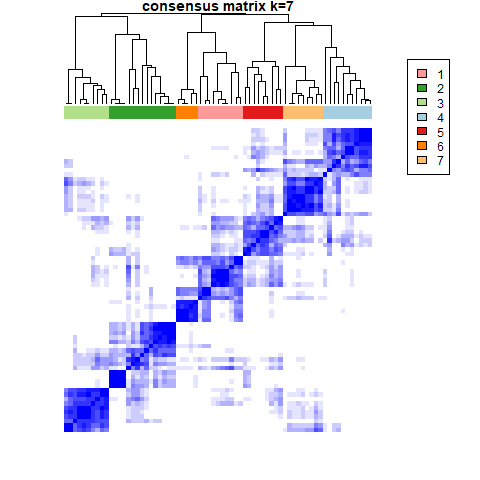

Supplement: Supplementary file 12 [file Data_Sheet_12.ZIP › Supplementary Figures 1/14.cluster-╤∙╞╖╖╓╨═/consensus007.png]

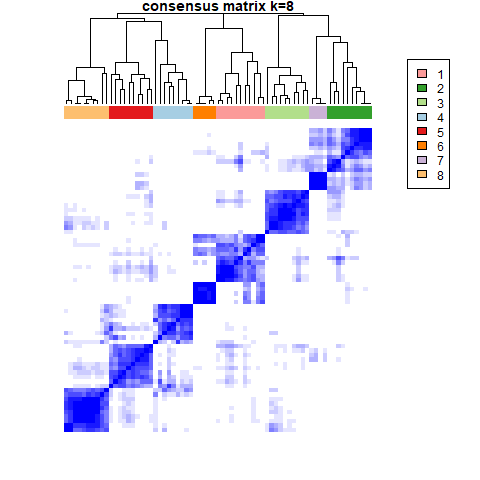

Supplement: Supplementary file 12 [file Data_Sheet_12.ZIP › Supplementary Figures 1/14.cluster-╤∙╞╖╖╓╨═/consensus008.png]

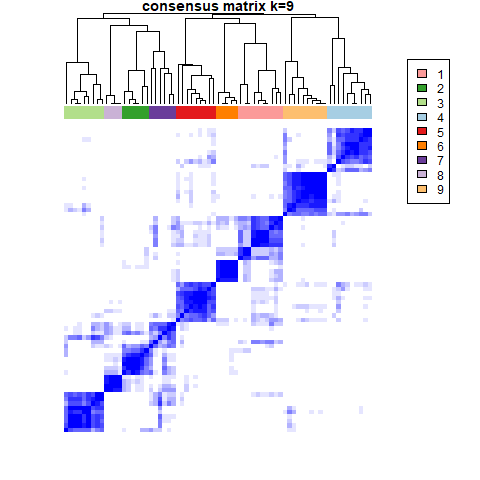

Supplement: Supplementary file 12 [file Data_Sheet_12.ZIP › Supplementary Figures 1/14.cluster-╤∙╞╖╖╓╨═/consensus009.png]

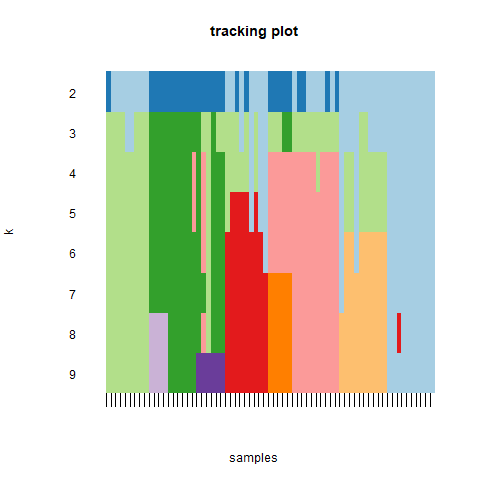

Supplement: Supplementary file 12 [file Data_Sheet_12.ZIP › Supplementary Figures 1/14.cluster-╤∙╞╖╖╓╨═/consensus012.png]

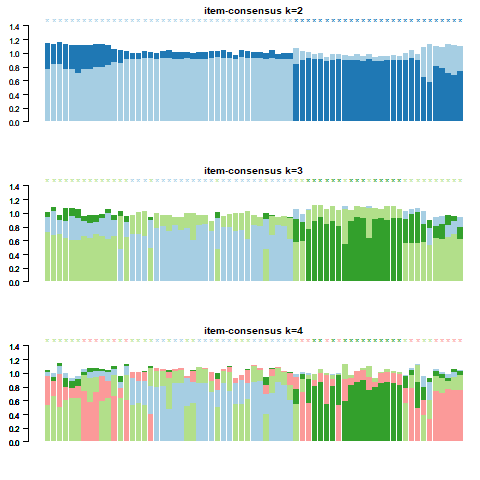

Supplement: Supplementary file 12 [file Data_Sheet_12.ZIP › Supplementary Figures 1/14.cluster-╤∙╞╖╖╓╨═/consensusScore/icl001.png]

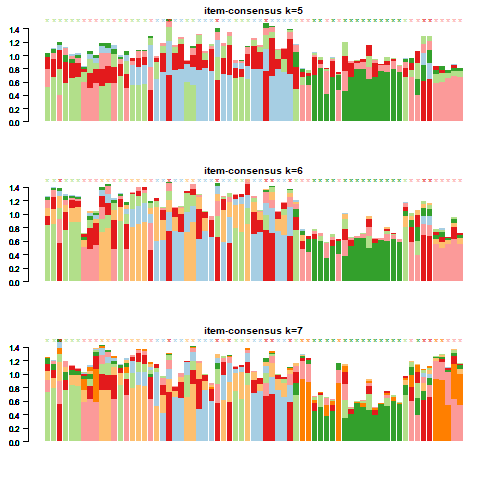

Supplement: Supplementary file 12 [file Data_Sheet_12.ZIP › Supplementary Figures 1/14.cluster-╤∙╞╖╖╓╨═/consensusScore/icl002.png]

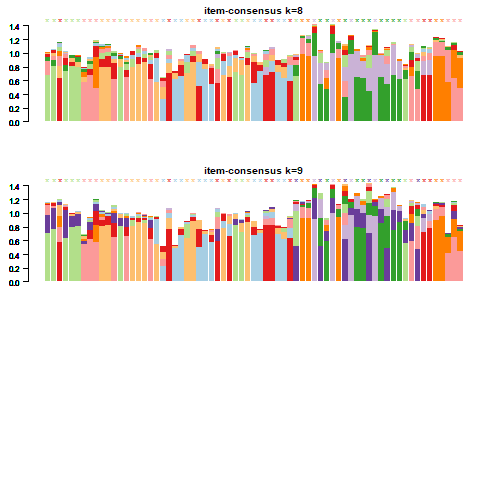

Supplement: Supplementary file 12 [file Data_Sheet_12.ZIP › Supplementary Figures 1/14.cluster-╤∙╞╖╖╓╨═/consensusScore/icl003.png]

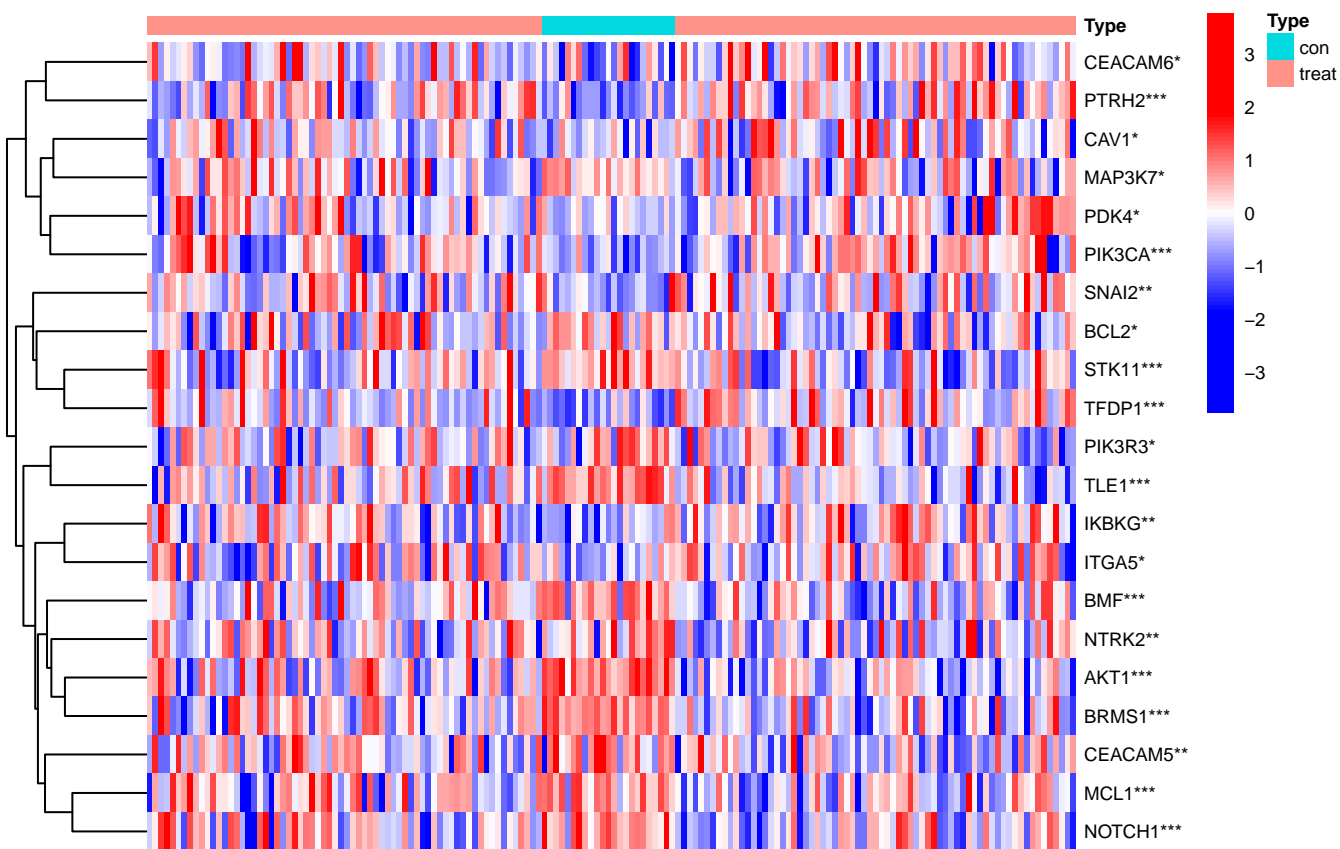

Supplement: Supplementary file 12 [file Data_Sheet_12.ZIP › Supplementary Figures 2/54.╤Θ╓ñ╤∙╞╖╖╓╨═HSE16561/07.diff/heatmap.pdf]

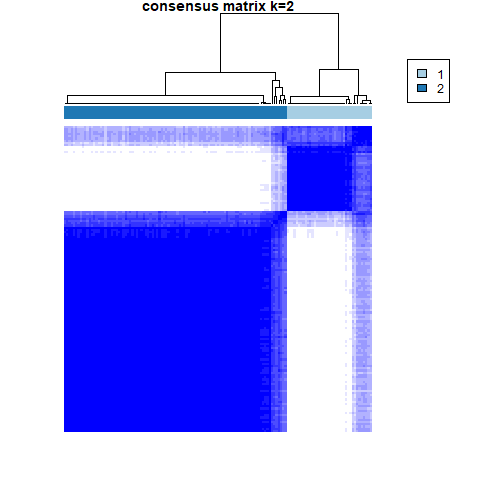

Supplement: Supplementary file 12 [file Data_Sheet_12.ZIP › Supplementary Figures 2/54.╤Θ╓ñ╤∙╞╖╖╓╨═HSE16561/14.cluster/consensus002.png]

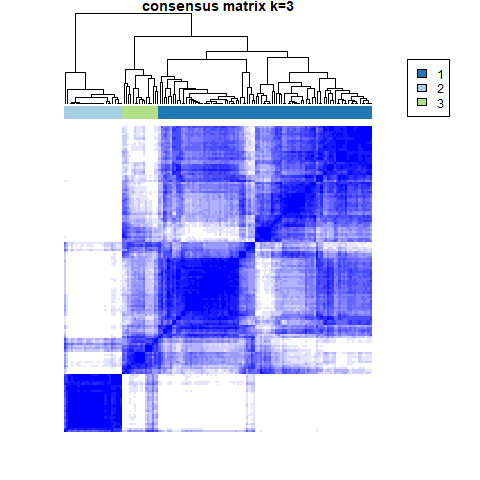

Supplement: Supplementary file 12 [file Data_Sheet_12.ZIP › Supplementary Figures 2/54.╤Θ╓ñ╤∙╞╖╖╓╨═HSE16561/14.cluster/consensus003.png]

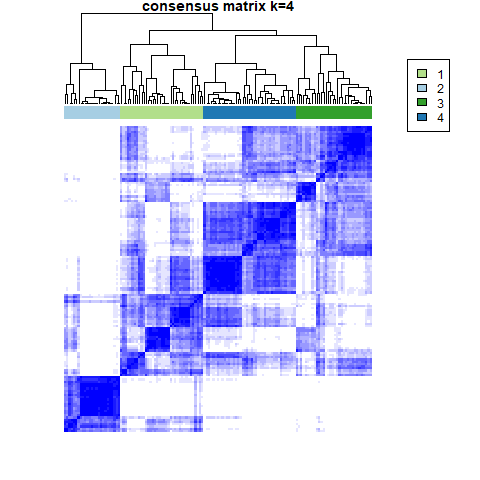

Supplement: Supplementary file 12 [file Data_Sheet_12.ZIP › Supplementary Figures 2/54.╤Θ╓ñ╤∙╞╖╖╓╨═HSE16561/14.cluster/consensus004.png]

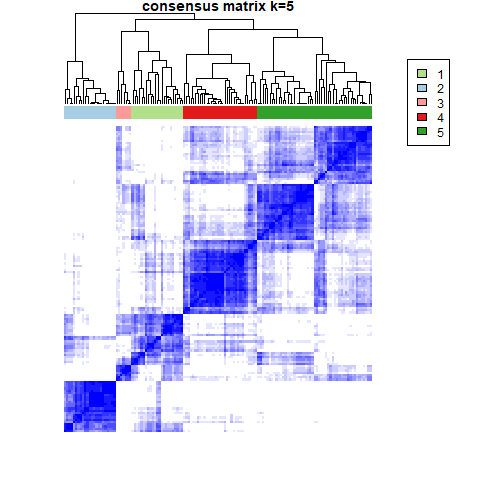

Supplement: Supplementary file 12 [file Data_Sheet_12.ZIP › Supplementary Figures 2/54.╤Θ╓ñ╤∙╞╖╖╓╨═HSE16561/14.cluster/consensus005.png]

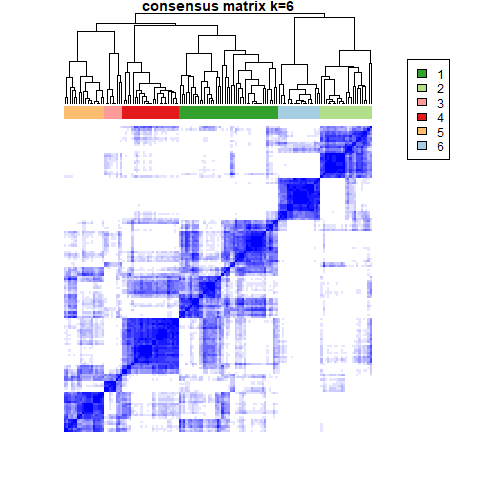

Supplement: Supplementary file 12 [file Data_Sheet_12.ZIP › Supplementary Figures 2/54.╤Θ╓ñ╤∙╞╖╖╓╨═HSE16561/14.cluster/consensus006.png]

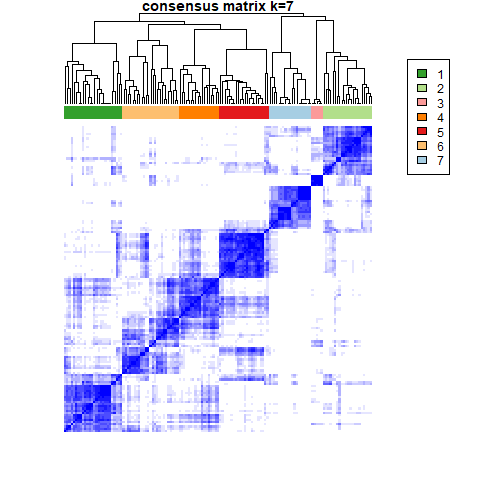

Supplement: Supplementary file 12 [file Data_Sheet_12.ZIP › Supplementary Figures 2/54.╤Θ╓ñ╤∙╞╖╖╓╨═HSE16561/14.cluster/consensus007.png]

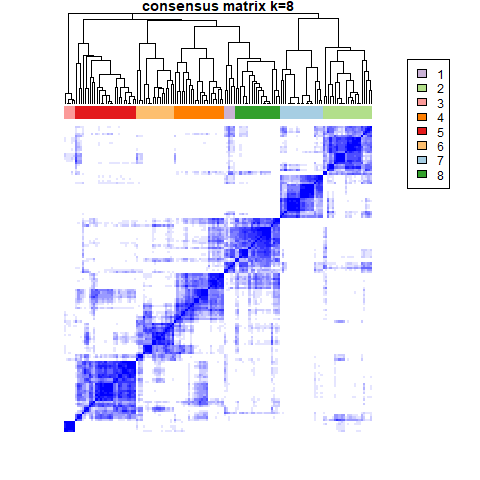

Supplement: Supplementary file 12 [file Data_Sheet_12.ZIP › Supplementary Figures 2/54.╤Θ╓ñ╤∙╞╖╖╓╨═HSE16561/14.cluster/consensus008.png]

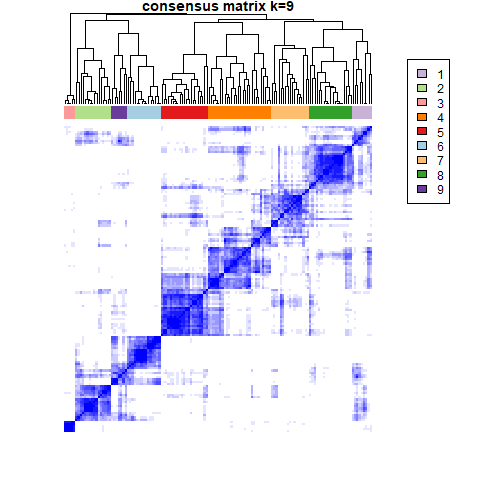

Supplement: Supplementary file 12 [file Data_Sheet_12.ZIP › Supplementary Figures 2/54.╤Θ╓ñ╤∙╞╖╖╓╨═HSE16561/14.cluster/consensus009.png]

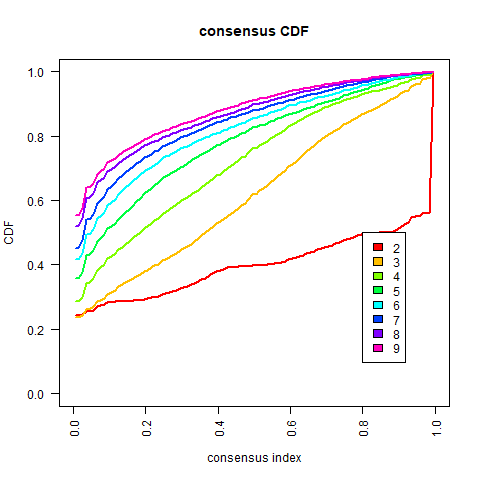

Supplement: Supplementary file 12 [file Data_Sheet_12.ZIP › Supplementary Figures 2/54.╤Θ╓ñ╤∙╞╖╖╓╨═HSE16561/14.cluster/consensus010.png]

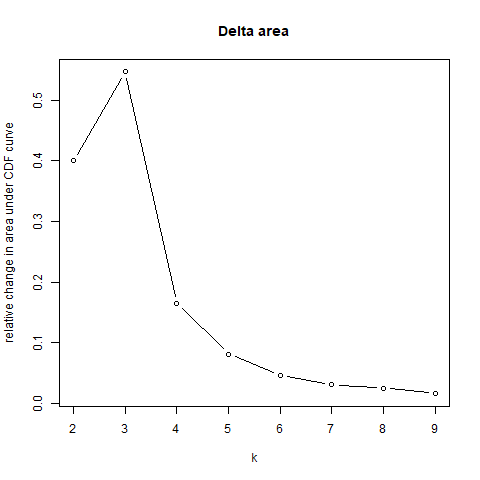

Supplement: Supplementary file 12 [file Data_Sheet_12.ZIP › Supplementary Figures 2/54.╤Θ╓ñ╤∙╞╖╖╓╨═HSE16561/14.cluster/consensus011.png]

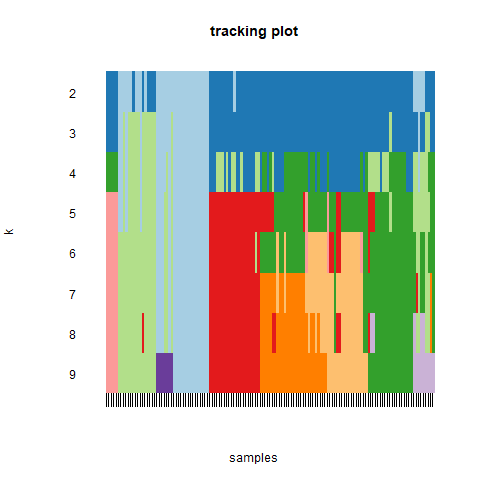

Supplement: Supplementary file 12 [file Data_Sheet_12.ZIP › Supplementary Figures 2/54.╤Θ╓ñ╤∙╞╖╖╓╨═HSE16561/14.cluster/consensus012.png]

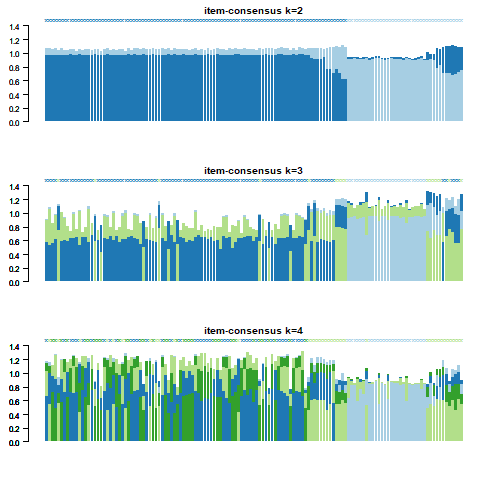

Supplement: Supplementary file 12 [file Data_Sheet_12.ZIP › Supplementary Figures 2/54.╤Θ╓ñ╤∙╞╖╖╓╨═HSE16561/14.cluster/consensusScore/icl001.png]

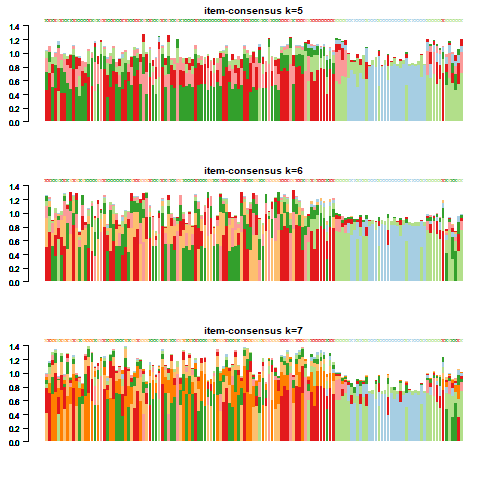

Supplement: Supplementary file 12 [file Data_Sheet_12.ZIP › Supplementary Figures 2/54.╤Θ╓ñ╤∙╞╖╖╓╨═HSE16561/14.cluster/consensusScore/icl002.png]

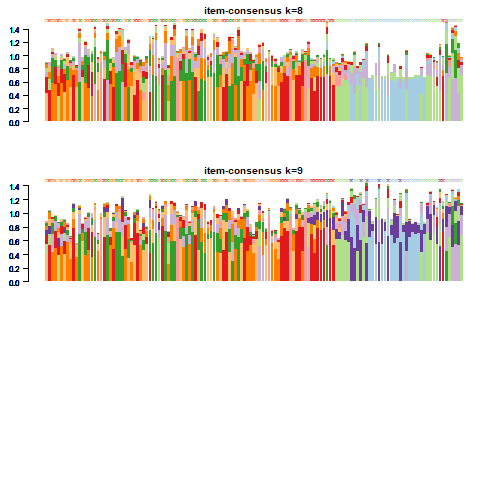

Supplement: Supplementary file 12 [file Data_Sheet_12.ZIP › Supplementary Figures 2/54.╤Θ╓ñ╤∙╞╖╖╓╨═HSE16561/14.cluster/consensusScore/icl003.png]

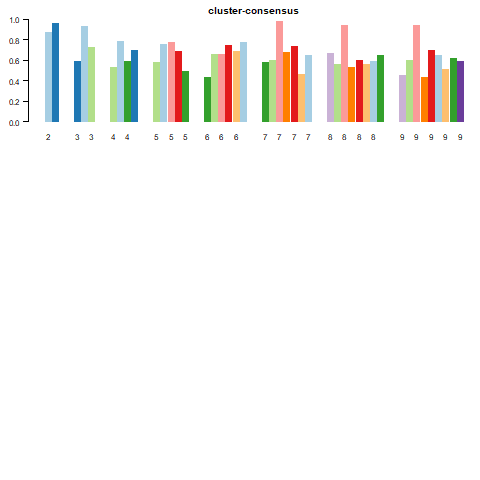

Supplement: Supplementary file 12 [file Data_Sheet_12.ZIP › Supplementary Figures 2/54.╤Θ╓ñ╤∙╞╖╖╓╨═HSE16561/14.cluster/consensusScore/icl004.png]

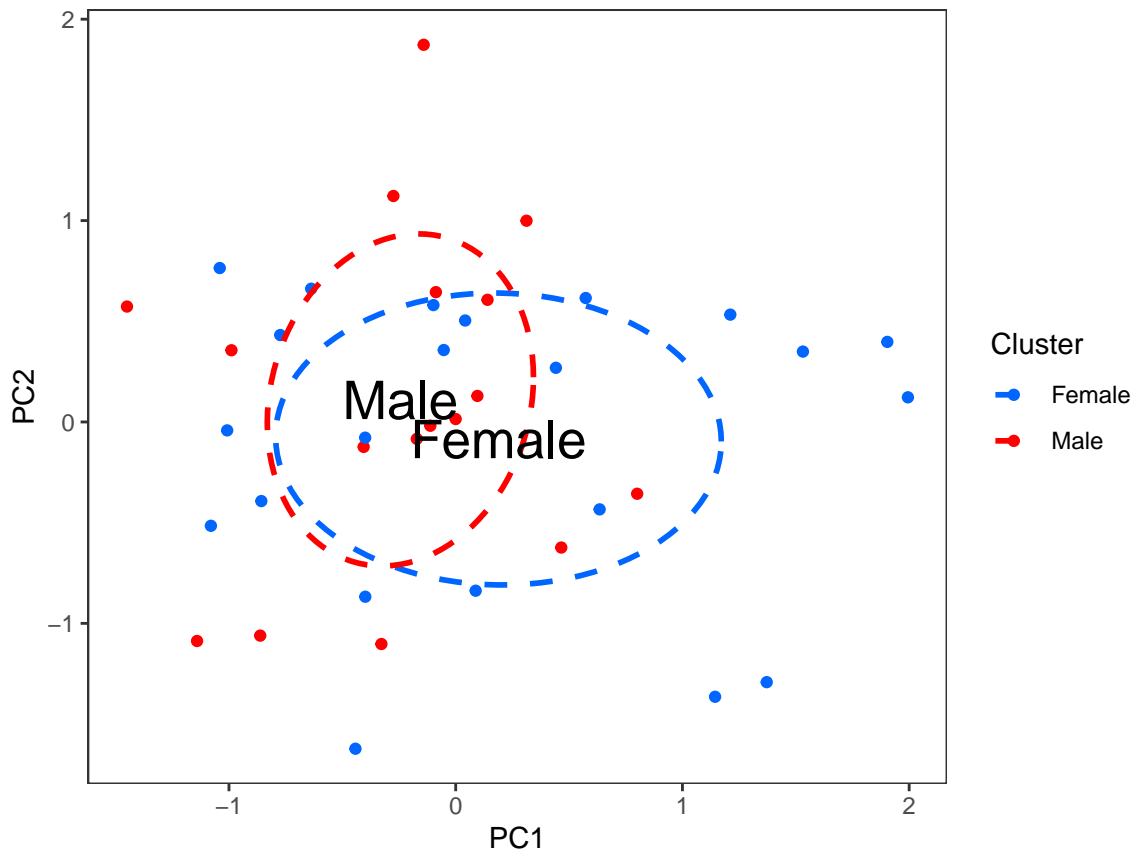

Supplement: Supplementary file 12 [file Data_Sheet_12.ZIP › Supplementary Figures 3/sex/16.PCA/PCA.pdf]

Cluster 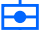 <=60 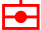 >60

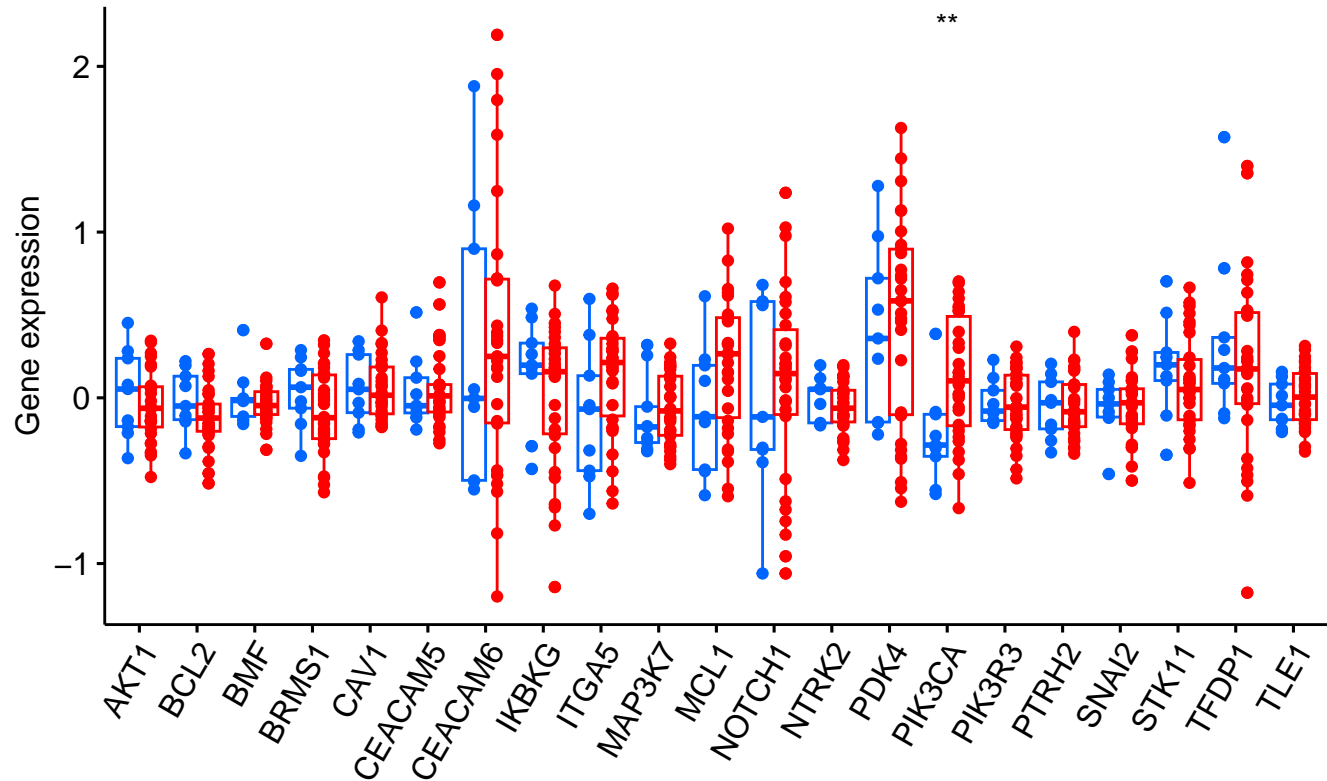

Supplement: Supplementary file 12 [file Data_Sheet_12.ZIP › Supplementary Figures 3/Supplementary Figures 3/boxplot-─Ω┴Σ.pdf]

Cluster 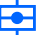 Female 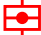 Male

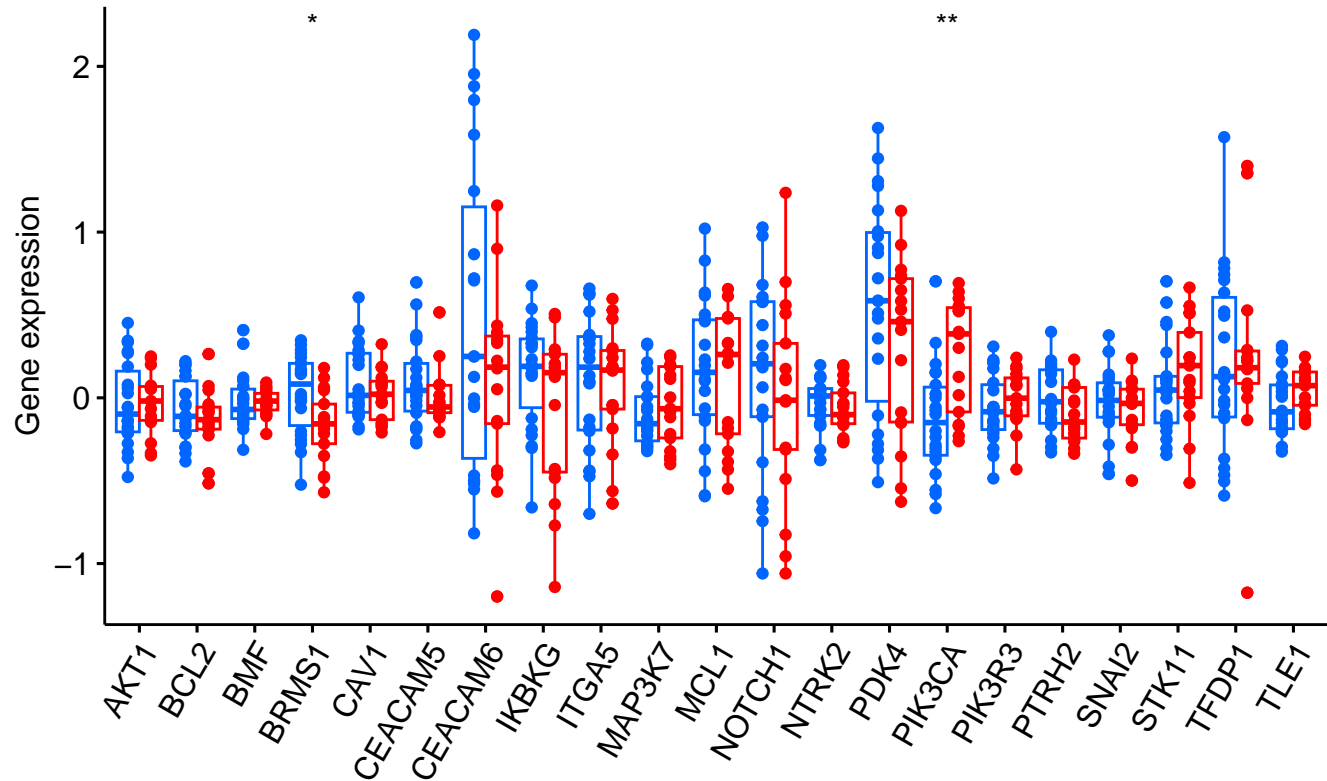

Supplement: Supplementary file 12 [file Data_Sheet_12.ZIP › Supplementary Figures 3/Supplementary Figures 3/boxplot-╨╘▒≡.pdf]

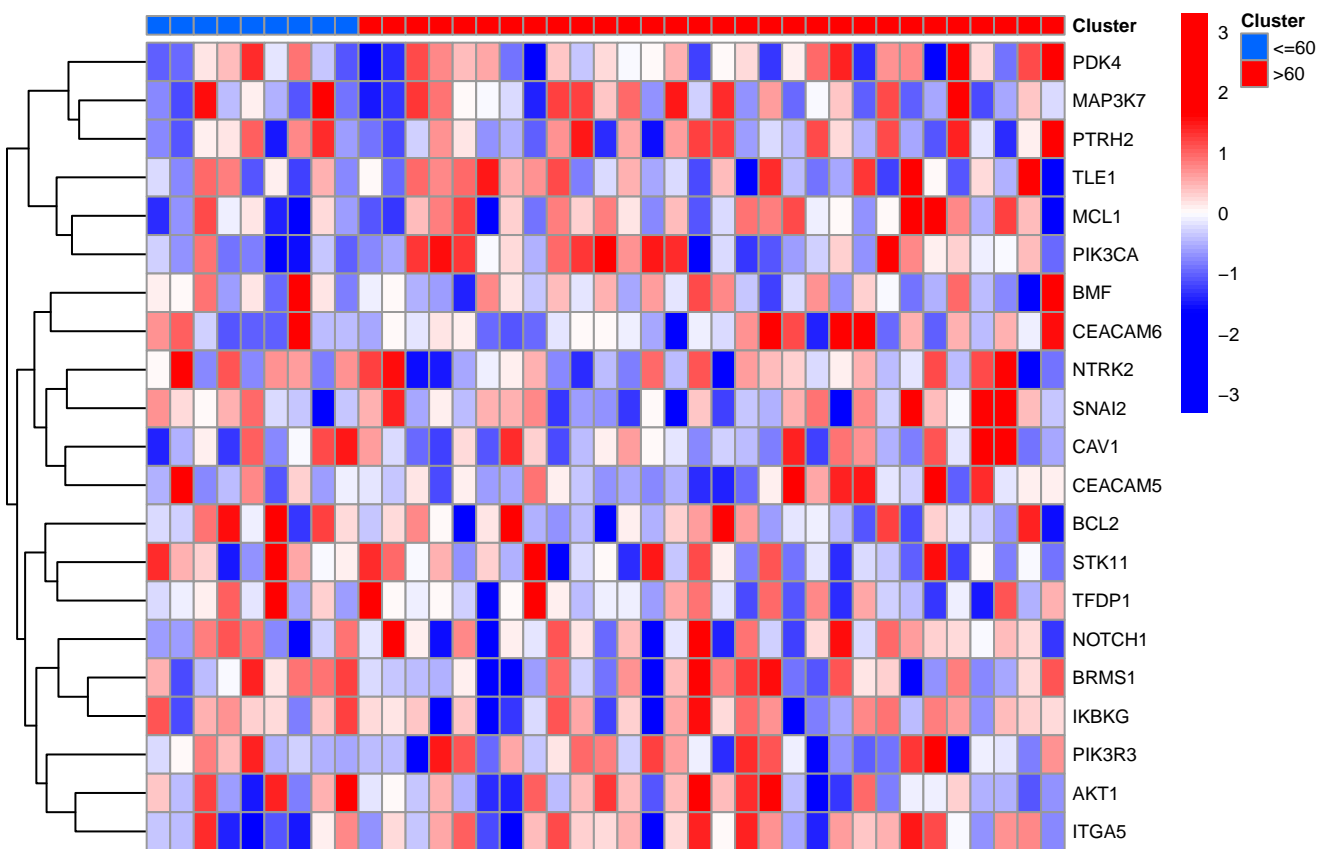

Supplement: Supplementary file 12 [file Data_Sheet_12.ZIP › Supplementary Figures 3/Supplementary Figures 3/heatmap-─Ω┴Σ.pdf]

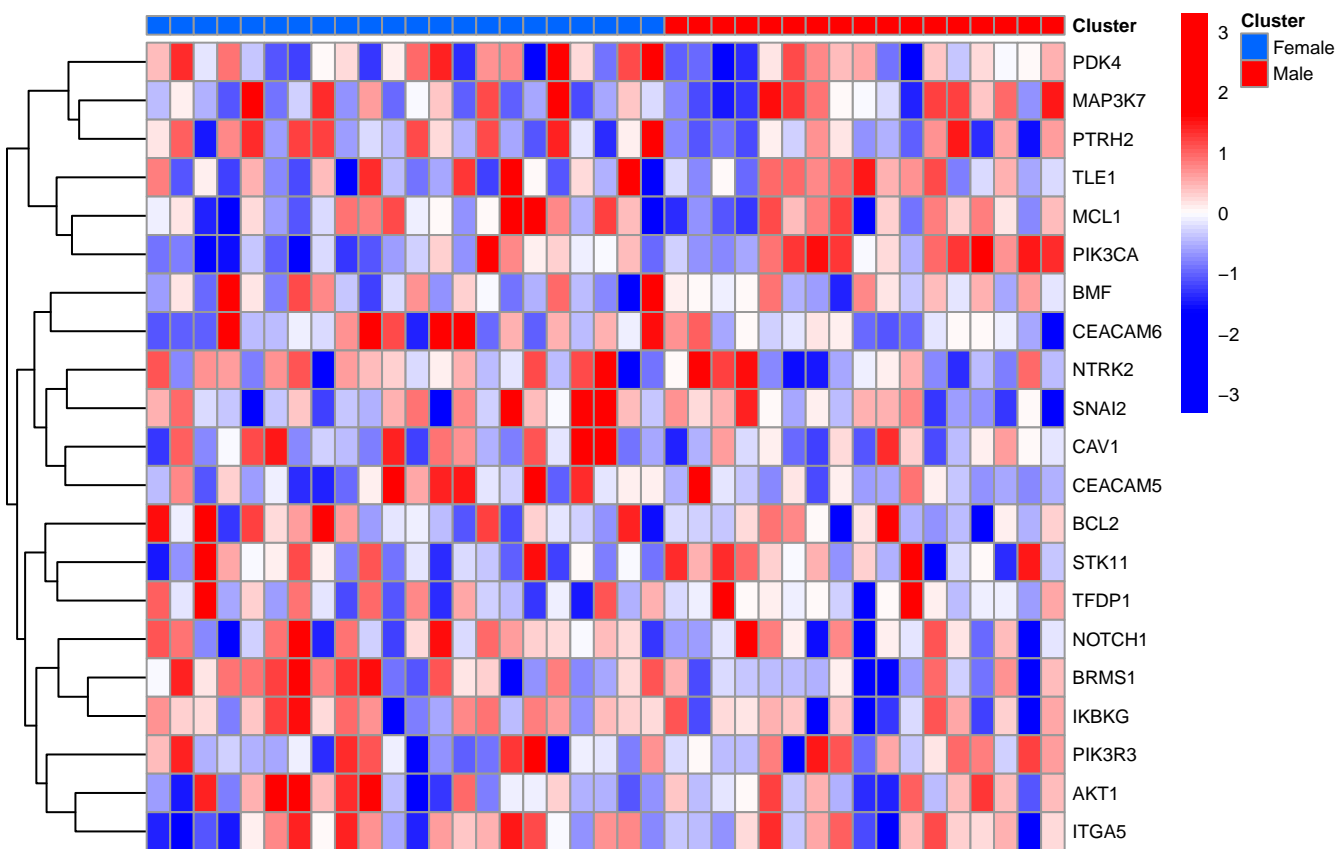

Supplement: Supplementary file 12 [file Data_Sheet_12.ZIP › Supplementary Figures 3/Supplementary Figures 3/heatmap-╨╘▒≡.pdf]

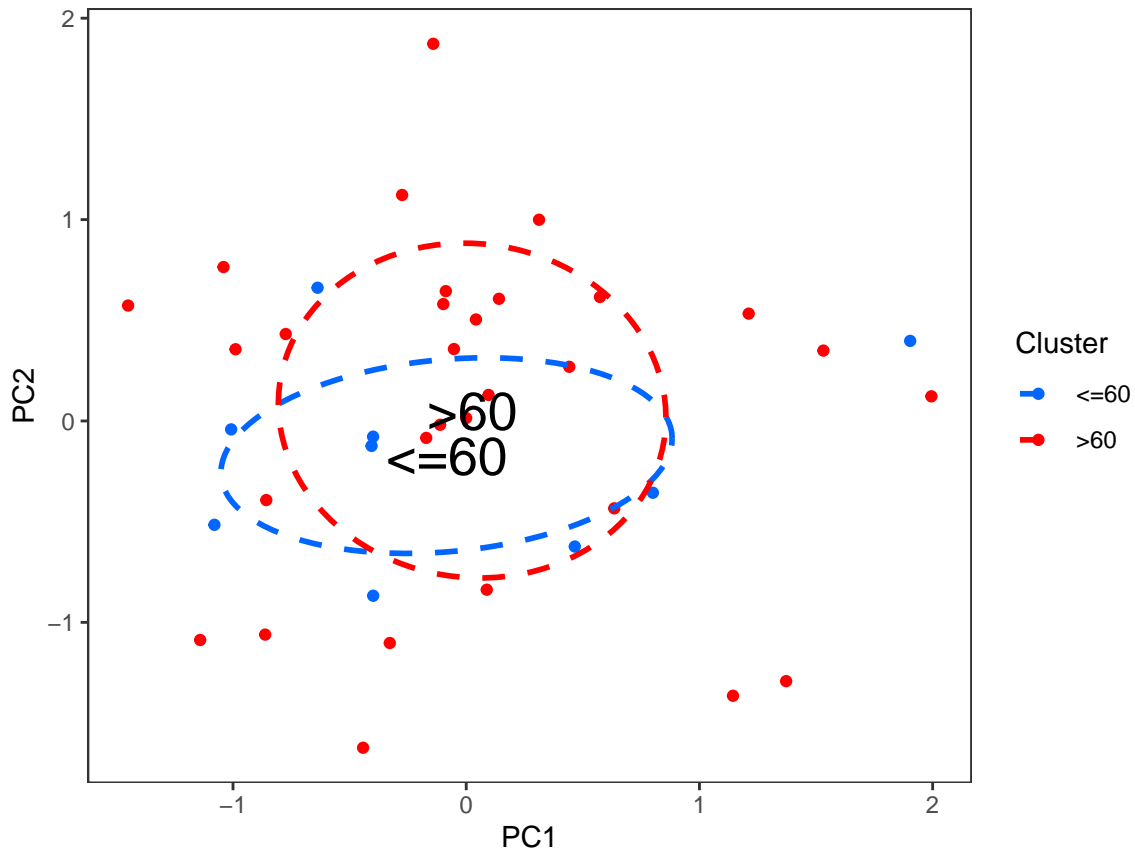

Supplement: Supplementary file 12 [file Data_Sheet_12.ZIP › Supplementary Figures 3/Supplementary Figures 3/PCA--─Ω┴Σ.pdf]
