# Supplementary material for: Identification of anoikis-related genes classification patterns and immune infiltration characterization in ischemic stroke based on machine learning
Source: Front Aging Neurosci. 2023 Mar 23;15:1142163. doi: 10.3389/fnagi.2023.1142163 (PMC10076550; doi:10.3389/fnagi.2023.1142163)
Supplement: Supplementary file 12 [file boxplot.pdf › Supplementary Figures 2/54.╤Θ╓ñ╤∙╞╖╖╓╨═HSE16561/07.diff/boxplot.pdf]

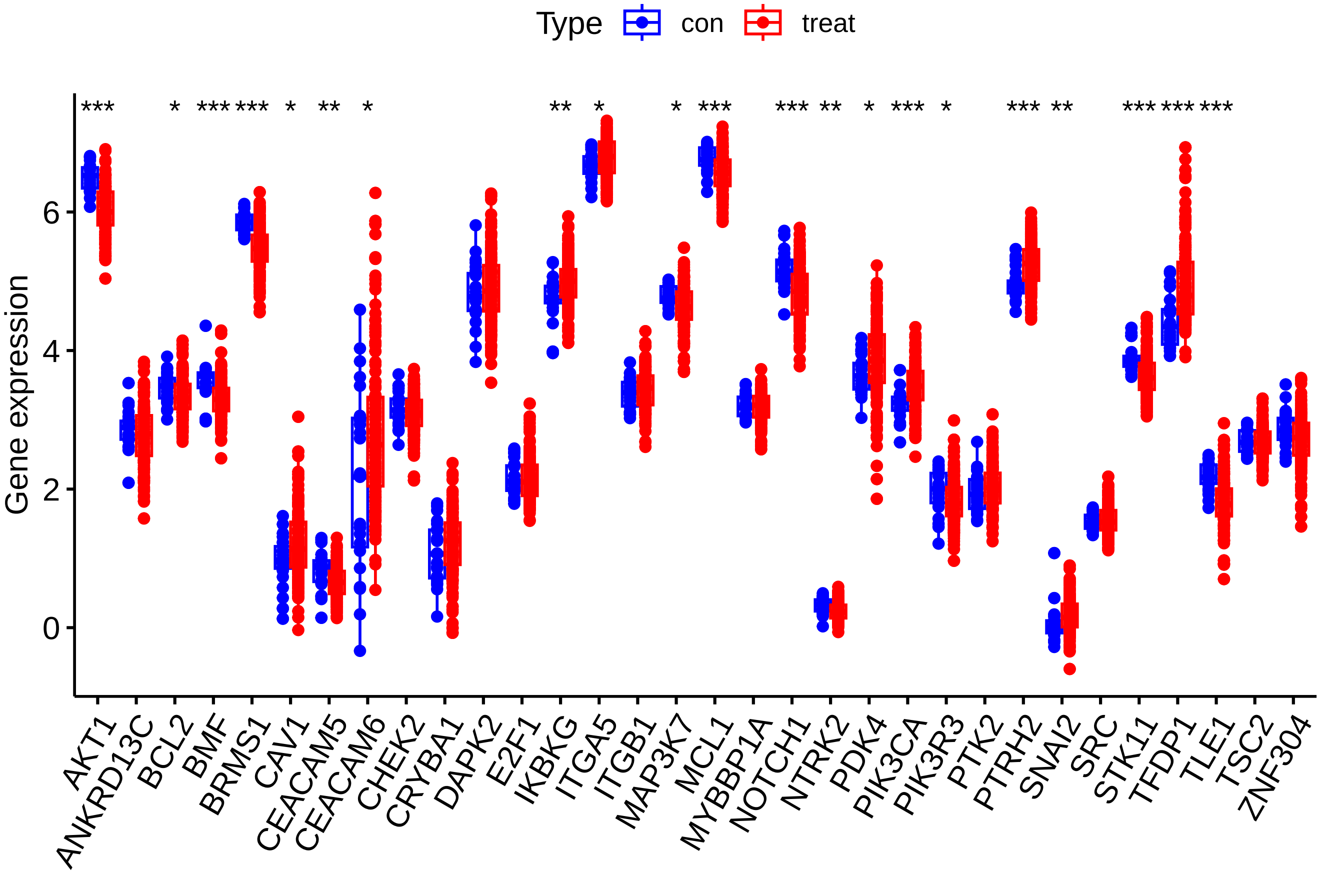

Type
con
treat
Gene expression
6
4
2
0
*** * *** *** * ** * ** * * *** *** ** * *** * *** ** *** *** ***
AKT1
ANKRD13C
BCL2
BMF
BRMS1
CAV1
CEACAM5
CEACAM6
CHEK2
CRYBA1
DAPK2
E2F1
IKBKG
ITGA5
ITGB1
MAP3K7
MCL1
MYBBP1A
NOTCH1
NTRK2
PDK4
PIK3CA
PIK3R3
PTK2
PTRH2
SNAI2
SRC
STK11
TFDP1
TLE1
TSC2
ZNF304
